# Single-cell sequencing of human midbrain reveals glial activation and a Parkinson-specific neuronal state

**DOI:** 10.1093/brain/awab446

**Published:** 2021-12-17

**Authors:** Semra Smajić, Cesar A. Prada-Medina, Zied Landoulsi, Jenny Ghelfi, Sylvie Delcambre, Carola Dietrich, Javier Jarazo, Jana Henck, Saranya Balachandran, Sinthuja Pachchek, Christopher M. Morris, Paul Antony, Bernd Timmermann, Sascha Sauer, Sandro L. Pereira, Jens C. Schwamborn, Patrick May, Anne Grünewald, Malte Spielmann

**Affiliations:** 1 Luxembourg Centre for Systems Biomedicine, University of Luxembourg, L-4362 Esch-sur-Alzette, Luxembourg; 2 Max Planck Institute for Molecular Genetics, D-14195 Berlin, Germany; 3 OrganoTherapeutics SARL-S, L-4362 Esch-sur-Alzette, Luxembourg; 4 Institute of Human Genetics, Kiel University, D-42118 Kiel, Germany; 5 Newcastle Brain Tissue Resource, Translational and Clinical Research Institute, Faculty of Medical Sciences, Newcastle University, NE1 7RU Newcastle upon Tyne, UK; 6 Max-Delbrück-Centrum für Molekulare Medizin, Genomics Group, D-13125 Berlin, Germany; 7 Institute of Neurogenetics, University of Lübeck, D-23562 Lübeck, Germany; 8 Institute of Human Genetics, University of Lübeck, D-23562 Lübeck, Germany

**Keywords:** Parkinson’s disease, midbrain substantia nigra, single-cell sequencing, microglia, neuroinflammation

## Abstract

Idiopathic Parkinson’s disease is characterized by a progressive loss of dopaminergic neurons, but the exact disease aetiology remains largely unknown. To date, Parkinson’s disease research has mainly focused on nigral dopaminergic neurons, although recent studies suggest disease-related changes also in non-neuronal cells and in midbrain regions beyond the substantia nigra. While there is some evidence for glial involvement in Parkinson’s disease, the molecular mechanisms remain poorly understood. The aim of this study was to characterize the contribution of all cell types of the midbrain to Parkinson’s disease pathology by single-nuclei RNA sequencing and to assess the cell type-specific risk for Parkinson’s disease using the latest genome-wide association study.

We profiled >41 000 single-nuclei transcriptomes of post-mortem midbrain from six idiopathic Parkinson’s disease patients and five age-/sex-matched controls. To validate our findings in a spatial context, we utilized immunolabelling of the same tissues. Moreover, we analysed Parkinson’s disease-associated risk enrichment in genes with cell type-specific expression patterns. We discovered a neuronal cell cluster characterized by *CADPS2* overexpression and low *TH* levels, which was exclusively present in idiopathic Parkinson’s disease midbrains. Validation analyses in laser-microdissected neurons suggest that this cluster represents dysfunctional dopaminergic neurons. With regard to glial cells, we observed an increase in nigral microglia in Parkinson’s disease patients. Moreover, nigral idiopathic Parkinson’s disease microglia were more amoeboid, indicating an activated state. We also discovered a reduction in idiopathic Parkinson’s disease oligodendrocyte numbers with the remaining cells being characterized by a stress-induced upregulation of *S100B*. Parkinson’s disease risk variants were associated with glia- and neuron-specific gene expression patterns in idiopathic Parkinson’s disease cases. Furthermore, astrocytes and microglia presented idiopathic Parkinson’s disease-specific cell proliferation and dysregulation of genes related to unfolded protein response and cytokine signalling. While reactive patient astrocytes showed *CD44* overexpression, idiopathic Parkinson’s disease microglia revealed a pro-inflammatory trajectory characterized by elevated levels of *IL1B*, *GPNMB* and *HSP90AA1*.

Taken together, we generated the first single-nuclei RNA sequencing dataset from the idiopathic Parkinson’s disease midbrain, which highlights a disease-specific neuronal cell cluster as well as ‘pan-glial’ activation as a central mechanism in the pathology of the movement disorder. This finding warrants further research into inflammatory signalling and immunomodulatory treatments in Parkinson’s disease.

## Teaser

To elucidate the contribution of individual cell types to Parkinson’s disease, Smajic *et al*. apply scRNA-seq to postmortem midbrain tissue. The results reveal a Parkinson’s disease-specific neuronal cluster, as well as changes in abundance, activation and an enrichment of Parkinson’s disease risk variants in glial cells.

See Lin and Narendra (https://doi.org/10.1093/brain/awac071) for a scientific commentary on this article.

## Introduction

Parkinson’s disease is a neurological disorder that is commonly characterized by a progressive loss of neuromelanin-containing dopaminergic neurons (DaNs) in the substantia nigra (SN).^1,2^ Age, genetic and environmental factors contribute to Parkinson’s disease pathogenesis, but disease pathology and aetiology remain mostly unknown.^3^ Approximately 95% of Parkinson’s disease patients do not harbour an interpretable genetic cause; therefore, they are classified as idiopathic Parkinson’s disease.^4^

So far Parkinson’s disease research has mainly focused on nigral dopaminergic neurons. By contrast, recent studies suggest disease-related changes also in non-neuronal cells and in brain regions beyond the SN. For instance, PET of drug-naive Parkinson’s disease patients revealed microglial activation of the entire brain.^5^ This finding is supported by histological analyses in post-mortem Parkinson’s disease tissue, which indicate microglial activation in the nigra but also in the putamen, hippocampus, and cortex.^6^ Reactive microglia can trigger the induction of neurotoxic reactive astrocytes,^7^ which, in turn, interfere with oligodendrocyte survival.^8^ Accordingly, glial pathology is suspected to drive neuroinflammatory processes throughout the brain, which contribute to neuronal demise in Parkinson’s disease.

The current understanding of neuron-glia cellular perturbations in Parkinson’s disease relies largely on experimental models that lack adequate representation of the disease complexity. For instance, toxin-induced animal models capture neither the nature of the human brain nor the multifactorial aspect of the disease.^9^ Also, induced pluripotent stem cell (iPSC) models derived from idiopathic Parkinson’s disease patients lack the complex cellular composition and dynamics found in a human brain. Several transcriptomic studies using human post-mortem midbrain tissue have investigated the transcriptional programs disrupted in idiopathic Parkinson’s disease. However, these studies used either bulk RNA-seq approaches on midbrain tissue or on laser capture-microdissected dopaminergic neurons, thereby failing to disentangle cell-type-specific contributions to the disease pathology.^10^ The recent development of single-cell sequencing technologies offers the possibility to overcome these challenges. In particular, transcriptional profiling of single cells (scRNA-seq) or nuclei (snRNA-seq) has proved itself to be an effective strategy to obtain a global view of disease-associated changes at an unprecedented resolution.^11^ Moreover, this single-cell approach can be linked to known disease-specific genetic variants to reveal disease trait association in specific cell types.

To address the above-described knowledge gaps and technical limitations, we performed snRNA-seq of post-mortem adult human midbrain tissue of idiopathic Parkinson’s disease patients and age-matched control subjects. Using this approach, we obtained an unbiased and global view of the cell type composition as well as the transcriptional programmes disrupted in idiopathic Parkinson’s disease glia and neurons at single-cell resolution.

## Materials and methods

### Human brain tissue cryosectioning

Frozen human post-mortem midbrain tissue sections and the associated clinical and neuropathological data were supplied by the Parkinson’s UK Brain Bank and the Newcastle Brain Tissue Resource. According to the neuropathological procedure, after removing the brainstem and cerebellum, the brain hemispheres were divided down the midline, with the hemi-midbrain associated with each hemisphere. The left hemi-midbrain was removed with a transverse section by taking a line from just behind the mammillary body through the superior colliculus. This midbrain block was then snap-frozen at −120°C and cryosectioned at ∼15 μm thickness in the transverse plane. The resulting sections were stored at −80°C.

Patients and control subjects gave written informed consent with the brain banks, which, together with the ethics review panel of the University of Luxembourg, approved the study.

### Sample preparation for nuclei isolation

Six to eight sections were combined from one individual for nuclei isolation. Nuclei were isolated by adapting the published 10X Genomics® protocol for ‘Isolation of Nuclei for Single Cell RNA Sequencing’. In brief, the tissue was lysed in a chilled lysis buffer (10 mM Tris-HCl, 10 mM NaCl, 3 mM MgCl_2_, 0,1% Nonidet™ P40). Then, the suspension was filtered and nuclei were pelleted by centrifugation. Nuclei-pellets were then washed in a ‘nuclei wash and resuspension buffer’ [1× PBS (phosphate-buffered saline), 1% BSA (bovine serum albumin), 0.2 U/μl RNase inhibitor], filtered, and pelleted again. Nuclei-pellets were suspended in the DAPI solution (1.5 μM DAPI in 1× PBS) and incubated for 5 min prior to FACS sorting. After dissociation, single DAPI-positive nuclei were filtered by size and granularity using a FACSDiva Cell Sorter (BD Biosciences) to minimize the amount of cell debris in the suspension. The resulting nuclei were inspected under the microscope. Only those that appeared intact were considered when adjusting the nuclei concentration prior to loading of the sequencer.

### Library preparation and sequencing

Sorted nuclei were processed using the Chromium Next GEM Single Cell 3′ Kit v3.1 to generate the cDNA libraries. The quality of cDNA was assessed using the Agilent 2100 Bioanalyzer System. Sequencing was performed on Illumina NovaSeq 6000-S2.

### Transcript quantification and filtering

FASTQ files were generated from the raw base call (BCL) outputs with the Cell Ranger (10× Genomics) *mkfastq* pipeline v.3.0. From this, we obtained a gene-barcode UMI count matrix per sample using the Cell Ranger (10× Genomics) count pipeline v.3.0 using default parameters. The Cell Ranger count pipeline only considers exon-mapping reads during UMI-counting. Also, single-nuclei sequencing readouts are enriched in intronic regions. To account for this, we used the Cell Ranger recommended variation of the human reference transcriptome (hg38), where introns are annotated as exons. The CellRanger pipeline predicted 51 929 barcodes to represent intact single nuclei across all samples, from which 10 494 were filtered out. We retained barcodes with >1500 UMIs and >1000 genes, as well as <10% of mitochondrial-encoded (mtDNA) and <10% of ribosomal gene counts. We only kept genes that were detected in at least three barcodes. Next, we removed ribosomal and mtDNA-encoded genes from the count matrix. We then used Scrublet^12^ to identify potential multiplet-barcodes, and only kept barcodes with an estimated doublet score <0.15 for downstream analysis.

### Normalization, sample integration and cell clustering

To identify the major cell types comprising the human midbrain, we combined the samples in a single embedding following the Seurat v3^13^ CCA integration workflow. First, each sample was normalized using the SCTransform approach.^14^ Cell-cycle phase assignment was performed based on this normalized expression matrix. We used the Seurat *CellCycleScoring* function and the Seurat v3 reference genes for the S and G2/M cell-cycle phases. To determine the inter-sample anchors for integration, we used the *FindIntegrationAnchors* Seurat function with the top 4000 consistent highly variable genes across the samples, identified with the *SelectIntegrationFeatures* function. We then used the *IntegrateData* Seurat function to obtain a combined and centred expression matrix. Principal component analysis was carried out on this centred expression matrix. The top 25 principal components were used to build a shared nearest neighbour (SNN) cell graph, which was then clustered using the Louvain algorithm (resolution = 1.5) implemented in the Seurat *FindClusters* function. The top 25 principal components were embedded onto two dimensions using the Uniform Manifold Approximation and Projection (UMAP) algorithm with the number of neighbours set to 30 and a minimum distance set to 0.3 following Seurat3 default implementation.^15^ We identified marker genes for each cluster by using the ROC method of the Seurat *FindAllMarkers* function. The top marker genes were used to assign cell type annotations manually for each cell cluster. We compared the cell types by correlating their pseudo bulk profiles. The resulting gene-cell type matrix was normalized (transcript per million) and log2 transformed. The Pearson correlation estimates among the normalized cell type profiles were used as the input distance matrix for hierarchical clustering.

### Machine learning cross-validation of cell-type annotation

To quantitatively validate the cell-cluster definition and annotation, we implemented a stratified cross-validation machine learning approach. Briefly, we removed the sample effects on the combined UMI count dataset using Harmony.^16^ For normalization, we used the loess transform^17^ to fit a smooth curve between mean and variance using the log-transformed data. We then scaled the data with the fitted mean and standard deviation (SD). The identified marker genes (Supplementary Table 3) were selected as the features of the model. We considered each cell type’s median cell type to subsample the dataset as a few cell types (DaNs and CADPS2^high^) were under-represented. To ensure similar label composition in the training and test sets, we split the data using scikit-learn^18^*StratifiedKFold* with 70% of the data as training and 30% as test dataset 5-fold cross-validation. We performed dimensionality reduction with *truncatedSVD* to 30 components. These 30 components were classified based on scikit-multilearn’s ensemble classification,^19^ which uses Louvain-based clustering^20,21^ and a random forest classification to account for the clustered and sparse nature of the snRNA-seq data. The predicted cell types were then compared to the manually curated cell label assignments using a confusion matrix.

### Differential cell-type composition

We estimated the differential cell type composition by comparing the UMAP embeddings and the cell type proportions between the idiopathic Parkinson’s disease and control samples. We considered the two-dimensional kernel cell density of the idiopathic Parkinson’s disease and control cells independently on the first two UMAP components using the *kde2d* function (bins = 100) implemented in the *MASS* R package.^22^ The idiopathic Parkinson’s disease log2 differential UMAP density was calculated. Also, for each cell type, we compared the proportion of cells per sample between the idiopathic Parkinson’s disease patients and control individuals. We assessed this difference with the Student’s *t*-test implemented in the *t.test* function of the R stats package.^23^ Furthermore, we used the beta-regression model to estimate the contribution of the sample clinical features [e.g. condition, post-mortem interval (PMI), age] on the cell proportion variation. We modelled the cell type proportion using the *betareg* R package.^24^

### Sub-clustering, trajectory reconstruction, and differential gene expression in three glial cell types

We subset cell-type-specific UMI raw counts. To identify the glial subpopulations, we integrated cells from different samples following the Seurat3 reciprocal principal component analysis based protocol considering the top 1000 highly variable genes for astrocytes and oligodendrocytes and the top 500 highly variable genes for microglia. Then we used the unsupervised and network-based Louvain clustering approach based on the top 25 principal components of the integrated datasets. Marker genes were defined as described before. We reconstructed the cellular activation trajectories following the *monocle3* approach. Briefly, cells from different samples were integrated, and factor size normalized. The sample effect was removed using the Mutual Nearest Neighbor method.^25^ Then the highly variable genes defined before were embedded in the first 25 principal components used for dimensionality reduction and trajectory inference using the DDR algorithm implemented in the *learn_graph* function of the *monocle3* R package.^11^ Pseudotime ordering was done in a supervised manner by rooting the trajectory in the graph node that maximizes the distance to the known activated cell subpopulation. We identified cell type-specific perturbed genes in idiopathic Parkinson’s disease using the Quasi-Poisson generalized linear model implemented in the *fit_models* function of the *monocle3* R package.^11^ Idiopathic Parkinson’s disease differential expression coefficient with *q* < 0.05 were considered as differentially expressed genes. Highly variable genes associated with the cell trajectories were identified using the spatial correlation analysis Moran’s I approach implemented in the *graph_test* function of the *monocle3* R package.^11^ Functional enrichment analysis of the differentially perturbed genes was done using Enrichr.^26^

### *CADPS2* expression validation in dopaminergic neurons

Midbrain tissues on PEN slides were fixed in ice-cold 75% ethanol for 3 min, then in 99% ethanol for 1 min and then air-dried for 5–10 min prior to dissection.^27^ From the SN of each sample, 150 neuromelanin-positive neurons were captured with laser-microdissection using the PALM Microbeam (Zeiss) in 20 µl nuclease-free water with 0.2 U/µl RNase inhibitor (Roche). RNA was extracted with the NucleoSpin RNA XS purification kit (Macherey-Nagel) according to the manufacturer’s protocol. The reverse-transcription into cDNA was performed with SuperScript III Reverse Transcriptase (ThermoFisher).

The *CADPS2* expression was quantified by means of digital PCR (dPCR) using the QuantStudio™ 3D Digital PCR System (Applied Biosystems). Samples were prepared following the manufacturer’s instructions using SYBR™ Green (S5763, Life Technologies) and QuantStudio™ 3D digital Master Mix v2 (A26359, Life Technologies). Primer sequences for CADPS2 are: forward 3′-AAACTCTGTGCCCTGGATGG-5′ and reverse 3′-GACAACACGCCTTCCAACAC-5′. Primer sequences for Actin are: forward 3′-CGAGGACTTTGATTGCACATTGTT-5′ and reverse 3′-TGGGGTGGCTTTTAGGATGG-5′. Samples were loaded on a QuantStudio™ 3D digital PCR Chip v2 using the QuantStudio™ 3D Digital PCR Chip loader. The PCR was then performed on the ProFlex™ 2X Flat PCR System using the following parameters: 95°C for 5 min, 45 cycles of 95°C for 10 s, and 60°C for 10 s, 72°C for 10 s. The chips were read using the QuantStudio™ 3D Digital PCR instrument and the data were analysed using the QuantStudio™ 3D AnalysisSuite, version 3.1.6-PRC-build18.

### Multi-fluorescence immunolabelling of the tissue

Paraffin-embedded PFA-fixed midbrain sections were deparaffinized by incubation at 60°C for 30 min. This was followed by washing with Histoclear (2 × 5 min) and ethanol gradient series (100%, 100%, 95%, 70% vol/vol, 5 min each), and finally in distilled water for 10 min. Antigen retrieval was performed in 1 mM EDTA, pH = 8, in a pressure cooker for 40 min. Next, the slides were washed in distilled water and 1% TBST and blocked with 10% NGS in 1% TBST for 1 h. The sections were then incubated in the primary antibody (anti-tyrosine hydroxylase MAB318, 1:100, Millipore; anti-myelin PLP, 1:100, Abcam; anti-IBA1 019-19741, 1:500, FUJIFILM Wako; anti-GFAP ab7260, 1:100, Abcam), diluted to a working concentration in 1% NGS in 0.1% TBST, at 4°C overnight. This was followed by washing 3 × 5 min in 1% TBST. Then, the midbrain sections were incubated with a secondary antibody (goat anti-mouse IgG1 Alexa Fluor 647, A21240; goat anti-mouse IgG2a Alexa Fluor 546, A-21133; goat anti-rabbit IgG Alexa Fluor 488, A-27034), diluted to a working concentration of 1:100 in 1% NGS in 0.1% TBST, for 1 h. Sections were rewashed 3 × 5 min in 1% TBST and incubated in Sudan black solution for 2 min. This was followed by three washes in 1% TBST and mounting in ProLong Gold mounting medium.

### Automated image analysis

Immunofluorescence images of human post-mortem midbrain sections were acquired with Carl Zeiss Axio Observer Inverted Microscope Z1 with 20× objective and analysed in MATLAB (Version 2019B, Mathworks). Automated in-house developed image analysis algorithms segmented the fluorescent cell areas (neurons, astrocytes, microglia, oligodendrocyte) extracting features such as area and perimeter. The segmentation of dopaminergic neurons was computed by convolving the raw TH channel with a Gaussian filter. TH-positive cell areas were detected by setting a pixel threshold followed by *bwareaopen* to remove small connected components to generate a TH area mask. The neuromelanin mask was computed by identifying areas below the selected pixel threshold and subtracting the small connected components with *bwareaopen*. The segmentation of astrocytes and microglia was calculated by selecting a pixel threshold, followed by an *imfill* filter to generate the cell area masks for GFAP or IBA1, respectively. Further, the skeleton of the IBA1 mask was generated with a thinning function to identify the branching of the mask. Because of the massive oligodendrocyte population, we generated the mask by selecting a pixel threshold to identify all the PLP1 positive areas without segmentation. The mean area of each individual was calculated, and the groups were compared with an unpaired *t*-test. The results were visualized with ggplot2 in R 4.0.0.

### Genotyping of Parkinson’s disease cases using NeuroChip

DNA samples from all idiopathic Parkinson’s disease cases underwent genotyping at the Institute of Human Genetics at the Helmholtz Zentrum München using the Illumina (San Diego, CA) NeuroChip.^28^ Standard genotype data quality control (QC) steps were carried out.^29^ Single nucleotide polymorphism (SNP) imputation was carried out on our NeuroChip data using the Michigan Imputation Server^30^ to produce a final list of common (minor allele frequency ≥1%) variants for further analyses. Imputed SNP positions were based on Genome Reference Consortium Human 37/human genome version 19 (GRCh37/hg19). All cases were screened for disease-associated variants in known major Parkinson’s disease genes (*SNCA*, *LRRK2*, *DJ-1*, *PRKN*, *GBA*, *PINK1*, *ATP13A2*, *VPS35*, *MAPT*, *DCTN1*, *DNAJC6*, *SYNJ1*, *VPS13C* and *MAPT*) covered by the NeuroChip.

### Cell type association with genetic risk of Parkinson’s disease

Association analysis of cell type-specific expressed genes with genetic risk of Parkinson’s disease was performed using Multi-marker Analysis of GenoMic Annotation (MAGMA) v1.08, to identify idiopathic Parkinson’s disease-relevant cell types in the midbrain. MAGMA is a gene-set enrichment analysis method that tests the joint association of all risk SNPs in a gene with the phenotype while accounting for the linkage disequilibrium (LD) structure between SNPs.^31^ In our study, the SNPs and their *P*-values were taken from the summary statistics of the Parkinson’s disease genome-wide association study (GWAS) from Nalls *et al*.^32^ (excluding 23andMe). The publicly available European subset of 1000 Genomes Phase 3 was used as a reference panel to estimate LD between SNPs. MAGMA analysis consists of three steps. First, the annotation step, where SNPs were mapped to genes using the NCBI GRCh37 build (annotation release 105). Gene boundaries were defined as the transcribed region of each gene. An extended window of 35 kb upstream and 10 kb downstream of each gene was added to the gene boundaries.^33^ Second, the gene analysis step computes gene-wise *P*-values based on SNP GWAS *P*-values. The third step is the competitive gene-set analysis implemented as a linear regression model on a gene-set data matrix. The gene-sets used here are the differentially expressed genes in every cell type or the cell-type-specific expressed genes [filtered for false discovery rate (FDR)-corrected *P*-values < 0.05, percentage of cells of the cluster where the expression was detected >0.5, and logFC > 0.25]. MAGMA gene-set analysis provides association results for every gene-set and for every gene in the gene-sets. The association of a gene with a cell type is quantified as a *z*-score. *Z*-scores will be close to zero if a gene is not differentially expressed, while high positive *z*-scores indicate most differentially expressed genes.

### Data availability

Raw snRNA-seq data for the 11 samples presented in this study are available in the Gene Expression Omnibus (GEO) with accession number GSE157783. Imaging data are available upon request.

## Results

We sampled adult human post-mortem midbrain tissue from five idiopathic Parkinson’s disease cases, for which pathology reports described a severe neuronal loss in the SN without a family history of Parkinson’s disease (Supplementary Table 1). We confirmed the idiopathic nature by SNP-Chip profiling of 179 467 known variants associated with neurological diseases, including Parkinson’s disease,^34^ which did not reveal a genetic aetiology (Supplementary Table 2). We sampled six control midbrains to match the idiopathic Parkinson’s disease patient characteristics. The average age of idiopathic Parkinson’s disease patients and control individuals were ∼77 [standard error of the mean (SEM = 3)] and ∼81 (SEM = 4) years, respectively, and both groups had similar post-mortem intervals (idiopathic Parkinson’s disease ∼22 and controls ∼16 h) (Supplementary Table 1).

**Figure 1:**
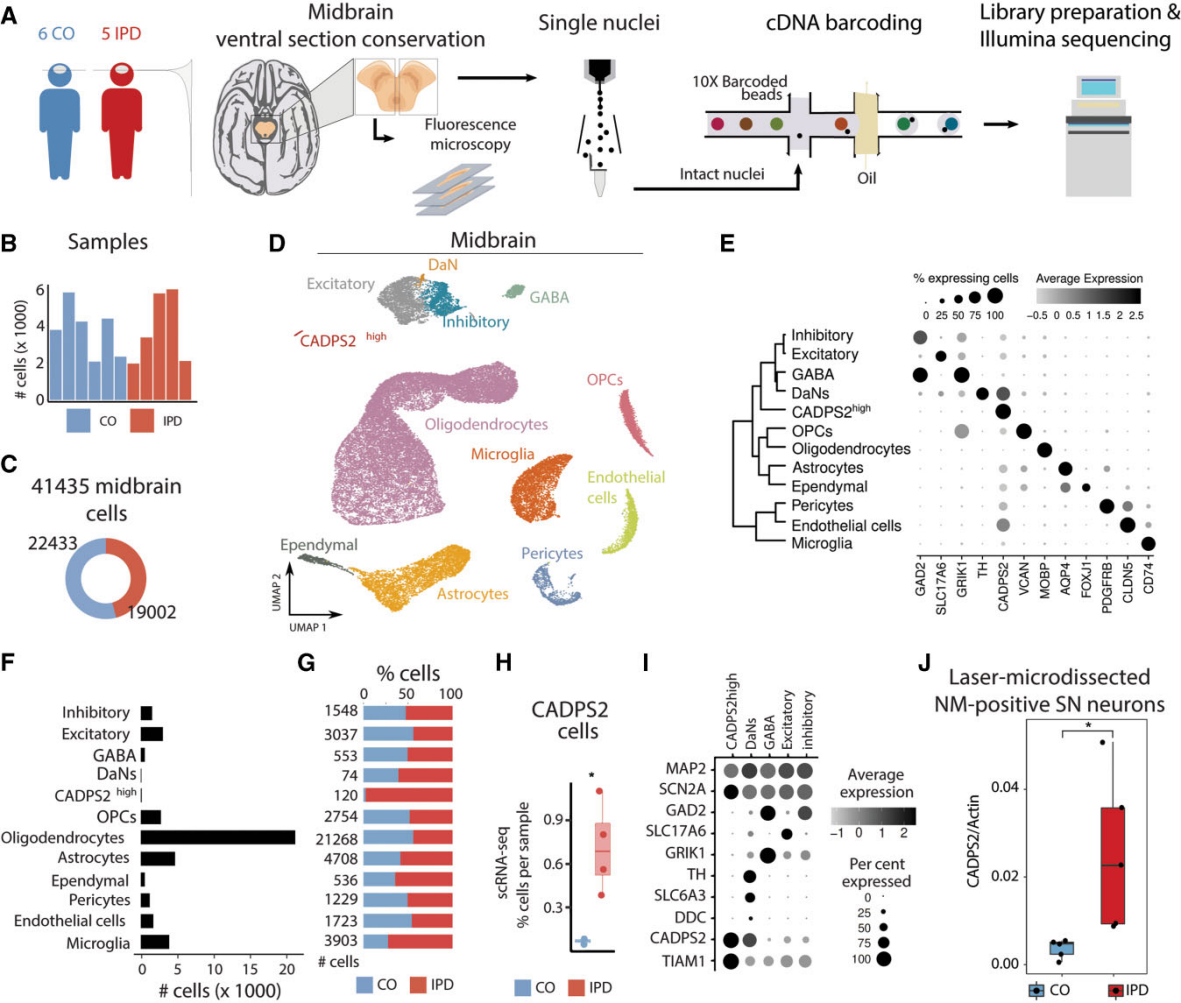
**Cell type composition of human midbrain.** (**A**) The experimental approach to midbrain tissue processing and nuclei extraction. Nuclei suspensions were processed with the 10× Genomics platform and sequenced with an Illumina sequencer. (**B**) Contribution of nuclei from idiopathic Parkinson’s disease (IPD) patients or controls to each cell type. (**C**) The number of high-quality nuclei per sample. Overall, the population consists of 19 002 nuclei from idiopathic Parkinson’s disease patients and 22 433 nuclei from controls. (**D**) UMAP embedding of the 41 435 human midbrain nuclei. Cells are coloured by cell type. (**E**) Cell type transcriptome similarity and representative marker genes. CADPS2^high^ cells cluster together with the neuronal cells. (**F**) The number of profiled nuclei per cell type. (**G**) The proportion of idiopathic Parkinson’s disease and control profiled cells per cell type. (**H**) CADPS2^high^ cell proportion per sample, (*t*-test *P* = 0.02). (**I**) CADPS2^high^ cells are neurons. They express *MAP2*, *SCN2A*, and *TIAM1*, but have low levels of *TH*. (**J**) Digital PCR reveals significantly higher expression of *CADPS2* in neuromelanin-positive nigral neurons (*n* = 150 per person) dissected from idiopathic Parkinson’s disease midbrain sections compared to those isolated from control tissue (*t*-test *P* = 0.027).

We sequenced single nuclei from frozen ventral sections of human post-mortem midbrains (Fig. 1A) and obtained ∼2000–6000 high-quality nuclei per sample with an average of ∼7600 transcripts and ∼2700 genes per nucleus after filtering out poorly sequenced nuclei and potential doublets (Fig. 1B). This dataset comprised 22 433 and 19 002 single nuclei from control individuals and patients with idiopathic Parkinson’s disease, respectively (Fig. 1C).

We embedded the 41 435 nuclei transcriptomes into two dimensions using the UMAP algorithm. We found that the overall cluster structure was mostly driven by cell-type identity and inter-sample variability (Supplementary Fig. 1A and B). Of note, patient and control cells gathered together within the major cell clusters (Supplementary Fig. 1B). To account for this inter-individual variation during the cell-type identification, we followed the Seurat3 sample integration protocol (see ‘Materials and methods’ section) (Fig. 1D). Using this corrected principal component analysis embedding and the unsupervised, network-based Louvain clustering approach, we found that the human midbrain comprised 12 major cell types (Fig. 1D and E and Supplementary Fig. 1C).

The studied human midbrain tissue was composed of glial, neuronal, and vascular cells (Fig. 1D and E). We annotated most cell clusters by manually comparing well-known marker genes in the literature and the identified marker genes of each unsupervised cell cluster (Supplementary Fig. 1C–I and Supplementary Table 3). Oligodendrocytes, the most abundant cell type in the midbrain (Fig. 1F), were characterized by the expression of *MOBP*.^35^ Oligodendrocyte precursor cells (OPCs) highly express *VCAN.*^36^ Expression of *AQP4* was characteristic for astrocytes^37^ and *FOXJ1* for ependymal cells (Fig. 1E–G and Supplementary Fig. 1C–I).^38^ Also, immune and vascular cells displayed a highly specific expression of well-known marker genes; *CD74* in microglia,^39^*CLDN5* in endothelial cells,^40,41^ and *GFRB* in pericytes^42^ (Fig. 1E–G and Supplementary Fig. 1C). Regarding neuronal cells, we identified four cell types: excitatory (*SLC17A6*),^43^ inhibitory (*GAD2*),^43^ GABAergic (*GAD2*/*GRIK1*)^44,45^ and, dopaminergic neurons (*TH*) (Fig. 1E–G and Supplementary Fig. 1C–I).^46^

A closer look at the number of profiled nuclei indicated that DaNs only comprised 0.18% of the total cell count limiting the comparison between the idiopathic Parkinson’s disease and control DaNs (Fig. 1F). Therefore, we performed quantitative immunofluorescence imaging analysis of TH- or neuromelanin-positive cells in idiopathic Parkinson’s disease and control tissues and confirmed a significant reduction in TH- or neuromelanin-positive nigral DaNs in the idiopathic Parkinson’s disease midbrains compared to controls (Supplementary Fig. 2A–D).

Interestingly, we also found a neuronal cluster of 120 cells, which we could not annotate initially based on known marker genes, that was characterized by high expression of *CADPS2* (CADPS2^high^ cells) (Fig. 1E–F and H–I and Supplementary Fig. 1C and I). These cells almost exclusively originated from idiopathic Parkinson’s disease patients (idiopathic Parkinson’s disease, 98.4%; control, 1.6%) (Fig. 1G). Quantitative assessment of the cell annotation assignment validated our cell-type annotation (Supplementary Fig. 1D). With regard to neuronal markers, these cells show a similar profile to DaNs, except for low *TH* abundance (Fig. 1I). Moreover, CADPS2^high^ cells express even higher levels of *TIAM1* than DaNs (Fig. 1I). TIAM1 has been identified as a regulator of the Wnt/Dvl/Rac1 pathway, which controls midbrain DaN differentiation.^47,48^ Thus, we hypothesized that CADPS2^high^ cells might constitute degenerating DaNs.

In order to test this hypothesis, we applied laser-capture microdissection (LCM) to frozen midbrain sections from additional idiopathic Parkinson’s disease patients and aged control subjects. Our ‘validation cohort’ included C1 and IPD4 from the original ‘snRNA-seq cohort’ as well as sections from four previously unstudied cases and four new controls (Supplementary Table 1). From each individual, we isolated 150 neuromelanin-containing neurons that were subjected to *CADPS2* gene expression analysis by means of digital PCR, whereby *Beta-actin* served as a house-keeping gene. This experiment indicated significantly higher *CADPS2:Beta-actin* ratios in idiopathic Parkinson’s disease compared to control DaNs with neuromelanin deposits (Fig. 1J), suggesting that CADPS2^high^ cells are indeed of dopaminergic origin.

**Figure 2:**
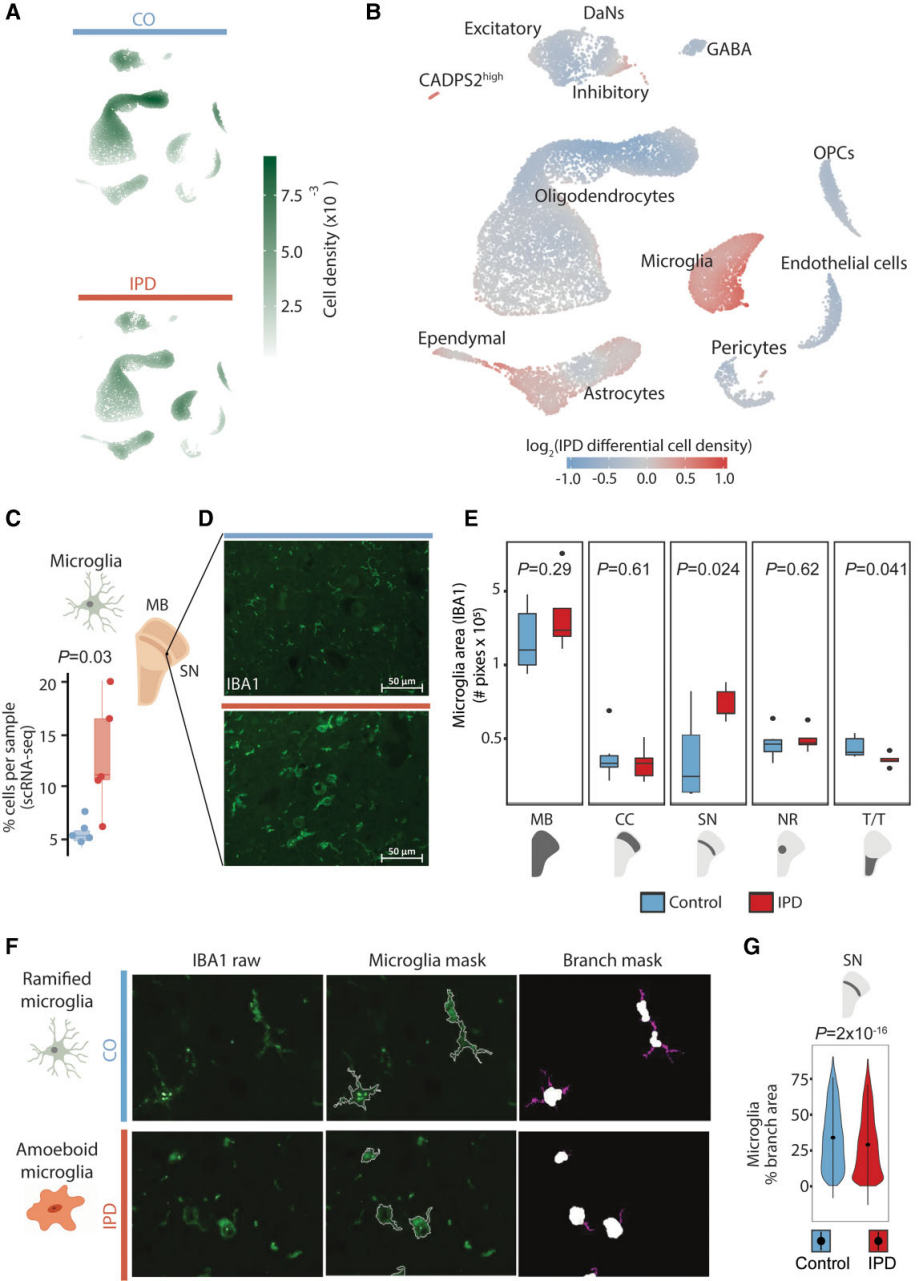
**Idiopathic Parkinson’s disease (IPD) midbrain is characterized by an increase in microglia.** Differential cell type composition in idiopathic Parkinson’s disease patients compared to age-matched control subjects. (**A**) Two-dimensional cell density in the first UMAP embeddings of the human midbrain for idiopathic Parkinson’s disease patients and control subjects independently. (**B**) Differential 2D cell density in idiopathic Parkinson’s disease midbrain. Idiopathic Parkinson’s disease midbrain has a larger population of microglia and astrocytes than control midbrain tissue. (**C**) Microglia cell proportion per sample. Idiopathic Parkinson’s disease patients display a higher proportion of microglia cells (*t*-test *P* = 0.03). (**D**) IBA1 immunofluorescence in idiopathic Parkinson’s disease and control ventral midbrain sections. (**E**) IBA1-positive areas in the entire midbrain and individual regions of 11 individuals. The Parkinson’s disease-associated increase of microglia is the most significant in the SN (*t*-test *P* = 0.024). (**F**) Microglia morphology analysis. (**G**) An idiopathic Parkinson’s disease-associated reduction of microglia branching indicates less ramified microglia in the SN (*t*-test *P* = 2 × 10^−16^), which implies increased cell reactivity. MB = midbrain; PD = Parkinson’s disease; NR = nucleus ruber; TT = tectum/tegmentum; CC = crus cerebri. IPD: red bar; control: blue bar; scale bar = 50 μm.

Next, we aimed to reveal cell-type composition changes of the midbrain associated with idiopathic Parkinson’s disease and followed three approaches. We compared idiopathic Parkinson’s disease and control cell density distributions in the 2D UMAP representation (Fig. 2A and B) and analysed the idiopathic Parkinson’s disease and control distributions of the cell type proportions per sample. Altogether, these results revealed an increase in the fraction of microglia and astrocytes and a decreased fraction of oligodendrocytes in idiopathic Parkinson’s disease midbrains compared to controls (Fig. 2C, Supplementary Fig. 3A and D and Supplementary Table 6). To validate these results with an independent approach, we examined paraformaldehyde-fixed paraffin-embedded sections from the right hemi-midbrain of the same 11 individuals by performing multi-labelling immunofluorescence analysis.^49^ First, we confirmed the increased fraction of microglia in idiopathic Parkinson’s disease midbrains by labelling it with an antibody against the marker protein IBA1 (Fig. 2D). Automated image analysis demonstrated an increase in IBA1-positive areas in idiopathic Parkinson’s disease midbrain tissue compared to control samples (Fig. 2E and Supplementary Table 7). This microglia increase was the most significant in the SN compared to other midbrain regions (Fig. 2E and Supplementary Table 7). Further image analysis of the microglia cellular shape in the SN of age- and sex-matched idiopathic Parkinson’s disease and control cases (Fig. 2F) revealed an idiopathic Parkinson’s disease-related decrease in microglial branching, indicating cellular activation (Fig. 2G and Supplementary Table 7).^50^ Second, we validated the increased fraction of astrocytes and a decreased fraction of oligodendrocytes in idiopathic Parkinson’s disease midbrains. We labelled astrocytes and oligodendrocytes with antibodies against their marker proteins GFAP and PLP1, respectively (Supplementary Fig. 3B and E). We observed a trend towards a higher abundance of GFAP-positive areas throughout all the regions in idiopathic Parkinson’s disease midbrain tissue compared to control subjects (Supplementary Fig. 3C, Supplementary Table 7). Moreover, we detected a reduction of PLP1-positive areas in the idiopathic Parkinson’s disease midbrain sections compared to controls with the highest significance in the SN (Supplementary Fig. 3F). In contrast, the other midbrain cell types, OPCs, pericytes, ependymal, excitatory, inhibitory, and GABAergic cells, did not display significant deviations associated with idiopathic Parkinson’s disease (Supplementary Fig. 4 and Supplementary Table 6).

We also investigated how other clinical characteristics, in addition to the disease status (condition), affect the midbrain cellular composition. For this, we modelled the percentage of each cell type per sample as a function of age, post-mortem interval, and condition. We used beta-regression modelling to estimate the coefficients of these clinical features (Supplementary Fig. 5). Condition (idiopathic Parkinson’s disease) appeared to be the sample characteristics with the highest impact on the midbrain cellular composition. For instance, the most significant coefficients were the loss of DaNs and the gain of CADPS2^high^ cells associated with idiopathic Parkinson’s disease (Supplementary Fig. 5).

**Figure 3:**
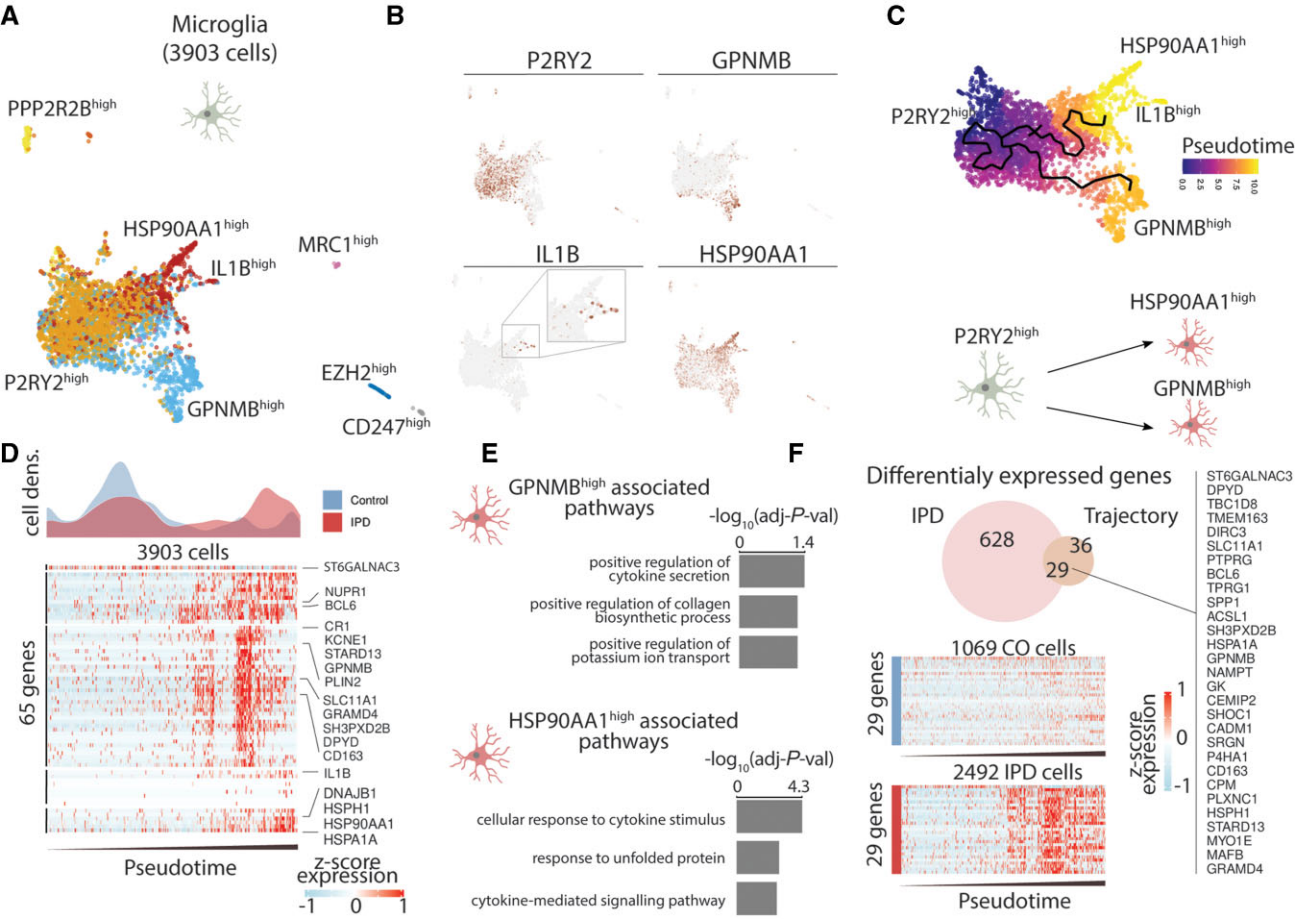
**Trajectory reconstruction reveals microglia differential activation in idiopathic Parkinson’s disease (IPD).** (**A**) Microglia subpopulations labelled with a representative marker gene. (**B**) Expression of *P2RY12*, *GPNMB*, *IL1B* and *HSP90AA1* along the ∼4000 microglia cells. These genes are characteristic of the major three microglia subpopulations. (**C**). Trajectory reconstruction and pseudotime representation based on the *P2RY1*^high^, *GPNMB*^high^, and *HSP90AA1B*^high^ subpopulations. This reveals a two-branches activation trajectory. (**D**) Differential cell-density distribution along pseudotime for idiopathic Parkinson’s disease and control samples. Also, the expression of 65 genes, whose expression is associated with the microglia activation trajectory. *Z*-score normalized expression is presented for each gene over ∼4000 microglia cells organized by their pseudotime. (**E**) Gene ontology (GO) molecular function enrichment of the genes associated with the *GPNMB* and *HSP90AA1* activation trajectories. (**F**) Twenty-nine idiopathic Parkinson’s disease differentially expressed genes intersect with the differentially expressed genes along the microglia activation trajectory.

To reveal the transcriptional programmes and pathways associated with the increased fraction of microglia and astrocytes in idiopathic Parkinson’s disease, we subclustered these cell types to identify glial subpopulations and reconstruct their activation trajectories. We identified seven microglia subpopulations characterized by the expression of a few marker genes (Fig. 3A). The three biggest subpopulations are defined by the high expression of *P2RY12*, *GPNMB*, and *HSP90AA1* (Fig. 3B). Given that these three subpopulations conform to a continuum in the UMAP projection and both *GPNMB* and *HSP90AA1* are microglia activation markers, we estimated a cell trajectory structure comprising these major subpopulations (Fig. 3C). We then organized cells along this trajectory (pseudotime), starting from the trajectory node that maximizes the distance to the *GPNMB* and *HSP70AA1* trajectory branch tips (Fig. 3C). This microglia activation trajectory spans from *P2RY12*^high^ cells towards two activation branches, one containing *GPNMB*^high^ cells and another with cells highly expressing *HSP90AA1* or *IL1B* (Fig. 3C). Idiopathic Parkinson’s disease cells differentially distribute along the microglia activation trajectory being enriched towards the activated state compared (Fig. 3D and Supplementary Fig. 6). While P2RY12 is highly abundant in the resting microglia,^51^ GPNMB,^52^ HSP90,^53^ and IL-1β^54^ are involved in the inflammatory response and have previously been linked to neurodegeneration^53,55,56^ supporting the notion that the idiopathic Parkinson’s disease-specific upregulation of *GPNMB* and *HSP90AA1* are markers of microglial activation. To further characterize the molecular phenotype of these two activated microglia states, we identified genes whose expression was associated with the activation trajectory and functionally enriched them to gene-ontology molecular functions (Fig. 3D and E). This analysis revealed that these subpopulations are enriched in cytokine secretion and the stress response to unfolded protein pathways (Fig. 3E). Next, we identified the genes whose expression was differentially upregulated in idiopathic Parkinson’s disease microglia across the activation trajectory. We intersected the upregulated genes in idiopathic Parkinson’s disease and the activation-trajectory associated genes in microglia and identified 29 genes linked with the differential activation of the midbrain microglia in idiopathic Parkinson’s disease (Fig. 3F and Supplementary Table 8), several of which have previously been associated with idiopathic Parkinson’s disease.^57–59^

**Figure 4:**
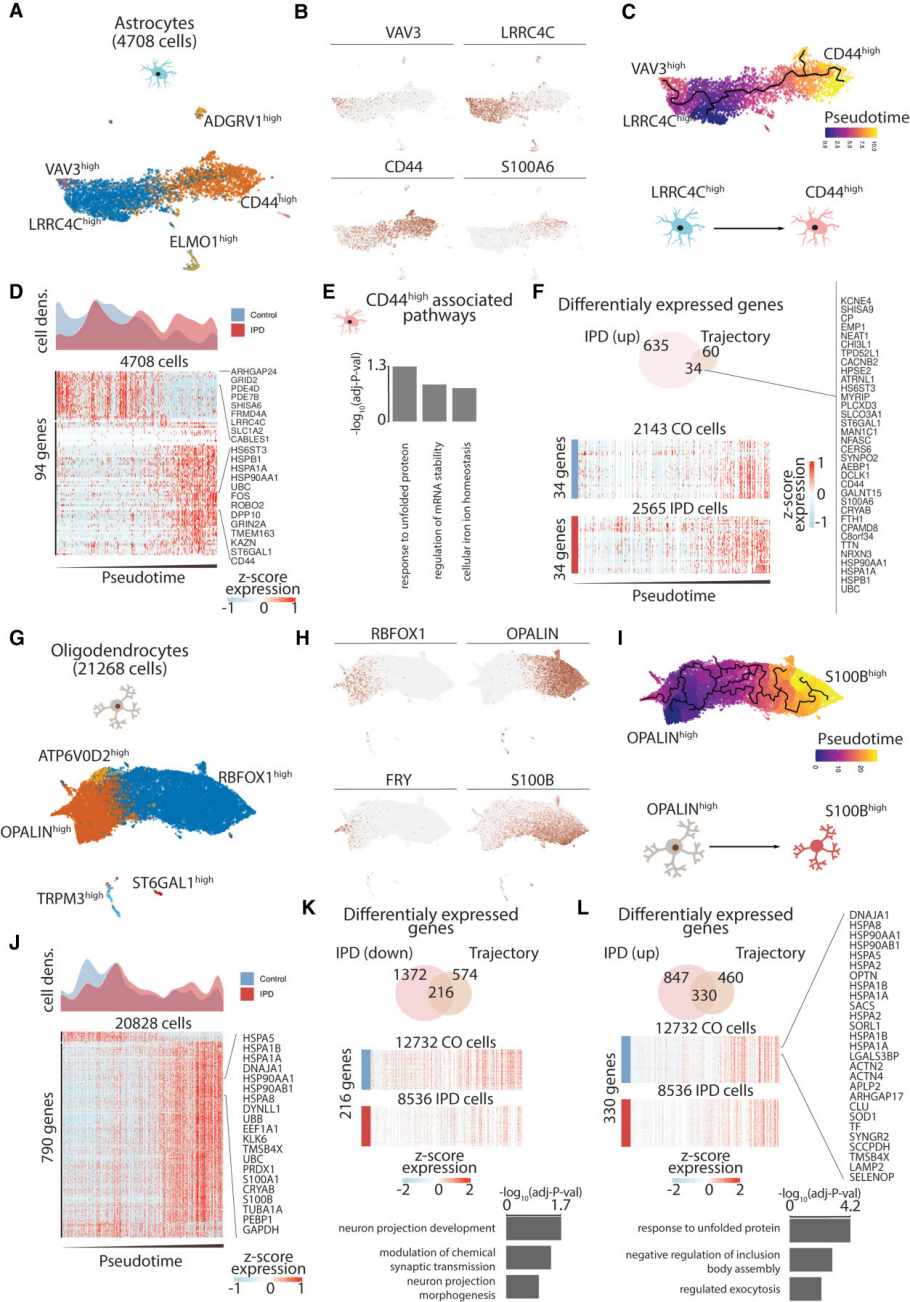
**Trajectory reconstruction reveals astrocyte differential activation, loss of myelinating oligodendrocytes, and differential activation in idiopathic Parkinson’s disease (IPD).** (**A**) Astroglial subpopulations are named based on characteristic marker genes. (**B**) *VAV3*, *LRRC4C*, *CD44*, and *S100A6* expression across the ∼4700 astrocytes. (**C**) Inferred cell trajectory and pseudotime for the major astrocyte subpopulations, *VAV3*^high^, *LRRC4C*^high^ and *CD44*^high^ cells. (**D**) Idiopathic Parkinson’s disease and control differential cell-density distribution over pseudotime and the expression of the 94 genes highly associated with the astrogliosis trajectory in the ∼4700 astrocytes organized by pseudotime. (**E**) GO molecular function pathway enrichment of the upregulated genes in the *CD44*^high^ activated branch. (**F**) The 34 intersected genes between the upregulated genes in idiopathic Parkinson’s disease and across the astrocyte activation trajectory. (**G**) Oligodendrocyte subpopulations are named based on representative marker genes. (**H**) Expression of *OPALIN*, *RBFOX1*, *FRY* and *S100B* in the ∼21 000 oligodendrocytes. (**I**) Inferred cell trajectory and pseudotime ordering of the major oligodendrocytes subpopulations, *OPALIN*^high^, *ATP6V0D2*^high^, and *S100B*^high^ cells. (**J**) Idiopathic Parkinson’s disease and control differential cell density over pseudotime. Expression levels of 790 highly variable genes across the oligodendrocyte trajectory. Expression is presented for ∼21 000 oligodendrocytes organized by their pseudotime. (**K** and **L**) The intersection of idiopathic Parkinson’s disease differentially expressed and trajectory-associated genes. Also, the GO molecular enrichment of the intersected genes is presented. (**K**) Two hundred and sixteen idiopathic Parkinson’s disease downregulated genes across the trajectory are associated with pathways important for neuron projection and synaptic transmission. (**L**) Three hundred and thirty genes are idiopathic Parkinson’s disease upregulated along the oligodendrocyte trajectory. These genes are mainly associated with the unfolded protein response.

We also characterized the astrocyte and oligodendrocyte subpopulations, reconstructed their activation trajectories, and identified gene signatures associated with idiopathic Parkinson’s disease differential activation (Fig. 4). First, we identified five astrocyte subpopulations characterized by high expression of *VAV3*, *LRRC4C*, *ELMO1*, *ADGRV1* and *CD44* (Fig. 4A and B). We recovered the astrocyte activation trajectory based on the main cell types comprising *VAV3*^high^, *LRRC4C*^high^, and *CD44*/*S100A6*^high^ subpopulations (Fig. 4C). Given that *CD44* expression implicates reactive astrogliosis,^60^ we ordered cells on the activation trajectory by setting the root in the trajectory graph-node that maximizes the distance from the *CD44*^high^ branch end. These results implied an astrocyte activation transition from *LRRC4C*^high^ to *CD44*^high^ subpopulations (Fig. 4C). Indeed, idiopathic Parkinson’s disease astrocytes were highly enriched at the end of the astrogliosis trajectory compared to control astrocytes (Fig. 4D and Supplementary Fig. 6). We further characterized the molecular phenotype of the *CD44*^high^ astrocyte activated state by enriching GO molecular functions to the highly upregulated genes across the astrocytes activation trajectory (Fig. 4D and E). The *CD44*^high^ subpopulation was related to the unfolded protein response (UPR) pathway, which has recently been linked to a specific astrocyte reactivity state that is detrimental to the survival of neurons (Fig. 4E).^61^ Next, we calculated idiopathic Parkinson’s disease-differentially upregulated genes, which were also highly expressed towards the end of the astrogliosis trajectory (Fig. 4F), and identified 34 genes associated with idiopathic Parkinson’s disease differential astrogliosis (Fig. 4F and Supplementary Table 8). These genes include several heat-shock proteins that have previously been shown to co-localize with α-synuclein deposits in the human brain.^62^ Similarly, we investigated the oligodendrocyte diversity and reconstructed its differentiation trajectory (Fig. 4G–L). We identified five subpopulations characterized by the expression of *ATP6V02*, *OPALIN*, *TRPM3*, *ST6GAL1*, and *RBFOX1* (Fig. 4G and H). The inferred trajectory based on subpopulations recovered differentiation trajectory spanning from *FRY*/*OPALIN*^high^ cells towards *RBFOX1*/*S100B*^high^ cells (Fig. 4H and I). *OPALIN* (also denominated as *Tmem*) is a marker of myelinating oligodendrocytes,^63^ while *S100B* has been associated with glial stress response in Parkinson’s disease post-mortem midbrain.^64^ When comparing idiopathic Parkinson’s disease oligodendrocyte density across this trajectory, we found a reduced fraction of myelinating *OPALIN*^high^ cells compared to controls (Fig. 4J and Supplementary Fig. 6). An overlay of the idiopathic Parkinson’s disease-differentially expressed genes and of such genes defining the oligodendrocyte trajectory identified 216 and 330 downregulated and upregulated genes across the trajectory. Downregulated genes are associated with neuronal maintaining pathways, while upregulated genes are related to the response to unfolded protein pathways (Fig. 4K and L and Supplementary Table 8). We also investigated the idiopathic Parkinson’s disease differential expression across all cell types in the human midbrain and evidenced that the unfolded protein pathways are also upregulated in the OPCs, and the vascular cells (Supplementary Table 8).

**Figure 5:**
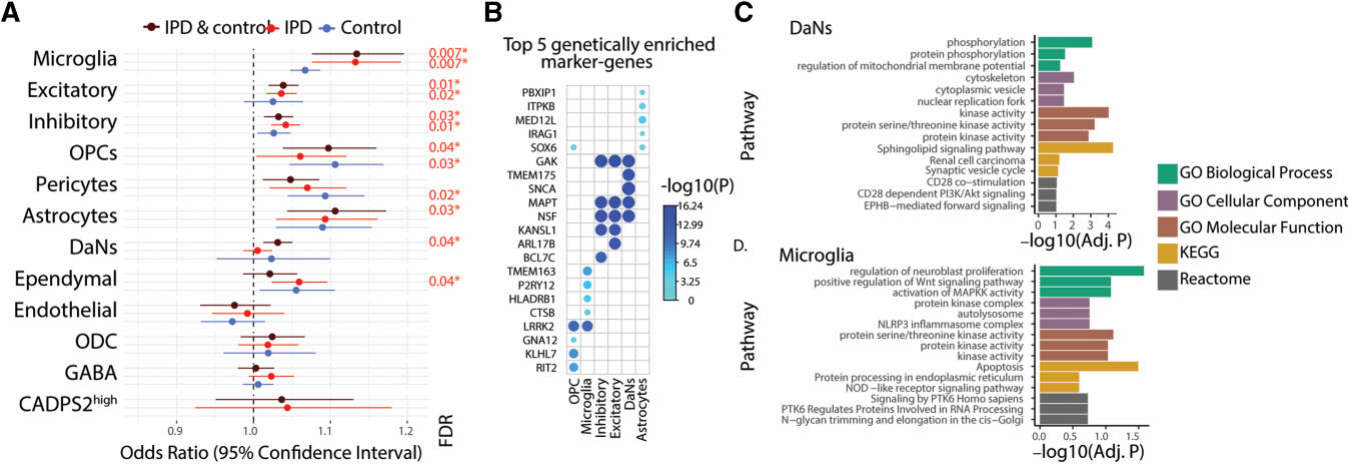
**Idiopathic Parkinson’s disease (IPD)-associated genetic variants enriched in microglia and neuron-specific genes.** (**A**) Forest plots of the odds ratio (OR) and 95% confidence intervals for the association between the Parkinson’s disease-associated variants and the marker genes of the midbrain cell types from idiopathic Parkinson’s disease patients, control subjects and both conditions. This approach describes the enrichment of Parkinson’s disease risk variants, taken from the latest Parkinson’s disease GWAS, in genes with cell-type-specific patterns in order to identify idiopathic Parkinson’s disease relevant cell types in the midbrain. Only significant association *P*-values were shown (*P < 0.05). (**B**) Top five enriched genes in six midbrain cell types. The association of a gene with a cell type is quantified and the most responsible genes for the genetic variant enrichment observed in (**A**) were shown. The *P*-values of genes association are colour coded from light to dark blue and the size of circles is inversely proportional to *P*-values. (**C** and **D**) Gene Ontology terms (GO) and molecular pathways (KEGG, Reactome) associated respectively with the DaNs and microglia marker genes responsible for the Parkinson’s disease variant enrichment.

To gain cellular mechanistic insights into how the Parkinson’s disease-associated genetic variants could affect the midbrain physiology, we evaluated the enrichment of midbrain cell-type marker genes with the Parkinson’s disease-associated genetic variants. We found that Parkinson’s disease risk variants are significantly associated with microglia, neurons, astrocytes, and OPCs (Fig. 5A and Supplementary Table 4). Having access to both control and idiopathic Parkinson’s disease tissue, we tested whether these associations depend on the disease context. After analysing each condition separately, we found that the Parkinson’s disease risk variants associate differently with patients and controls (Fig. 5A). Considering idiopathic Parkinson’s disease samples alone, microglia and neurons remain significantly associated with Parkinson’s disease risk variants (Fig. 5A and Supplementary Table 4). By contrast, in control subjects, disease variants are associated with pericytes and OPCs (Fig. 5A and Supplementary Table 4). These results show that the link between Parkinson’s disease genetic risk and cell type is highly influenced by the disease status. When analysing the samples separately (Fig. 5A), the association of DaNs to risk variants is weaker, presumably due to the low number of DaNs. Therefore, we utilized the entire dataset (controls and idiopathic Parkinson’s disease cases) for further analyses (Fig. 5B–D).

We prioritized the cell-type-specific and Parkinson’s disease risk-associated genes based on their enrichment contribution for each cell type (Fig. 5B). We found that *LRRK2* showed the highest association with microglia and OPCs, and *SNCA* was the most prominent Parkinson’s disease-associated gene in DaNs (Fig. 5B and Supplementary Table 5). These findings are in line with previous reports of Parkinson’s disease-associated mutations in α-synuclein promoting Lewy body formation in DaNs^65^ and with studies suggesting a role for *LRRK2* mutations in the activation of microglia in Parkinson’s disease.^66^ Lastly, we investigated which pathways are associated with the Parkinson’s disease variant enrichment in DaNs and microglial differentially expressed genes (Fig. 5C and D). Among the key hits from GO, KEGG, and Reactome, we identified terms such as ‘phosphorylation’ and ‘kinase activity’ in DaNs and ‘NLRP3 inflammasome complex’ in microglia (Fig. 5D). In particular, the latter finding further supports a role for inflammatory signalling in Parkinson’s disease.

## Discussion

This study provides the first single-cell atlas of the human midbrain from idiopathic Parkinson’s disease patients and age-matched control subjects to the best of our knowledge. Rather than exclusively focusing on nigral DaNs, the most studied cell type in Parkinson’s disease, we aimed to characterize cell- and disease-specific molecular signatures associated with idiopathic Parkinson’s disease in the entire midbrain. In addition, we associated Parkinson’s disease risk variants to specific midbrain cell types in idiopathic Parkinson’s disease patients and control subjects.

Our key observations include an increment in the astrocytes and microglia midbrain fractions, which coincided with a reduction of oligodendrocyte fraction in the idiopathic Parkinson’s disease midbrain. Immunofluorescence analysis and pseudotime trajectory reconstructions revealed glial activation in idiopathic Parkinson’s disease—a finding that was further supported by Parkinson’s disease variant enrichment in the idiopathic Parkinson’s disease microglia. Finally, we discovered a small CADPS2-positive neuronal cell cluster in idiopathic Parkinson’s disease midbrain tissue, which warrants further investigations in a larger sample set.

When assessing DaNs in our snRNA-seq data, we did not observe a significant loss in idiopathic Parkinson’s disease tissue. The low abundance of DaNs likely hampered this compared to other cell types in the midbrain. However, the automated image analysis of immunofluorescence-labelled idiopathic Parkinson’s disease and control midbrain sections confirmed a significant loss of TH-positive DaNs and neuromelanin aggregates. This result was in line with the neuropathological reports, which described severe DaN degeneration in all idiopathic Parkinson’s disease patient samples (*cf*. Supplementary Table 1). Thus, technical limitations may have caused the under-representation of DaNs in the transcriptomic data. First, we used 15-µm thick midbrain slices, which are in the size range of the rather large DaN nuclei (10–20 µM). Hence, a considerable proportion of nuclei may not have remained intact during the sectioning process—a prerequisite for high-quality snRNA-seq results. Second, rather than sampling only SN cells, we extracted nuclei from the entire midbrain. This may have led to an under-representation of nigral neurons in our dataset. Despite these constraints, when combining the latest GWAS^32^ with our snRNA-seq results from idiopathic Parkinson’s disease and control midbrain sections, we observed an enrichment of Parkinson’s disease variants in DaNs. Pathway analyses of differentially expressed DaN marker genes with Parkinson’s disease variant enrichment identified processes such as ‘mitochondrial function’ and ‘kinase activity’ that have previously been associated with Parkinson’s disease.

In addition, we identified a disease-specific cell type, consisting of only 120 cells, characterized by its transcriptional similarity to midbrain DaNs but with low *TH* levels and high *CADPS2* expression. CADPS2 has previously been linked to catecholamine uptake and genetic Parkinson’s disease.^67–69^ In addition, elevated levels of *TIAM1*, which is involved in Wnt/Dvl/Rac1 signalling,^47,48^ made us wonder whether CADPS2^high^ cells constitute degenerating DaNs that have lost their dopaminergic identity. Aberrant dopamine function in metabolically impaired but viable neurons in the SN has previously been observed in Parkinson’s disease post-mortem tissue.^70^ Corroborating our hypothesis, *CADPS2* quantification in neuromelanin-positive DaNs isolated from midbrain tissue of two scRNA-sequenced samples (C1 and IPD4) and four additional idiopathic Parkinson’s disease patients and four new control subjects revealed higher levels of *CADPS2* in the former cells. However, further mechanistic studies beyond the scope of the manuscript will be needed to uncover the physiological cause and consequence of *CADPS2* upregulation in DaNs.

In our dataset, glia made up ∼80% of all sequenced cells, enabling an in-depth analysis of their contribution to the pathogenesis of idiopathic Parkinson’s disease. We identified a disease-specific upregulation of microglia, which mediates the innate immune defence in the brain. During microgliosis, microglia amplify, undergo morphological changes, and secrete cytokines, which can further engage surrounding microglia and astrocytes.^7,71^ Suggestive of an activated state, we detected fewer ramified microglia in idiopathic Parkinson’s disease post-mortem SN tissue using a quantitative immunofluorescence approach. Moreover, we identified a significant Parkinson’s disease risk variant enrichment in microglia, showing the strongest association with the Parkinson’s disease gene *LRRK2*. The kinase LRRK2 is most abundant in immune cells and may contribute to inflammasome formation via the phosphorylation of Rab GTPases.^72^ In line with this finding, Parkinson’s disease risk variant enrichment analysis in microglial differentially expressed genes highlighted the kinase activity and NLRP3 inflammasome pathways. By inferring the activation trajectories of the microglial subpopulations, we observed an increase in cells from resting into an activated state. Interestingly, our finding of *GPNMB* upregulation in activated microglia is supported by recent results in Alzheimer’s disease brains. Reactive patient microglia, which presented an amoeboid shape, also showed higher GPNMB protein levels in this immunohistochemistry study.^55^ Moreover, pathway analyses in the activated cell populations identified cytokine signalling and, likely upstream of this, induction of the UPR pathway in the microglia. We also found chaperones and heat-shock proteins to be overexpressed along the disease trajectory, which, when they are released from the cell, can act as damage-associated molecular patterns (DAMPs) that trigger an immune reaction.^73^

Astrocytes can equally act as immune effector cells in the brain by releasing proinflammatory cytokines.^74^ When modelling astroglial activation trajectories, we detected reactive astrogliosis specifically in idiopathic Parkinson’s disease patient cells.^60^ As for microglia, pathway analysis along the trajectory identified the UPR pathway, which has recently been described to influence the astrocytic secretome.^61^ Neurotrophic factors released from reactive astrocytes were shown to accelerate neuronal demise^61^—a disease mechanism that has not gained much attention in Parkinson’s disease research so far. Besides neurons, reactive astrocytes can also affect oligodendrocyte function and survival.^7^

Accordingly, our snRNA-seq data also showed a trend towards decreased oligodendrocyte numbers in idiopathic Parkinson’s disease midbrain tissue. Immunofluorescence analyses suggest that this reduction is the most profound in the SN. In the white matter, oligodendrocytes generate myelin sheets, which provide insulation of axons and ensure saltatory conduction.^75^ However, since Parkinson’s disease has long been considered a ‘grey matter’ disease, oligodendrocytes only recently gained attention in the field. A single-cell study^76^ in nigral tissue from controls showed that common genetic Parkinson’s disease risk variants are associated with oligodendrocyte-specific expression. Another study on the entire mouse nervous system also reported an association with oligodendrocytes.^77^ By contrast, we did not observe an enrichment of Parkinson’s disease risk variants in oligodendrocytes from control or idiopathic Parkinson’s disease tissue. This may be explained by the fact that our data are based on nuclei from the entire midbrain, possibly masking nigra-specific genetic effects. However, a closer look into trajectory inference analysis in oligodendrocytes revealed a transition from high *OPALIN* to high *S100B* expression subpopulations. S100B was shown to control the maturation process of oligodendrocytes^78^ and has previously been linked to neurodegeneration.^79^*S100B* overexpression in response to cytokine injections mediates dystrophic neurite formation in an Alzheimer rat model.^79^ Accordingly, the oligodendrocyte-specific upregulation of *S100B* observed in the idiopathic Parkinson’s disease midbrains may be the result of enhanced cytokine release from microglia and astrocytes. These results further implicate glial cells in the propagation of neuroinflammatory and neurodegenerative processes in idiopathic Parkinson’s disease.

In summary, our study reinforces the relevance of neuroinflammation in idiopathic Parkinson’s disease. Applying snRNA-seq for the first time to post-mortem midbrain tissue from patients and matched control subjects, we identified a disease-specific upregulation of microglia and astrocytes as well as a loss of oligodendrocytes. In addition, we discovered a small neuronal cell population that was almost exclusively identified in idiopathic Parkinson’s disease midbrain tissue, likely representing degenerating DaNs. Disease trajectory analyses in the glial cell populations identified stress in response to misfolded proteins as the major trigger of inflammatory signalling in idiopathic Parkinson’s disease, extending from microglia via astrocytes to oligodendrocytes. Further strengthening this finding, Parkinson’s disease risk variants were specifically enriched in microglia from idiopathic Parkinson’s disease patients.

Our study also has several limitations. Due to the precious nature of post-mortem brain tissue, our results are based on snRNA-seq of sections from 11 individuals and dPCR analysis of laser-capture microdissected DaNs from eight additional individuals. Therefore, single-cell RNA analyses in independent cohorts will be needed to validate the key findings from our study. Moreover, additional *in vitro* and *in vivo* experiments are necessary to explore the role of CADPS2 in the pathogenesis of Parkinson’s disease and to elucidate how the observed glial interplay perpetuates or induces DaN demise.

Despite these challenges, our unique human single-cell dataset provides the basis for new research approaches investigating the role of the different midbrain cell types in idiopathic Parkinson’s disease and for translational programmes that aim to develop immunomodulatory Parkinson’s disease therapies.

## Supplementary Material

awab446_Supplementary_Data

## Abbreviations

DaNs: dopaminergic neurons
OPCs: oligodendrocyte precursor cells
SN: substantia nigra;
SNP: single nucleotide polymorphism
snRNA-seq: single-nucleus RNA sequencing
UMAP: uniform manifold approximation and projection
