# Supplementary material for: Single-cell sequencing of human midbrain reveals glial activation and a Parkinson-specific neuronal state
Source: Brain. 2021 Dec 17;145(3):964–78. doi: 10.1093/brain/awab446 (PMC9050543; doi:10.1093/brain/awab446)
Supplement: awab446_Supplementary_Data [file awab446_supplementary_data.zip › awab446 Supplementary_data 1.pdf]

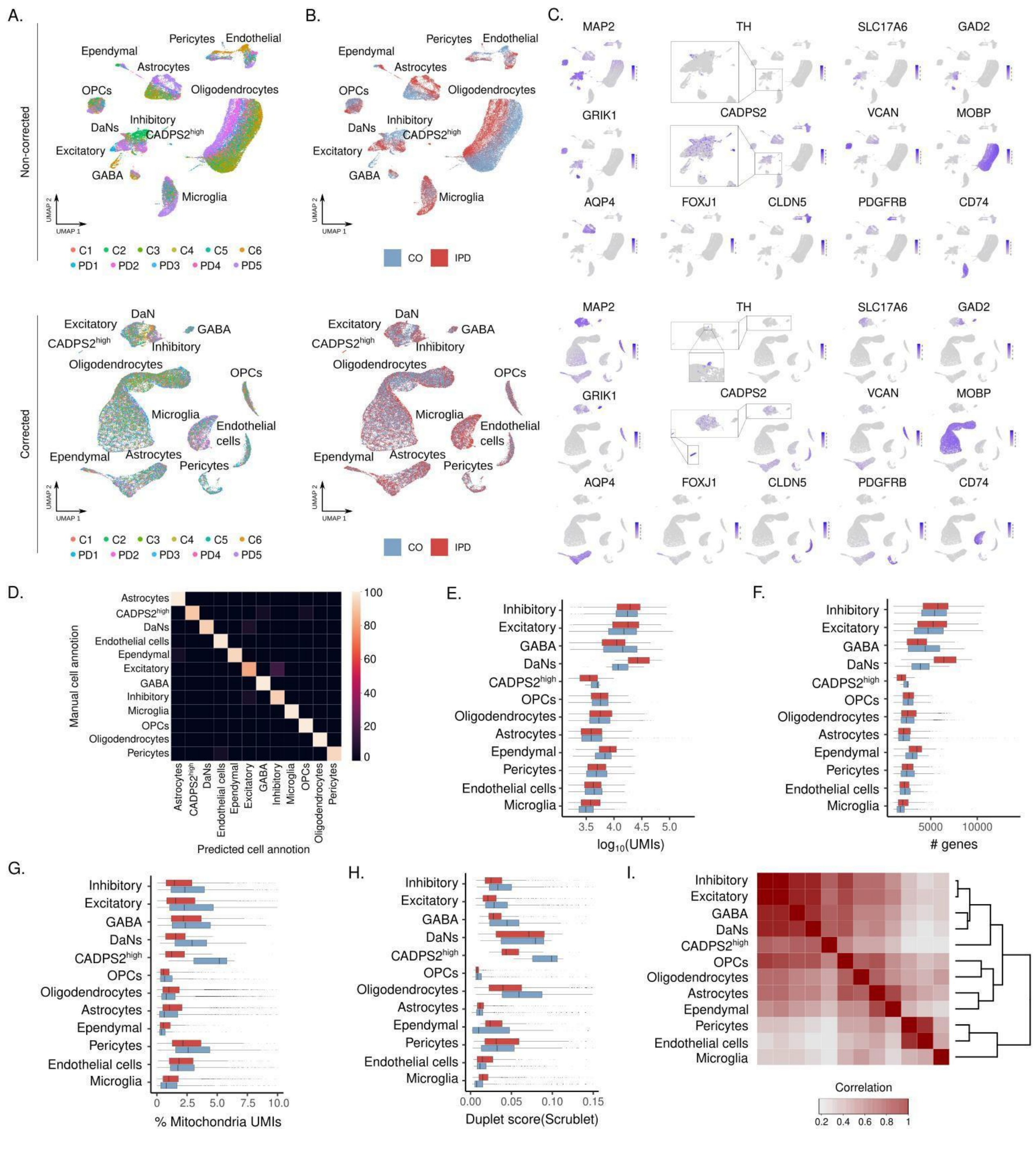

Supplementary Fig. 1 Midbrain cell UMAP embedding and cell type-specific snRNA-seq quality control metrics and similarity. (**A-B**) Midbrain single-cell atlas UMAP embedding colored by sample and condition. Top panels display UMAP embeddings based on the top 25 non-corrected principal components. Bottom panels show the UMAP embeddings based on the top 25 principal components after removing the inter-individual variability using the Seurat3 Canonical Correlation Analysis based integration protocol. (**C**) Expression distribution of cell type marker genes on the ~41,000 midbrain cells. (**D**) Confusion matrix results of the machine learning cross-validation approach to validate the cell type definition. (**E**) UMI count distribution. (**F**) Number of detected genes. (**G**) Percentage of mtDNA-encoded transcripts per cell. (**H**) Scrublet duplet score. (**I**) Unsupervised clustering of the midbrain cell-types based on their transcriptional pseudo-bulk profile correlation. CADPS2^{high} transcriptome profile clusters together with the neuronal cells.

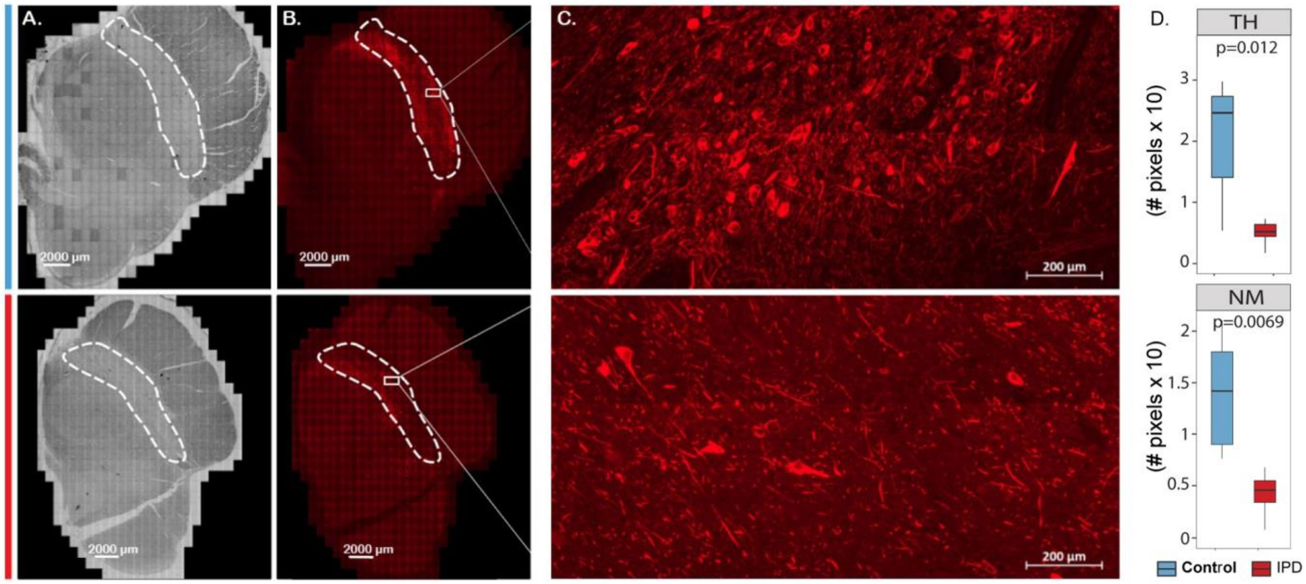

Supplementary Fig. 2 Immunofluorescent labelling of idiopathic Parkinson's disease (IPD) and control midbrain tissue with TH. (**A**) Representative control (top) and IPD (bottom) sections in brightfield reveal midbrain sub-areas. (**B**) Representative control (top) and IPD (bottom) sections stained for TH show a dopaminergic neuron reduction in IPD patients. (**C**) SN area of the control (top) and IPD (bottom) midbrain stained for TH. (**D**) Quantification of TH-positive and neuromelanin-containing neurons. Immunofluorescence analysis with an antibody targeting TH revealed loss of nigral DaNs in IPD samples compared to controls. Similarly, the number of DaNs with NM deposits was reduced in the SN of IPD patients. Scale bar = 2000μm (**A**, **B**); scale bar = 200μm (**C**). DaNs, dopaminergic neurons; IPD, idiopathic Parkinson’s disease; NM, neuromelanin; SN, substantia nigra; TH, tyrosine hydroxylase.

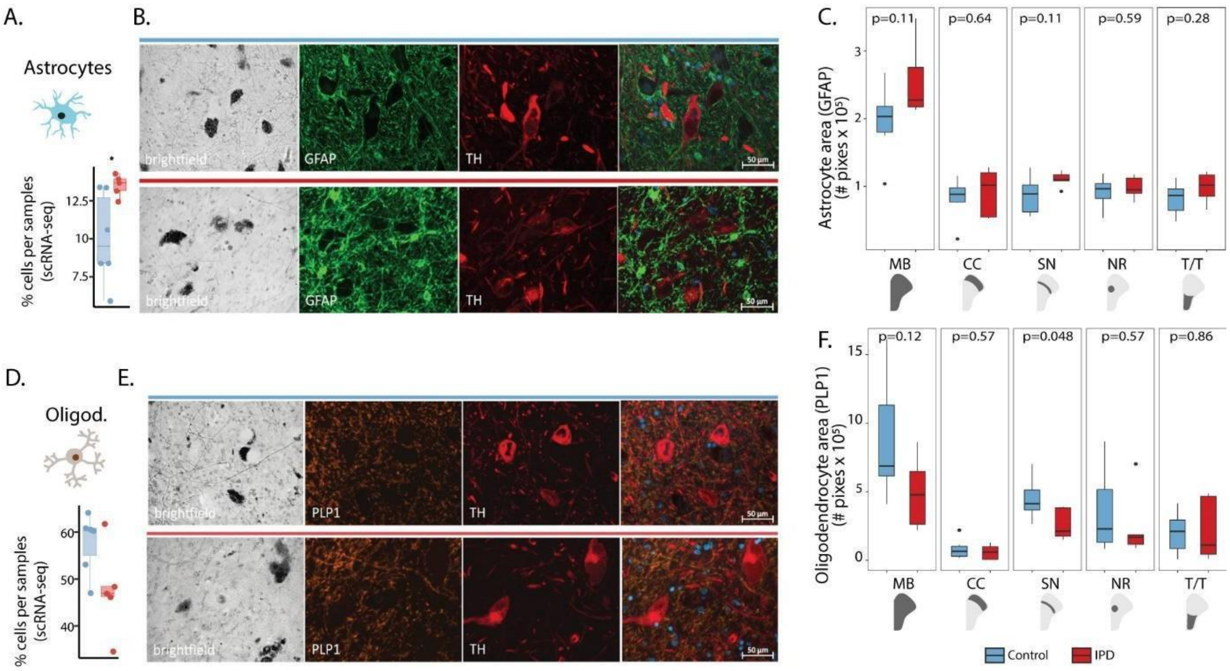

Supplementary Fig. 3 Quantitative immunofluorescence analysis of control and idiopathic Parkinson's disease (IPD) midbrain astrocytes and oligodendrocytes. (**A**) Astrocyte cell proportion per sample. IPD patients display a higher proportion of astrocyte cells (t-test p = 0.03). (**B**) Representative control and IPD sections in brightfield (neuromelanin deposits) or stained for GFAP (astrocytes) and TH (DaNs). (**C**) GFAP astrocyte area quantification. (**D**) Oligodendrocytecell proportion per sample. IPD patients display a lower proportion of oligodendrocyte cells (t-test p = 0.08). (**E**) Representative control and IPD sections in brightfield (neuromelanin deposits) or stained for PLP1 (oligodendrocytes) and TH (DaNs). (**F**) PLP1 oligodendrocyte area quantification. MB, midbrain; SN, substantia nigra; NR, nucleus ruber; T/T, tectum/tegmentum; CC, crus cerebri; PD, red bar; control, blue bar; scale bar = 50µm

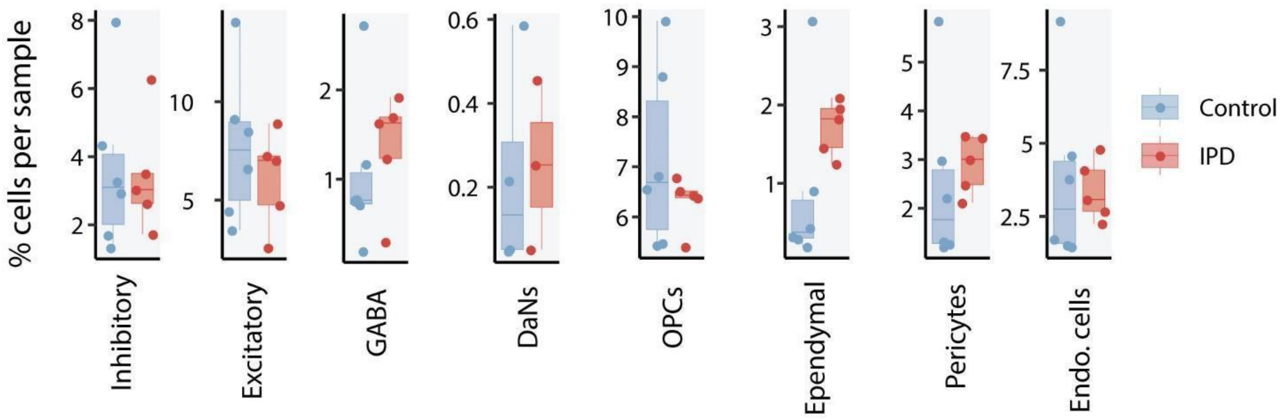

Supplementary Fig. 4 Idiopathic Parkinson's disease (IPD) differential cell-type composition. The percentage of cells per sample is presented for control individuals and IPD patients. The percentage of inhibitory, excitatory, GABA and dopaminergic (DaNs) neurons is given alongside the proportion of Oligodendrocyte precursor cells (OPCs), ependymal, pericytes and endothelial cells.

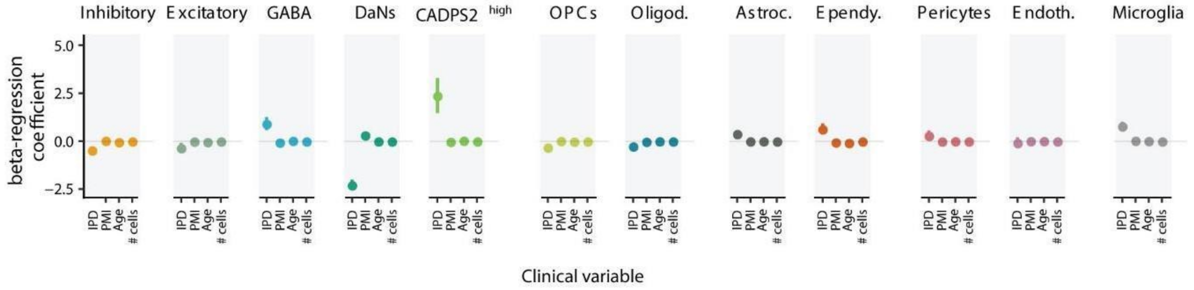

Supplementary Fig. 5 Cell type proportion beta-regression modeling estimates for the clinical variables: disease condition (IPD), PMI, age and number of cells detected.

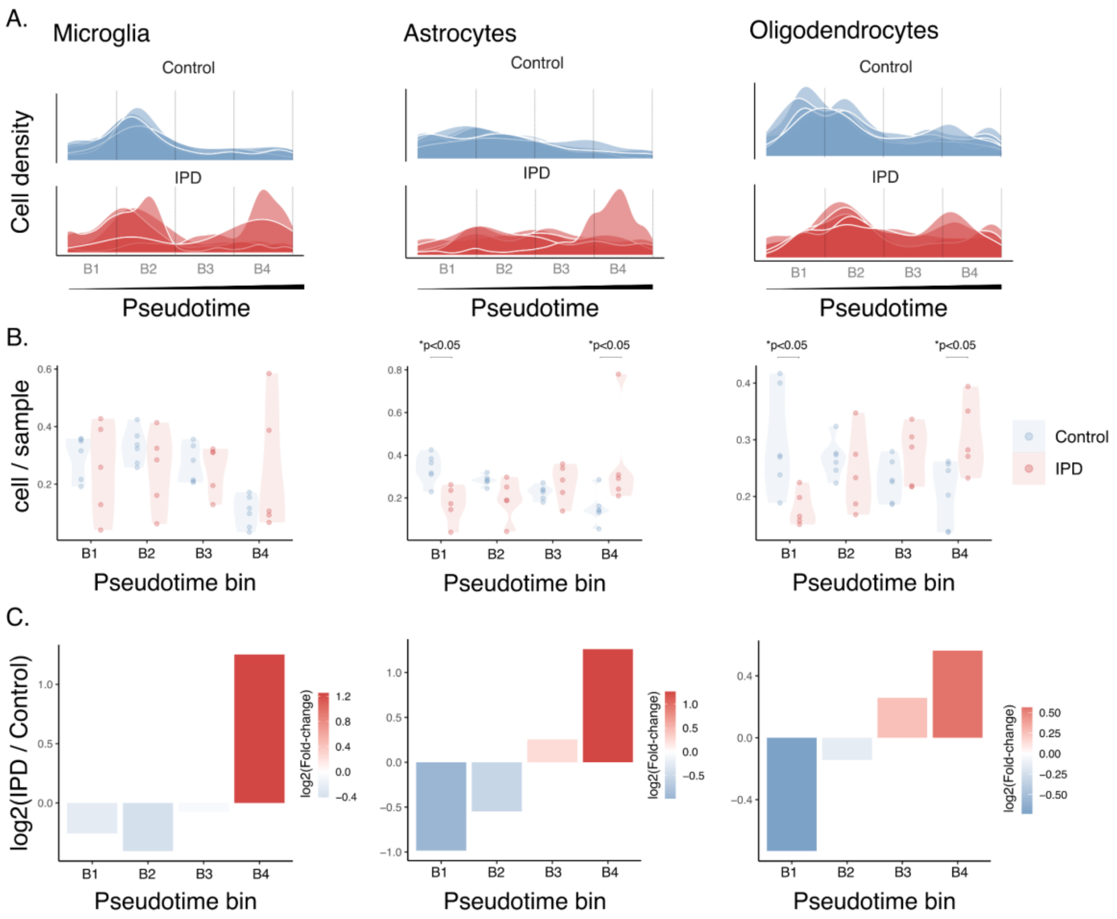

Supplementary Figure 6. Sample heterogeneity of microglia, astrocyte, and oligodendrocyte distribution along their respective activation trajectories. (**A**) Cellular density distribution along the activation trajectory per sample for controls and IPD patients. Four pseudotime bins are indicated. (**B**) Fraction of cells per sample for each pseudotime bin. IPD patient and control distributions per bin were compared using the Wilcoxon test. (**C**) Differential distribution of the IPD cells along the four pseudotime bins of the activation trajectory. Bar plots represent the log2 IPD fold-change of the mean fraction of cells per sample for each pseudotime bin.

Supplementary Table 1 Patient Information

Patient	Sex	Age at onset (years)	Age at death (years)	Disease duration (years)	PMI (h)	Lewy body Braak stage	Non-motor features	Profiled nuclei	SN neuron loss
**Single-cell RNA sequenced cohort**									
IPD1	F	71	84	13	25	5	Depression, visual hallucinations	1943	severe
IPD2	M	57	66	10	24	6	Hallucinations, paranoia	5925	severe
IPD3	M	71	77	7	24	6	Stroke, cognitive impairment, dementia	3345	severe
IPD4	M	69	81	12	13	6	Cognitive impairment, hallucinations, depression	2083	severe
IPD5	M	71	79	8	25	5	Blindness, memory impairment	5706	severe
	**Mean**	**67,8**	**77,4**	**10**	**22,2**			**3800,4**	
	**SEM**	**2,7**	**3**	**1,1**	**2,3**			**858,8**	
C1	F	-	93	-	29	-	-	3753	mild
C2	M	-	66	-	16	-	-	4358	none
C3	M	-	77	-	22	-	-	2047	none
C4	M	-	84	-	5	-	-	4199	none
C5	M	-	88	-	8	-	-	2318	none
C6	M	-	90	-	12	-	-	5758	none
	**Mean**		**83**		**15,3**			**3738,8**	
	**SEM**		**4**		**3,6**			**564,3**	

Patient	Sex	Age at onset (years)	Age at death (years)	Disease duration (years)	PMI (h)	Lewy body Braak stage	Non-motor features	Dissected DaNs	SN neuron loss
**LCM validation cohort**									
IPD4	M	69	81	12	13	6	Cognitive impairment, hallucinations, depression	150	severe
IPD6	M	56	61	5	90	4	hypophonia	150	severe
IPD7	M	70	80	10	34	6	Impaired memory, hallucinations	150	severe
IPD8	M	67	72	5	41	4	No cognitive impairments	150	severe
IPD9	F	66	81	15	35	6	dementia, impaired consciousness, auditory and visual hallucinations	150	severe
C1	F	-	93	-	29	-	-	150	mild
C7	M	-	81	-	34	-	Cognitive decline	150	none
C8	M	-	90	-	80	-	-	150	none

C9	M	-	96	-	16	-	-	150	none
C10	M	-	92	-	9	-		150	none

IPD: idiopathic Parkinson's disease
C: control
PMI: post-mortem interval
SN: substantia nigra
LCM: laser capture microdissection

Supplementary Table 2 Genotyping results in IPD patients

Sample	Genomic position Zygosity	Gene	Exonic variant type	Aminoacid change	GnomAD Exon NFE	GnomAD Genome NFE	ClinVar
IPD4	1:153066050A>C het	SPRR2E	nonsyn	p.S60A (NM_001024209)	0.0021	0.0011	
IPD1	2:40653336G>T het	LRRK2	nonsyn	p.M491I (NM_198578)	8.99E-05	6.67E-05	
IPD5	115:62261612T>C het	VPS13C	nonsyn	p.T890A (NM_017684)	0.0053	0.009	
IPD5	16:12798607A>G het	CPPED1	nonsyn	p.C197R (NM_018340)	0.0045	0.0043	
IPD2	5:121786403C>T het	SNCAIP	nonsyn	p.R255C (NM_001242935)	0.0054	0.0051	PD late onset
IPD2	5:141694021G>T het	SPRY4	nonsyn	p.S218Y (NM_001127496)	0.0061	0.0078	Hypogonado-tropic hypogonadism
IPD2	6:136882715C>T het	MAP3K5	nonsyn	p.D1315N (NM_005923)	0.0084	0.0069	

gnomAD: genome annotation databse
NFE: non-Finish European ethnic group

Supplementary Table 3 Gene expression markers of the midbrain comprising cell types

Cell type	Gene name	Gene ensembl ID	myAUC	avg_diff	Power	pct.1	pct.2
Oligodendrocytes	ENPP2	ENSG00000136960	0.977	2.235075586	0.954	0.967	0.098
Oligodendrocytes	ST18	ENSG00000147488	0.994	3.294565969	0.988	0.996	0.139
Oligodendrocytes	MOBP	ENSG00000168314	0,967	2,58678107	0,934	0,95	0,1
Oligodendrocytes	MOG	ENSG00000204655	0,933	1,776167992	0,866	0,88	0,04
Oligodendrocytes	PCSK6	ENSG00000140479	0,947	2,347402667	0,894	0,92	0,11
Oligodendrocytes	SLC5A11	ENSG00000158865	0,929	2,441596733	0,858	0,88	0,07
Oligodendrocytes	UGT8	ENSG00000174607	0,947	1,932001447	0,894	0,92	0,12
Oligodendrocytes	ANLN	ENSG00000011426	0,928	1,798336266	0,856	0,88	0,08
Oligodendrocytes	SYNJ2	ENSG00000078269	0,955	2,138356041	0,91	0,93	0,14
Oligodendrocytes	CNTN2	ENSG00000184144	0,952	1,873124432	0,904	0,93	0,15
Oligodendrocytes	MAG	ENSG00000105695	0,904	1,757176387	0,808	0,83	0,05
Oligodendrocytes	CNDP1	ENSG00000150656	0,9	1,757811884	0,8	0,82	0,04
Oligodendrocytes	CDK18	ENSG00000117266	0,954	2,078721588	0,908	0,93	0,16
Oligodendrocytes	FA2H	ENSG00000103089	0,913	1,590872312	0,826	0,87	0,09
Oligodendrocytes	SLCO1A2	ENSG00000084453	0,912	1,646108064	0,824	0,87	0,1
Oligodendrocytes	C10orf90	ENSG00000154493	0,963	2,037208564	0,926	0,97	0,19
Oligodendrocytes	MYRF	ENSG00000124920	0,898	1,480018545	0,796	0,82	0,04
Oligodendrocytes	ABCA8	ENSG00000141338	0,898	1,535561375	0,796	0,83	0,06
Oligodendrocytes	FAM107B	ENSG00000065809	0,943	1,81357168	0,886	0,93	0,16
Oligodendrocytes	AC026316.4	ENSG00000285218	0,928	2,278849715	0,856	0,89	0,13
Oligodendrocytes	CLMN	ENSG00000165959	0,94	1,825492906	0,88	0,95	0,19
Oligodendrocytes	TMEM144	ENSG00000164124	0,981	2,339162961	0,962	0,98	0,22
Oligodendrocytes	CD22	ENSG00000012124	0,884	1,430435733	0,768	0,79	0,03
Oligodendrocytes	CERCAM	ENSG00000167123	0,924	1,733621005	0,848	0,89	0,14
Oligodendrocytes	TF	ENSG00000091513	0,981	2,47039125	0,962	0,98	0,23
Oligodendrocytes	ZNF536	ENSG00000198597	0,957	1,926078139	0,914	0,96	0,21
Oligodendrocytes	SH3TC2	ENSG00000169247	0,882	1,452266349	0,764	0,78	0,03
Oligodendrocytes	LRP2	ENSG00000081479	0,887	1,809192526	0,774	0,79	0,05
Oligodendrocytes	PIEZO2	ENSG00000154864	0,884	1,69566892	0,768	0,79	0,05
Oligodendrocytes	BCAS1	ENSG00000064787	0,901	1,473374593	0,802	0,91	0,17
Oligodendrocytes	HAPLN2	ENSG00000132702	0,897	1,644242222	0,794	0,83	0,09
Oligodendrocytes	LINC00639	ENSG00000259070	0,878	1,569803014	0,756	0,79	0,05
Oligodendrocytes	SHROOM4	ENSG00000158352	0,899	1,593097624	0,798	0,87	0,14
Oligodendrocytes	FGFR2	ENSG00000066468	0,917	1,709726887	0,834	0,89	0,16
Oligodendrocytes	PLEKHH1	ENSG00000054690	0,953	1,884696908	0,906	0,94	0,21
Oligodendrocytes	NKX6-2	ENSG00000148826	0,87	1,416415787	0,74	0,76	0,04
Oligodendrocytes	LDB3	ENSG00000122367	0,867	1,465226066	0,734	0,75	0,04
Oligodendrocytes	PDE1C	ENSG00000154678	0,927	2,049363969	0,854	0,93	0,22
Oligodendrocytes	FOLH1	ENSG00000086205	0,863	1,466844663	0,726	0,74	0,03
Oligodendrocytes	PLD1	ENSG00000075651	0,895	1,519277677	0,79	0,87	0,16
Oligodendrocytes	COL4A5	ENSG00000188153	0,896	1,477472114	0,792	0,88	0,17
Oligodendrocytes	CTNNA3	ENSG00000183230	0,993	3,118061362	0,986	1	0,29
Oligodendrocytes	LINC00609	ENSG00000257585	0,902	2,066476511	0,804	0,85	0,15
Oligodendrocytes	ERMN	ENSG00000136541	0,863	1,416798769	0,726	0,75	0,05
Oligodendrocytes	CARNS1	ENSG00000172508	0,863	1,482623168	0,726	0,76	0,06
Oligodendrocytes	AC009063.2	ENSG00000260788	0,856	1,465175447	0,712	0,73	0,03
Oligodendrocytes	RAPGEF5	ENSG00000136237	0,94	1,819136703	0,88	0,95	0,25
Oligodendrocytes	ATP10B	ENSG00000118322	0,873	1,446988424	0,746	0,82	0,13
Oligodendrocytes	LPAR1	ENSG00000198121	0,869	1,387648352	0,738	0,84	0,14
Oligodendrocytes	CNP	ENSG00000173786	0,924	1,894812941	0,848	0,9	0,21
Oligodendrocytes	CREB5	ENSG00000146592	0,907	1,598161312	0,814	0,92	0,23

Oligodendrocytes	RNF220	ENSG00000187147	0,99	2,725357556	0,98	1	0,31
Oligodendrocytes	SH3GL3	ENSG00000140600	0,936	1,78129152	0,872	0,94	0,27
Oligodendrocytes	GRM3	ENSG00000198822	0,883	1,375437478	0,766	0,9	0,23
Oligodendrocytes	KCNH8	ENSG00000183960	0,872	1,599133366	0,744	0,85	0,18
Oligodendrocytes	SCD	ENSG00000099194	0,893	1,673034222	0,786	0,86	0,19
Oligodendrocytes	GPR37	ENSG00000170775	0,845	1,23923656	0,69	0,73	0,06
Oligodendrocytes	ABCA2	ENSG00000107331	0,946	1,719217176	0,892	0,95	0,28
Oligodendrocytes	PEX5L	ENSG00000114757	0,976	2,303625915	0,952	0,99	0,33
Oligodendrocytes	CNTNAP4	ENSG00000152910	0,888	1,768043796	0,776	0,86	0,2
Oligodendrocytes	FAM124A	ENSG00000150510	0,865	1,349130653	0,73	0,79	0,13
Oligodendrocytes	TTYH2	ENSG00000141540	0,861	1,218440429	0,722	0,82	0,17
Oligodendrocytes	APLP1	ENSG00000105290	0,927	1,793199921	0,854	0,91	0,26
Oligodendrocytes	PLP1	ENSG00000123560	0,985	3,030458567	0,97	0,99	0,34
Oligodendrocytes	QDPR	ENSG00000151552	0,922	1,908551315	0,844	0,91	0,26
Oligodendrocytes	PRR5L	ENSG00000135362	0,853	1,355347897	0,706	0,78	0,14
Oligodendrocytes	ITGA2	ENSG00000164171	0,831	1,331130911	0,662	0,72	0,08
Oligodendrocytes	CLDND1	ENSG00000080822	0,904	1,627655436	0,808	0,88	0,24
Oligodendrocytes	SLC24A2	ENSG00000155886	0,975	2,242823775	0,95	0,99	0,36
Oligodendrocytes	PRUNE2	ENSG00000106772	0,975	2,162611024	0,95	0,98	0,35
Oligodendrocytes	TMEM63A	ENSG00000196187	0,836	1,174947996	0,672	0,75	0,13
Oligodendrocytes	SELENOP	ENSG00000250722	0,854	1,587808346	0,708	0,79	0,16
Oligodendrocytes	MYO1D	ENSG00000176658	0,895	1,500390789	0,79	0,89	0,27
Oligodendrocytes	AL359091.1	ENSG00000207955	0,819	1,417318225	0,638	0,68	0,06
Oligodendrocytes	AK5	ENSG00000154027	0,866	1,435338775	0,732	0,84	0,23
Oligodendrocytes	NKAIN2	ENSG00000188580	0,917	1,727126438	0,834	0,96	0,35
Oligodendrocytes	NDRG1	ENSG00000104419	0,866	1,253531713	0,732	0,85	0,24
Oligodendrocytes	EDIL3	ENSG00000164176	0,978	2,077058104	0,956	0,99	0,39
Oligodendrocytes	KIRREL3	ENSG00000149571	0,837	1,443408156	0,674	0,85	0,24
Oligodendrocytes	EFHD1	ENSG00000115468	0,835	1,174909083	0,67	0,8	0,19
Oligodendrocytes	COBL	ENSG00000106078	0,849	1,16188646	0,698	0,89	0,28
Oligodendrocytes	MAL	ENSG00000172005	0,811	1,160629111	0,622	0,67	0,07
Oligodendrocytes	DAAM2	ENSG00000146122	0,826	0,990998099	0,652	0,9	0,3
Oligodendrocytes	SRCIN1	ENSG00000277363	0,873	1,3377086	0,746	0,83	0,23
Oligodendrocytes	DOCK5	ENSG00000147459	0,943	1,720383305	0,886	0,98	0,38
Oligodendrocytes	PLAAT3	ENSG00000176485	0,851	1,187239936	0,702	0,82	0,23
Oligodendrocytes	NECAB1	ENSG00000123119	0,818	1,183300194	0,636	0,73	0,14
Oligodendrocytes	PXK	ENSG00000168297	0,949	1,723215631	0,898	0,96	0,37
Oligodendrocytes	FRMD4B	ENSG00000114541	0,937	1,680495014	0,874	0,98	0,39
Oligodendrocytes	PPP1R14A	ENSG00000167641	0,805	1,124545912	0,61	0,69	0,1
Oligodendrocytes	SEMA3B	ENSG00000012171	0,804	1,046146446	0,608	0,69	0,1
Oligodendrocytes	AATK	ENSG00000181409	0,86	1,218118025	0,72	0,81	0,22
Oligodendrocytes	LRRC63	ENSG00000173988	0,805	1,25838343	0,61	0,64	0,05
Oligodendrocytes	KCNMB4	ENSG00000135643	0,822	1,477257384	0,644	0,72	0,14
Oligodendrocytes	MEGF10	ENSG00000145794	0,829	1,083061943	0,658	0,82	0,24
Oligodendrocytes	POLR2F	ENSG00000100142	0,864	1,56208217	0,728	0,82	0,23
Oligodendrocytes	DSCAML1	ENSG00000177103	0,858	1,231309362	0,716	0,92	0,33
Oligodendrocytes	HHIP	ENSG00000164161	0,807	1,454279321	0,614	0,66	0,08
Oligodendrocytes	CRYAB	ENSG00000109846	0,859	1,657299097	0,718	0,84	0,26
Oligodendrocytes	HDAC2-AS2	ENSG00000228624	0,834	1,541394988	0,668	0,76	0,18
Oligodendrocytes	AC016597.1	ENSG00000261329	0,793	1,08451799	0,586	0,61	0,03
Oligodendrocytes	SPOCK3	ENSG00000196104	0,924	1,580612112	0,848	0,98	0,41
Oligodendrocytes	NDE1	ENSG00000072864	0,804	1,059343866	0,608	0,7	0,14
Oligodendrocytes	HS3ST5	ENSG00000249853	0,808	1,230152296	0,616	0,74	0,18
Oligodendrocytes	GLDN	ENSG00000186417	0,793	0,773659929	0,586	0,77	0,21
Oligodendrocytes	TTLL7	ENSG00000137941	0,963	1,876115312	0,926	0,98	0,42

Oligodendrocytes	ARHGAP23	ENSG00000275832	0,826	1,096360027	0,652	0,75	0,19
Oligodendrocytes	AMER2	ENSG00000165566	0,843	1,299817317	0,686	0,81	0,25
Oligodendrocytes	ATP8A1	ENSG00000124406	0,957	1,688477577	0,914	0,98	0,42
Oligodendrocytes	SEC14L5	ENSG00000103184	0,785	1,015275926	0,57	0,59	0,04
Oligodendrocytes	AC096564.1	ENSG00000245293	0,801	1,125418638	0,602	0,65	0,1
Oligodendrocytes	LANCL1	ENSG00000115365	0,892	1,437643125	0,784	0,88	0,33
Oligodendrocytes	VRK2	ENSG00000028116	0,8	1,170662791	0,6	0,74	0,19
Oligodendrocytes	COLGALT2	ENSG00000198756	0,844	1,239225585	0,688	0,82	0,27
Oligodendrocytes	SLAIN1	ENSG00000139737	0,942	1,77001968	0,884	0,96	0,42
Oligodendrocytes	PPP1R16B	ENSG00000101445	0,822	1,114420225	0,644	0,76	0,22
Oligodendrocytes	UNC5C	ENSG00000182168	0,906	1,571654934	0,812	0,97	0,42
Oligodendrocytes	GNAI1	ENSG00000127955	0,844	1,203692556	0,688	0,81	0,27
Oligodendrocytes	KIF6	ENSG00000164627	0,813	1,219756066	0,626	0,74	0,2
Oligodendrocytes	ASPA	ENSG00000108381	0,774	1,002238372	0,548	0,58	0,04
Oligodendrocytes	AC079352.1	ENSG00000226994	0,778	1,370024902	0,556	0,63	0,09
Oligodendrocytes	LAMP2	ENSG00000005893	0,872	1,305184289	0,744	0,88	0,35
Oligodendrocytes	TMEFF2	ENSG00000144339	0,809	1,083986066	0,618	0,86	0,32
Oligodendrocytes	SHTN1	ENSG00000187164	0,933	1,527112743	0,866	0,98	0,45
Oligodendrocytes	ANK3	ENSG00000151150	0,776	0,896115701	0,552	0,96	0,43
Oligodendrocytes	RAB40B	ENSG00000141542	0,802	1,021236158	0,604	0,69	0,16
Oligodendrocytes	DPYSL5	ENSG00000157851	0,831	1,434099493	0,662	0,75	0,22
Oligodendrocytes	IGSF11	ENSG00000144847	0,811	1,037731149	0,622	0,81	0,29
Oligodendrocytes	IL1RAPL1	ENSG00000169306	0,961	1,946994182	0,922	1	0,47
Oligodendrocytes	ZCCHC24	ENSG00000165424	0,777	0,768666012	0,554	0,8	0,27
Oligodendrocytes	MAN2A1	ENSG00000112893	0,975	2,284350655	0,95	0,99	0,46
Oligodendrocytes	AC004690.2	ENSG00000241345	0,764	1,035420366	0,528	0,54	0,01
Oligodendrocytes	LINC01630	ENSG00000227115	0,771	1,172694213	0,542	0,58	0,06
Oligodendrocytes	LINC01170	ENSG00000253807	0,774	1,445932272	0,548	0,61	0,08
Oligodendrocytes	CDKN1C	ENSG00000129757	0,773	1,051779249	0,546	0,6	0,08
Oligodendrocytes	TMEM98	ENSG00000006042	0,766	1,094707955	0,532	0,56	0,03
Oligodendrocytes	RASGRF2	ENSG00000113319	0,813	1,180286982	0,626	0,79	0,26
Oligodendrocytes	SEMA4D	ENSG00000187764	0,857	1,242445304	0,714	0,85	0,32
Oligodendrocytes	TMTC4	ENSG00000125247	0,783	0,997477597	0,566	0,65	0,13
Oligodendrocytes	TMTC2	ENSG00000179104	0,961	1,963585141	0,922	0,99	0,47
Oligodendrocytes	PLCL1	ENSG00000115896	0,962	2,082854055	0,924	0,99	0,47
Oligodendrocytes	JAM3	ENSG00000166086	0,843	1,152162546	0,686	0,83	0,31
Oligodendrocytes	RFTN2	ENSG00000162944	0,79	0,878673306	0,58	0,84	0,32
Oligodendrocytes	MYO1E	ENSG00000157483	0,778	0,744198428	0,556	0,82	0,3
Oligodendrocytes	PLLP	ENSG00000102934	0,765	0,89557781	0,53	0,67	0,16
Oligodendrocytes	TUBB4A	ENSG00000104833	0,796	1,177604079	0,592	0,69	0,18
Oligodendrocytes	KLHL4	ENSG00000102271	0,763	1,112117239	0,526	0,58	0,07
Oligodendrocytes	FRMD5	ENSG00000171877	0,961	1,816705997	0,922	0,99	0,49
Oligodendrocytes	RASGRP3	ENSG00000152689	0,761	0,872813977	0,522	0,63	0,12
Oligodendrocytes	CDH19	ENSG00000071991	0,756	1,02366294	0,512	0,55	0,04
Oligodendrocytes	FEZ1	ENSG00000149557	0,823	1,133435376	0,646	0,77	0,27
Oligodendrocytes	PHLDB1	ENSG00000019144	0,812	1,011449098	0,624	0,82	0,31
Oligodendrocytes	HSPA2	ENSG00000126803	0,76	0,932416537	0,52	0,6	0,09
Oligodendrocytes	HECW2	ENSG00000138411	0,807	1,047625145	0,614	0,82	0,32
Oligodendrocytes	ATP1B1	ENSG00000143153	0,816	0,985549819	0,632	0,86	0,36
Oligodendrocytes	DNAJC6	ENSG00000116675	0,932	1,582815259	0,864	0,96	0,46
Oligodendrocytes	MAP7	ENSG00000135525	0,979	1,860411091	0,958	0,99	0,5
Oligodendrocytes	NCKAP5	ENSG00000176771	0,947	1,875974997	0,894	0,98	0,48
Oligodendrocytes	KLK6	ENSG00000167755	0,749	0,886875454	0,498	0,51	0,02
Oligodendrocytes	TFEB	ENSG00000112561	0,759	0,802523725	0,518	0,67	0,18
Oligodendrocytes	RYBP	ENSG00000163602	0,85	1,169200721	0,7	0,86	0,37

Oligodendrocytes	SUN2	ENSG00000100242	0,78	0,947686832	0,56	0,73	0,24
Oligodendrocytes	GPRC5B	ENSG00000167191	0,811	0,944577709	0,622	0,87	0,38
Oligodendrocytes	KIF13B	ENSG00000197892	0,826	1,087681268	0,652	0,82	0,34
Oligodendrocytes	SLCO3A1	ENSG00000176463	0,879	1,239982407	0,758	0,94	0,45
Oligodendrocytes	TP53TG5	ENSG00000124251	0,752	0,956400273	0,504	0,54	0,05
Oligodendrocytes	TMEM165	ENSG00000134851	0,975	1,914374779	0,95	0,99	0,5
Oligodendrocytes	LIPA	ENSG00000107798	0,82	1,042001169	0,64	0,8	0,32
Oligodendrocytes	MBP	ENSG00000197971	0,994	2,48223528	0,988	1	0,52
Oligodendrocytes	AC090015.1	ENSG00000251138	0,746	1,243884593	0,492	0,51	0,02
Oligodendrocytes	CPOX	ENSG00000080819	0,774	1,026471899	0,548	0,63	0,15
Oligodendrocytes	RNASE1	ENSG00000129538	0,737	0,772262506	0,474	0,59	0,11
Oligodendrocytes	CDK19	ENSG00000155111	0,875	1,313022314	0,75	0,92	0,44
Oligodendrocytes	SPOCK1	ENSG00000152377	0,849	1,363458675	0,698	0,93	0,45
Oligodendrocytes	LINC01608	ENSG00000253877	0,742	1,625079096	0,484	0,5	0,03
Oligodendrocytes	KIAA1755	ENSG00000149633	0,737	0,663066822	0,474	0,68	0,21
Oligodendrocytes	MEIS1	ENSG00000143995	0,755	0,902440096	0,51	0,64	0,17
Oligodendrocytes	BIN1	ENSG00000136717	0,804	0,944210946	0,608	0,81	0,34
Oligodendrocytes	BOK	ENSG00000176720	0,747	0,879314996	0,494	0,55	0,07
Oligodendrocytes	SLC31A2	ENSG00000136867	0,739	0,801517376	0,478	0,52	0,05
Oligodendrocytes	PTPRD	ENSG00000153707	0,869	0,985826092	0,738	1	0,53
Oligodendrocytes	SLC12A2	ENSG00000064651	0,808	0,891376898	0,616	0,83	0,37
Oligodendrocytes	SEPTIN4	ENSG00000108387	0,757	0,877815185	0,514	0,61	0,14
Oligodendrocytes	RASSF2	ENSG00000101265	0,743	0,773041448	0,486	0,64	0,17
Oligodendrocytes	RHOU	ENSG00000116574	0,745	0,810202188	0,49	0,6	0,13
Oligodendrocytes	PSEN1	ENSG00000080815	0,845	1,107038208	0,69	0,86	0,39
Oligodendrocytes	RETREG1	ENSG00000154153	0,795	0,999037711	0,59	0,76	0,3
Oligodendrocytes	NFIX	ENSG00000008441	0,765	0,782787122	0,53	0,77	0,31
Oligodendrocytes	PTPRK	ENSG00000152894	0,891	1,207539923	0,782	0,98	0,51
Oligodendrocytes	AGPAT4	ENSG00000026652	0,842	1,35117944	0,684	0,82	0,35
Oligodendrocytes	ENOX1	ENSG00000120658	0,818	1,087198196	0,636	0,9	0,44
Oligodendrocytes	LINC00320	ENSG00000224924	0,739	1,01286454	0,478	0,54	0,07
Oligodendrocytes	C12orf76	ENSG00000174456	0,771	0,865882586	0,542	0,68	0,22
Oligodendrocytes	ARAP2	ENSG00000047365	0,875	1,290576066	0,75	0,95	0,49
Oligodendrocytes	FAM13C	ENSG00000148541	0,825	1,118116254	0,65	0,82	0,36
Oligodendrocytes	SOX10	ENSG00000100146	0,733	0,727668798	0,466	0,56	0,1
Oligodendrocytes	YPEL2	ENSG00000175155	0,789	0,982841403	0,578	0,76	0,3
Oligodendrocytes	LPGAT1	ENSG00000123684	0,844	1,189576015	0,688	0,86	0,4
Oligodendrocytes	GAS7	ENSG00000007237	0,772	0,851453281	0,544	0,75	0,29
Oligodendrocytes	ATG4C	ENSG00000125703	0,808	0,983771307	0,616	0,81	0,35
Oligodendrocytes	NINJ2	ENSG00000171840	0,733	0,894346052	0,466	0,51	0,05
Oligodendrocytes	ZEB2	ENSG00000169554	0,879	1,179753946	0,758	0,98	0,52
Oligodendrocytes	ENPP6	ENSG00000164303	0,733	0,902168463	0,466	0,48	0,02
Oligodendrocytes	PLEKHB1	ENSG00000021300	0,746	0,808490325	0,492	0,62	0,16
Oligodendrocytes	ARFGEF3	ENSG00000112379	0,803	0,901138773	0,606	0,85	0,4
Oligodendrocytes	NT5DC1	ENSG00000178425	0,813	1,093752987	0,626	0,8	0,35
Oligodendrocytes	CPB2-AS1	ENSG00000235903	0,76	0,990436905	0,52	0,63	0,18
Oligodendrocytes	GLTP	ENSG00000139433	0,754	0,846808514	0,508	0,62	0,17
Oligodendrocytes	KIAA1324L	ENSG00000164659	0,787	0,995889837	0,574	0,74	0,29
Oligodendrocytes	KLHL32	ENSG00000186231	0,862	1,288793	0,724	0,92	0,47
Oligodendrocytes	INF2	ENSG00000203485	0,738	0,741793393	0,476	0,59	0,14
Oligodendrocytes	SLC22A23	ENSG00000137266	0,839	1,103996677	0,678	0,87	0,42
Oligodendrocytes	PRIMA1	ENSG00000175785	0,728	0,800902306	0,456	0,48	0,04
Oligodendrocytes	HEPACAM	ENSG00000165478	0,725	0,5936985	0,45	0,68	0,23
Oligodendrocytes	HIP1	ENSG00000127946	0,759	0,700305694	0,518	0,87	0,42
Oligodendrocytes	PIP4K2A	ENSG00000150867	0,988	2,153074657	0,976	1	0,55

Oligodendrocytes	LRRC1	ENSG00000137269	0,779	0,932150092	0,558	0,75	0,3
Oligodendrocytes	PPM1H	ENSG00000111110	0,79	1,017618978	0,58	0,77	0,33
Oligodendrocytes	CSRP1	ENSG00000159176	0,731	0,682572134	0,462	0,69	0,25
Oligodendrocytes	MARCKSL1	ENSG00000175130	0,765	1,060498795	0,53	0,67	0,23
Oligodendrocytes	PMP22	ENSG00000109099	0,73	0,700725158	0,46	0,63	0,19
Oligodendrocytes	SEMA6A	ENSG00000092421	0,797	0,921193661	0,594	0,81	0,37
Oligodendrocytes	TPPP	ENSG00000171368	0,76	0,909393253	0,52	0,64	0,21
Oligodendrocytes	DOCK10	ENSG00000135905	0,928	1,453964029	0,856	0,99	0,55
Oligodendrocytes	RNF144A	ENSG00000151692	0,812	0,983740398	0,624	0,83	0,39
Oligodendrocytes	LSS	ENSG00000160285	0,778	0,928789184	0,556	0,72	0,28
Oligodendrocytes	AL033523.1	ENSG00000228793	0,72	0,878490094	0,44	0,46	0,03
Oligodendrocytes	CA2	ENSG00000104267	0,724	0,841463202	0,448	0,52	0,09
Oligodendrocytes	P2RX7	ENSG00000089041	0,75	0,733216591	0,5	0,75	0,32
Oligodendrocytes	S100B	ENSG00000160307	0,766	1,201064948	0,532	0,72	0,28
Oligodendrocytes	ARHGEF2	ENSG00000116584	0,736	0,721761792	0,472	0,63	0,19
Oligodendrocytes	EPCAM-DT	ENSG00000234690	0,724	0,836302569	0,448	0,51	0,08
Oligodendrocytes	FAXDC2	ENSG00000170271	0,731	0,742912814	0,462	0,65	0,22
Oligodendrocytes	GAB1	ENSG00000109458	0,908	1,391257593	0,816	0,96	0,53
Oligodendrocytes	PDE1A	ENSG00000115252	0,726	0,926212937	0,452	0,59	0,16
Oligodendrocytes	DOCK1	ENSG00000150760	0,785	0,802619261	0,57	0,92	0,49
Oligodendrocytes	ZDHHC20	ENSG00000180776	0,86	1,583938221	0,72	0,85	0,42
Oligodendrocytes	NCOA7	ENSG00000111912	0,829	1,055080642	0,658	0,86	0,43
Oligodendrocytes	ADIPOR2	ENSG00000006831	0,813	0,880621801	0,626	0,88	0,45
Oligodendrocytes	GPM6B	ENSG00000046653	0,934	1,620537198	0,868	0,99	0,56
Oligodendrocytes	CCP110	ENSG00000103540	0,763	1,029349175	0,526	0,66	0,24
Oligodendrocytes	TMC7	ENSG00000170537	0,723	0,746526892	0,446	0,51	0,08
Oligodendrocytes	USP31	ENSG00000103404	0,779	1,032323482	0,558	0,71	0,28
Oligodendrocytes	SEMA3C	ENSG00000075223	0,717	0,816009981	0,434	0,51	0,08
Oligodendrocytes	CPNE2	ENSG00000140848	0,727	0,716747843	0,454	0,57	0,14
Oligodendrocytes	MARCH1	ENSG00000145416	0,782	0,805826498	0,564	0,97	0,54
Oligodendrocytes	GNAO1	ENSG00000087258	0,816	1,095853451	0,632	0,89	0,47
Oligodendrocytes	B3GAT1	ENSG00000109956	0,753	0,811107304	0,506	0,65	0,23
Oligodendrocytes	CORO2B	ENSG00000103647	0,763	0,895538394	0,526	0,7	0,28
Oligodendrocytes	FBXL7	ENSG00000183580	0,765	0,828315963	0,53	0,88	0,46
Oligodendrocytes	TRIM2	ENSG00000109654	0,903	1,307918774	0,806	0,97	0,55
Oligodendrocytes	ELMO1	ENSG00000155849	0,884	1,19386876	0,768	0,99	0,57
Oligodendrocytes	SLC44A1	ENSG00000070214	0,992	2,258648496	0,984	1	0,58
Oligodendrocytes	CBR1	ENSG00000159228	0,721	0,770493724	0,442	0,53	0,12
Oligodendrocytes	ERBB3	ENSG00000065361	0,709	0,684595519	0,418	0,46	0,05
Oligodendrocytes	TSPAN15	ENSG00000099282	0,709	0,656436481	0,418	0,49	0,08
Oligodendrocytes	OTUD7A	ENSG00000169918	0,862	1,176053423	0,724	0,93	0,52
Oligodendrocytes	SORCS2	ENSG00000184985	0,731	0,797627619	0,462	0,65	0,24
Oligodendrocytes	CHADL	ENSG00000100399	0,711	0,683868551	0,422	0,48	0,07
Oligodendrocytes	IQGAP1	ENSG00000140575	0,78	0,8455834	0,56	0,81	0,4
Oligodendrocytes	PACS2	ENSG00000179364	0,827	1,10999264	0,654	0,83	0,42
Oligodendrocytes	TTLL11	ENSG00000175764	0,77	0,94726702	0,54	0,7	0,29
Oligodendrocytes	SH3TC2-DT	ENSG00000250072	0,705	0,793168704	0,41	0,42	0,01
Oligodendrocytes	MVB12B	ENSG00000196814	0,872	1,247406856	0,744	0,91	0,5
Oligodendrocytes	FGF1	ENSG00000113578	0,716	0,693082144	0,432	0,6	0,2
Oligodendrocytes	LIMCH1	ENSG00000064042	0,896	1,215625936	0,792	0,98	0,57
Oligodendrocytes	NFASC	ENSG00000163531	0,795	0,968051538	0,59	0,89	0,48
Oligodendrocytes	DNM3	ENSG00000197959	0,922	1,269940908	0,844	0,99	0,59
Oligodendrocytes	LGR5	ENSG00000139292	0,715	0,876885981	0,43	0,51	0,11
Oligodendrocytes	AKAP6	ENSG00000151320	0,854	0,997524059	0,708	0,97	0,56
Oligodendrocytes	FBXO32	ENSG00000156804	0,724	0,990142708	0,448	0,56	0,16

Oligodendrocytes	LIPE	ENSG00000079435	0,712	0,723326433	0,424	0,49	0,09
Oligodendrocytes	GATM	ENSG00000171766	0,714	0,648280927	0,428	0,6	0,19
Oligodendrocytes	STMN1	ENSG00000117632	0,75	0,98342884	0,5	0,65	0,25
Oligodendrocytes	PIK3C2B	ENSG00000133056	0,731	0,779270748	0,462	0,61	0,21
Oligodendrocytes	NPC1	ENSG00000141458	0,748	0,729239453	0,496	0,74	0,34
Oligodendrocytes	SEPTIN8	ENSG00000164402	0,76	0,883235343	0,52	0,7	0,3
Oligodendrocytes	SECISBP2L	ENSG00000138593	0,817	0,963817803	0,634	0,83	0,44
Oligodendrocytes	DPYD	ENSG00000188641	0,89	1,224369852	0,78	0,98	0,58
Oligodendrocytes	GRID1	ENSG00000182771	0,817	0,962111698	0,634	0,92	0,53
Oligodendrocytes	ZNF708	ENSG00000182141	0,761	0,86484406	0,522	0,71	0,31
Oligodendrocytes	FAM102A	ENSG00000167106	0,721	0,710152594	0,442	0,58	0,18
Oligodendrocytes	APBB2	ENSG00000163697	0,831	0,920908209	0,662	0,95	0,56
Oligodendrocytes	SGK1	ENSG00000118515	0,793	1,08844688	0,586	0,86	0,47
Oligodendrocytes	OLMALINC	ENSG00000235823	0,721	0,787928994	0,442	0,65	0,25
Oligodendrocytes	ELOVL1	ENSG00000066322	0,702	0,670083375	0,404	0,48	0,09
Oligodendrocytes	PHACTR3	ENSG00000087495	0,76	0,720419701	0,52	0,86	0,47
Oligodendrocytes	DUBR	ENSG00000243701	0,746	0,825238972	0,492	0,69	0,3
Oligodendrocytes	NEO1	ENSG00000067141	0,8	0,869323968	0,6	0,88	0,49
Oligodendrocytes	SLC22A15	ENSG00000163393	0,76	0,830441678	0,52	0,75	0,36
Oligodendrocytes	SLC48A1	ENSG00000211584	0,714	0,687496637	0,428	0,57	0,18
Oligodendrocytes	KIF5C	ENSG00000168280	0,764	0,835417045	0,528	0,77	0,38
Oligodendrocytes	FAM95C	ENSG00000283486	0,714	0,976287771	0,428	0,49	0,1
Oligodendrocytes	CAMK2N1	ENSG00000162545	0,736	0,739027312	0,472	0,7	0,31
Oligodendrocytes	SAMD12	ENSG00000177570	0,824	1,047900204	0,648	0,89	0,51
Oligodendrocytes	TUBA1A	ENSG00000167552	0,784	1,161217668	0,568	0,77	0,39
Oligodendrocytes	SGK3	ENSG00000104205	0,817	0,99580427	0,634	0,86	0,48
Oligodendrocytes	LINC00877	ENSG00000241163	0,702	0,851328337	0,404	0,45	0,06
Oligodendrocytes	USP54	ENSG00000166348	0,836	0,943643411	0,672	0,93	0,54
Oligodendrocytes	RHOBTB1	ENSG00000072422	0,704	0,775109889	0,408	0,53	0,15
Oligodendrocytes	AGTPBP1	ENSG00000135049	0,818	1,00189802	0,636	0,87	0,49
Oligodendrocytes	FOXN2	ENSG00000170802	0,728	0,66218979	0,456	0,72	0,34
Oligodendrocytes	CPEB2	ENSG00000137449	0,776	0,869895756	0,552	0,78	0,4
Oligodendrocytes	MIR181A1HG	ENSG00000229989	0,73	0,738415306	0,46	0,75	0,37
Oligodendrocytes	ACTN2	ENSG00000077522	0,703	0,786133395	0,406	0,5	0,12
Oligodendrocytes	FUT8	ENSG00000033170	0,884	1,273133709	0,768	0,94	0,56
Oligodendrocytes	TMEM151A	ENSG00000179292	0,705	0,744498373	0,41	0,47	0,09
Oligodendrocytes	TECPR2	ENSG00000196663	0,747	0,798332733	0,494	0,71	0,33
Oligodendrocytes	SIRT2	ENSG00000068903	0,742	0,772678991	0,484	0,67	0,29
Oligodendrocytes	PDE8A	ENSG00000073417	0,883	1,228590709	0,766	0,96	0,58
Oligodendrocytes	GSN	ENSG00000148180	0,775	0,767931408	0,55	0,87	0,49
Oligodendrocytes	NXPE3	ENSG00000144815	0,732	0,757110903	0,464	0,63	0,25
Oligodendrocytes	MOB3B	ENSG00000120162	0,779	0,910308392	0,558	0,82	0,44
Oligodendrocytes	ZDHHC9	ENSG00000188706	0,707	0,700857051	0,414	0,52	0,14
Oligodendrocytes	BTBD3	ENSG00000132640	0,732	0,737966527	0,464	0,67	0,3
Oligodendrocytes	FAM222A	ENSG00000139438	0,702	0,669818201	0,404	0,5	0,13
Oligodendrocytes	ELAVL3	ENSG00000196361	0,741	0,735932332	0,482	0,75	0,38
Oligodendrocytes	TMCC2	ENSG00000133069	0,707	0,693814482	0,414	0,51	0,14
Oligodendrocytes	OTUD7B	ENSG00000264522	0,74	0,706152748	0,48	0,73	0,36
Oligodendrocytes	PHLPP1	ENSG00000081913	0,93	1,531048738	0,86	0,98	0,61
Oligodendrocytes	C1QTNF3-AMACR	ENSG00000273294	0,741	0,821489233	0,482	0,7	0,33
Oligodendrocytes	PKP4	ENSG00000144283	0,834	1,090120238	0,668	0,91	0,55
Oligodendrocytes	SORT1	ENSG00000134243	0,862	1,064848854	0,724	0,94	0,58
Oligodendrocytes	RAB30	ENSG00000137502	0,77	0,938148927	0,54	0,75	0,39
Oligodendrocytes	TARSL2	ENSG00000185418	0,743	0,748215552	0,486	0,72	0,35

Oligodendrocytes	KNOP1	ENSG00000103550	0,707	0,702670275	0,414	0,58	0,22
Oligodendrocytes	BRMS1L	ENSG00000100916	0,716	0,754772285	0,432	0,57	0,2
Oligodendrocytes	NALCN	ENSG00000102452	0,754	0,698176765	0,508	0,88	0,52
Oligodendrocytes	STMN4	ENSG00000015592	0,707	0,828192207	0,414	0,51	0,15
Oligodendrocytes	DNAJB2	ENSG00000135924	0,728	0,732258275	0,456	0,66	0,3
Oligodendrocytes	REPS2	ENSG00000169891	0,727	0,777220189	0,454	0,66	0,29
Oligodendrocytes	DLC1	ENSG00000164741	0,817	0,700290791	0,634	0,9	0,54
Oligodendrocytes	SLC25A13	ENSG00000004864	0,801	1,019273897	0,602	0,82	0,46
Oligodendrocytes	MTUS1	ENSG00000129422	0,805	0,889200899	0,61	0,89	0,53
Oligodendrocytes	ANKRD18A	ENSG00000180071	0,705	0,948339672	0,41	0,5	0,14
Oligodendrocytes	LRRC8B	ENSG00000197147	0,705	0,657746604	0,41	0,58	0,22
Oligodendrocytes	BACE1	ENSG00000186318	0,72	0,752364487	0,44	0,59	0,23
Oligodendrocytes	SH3D19	ENSG00000109686	0,74	0,734989235	0,48	0,81	0,45
Oligodendrocytes	SVIP	ENSG00000198168	0,706	0,730017576	0,412	0,53	0,18
Oligodendrocytes	JAKMIP3	ENSG00000188385	0,723	0,835176881	0,446	0,58	0,23
Oligodendrocytes	STK39	ENSG00000198648	0,762	0,757694069	0,524	0,81	0,45
Oligodendrocytes	TSPAN5	ENSG00000168785	0,761	0,802742604	0,522	0,83	0,47
Oligodendrocytes	OSBPL1A	ENSG00000141447	0,817	0,948271314	0,634	0,89	0,54
Oligodendrocytes	HBS1L	ENSG00000112339	0,726	0,729957172	0,452	0,66	0,31
Oligodendrocytes	NCAM2	ENSG00000154654	0,77	0,816219315	0,54	0,98	0,64
Oligodendrocytes	ALCAM	ENSG00000170017	0,762	0,703980075	0,524	0,93	0,58
Oligodendrocytes	FRYL	ENSG00000075539	0,942	1,489876748	0,884	0,98	0,64
Oligodendrocytes	LMCD1-AS1	ENSG00000227110	0,739	0,887871375	0,478	0,78	0,43
Oligodendrocytes	GNG7	ENSG00000176533	0,766	0,888155617	0,532	0,82	0,47
Oligodendrocytes	LHPP	ENSG00000107902	0,707	0,698179587	0,414	0,58	0,23
Oligodendrocytes	ABHD17B	ENSG00000107362	0,703	0,699726269	0,406	0,56	0,22
Oligodendrocytes	TBC1D12	ENSG00000108239	0,734	0,774706084	0,468	0,72	0,38
Oligodendrocytes	NCAM1	ENSG00000149294	0,846	0,886840911	0,692	0,99	0,65
Oligodendrocytes	RCAN2	ENSG00000172348	0,701	0,642622746	0,402	0,69	0,34
Oligodendrocytes	SLC13A3	ENSG00000158296	0,712	0,724011854	0,424	0,63	0,28
Oligodendrocytes	RASGRF1	ENSG00000058335	0,715	1,002505646	0,43	0,57	0,22
Oligodendrocytes	SNX30	ENSG00000148158	0,702	0,628214183	0,404	0,61	0,26
Oligodendrocytes	SEPTIN7	ENSG00000122545	0,904	1,30400155	0,808	0,96	0,62
Oligodendrocytes	CLIP4	ENSG00000115295	0,746	0,744179586	0,492	0,77	0,43
Oligodendrocytes	CDC14B	ENSG00000081377	0,758	0,774915599	0,516	0,82	0,48
Oligodendrocytes	CUEDC1	ENSG00000180891	0,703	0,635838361	0,406	0,64	0,3
Oligodendrocytes	AMD1	ENSG00000123505	0,709	0,681085651	0,418	0,63	0,3
Oligodendrocytes	PPP2R2B	ENSG00000156475	0,834	1,066980836	0,668	0,98	0,65
Oligodendrocytes	KCTD8	ENSG00000183783	0,714	0,74630307	0,428	0,73	0,4
Oligodendrocytes	MICAL3	ENSG00000243156	0,737	0,683403651	0,474	0,81	0,49
Oligodendrocytes	PPP1R21	ENSG00000162869	0,716	0,644079612	0,432	0,71	0,38
Oligodendrocytes	LINC01505	ENSG00000234323	0,703	1,355969048	0,406	0,57	0,25
Oligodendrocytes	WDR20	ENSG00000140153	0,721	0,668190704	0,442	0,7	0,38
Oligodendrocytes	DIP2C	ENSG00000151240	0,849	0,976274771	0,698	0,96	0,64
Oligodendrocytes	ZNF652	ENSG00000198740	0,772	0,853704121	0,544	0,82	0,5
Oligodendrocytes	HIPK2	ENSG00000064393	0,794	0,849679266	0,588	0,9	0,59
Oligodendrocytes	NTM	ENSG00000182667	0,738	0,651586708	0,476	0,96	0,65
Oligodendrocytes	TULP4	ENSG00000130338	0,771	0,798632265	0,542	0,88	0,57
Oligodendrocytes	DLG1	ENSG00000075711	0,926	1,407204503	0,852	0,97	0,66
Oligodendrocytes	CPQ	ENSG00000104324	0,71	0,649420106	0,42	0,78	0,48
Oligodendrocytes	DICER1	ENSG00000100697	0,779	0,763579468	0,558	0,85	0,54
Oligodendrocytes	AOPEP	ENSG00000148120	0,791	0,808913079	0,582	0,91	0,6
Oligodendrocytes	PTBP2	ENSG00000117569	0,795	0,838513672	0,59	0,89	0,59
Oligodendrocytes	TLE4	ENSG00000106829	0,72	0,731285247	0,44	0,75	0,45
Oligodendrocytes	ZFYVE16	ENSG00000039319	0,784	0,875845366	0,568	0,84	0,53

Oligodendrocytes	MAP4K5	ENSG00000012983	0,853	1,042664131	0,706	0,93	0,63
Oligodendrocytes	RNF13	ENSG00000082996	0,781	0,765764039	0,562	0,86	0,56
Oligodendrocytes	SAMD4B	ENSG00000179134	0,709	0,625239339	0,418	0,69	0,39
Oligodendrocytes	SCARB2	ENSG00000138760	0,726	0,651662939	0,452	0,75	0,45
Oligodendrocytes	DIP2B	ENSG00000066084	0,889	1,117085671	0,778	0,97	0,68
Oligodendrocytes	MYO6	ENSG00000196586	0,755	0,680106694	0,51	0,86	0,57
Oligodendrocytes	NLK	ENSG00000087095	0,715	0,585190818	0,43	0,79	0,51
Oligodendrocytes	RDX	ENSG00000137710	0,726	0,684629385	0,452	0,79	0,51
Oligodendrocytes	AGAP1	ENSG00000157985	0,854	0,915601403	0,708	0,99	0,71
Oligodendrocytes	ANKIB1	ENSG00000001629	0,772	0,746064392	0,544	0,89	0,61
Oligodendrocytes	RBPJ	ENSG00000168214	0,743	0,632309062	0,486	0,85	0,57
Oligodendrocytes	ITCH	ENSG00000078747	0,738	0,627380579	0,476	0,82	0,55
Oligodendrocytes	FAM171A1	ENSG00000148468	0,706	0,605276798	0,412	0,74	0,47
Oligodendrocytes	MAPRE2	ENSG00000166974	0,746	0,789525876	0,492	0,8	0,53
Oligodendrocytes	DLG2	ENSG00000150672	0,865	0,947550418	0,73	1	0,73
Oligodendrocytes	MBNL2	ENSG00000139793	0,846	0,900099393	0,692	0,95	0,68
Oligodendrocytes	PCDH9	ENSG00000184226	0,909	1,252405655	0,818	1	0,73
Oligodendrocytes	PDE4B	ENSG00000184588	0,933	1,416483973	0,866	0,99	0,73
Oligodendrocytes	PEBP1	ENSG00000089220	0,714	0,691660185	0,428	0,75	0,49
Oligodendrocytes	USP32	ENSG00000170832	0,717	0,642879223	0,434	0,79	0,53
Oligodendrocytes	ERBIN	ENSG00000112851	0,919	1,452307659	0,838	0,99	0,73
Oligodendrocytes	PPP1R12B	ENSG00000077157	0,752	0,644077208	0,504	0,87	0,61
Oligodendrocytes	UBE4B	ENSG00000130939	0,713	0,588049146	0,426	0,8	0,54
Oligodendrocytes	ARHGAP21	ENSG00000107863	0,884	1,069305358	0,768	0,97	0,71
Oligodendrocytes	ZKSCAN1	ENSG00000106261	0,714	0,606848265	0,428	0,77	0,51
Oligodendrocytes	FAR1	ENSG00000197601	0,727	0,647424041	0,454	0,77	0,52
Oligodendrocytes	APP	ENSG00000142192	0,751	0,599609948	0,502	0,94	0,69
Oligodendrocytes	MAGI2	ENSG00000187391	0,854	0,869720437	0,708	1	0,75
Oligodendrocytes	TJP1	ENSG00000104067	0,817	0,812279	0,634	0,96	0,72
Oligodendrocytes	CCDC88A	ENSG00000115355	0,837	0,845714661	0,674	0,96	0,73
Oligodendrocytes	RNF130	ENSG00000113269	0,758	0,628888482	0,516	0,91	0,69
Oligodendrocytes	RTN4	ENSG00000115310	0,902	1,093217859	0,804	0,98	0,75
Oligodendrocytes	CLASP2	ENSG00000163539	0,903	1,013594326	0,806	0,99	0,77
Oligodendrocytes	MAP4K4	ENSG00000071054	0,897	1,096173591	0,794	0,98	0,76
Oligodendrocytes	FMNL2	ENSG00000157827	0,915	1,311774904	0,83	0,99	0,8
Oligodendrocytes	FTH1	ENSG00000167996	0,725	0,776894145	0,45	0,85	0,66
Oligodendrocytes	PTK2	ENSG00000169398	0,756	0,639409331	0,512	0,96	0,8
Oligodendrocytes	SIK3	ENSG00000160584	0,881	1,11310599	0,762	0,99	0,83
Oligodendrocytes	ZNF638	ENSG00000075292	0,767	0,628759385	0,534	0,95	0,8
Oligodendrocytes	DST	ENSG00000151914	0,801	0,612109476	0,602	0,99	0,86
Oligodendrocytes	TCF12	ENSG00000140262	0,801	0,743308943	0,602	0,98	0,85
Oligodendrocytes	QKI	ENSG00000112531	0,948	1,29071784	0,896	1	0,88
Excitatory	MYT1L	ENSG00000186487	0,949	1,836078745	0,898	0,97	0,12
Excitatory	CELF4	ENSG00000101489	0,935	1,528801414	0,87	0,94	0,09
Excitatory	GRIN1	ENSG00000176884	0,925	1,291753069	0,85	0,92	0,08
Excitatory	SYT1	ENSG00000067715	0,938	2,003479071	0,876	0,96	0,12
Excitatory	PAK3	ENSG00000077264	0,93	1,532454893	0,86	0,94	0,1
Excitatory	ANKRD30BL	ENSG00000163046	0,943	1,68275509	0,886	0,95	0,12
Excitatory	SRRM3	ENSG00000177679	0,922	1,30050477	0,844	0,92	0,09
Excitatory	GRIN2B	ENSG00000273079	0,921	1,480829437	0,842	0,92	0,1
Excitatory	CACNA1B	ENSG00000148408	0,939	1,69432504	0,878	0,94	0,13
Excitatory	RIMS2	ENSG00000176406	0,952	2,108544744	0,904	0,97	0,16
Excitatory	LINGO2	ENSG00000174482	0,911	2,030034091	0,822	0,88	0,08
Excitatory	SYN2	ENSG00000157152	0,907	1,279209628	0,814	0,91	0,1
Excitatory	GABBR2	ENSG00000136928	0,904	1,238230212	0,808	0,89	0,08

Excitatory	ATP8A2	ENSG00000132932	0,934	1,697861983	0,868	0,95	0,15
Excitatory	SLC4A10	ENSG00000144290	0,908	1,313190073	0,816	0,9	0,1
Excitatory	CDH18	ENSG00000145526	0,929	2,194823648	0,858	0,92	0,13
Excitatory	GABRB3	ENSG00000166206	0,918	1,445003669	0,836	0,93	0,14
Excitatory	STMN2	ENSG00000104435	0,896	1,226738928	0,792	0,85	0,06
Excitatory	TENM2	ENSG00000145934	0,935	2,415074547	0,87	0,94	0,15
Excitatory	CELF5	ENSG00000161082	0,895	1,01373918	0,79	0,86	0,08
Excitatory	KCNB2	ENSG00000182674	0,9	1,896723618	0,8	0,83	0,05
Excitatory	FGF12	ENSG00000114279	0,924	1,713846524	0,848	0,97	0,18
Excitatory	SCN2A	ENSG00000136531	0,917	1,298722359	0,834	0,93	0,15
Excitatory	KSR2	ENSG00000171435	0,908	1,323234624	0,816	0,91	0,13
Excitatory	RAB3C	ENSG00000152932	0,897	1,292268148	0,794	0,89	0,11
Excitatory	MEG8	ENSG00000225746	0,901	1,38890458	0,802	0,89	0,11
Excitatory	LRFN5	ENSG00000165379	0,915	1,667067659	0,83	0,92	0,14
Excitatory	CHD5	ENSG00000116254	0,89	0,917338143	0,78	0,83	0,06
Excitatory	HECW1	ENSG00000002746	0,894	1,421957646	0,788	0,89	0,11
Excitatory	CAMK2B	ENSG00000058404	0,892	1,027051732	0,784	0,9	0,13
Excitatory	PTPRN2	ENSG00000155093	0,939	1,695309514	0,878	0,95	0,18
Excitatory	MIAT	ENSG00000225783	0,894	1,129147242	0,788	0,87	0,1
Excitatory	CADPS	ENSG00000163618	0,924	1,528831659	0,848	0,96	0,2
Excitatory	KCNC2	ENSG00000166006	0,888	1,488853556	0,776	0,81	0,05
Excitatory	NMNAT2	ENSG00000157064	0,891	1,128094288	0,782	0,88	0,12
Excitatory	DNM1	ENSG00000106976	0,892	1,029215195	0,784	0,89	0,13
Excitatory	OPCML	ENSG00000183715	0,872	1,214176777	0,744	0,94	0,18
Excitatory	NRG1	ENSG00000157168	0,898	2,343329129	0,796	0,86	0,1
Excitatory	XKR4	ENSG00000206579	0,884	1,34563042	0,768	0,9	0,14
Excitatory	DLGAP2	ENSG00000198010	0,89	1,51240628	0,78	0,86	0,1
Excitatory	RALYL	ENSG00000184672	0,937	2,116987122	0,874	0,97	0,21
Excitatory	SCN3A	ENSG00000153253	0,884	1,135297746	0,768	0,88	0,12
Excitatory	GABRB2	ENSG00000145864	0,883	1,436990823	0,766	0,82	0,07
Excitatory	GABRG2	ENSG00000113327	0,881	1,020336251	0,762	0,81	0,06
Excitatory	SHANK2	ENSG00000162105	0,879	1,044400357	0,758	0,88	0,13
Excitatory	ARHGAP44	ENSG00000006740	0,899	1,190827121	0,798	0,9	0,15
Excitatory	CACNA1E	ENSG00000198216	0,894	1,447986247	0,788	0,86	0,11
Excitatory	GRM7	ENSG00000196277	0,905	1,714651557	0,81	0,93	0,18
Excitatory	SCN1A	ENSG00000144285	0,876	1,141052593	0,752	0,91	0,16
Excitatory	WNK2	ENSG00000165238	0,876	0,973004115	0,752	0,88	0,13
Excitatory	UNC80	ENSG00000144406	0,88	1,064584128	0,76	0,93	0,18
Excitatory	GRM5	ENSG00000168959	0,886	1,458644988	0,772	0,91	0,16
Excitatory	SYN3	ENSG00000185666	0,883	1,394021221	0,766	0,85	0,11
Excitatory	GRIA1	ENSG00000155511	0,881	1,570289931	0,762	0,83	0,09
Excitatory	SNAP25	ENSG00000132639	0,892	1,211348459	0,784	0,93	0,19
Excitatory	GALNT17	ENSG00000185274	0,876	1,174919952	0,752	0,85	0,1
Excitatory	SEZ6L	ENSG00000100095	0,875	1,265310919	0,75	0,87	0,13
Excitatory	ROBO2	ENSG00000185008	0,908	2,165191603	0,816	0,92	0,17
Excitatory	MTUS2	ENSG00000132938	0,879	1,593867698	0,758	0,82	0,08
Excitatory	DNAH14	ENSG00000185842	0,886	1,279983337	0,772	0,85	0,12
Excitatory	EPHA6	ENSG00000080224	0,879	1,792841387	0,758	0,82	0,08
Excitatory	FGF14	ENSG00000102466	0,899	1,467022799	0,798	0,98	0,24
Excitatory	DCLK1	ENSG00000133083	0,886	1,148501274	0,772	0,94	0,2
Excitatory	KCNH7	ENSG00000184611	0,881	1,789290423	0,762	0,81	0,07
Excitatory	JPH4	ENSG00000092051	0,867	0,83685635	0,734	0,81	0,08
Excitatory	SUSD4	ENSG00000143502	0,87	0,952958051	0,74	0,82	0,09
Excitatory	MIR137HG	ENSG00000225206	0,875	1,361739271	0,75	0,79	0,06
Excitatory	AGBL4	ENSG00000186094	0,922	1,668228178	0,844	0,95	0,22

Excitatory	SYT16	ENSG00000139973	0,87	1,018537288	0,74	0,85	0,12
Excitatory	DOK6	ENSG00000206052	0,868	1,163711512	0,736	0,86	0,13
Excitatory	GRIP1	ENSG00000155974	0,908	1,639839567	0,816	0,92	0,19
Excitatory	AMPH	ENSG00000078053	0,871	1,033369364	0,742	0,84	0,11
Excitatory	RUNDC3B	ENSG00000105784	0,863	0,927942945	0,726	0,84	0,11
Excitatory	STXBP5L	ENSG00000145087	0,897	1,454810751	0,794	0,92	0,19
Excitatory	HS6ST3	ENSG00000185352	0,881	1,679383108	0,762	0,88	0,16
Excitatory	MAST1	ENSG00000105613	0,862	0,81628926	0,724	0,8	0,07
Excitatory	SPTBN4	ENSG00000160460	0,903	1,220484949	0,806	0,93	0,2
Excitatory	PCSK2	ENSG00000125851	0,872	1,409402138	0,744	0,8	0,07
Excitatory	CALY	ENSG00000130643	0,863	1,172618244	0,726	0,8	0,08
Excitatory	TENM3	ENSG00000218336	0,885	1,473352618	0,77	0,89	0,17
Excitatory	SRRM4	ENSG00000139767	0,859	0,976623481	0,718	0,78	0,06
Excitatory	GALNTL6	ENSG00000174473	0,872	1,967340051	0,744	0,82	0,1
Excitatory	PRKAR1B	ENSG00000188191	0,872	0,94370321	0,744	0,85	0,13
Excitatory	NELL2	ENSG00000184613	0,871	1,359528813	0,742	0,82	0,1
Excitatory	SNTG1	ENSG00000147481	0,867	1,656986759	0,734	0,87	0,15
Excitatory	KCNJ3	ENSG00000162989	0,861	1,129524508	0,722	0,83	0,11
Excitatory	UNC5D	ENSG00000156687	0,868	1,894736163	0,736	0,81	0,1
Excitatory	SYBU	ENSG00000147642	0,854	0,887320735	0,708	0,85	0,14
Excitatory	CCSER1	ENSG00000184305	0,913	1,632873222	0,826	0,96	0,25
Excitatory	TMEM130	ENSG00000166448	0,854	0,822758753	0,708	0,77	0,06
Excitatory	SORBS2	ENSG00000154556	0,859	1,035307352	0,718	0,9	0,2
Excitatory	PRICKLE1	ENSG00000139174	0,852	1,023419127	0,704	0,79	0,09
Excitatory	CACNA1A	ENSG00000141837	0,87	1,135196683	0,74	0,94	0,24
Excitatory	FRRS1L	ENSG00000260230	0,864	0,905900448	0,728	0,85	0,14
Excitatory	PLXNA4	ENSG00000221866	0,861	1,106433386	0,722	0,82	0,11
Excitatory	TUSC3	ENSG00000104723	0,851	0,845664185	0,702	0,81	0,1
Excitatory	SCN8A	ENSG00000196876	0,875	1,042393312	0,75	0,88	0,18
Excitatory	IQSEC3	ENSG00000120645	0,849	0,75312042	0,698	0,76	0,06
Excitatory	CNTN5	ENSG00000149972	0,872	2,307335272	0,744	0,81	0,1
Excitatory	BASP1	ENSG00000176788	0,857	0,953951943	0,714	0,88	0,17
Excitatory	GAP43	ENSG00000172020	0,854	0,946470331	0,708	0,78	0,08
Excitatory	GRIK2	ENSG00000164418	0,908	1,672820045	0,816	0,96	0,26
Excitatory	FRMPD4	ENSG00000169933	0,857	1,429255679	0,714	0,83	0,13
Excitatory	PTPRR	ENSG00000153233	0,852	1,069248841	0,704	0,75	0,05
Excitatory	ADD2	ENSG00000075340	0,849	0,763813784	0,698	0,78	0,08
Excitatory	KIAA1549L	ENSG00000110427	0,848	0,93457727	0,696	0,8	0,1
Excitatory	GABRG3	ENSG00000182256	0,852	1,570330139	0,704	0,75	0,05
Excitatory	ATRNL1	ENSG00000107518	0,838	0,937164967	0,676	0,87	0,18
Excitatory	ADAM23	ENSG00000114948	0,866	1,045191576	0,732	0,87	0,18
Excitatory	RIMS1	ENSG00000079841	0,91	1,500775303	0,82	0,93	0,24
Excitatory	KLHL29	ENSG00000119771	0,849	0,95856309	0,698	0,8	0,11
Excitatory	CNTNAP5	ENSG00000155052	0,859	1,628956776	0,718	0,84	0,15
Excitatory	SHISA9	ENSG00000237515	0,849	1,242819669	0,698	0,83	0,13
Excitatory	CACNB2	ENSG00000165995	0,877	1,238990006	0,754	0,94	0,24
Excitatory	GABRB1	ENSG00000163288	0,874	1,279647708	0,748	0,91	0,22
Excitatory	FHOD3	ENSG00000134775	0,851	1,010443778	0,702	0,81	0,11
Excitatory	FAM155A	ENSG00000204442	0,931	1,827240861	0,862	0,98	0,29
Excitatory	OLFM3	ENSG00000118733	0,855	1,296724951	0,71	0,79	0,1
Excitatory	KHDRBS2	ENSG00000112232	0,877	1,379594484	0,754	0,89	0,2
Excitatory	SLC8A1	ENSG00000183023	0,843	1,006918401	0,686	0,95	0,26
Excitatory	CAP2	ENSG00000112186	0,842	0,769229262	0,684	0,78	0,09
Excitatory	STXBP5-AS1	ENSG00000233452	0,856	1,228167931	0,712	0,81	0,12
Excitatory	REEP1	ENSG00000068615	0,843	0,810928791	0,686	0,78	0,1

Excitatory	NSG2	ENSG00000170091	0,837	0,818427178	0,674	0,78	0,1
Excitatory	ATCAY	ENSG00000167654	0,834	0,694109058	0,668	0,78	0,1
Excitatory	VSNL1	ENSG00000163032	0,842	0,956971125	0,684	0,75	0,06
Excitatory	RIMBP2	ENSG00000060709	0,846	0,815682256	0,692	0,8	0,11
Excitatory	SNRPN	ENSG00000128739	0,898	1,45028026	0,796	0,91	0,22
Excitatory	NEXMIF	ENSG00000050030	0,839	0,912110641	0,678	0,79	0,1
Excitatory	DSCAM	ENSG00000171587	0,844	0,700617852	0,688	0,95	0,27
Excitatory	CNTN4	ENSG00000144619	0,853	1,63310446	0,706	0,82	0,14
Excitatory	SLC2A13	ENSG00000151229	0,853	1,140690754	0,706	0,88	0,2
Excitatory	OSBPL6	ENSG00000079156	0,837	0,876932624	0,674	0,88	0,2
Excitatory	BICDL1	ENSG00000135127	0,843	0,798265564	0,686	0,77	0,1
Excitatory	ELAVL2	ENSG00000107105	0,838	0,971176942	0,676	0,73	0,05
Excitatory	DLGAP1	ENSG00000170579	0,867	1,342557138	0,734	0,92	0,25
Excitatory	NOL4	ENSG00000101746	0,826	0,822378014	0,652	0,85	0,18
Excitatory	PPM1E	ENSG00000175175	0,869	1,137385809	0,738	0,91	0,24
Excitatory	KCND2	ENSG00000184408	0,835	1,053742766	0,67	0,92	0,25
Excitatory	SLC41A2	ENSG00000136052	0,833	0,736811469	0,666	0,79	0,12
Excitatory	C11orf80	ENSG00000173715	0,839	0,797455118	0,678	0,79	0,12
Excitatory	SORCS3	ENSG00000156395	0,834	1,131425557	0,668	0,78	0,11
Excitatory	RAP1GAP2	ENSG00000132359	0,826	0,704166669	0,652	0,79	0,13
Excitatory	KCNIP4	ENSG00000185774	0,87	1,662495086	0,74	0,91	0,25
Excitatory	ZNF804A	ENSG00000170396	0,833	1,278761936	0,666	0,77	0,1
Excitatory	PLPPR4	ENSG00000117600	0,83	0,733983102	0,66	0,75	0,08
Excitatory	MYH10	ENSG00000133026	0,833	0,78160054	0,666	0,83	0,16
Excitatory	ELAVL4	ENSG00000162374	0,828	0,774086371	0,656	0,75	0,09
Excitatory	LRRC7	ENSG00000033122	0,885	1,562509689	0,77	0,94	0,27
Excitatory	CNTN1	ENSG00000018236	0,82	0,836436071	0,64	0,96	0,3
Excitatory	ANKRD34C-AS1	ENSG00000259234	0,83	0,861308332	0,66	0,7	0,04
Excitatory	PAM	ENSG00000145730	0,849	1,093142325	0,698	0,9	0,24
Excitatory	EPB41L4B	ENSG00000095203	0,832	0,812998394	0,664	0,75	0,09
Excitatory	SLC8A3	ENSG00000100678	0,824	0,78096972	0,648	0,77	0,11
Excitatory	TMEM132B	ENSG00000139364	0,83	0,95646704	0,66	0,79	0,13
Excitatory	KIAA0319	ENSG00000137261	0,823	0,74534728	0,646	0,77	0,11
Excitatory	RPH3A	ENSG00000089169	0,83	1,007261376	0,66	0,72	0,06
Excitatory	EFNA5	ENSG00000184349	0,827	1,093537905	0,654	0,78	0,12
Excitatory	CCDC85A	ENSG00000055813	0,823	0,916644115	0,646	0,77	0,12
Excitatory	CA10	ENSG00000154975	0,811	0,883974304	0,622	0,78	0,12
Excitatory	SLC24A3	ENSG00000185052	0,821	1,013047169	0,642	0,78	0,12
Excitatory	CHL1	ENSG00000134121	0,81	0,723794249	0,62	0,87	0,22
Excitatory	MDGA2	ENSG00000139915	0,874	1,332851979	0,748	0,96	0,31
Excitatory	AC073050.1	ENSG00000228222	0,833	1,1309326	0,666	0,8	0,15
Excitatory	ASIC2	ENSG00000108684	0,836	1,470915231	0,672	0,76	0,11
Excitatory	SLC17A6	ENSG00000091664	0,826	0,893899526	0,652	0,66	0,01
Excitatory	ANO5	ENSG00000171714	0,819	0,683252033	0,638	0,77	0,12
Excitatory	CAMK2A	ENSG00000070808	0,829	0,787996719	0,658	0,73	0,07
Excitatory	ATP2B2	ENSG00000157087	0,808	0,697245836	0,616	0,84	0,19
Excitatory	FGF13	ENSG00000129682	0,829	1,377968328	0,658	0,7	0,05
Excitatory	BASP1-AS1	ENSG00000215196	0,824	0,825243582	0,648	0,73	0,08
Excitatory	GUCY1A2	ENSG00000152402	0,826	0,968680079	0,652	0,81	0,17
Excitatory	LHFPL3	ENSG00000187416	0,82	0,819534017	0,64	0,83	0,18
Excitatory	LHFPL4	ENSG00000156959	0,82	0,633440714	0,64	0,75	0,1
Excitatory	LRRTM4	ENSG00000176204	0,862	1,528318444	0,724	0,91	0,26
Excitatory	MGAT4C	ENSG00000182050	0,839	1,61907205	0,678	0,81	0,16
Excitatory	CNKSR2	ENSG00000149970	0,822	0,953792643	0,644	0,8	0,15

Excitatory	DGKB	ENSG00000136267	0,838	1,373394197	0,676	0,82	0,17
Excitatory	CACNA1C	ENSG00000151067	0,895	1,386221178	0,79	0,96	0,31
Excitatory	NHS	ENSG00000188158	0,819	0,8770928	0,638	0,77	0,12
Excitatory	JAKMIP1	ENSG00000152969	0,819	0,699214629	0,638	0,72	0,07
Excitatory	SNAP91	ENSG00000065609	0,885	1,156963894	0,77	0,94	0,29
Excitatory	FAM189A1	ENSG00000104059	0,825	0,999919421	0,65	0,72	0,08
Excitatory	DENND1B	ENSG00000213047	0,817	0,727840433	0,634	0,78	0,14
Excitatory	KCNB1	ENSG00000158445	0,815	0,647498744	0,63	0,75	0,11
Excitatory	AC092683.1	ENSG00000230606	0,903	1,312822413	0,806	0,94	0,29
Excitatory	PRR16	ENSG00000184838	0,821	1,17195109	0,642	0,71	0,07
Excitatory	ZFR2	ENSG00000105278	0,82	0,654272762	0,64	0,73	0,09
Excitatory	CSMD3	ENSG00000164796	0,883	1,414820943	0,766	0,95	0,31
Excitatory	KCTD16	ENSG00000183775	0,836	1,208624592	0,672	0,82	0,18
Excitatory	EPHA5	ENSG00000145242	0,821	1,083273597	0,642	0,71	0,07
Excitatory	SLC44A5	ENSG00000137968	0,823	1,144364276	0,646	0,75	0,12
Excitatory	PTPRN	ENSG00000054356	0,818	0,678477827	0,636	0,69	0,05
Excitatory	CACNG2	ENSG00000166862	0,817	0,764329389	0,634	0,71	0,08
Excitatory	DPY19L2	ENSG00000177990	0,812	0,672751812	0,624	0,77	0,13
Excitatory	KCNQ3	ENSG00000184156	0,819	0,783981609	0,638	0,88	0,25
Excitatory	KCNJ6	ENSG00000157542	0,818	0,877572017	0,636	0,71	0,07
Excitatory	TENM1	ENSG00000009694	0,823	1,102615228	0,646	0,74	0,11
Excitatory	ABLIM2	ENSG00000163995	0,824	0,779732725	0,648	0,8	0,17
Excitatory	PCDH15	ENSG00000150275	0,816	1,322109856	0,632	0,79	0,16
Excitatory	RBFOX3	ENSG00000167281	0,817	0,836916621	0,634	0,69	0,06
Excitatory	PCLO	ENSG00000186472	0,912	1,364846243	0,824	0,96	0,33
Excitatory	CIT	ENSG00000122966	0,825	0,910154873	0,65	0,81	0,17
Excitatory	SIDT1	ENSG00000072858	0,812	0,699453418	0,624	0,71	0,08
Excitatory	PLCB4	ENSG00000101333	0,81	0,853859756	0,62	0,84	0,21
Excitatory	ANK1	ENSG00000029534	0,818	0,977515239	0,636	0,7	0,07
Excitatory	CLSTN3	ENSG00000139182	0,811	0,598520179	0,622	0,7	0,07
Excitatory	NYAP2	ENSG00000144460	0,812	0,722761919	0,624	0,68	0,05
Excitatory	BTBD11	ENSG00000151136	0,807	0,769904445	0,614	0,72	0,09
Excitatory	SCN9A	ENSG00000169432	0,816	0,933997796	0,632	0,74	0,11
Excitatory	SH3GL2	ENSG00000107295	0,809	0,776771703	0,618	0,76	0,13
Excitatory	MAP7D2	ENSG00000184368	0,812	0,612354704	0,624	0,69	0,06
Excitatory	SLC35F1	ENSG00000196376	0,803	0,7214571	0,606	0,82	0,2
Excitatory	SCN7A	ENSG00000136546	0,813	0,768899758	0,626	0,68	0,05
Excitatory	FP700111.1	ENSG00000224363	0,835	0,887797684	0,67	0,86	0,23
Excitatory	CSMD2	ENSG00000121904	0,82	0,902853503	0,64	0,86	0,23
Excitatory	PAK5	ENSG00000101349	0,807	0,690005836	0,614	0,72	0,1
Excitatory	GNB5	ENSG00000069966	0,806	0,588016683	0,612	0,74	0,11
Excitatory	RNF175	ENSG00000145428	0,811	0,641274999	0,622	0,69	0,06
Excitatory	CAMK4	ENSG00000152495	0,811	0,836164933	0,622	0,73	0,1
Excitatory	CNNM1	ENSG00000119946	0,809	0,598975289	0,618	0,69	0,06
Excitatory	NRG3	ENSG00000185737	0,847	1,091130079	0,694	0,98	0,35
Excitatory	NRSN1	ENSG00000152954	0,808	0,627838611	0,616	0,69	0,07
Excitatory	NEK10	ENSG00000163491	0,808	0,726295483	0,616	0,74	0,12
Excitatory	TMTC1	ENSG00000133687	0,804	0,809569524	0,608	0,82	0,2
Excitatory	CACNA1D	ENSG00000157388	0,823	0,787050455	0,646	0,87	0,25
Excitatory	GABRA2	ENSG00000151834	0,811	0,858549639	0,622	0,7	0,08
Excitatory	AKAP12	ENSG00000131016	0,798	0,599141329	0,596	0,77	0,14
Excitatory	RAD9A	ENSG00000172613	0,808	0,672622797	0,616	0,82	0,2
Excitatory	STXBP1	ENSG00000136854	0,836	0,800427893	0,672	0,88	0,27
Excitatory	GRM1	ENSG00000152822	0,812	1,130297904	0,624	0,68	0,06
Excitatory	PNMA2	ENSG00000240694	0,806	0,737984639	0,612	0,7	0,08

Excitatory	CLVS1	ENSG00000177182	0,803	0,659677241	0,606	0,76	0,15
Excitatory	KIAA1211	ENSG00000109265	0,807	0,76748139	0,614	0,81	0,19
Excitatory	MAP2	ENSG00000078018	0,916	1,374052122	0,832	0,98	0,37
Excitatory	SVOP	ENSG00000166111	0,809	0,632237755	0,618	0,66	0,05
Excitatory	ELMOD1	ENSG00000110675	0,799	0,718535119	0,598	0,74	0,12
Excitatory	GABRA1	ENSG00000022355	0,808	0,833291803	0,616	0,66	0,04
Excitatory	RGS17	ENSG00000091844	0,805	0,675179585	0,61	0,69	0,07
Excitatory	ERC2	ENSG00000187672	0,859	1,130196124	0,718	0,93	0,32
Excitatory	SV2A	ENSG00000159164	0,802	0,663787494	0,604	0,76	0,15
Excitatory	LINC01250	ENSG00000234423	0,808	0,781233333	0,616	0,66	0,05
Excitatory	CERS6	ENSG00000172292	0,809	0,788253946	0,618	0,86	0,25
Excitatory	MAP6	ENSG00000171533	0,804	0,604492318	0,608	0,77	0,16
Excitatory	SYT14	ENSG00000143469	0,818	0,776569162	0,636	0,86	0,25
Excitatory	SGCZ	ENSG00000185053	0,819	1,884327589	0,638	0,76	0,15
Excitatory	LINC01122	ENSG00000233723	0,808	0,960402425	0,616	0,78	0,17
Excitatory	LRFN2	ENSG00000156564	0,796	0,647541801	0,592	0,69	0,09
Excitatory	BRINP3	ENSG00000162670	0,803	1,096082874	0,606	0,78	0,17
Excitatory	RALGPS2	ENSG00000116191	0,791	0,636296593	0,582	0,76	0,16
Excitatory	PACRG	ENSG00000112530	0,803	0,774222071	0,606	0,8	0,2
Excitatory	HCN1	ENSG00000164588	0,803	1,188319502	0,606	0,66	0,06
Excitatory	FBXO16	ENSG00000214050	0,797	0,607220275	0,594	0,69	0,09
Excitatory	ADGRL2	ENSG00000117114	0,805	1,191564701	0,61	0,72	0,11
Excitatory	RAPGEF4	ENSG00000091428	0,816	0,775917868	0,632	0,91	0,31
Excitatory	PCDH7	ENSG00000169851	0,812	1,165839958	0,624	0,85	0,25
Excitatory	TMEM108	ENSG00000144868	0,81	0,993381197	0,62	0,84	0,24
Excitatory	KIF5A	ENSG00000155980	0,798	0,594320767	0,596	0,7	0,1
Excitatory	ASTN1	ENSG00000152092	0,804	0,74538418	0,608	0,85	0,25
Excitatory	RYR2	ENSG00000198626	0,852	1,526882085	0,704	0,85	0,26
Excitatory	VAT1L	ENSG00000171724	0,8	0,78347056	0,6	0,72	0,12
Excitatory	ME3	ENSG00000151376	0,792	0,623551302	0,584	0,77	0,17
Excitatory	TAFA2	ENSG00000198673	0,814	1,291586578	0,628	0,78	0,18
Excitatory	CSMD1	ENSG00000183117	0,877	1,378165766	0,754	0,96	0,36
Excitatory	LONRF2	ENSG00000170500	0,806	0,68797031	0,612	0,82	0,23
Excitatory	SYN1	ENSG00000008056	0,796	0,596585291	0,592	0,66	0,07
Excitatory	AFF3	ENSG00000144218	0,852	1,018109051	0,704	0,94	0,34
Excitatory	THY1	ENSG00000154096	0,794	0,760651957	0,588	0,68	0,09
Excitatory	KIAA0825	ENSG00000185261	0,806	0,782522262	0,612	0,85	0,26
Excitatory	ICA1	ENSG00000003147	0,791	0,616689343	0,582	0,69	0,1
Excitatory	KLHL1	ENSG00000150361	0,805	1,484632939	0,61	0,67	0,07
Excitatory	BRINP1	ENSG00000078725	0,788	0,791646927	0,576	0,73	0,14
Excitatory	RAB27B	ENSG00000041353	0,795	0,729729654	0,59	0,64	0,05
Excitatory	SPTAN1	ENSG00000197694	0,799	0,681993052	0,598	0,88	0,29
Excitatory	XKR6	ENSG00000171044	0,864	1,124997188	0,728	0,93	0,34
Excitatory	PATJ	ENSG00000132849	0,82	0,833271619	0,64	0,87	0,28
Excitatory	CLVS2	ENSG00000146352	0,792	0,610768487	0,584	0,65	0,06
Excitatory	LINC00937	ENSG00000226091	0,792	0,697979737	0,584	0,73	0,14
Excitatory	HMGCLL1	ENSG00000146151	0,791	0,644024712	0,582	0,75	0,16
Excitatory	SYP	ENSG00000102003	0,789	0,604378687	0,578	0,77	0,18
Excitatory	GNG2	ENSG00000186469	0,786	0,598771585	0,572	0,69	0,1
Excitatory	LINC01414	ENSG00000253554	0,795	1,007812523	0,59	0,66	0,07
Excitatory	NRXN1	ENSG00000179915	0,898	1,459646505	0,796	0,99	0,4
Excitatory	CACNG8	ENSG00000142408	0,792	0,60503273	0,584	0,65	0,07
Excitatory	CPNE4	ENSG00000196353	0,796	1,282271087	0,592	0,65	0,06
Excitatory	EML6	ENSG00000214595	0,789	0,735582378	0,578	0,79	0,2
Excitatory	TRERF1	ENSG00000124496	0,78	0,588678119	0,56	0,73	0,15

Excitatory	TMEM178B	ENSG00000261115	0,852	1,042136586	0,704	0,93	0,35
Excitatory	TMEM59L	ENSG00000105696	0,786	0,616464258	0,572	0,69	0,1
Excitatory	LINC02389	ENSG00000255693	0,79	0,69188611	0,58	0,64	0,06
Excitatory	ARPP21	ENSG00000172995	0,804	1,139646417	0,608	0,79	0,2
Excitatory	SCG2	ENSG00000171951	0,789	0,878328183	0,578	0,65	0,07
Excitatory	FRY	ENSG00000073910	0,793	0,70799437	0,586	0,9	0,32
Excitatory	VWC2L	ENSG00000174453	0,79	0,955024546	0,58	0,62	0,04
Excitatory	RIT2	ENSG00000152214	0,787	1,02852121	0,574	0,65	0,08
Excitatory	KIF9-AS1	ENSG00000227398	0,797	0,698827545	0,594	0,87	0,29
Excitatory	FBXL2	ENSG00000153558	0,791	0,661318018	0,582	0,8	0,22
Excitatory	GLRA3	ENSG00000145451	0,788	0,814399317	0,576	0,6	0,03
Excitatory	NDRG4	ENSG00000103034	0,778	0,587031564	0,556	0,68	0,11
Excitatory	KCND3	ENSG00000171385	0,776	0,702939115	0,552	0,74	0,17
Excitatory	CNTN3	ENSG00000113805	0,779	0,82224751	0,558	0,71	0,14
Excitatory	AC120193.1	ENSG00000253535	0,787	0,999331119	0,574	0,66	0,09
Excitatory	BAIAP3	ENSG00000007516	0,784	0,788894676	0,568	0,64	0,07
Excitatory	DPP10	ENSG00000175497	0,798	0,816658569	0,596	0,85	0,28
Excitatory	LIN7A	ENSG00000111052	0,777	0,661327948	0,554	0,67	0,11
Excitatory	CHRM3	ENSG00000133019	0,789	1,699494622	0,578	0,61	0,05
Excitatory	LINC00632	ENSG00000203930	0,862	1,021573588	0,724	0,91	0,34
Excitatory	TENM4	ENSG00000149256	0,834	1,027264622	0,668	0,9	0,33
Excitatory	UCHL1	ENSG00000154277	0,794	0,777212271	0,588	0,82	0,25
Excitatory	TAFA1	ENSG00000183662	0,79	1,401507084	0,58	0,69	0,12
Excitatory	WDR17	ENSG00000150627	0,788	0,628537077	0,576	0,81	0,24
Excitatory	FAT3	ENSG00000165323	0,763	0,642412388	0,526	0,75	0,19
Excitatory	GPR158	ENSG00000151025	0,779	0,667117542	0,558	0,85	0,28
Excitatory	SGSM1	ENSG00000167037	0,799	0,711052531	0,598	0,84	0,28
Excitatory	KCNH5	ENSG00000140015	0,784	1,003493176	0,568	0,61	0,05
Excitatory	TMEM232	ENSG00000186952	0,798	0,655461411	0,596	0,85	0,29
Excitatory	EPHB1	ENSG00000154928	0,788	0,880278767	0,576	0,72	0,15
Excitatory	CSRNP3	ENSG00000178662	0,846	0,948721257	0,692	0,92	0,35
Excitatory	TRMT9B	ENSG00000250305	0,778	0,660789787	0,556	0,67	0,11
Excitatory	HSPA4L	ENSG00000164070	0,775	0,588928437	0,55	0,73	0,17
Excitatory	PTPRO	ENSG00000151490	0,783	1,037009212	0,566	0,65	0,09
Excitatory	FSTL4	ENSG00000053108	0,78	1,235969541	0,56	0,61	0,06
Excitatory	VPS13A	ENSG00000197969	0,78	0,606632072	0,56	0,81	0,26
Excitatory	RUNX1T1	ENSG00000079102	0,776	0,744100114	0,552	0,82	0,26
Excitatory	EML5	ENSG00000165521	0,771	0,649556142	0,542	0,73	0,18
Excitatory	AFF2	ENSG00000155966	0,776	0,789599095	0,552	0,61	0,05
Excitatory	FOCAD	ENSG00000188352	0,844	0,979061284	0,688	0,91	0,36
Excitatory	MCF2L2	ENSG00000053524	0,825	0,831936796	0,65	0,89	0,34
Excitatory	LINC01322	ENSG00000244128	0,78	0,960582207	0,56	0,69	0,13
Excitatory	FAAH2	ENSG00000165591	0,774	0,626962687	0,548	0,69	0,14
Excitatory	MAGI3	ENSG00000081026	0,786	0,704044808	0,572	0,84	0,29
Excitatory	GNAL	ENSG00000141404	0,773	0,694525923	0,546	0,67	0,11
Excitatory	AC025159.1	ENSG00000257815	0,803	0,936177995	0,606	0,83	0,28
Excitatory	DAB1	ENSG00000173406	0,854	1,480675026	0,708	0,9	0,35
Excitatory	HSPA12A	ENSG00000165868	0,829	0,832447529	0,658	0,91	0,36
Excitatory	FAM135B	ENSG00000147724	0,765	0,613476019	0,53	0,75	0,2
Excitatory	AC092691.1	ENSG00000239268	0,783	0,878955425	0,566	0,88	0,33
Excitatory	RGS7	ENSG00000182901	0,825	1,02865025	0,65	0,93	0,39
Excitatory	PLCB1	ENSG00000182621	0,756	0,734305634	0,512	0,86	0,31
Excitatory	SLC25A12	ENSG00000115840	0,806	0,738874313	0,612	0,88	0,33
Excitatory	PWRN1	ENSG00000259905	0,771	0,701509028	0,542	0,78	0,24
Excitatory	CTNNA2	ENSG00000066032	0,817	0,906328657	0,634	0,97	0,43

Excitatory	CHSY3	ENSG00000198108	0,773	1,073318359	0,546	0,69	0,15
Excitatory	MCTP1	ENSG00000175471	0,766	0,77074091	0,532	0,68	0,14
Excitatory	INPP4B	ENSG00000109452	0,798	1,244364223	0,596	0,8	0,26
Excitatory	CACNA2D3	ENSG00000157445	0,764	0,770858598	0,528	0,8	0,26
Excitatory	RERG	ENSG00000134533	0,768	0,79459871	0,536	0,6	0,06
Excitatory	PCNX2	ENSG00000135749	0,805	0,682580313	0,61	0,91	0,37
Excitatory	NDST3	ENSG00000164100	0,77	0,925298996	0,54	0,6	0,06
Excitatory	ADGRV1	ENSG00000164199	0,767	0,704787038	0,534	0,64	0,1
Excitatory	NTNG1	ENSG00000162631	0,781	1,506315859	0,562	0,7	0,16
Excitatory	SLC35F4	ENSG00000151812	0,771	1,151471163	0,542	0,59	0,05
Excitatory	PLA2R1	ENSG00000153246	0,767	0,65616471	0,534	0,6	0,06
Excitatory	PLEKHA5	ENSG00000052126	0,805	0,791295148	0,61	0,93	0,4
Excitatory	NCDN	ENSG00000020129	0,764	0,628390783	0,528	0,67	0,14
Excitatory	DGKI	ENSG00000157680	0,824	0,981883833	0,648	0,9	0,37
Excitatory	CELF2	ENSG00000048740	0,768	0,76394756	0,536	0,93	0,4
Excitatory	EPHA7	ENSG00000135333	0,767	0,876818486	0,534	0,6	0,07
Excitatory	KALRN	ENSG00000160145	0,817	0,935837942	0,634	0,92	0,39
Excitatory	ABCA10	ENSG00000154263	0,762	0,597089307	0,524	0,64	0,11
Excitatory	FLRT2	ENSG00000185070	0,752	0,660096874	0,504	0,71	0,18
Excitatory	CEP112	ENSG00000154240	0,757	0,743111564	0,514	0,76	0,24
Excitatory	NSF	ENSG00000073969	0,839	0,9046371	0,678	0,94	0,42
Excitatory	UNC79	ENSG00000133958	0,839	0,9154308	0,678	0,93	0,42
Excitatory	KIAA1217	ENSG00000120549	0,755	0,878280107	0,51	0,74	0,22
Excitatory	LY6H	ENSG00000176956	0,756	0,603860476	0,512	0,57	0,06
Excitatory	PCSK1N	ENSG00000102109	0,765	0,968172785	0,53	0,71	0,2
Excitatory	CNTN6	ENSG00000134115	0,757	0,780801791	0,514	0,58	0,06
Excitatory	AC024901.1	ENSG00000255910	0,757	0,772679046	0,514	0,56	0,05
Excitatory	CDK14	ENSG00000058091	0,78	0,73357449	0,56	0,88	0,37
Excitatory	PRKG1	ENSG00000185532	0,763	1,051663566	0,526	0,74	0,23
Excitatory	UNC13C	ENSG00000137766	0,754	0,995550963	0,508	0,57	0,06
Excitatory	RBFOX1	ENSG00000078328	0,859	1,620097143	0,718	0,94	0,43
Excitatory	ZNF804B	ENSG00000182348	0,759	1,581423634	0,518	0,57	0,06
Excitatory	C8orf34	ENSG00000165084	0,754	0,929688567	0,508	0,61	0,1
Excitatory	DCC	ENSG00000187323	0,778	1,369498828	0,556	0,73	0,22
Excitatory	ZNF385B	ENSG00000144331	0,755	1,14541846	0,51	0,59	0,08
Excitatory	CLSTN2	ENSG00000158258	0,756	0,985087838	0,512	0,61	0,11
Excitatory	CUX2	ENSG00000111249	0,754	0,954978986	0,508	0,56	0,06
Excitatory	SLIT2	ENSG00000145147	0,766	1,085610814	0,532	0,68	0,17
Excitatory	ADAM22	ENSG00000008277	0,775	0,626500222	0,55	0,87	0,37
Excitatory	GRIN2A	ENSG00000183454	0,754	0,98884788	0,508	0,63	0,12
Excitatory	KIFAP3	ENSG00000075945	0,778	0,625127966	0,556	0,86	0,35
Excitatory	IDS	ENSG00000010404	0,776	0,702901831	0,552	0,83	0,33
Excitatory	GRIN3A	ENSG00000198785	0,751	0,698924684	0,502	0,54	0,04
Excitatory	KCNQ5	ENSG00000185760	0,754	1,524685706	0,508	0,57	0,07
Excitatory	VWC2	ENSG00000188730	0,748	0,824389002	0,496	0,58	0,08
Excitatory	AL033504.1	ENSG00000227681	0,748	0,943003264	0,496	0,55	0,05
Excitatory	OXR1	ENSG00000164830	0,786	0,68650776	0,572	0,91	0,41
Excitatory	LINC02223	ENSG00000249937	0,744	0,703037366	0,488	0,56	0,07
Excitatory	AC013652.1	ENSG00000259345	0,745	0,646949274	0,49	0,53	0,04
Excitatory	APBA1	ENSG00000107282	0,765	0,595928592	0,53	0,88	0,38
Excitatory	ST6GALNAC5	ENSG00000117069	0,747	1,33216422	0,494	0,53	0,04
Excitatory	FRMD4A	ENSG00000151474	0,854	0,956834809	0,708	0,97	0,48
Excitatory	CDH7	ENSG00000081138	0,746	0,749365149	0,492	0,52	0,03
Excitatory	SLIT1	ENSG00000187122	0,743	0,592372295	0,486	0,53	0,05
Excitatory	SAMD5	ENSG00000203727	0,742	0,945721374	0,484	0,54	0,06

Excitatory	AC090578.1	ENSG00000253553	0,739	0,595753742	0,478	0,57	0,08
Excitatory	ZNF385D	ENSG00000151789	0,744	1,288219651	0,488	0,6	0,11
Excitatory	ZMAT4	ENSG00000165061	0,738	0,823941456	0,476	0,54	0,06
Excitatory	PTPRT	ENSG00000196090	0,747	1,332493771	0,494	0,61	0,12
Excitatory	PLCXD3	ENSG00000182836	0,735	0,659226862	0,47	0,56	0,08
Excitatory	LRRTM3	ENSG00000198739	0,784	0,846756169	0,568	0,85	0,38
Excitatory	LUZP2	ENSG00000187398	0,725	0,709136919	0,45	0,7	0,23
Excitatory	HTR2C	ENSG00000147246	0,743	1,632985965	0,486	0,53	0,06
Excitatory	GLRA2	ENSG00000101958	0,737	0,692056935	0,474	0,52	0,05
Excitatory	CALB2	ENSG00000172137	0,736	0,681037951	0,472	0,51	0,03
Excitatory	SLIT3	ENSG00000184347	0,734	0,922183069	0,468	0,52	0,05
Excitatory	CDH4	ENSG00000179242	0,74	0,827390213	0,48	0,66	0,2
Excitatory	UBA6-AS1	ENSG00000248049	0,744	0,625575442	0,488	0,84	0,37
Excitatory	THSD7A	ENSG00000005108	0,724	0,59975237	0,448	0,72	0,25
Excitatory	LDB2	ENSG00000169744	0,732	0,843851719	0,464	0,62	0,16
Excitatory	FRAS1	ENSG00000138759	0,73	1,022381345	0,46	0,51	0,06
Excitatory	PCDH11X	ENSG00000102290	0,733	1,305678073	0,466	0,54	0,09
Excitatory	DYNC1I1	ENSG00000158560	0,79	0,738654251	0,58	0,94	0,48
Excitatory	GPC6	ENSG00000183098	0,735	1,171395302	0,47	0,64	0,19
Excitatory	MACROD2	ENSG00000172264	0,839	1,071580447	0,678	0,96	0,51
Excitatory	TMEM132D	ENSG00000151952	0,718	0,653899313	0,436	0,57	0,11
Excitatory	BRINP2	ENSG00000198797	0,722	0,595434879	0,444	0,56	0,11
Excitatory	NLGN1	ENSG00000169760	0,808	0,898108532	0,616	0,98	0,53
Excitatory	BMPER	ENSG00000164619	0,723	0,623640584	0,446	0,53	0,08
Excitatory	CNGB1	ENSG00000070729	0,724	0,63948134	0,448	0,46	0,02
Excitatory	COL25A1	ENSG00000188517	0,725	1,10022274	0,45	0,51	0,07
Excitatory	CHRM2	ENSG00000181072	0,718	0,810744439	0,436	0,49	0,04
Excitatory	CDH12	ENSG00000154162	0,741	1,542966866	0,482	0,6	0,16
Excitatory	DANT2	ENSG00000235244	0,775	0,696169659	0,55	0,91	0,47
Excitatory	GRID2	ENSG00000152208	0,82	1,240362595	0,64	0,92	0,49
Excitatory	CDH8	ENSG00000150394	0,735	0,799329387	0,47	0,73	0,29
Excitatory	ZFHX3	ENSG00000140836	0,743	0,881253572	0,486	0,83	0,4
Excitatory	CDH9	ENSG00000113100	0,718	0,862094171	0,436	0,45	0,02
Excitatory	PEG10	ENSG00000242265	0,714	0,655707276	0,428	0,56	0,13
Excitatory	CNTNAP2	ENSG00000174469	0,888	1,52535048	0,776	0,99	0,56
Excitatory	PREPL	ENSG00000138078	0,759	0,604019217	0,518	0,88	0,46
Excitatory	AEBP2	ENSG00000139154	0,751	0,641024171	0,502	0,88	0,45
Excitatory	GRM8	ENSG00000179603	0,714	0,903570044	0,428	0,52	0,1
Excitatory	PPP3CA	ENSG00000138814	0,775	0,709292737	0,55	0,94	0,52
Excitatory	DENND5B	ENSG00000170456	0,766	0,627826811	0,532	0,91	0,49
Excitatory	IQCJ-SCHIP1	ENSG00000283154	0,838	1,686951886	0,676	0,89	0,47
Excitatory	OPRM1	ENSG00000112038	0,708	0,711749325	0,416	0,46	0,04
Excitatory	PPFIA2	ENSG00000139220	0,808	0,878461858	0,616	0,96	0,54
Excitatory	RGS6	ENSG00000182732	0,701	0,779907724	0,402	0,59	0,17
Excitatory	PDE10A	ENSG00000112541	0,701	0,722805971	0,402	0,65	0,24
Excitatory	NXPH1	ENSG00000122584	0,702	1,131691449	0,404	0,51	0,1
Excitatory	SORCS1	ENSG00000108018	0,705	0,952499259	0,41	0,53	0,12
Excitatory	GRIA2	ENSG00000120251	0,799	0,827304681	0,598	0,95	0,54
Excitatory	SYNE1	ENSG00000131018	0,789	0,610206934	0,578	0,94	0,54
Excitatory	TTTY14	ENSG00000176728	0,738	0,723949619	0,476	0,79	0,39
Excitatory	LRRC4C	ENSG00000148948	0,79	0,794762065	0,58	0,92	0,53
Excitatory	CDH13	ENSG00000140945	0,702	0,867530136	0,404	0,57	0,18
Excitatory	NBEA	ENSG00000172915	0,865	1,09910362	0,73	0,95	0,57
Excitatory	NAV3	ENSG00000067798	0,822	0,971316584	0,644	0,96	0,59
Excitatory	AC124312.1	ENSG00000214265	0,859	0,916117105	0,718	0,96	0,58

Excitatory	ROBO1	ENSG00000169855	0,87	1,686411152	0,74	0,93	0,57
Excitatory	SMYD3	ENSG00000185420	0,812	0,833410591	0,624	0,95	0,59
Excitatory	NEGR1	ENSG00000172260	0,852	1,341992696	0,704	0,96	0,61
Excitatory	PHACTR1	ENSG00000112137	0,718	0,783172764	0,436	0,87	0,54
Excitatory	KAZN	ENSG00000189337	0,854	1,177578693	0,708	0,97	0,65
Excitatory	MAP1B	ENSG00000131711	0,788	0,747328116	0,576	0,97	0,65
Excitatory	DPP6	ENSG00000130226	0,751	0,675215046	0,502	0,97	0,66
Excitatory	GNAS	ENSG00000087460	0,788	0,969645586	0,576	0,93	0,62
Excitatory	AHI1	ENSG00000135541	0,88	0,982902942	0,76	0,98	0,67
Excitatory	PDE4D	ENSG00000113448	0,777	0,939199183	0,554	0,95	0,68
Excitatory	ERC1	ENSG00000082805	0,785	0,665695794	0,57	0,95	0,68
Excitatory	CACNA2D1	ENSG00000153956	0,744	0,795670263	0,488	0,91	0,66
Excitatory	ANKS1B	ENSG00000185046	0,808	0,842650867	0,616	0,98	0,74
Excitatory	ADGRL3	ENSG00000150471	0,744	0,605768288	0,488	0,98	0,77
Excitatory	LSAMP	ENSG00000185565	0,761	0,683076006	0,522	0,98	0,78
Excitatory	ADGRB3	ENSG00000135298	0,807	0,853239615	0,614	0,98	0,8
Excitatory	TNRC6A	ENSG00000090905	0,813	0,682118171	0,626	0,98	0,82
Excitatory	FTX	ENSG00000230590	0,804	0,635611403	0,608	0,98	0,93
Microglia	DOCK8	ENSG00000107099	0,98	2,991624235	0,96	0,96	0,02
Microglia	APBB1IP	ENSG00000077420	0,975	2,916228268	0,95	0,95	0,02
Microglia	ARHGAP15	ENSG00000075884	0,973	2,784046645	0,946	0,95	0,04
Microglia	FYB1	ENSG00000082074	0,962	2,650321886	0,924	0,93	0,02
Microglia	TBXAS1	ENSG00000059377	0,966	2,560827197	0,932	0,94	0,06
Microglia	PTPRC	ENSG00000081237	0,943	2,294188806	0,886	0,89	0,02
Microglia	ADAM28	ENSG00000042980	0,945	2,400703456	0,89	0,9	0,03
Microglia	INPP5D	ENSG00000168918	0,943	2,245567531	0,886	0,91	0,06
Microglia	CD74	ENSG00000019582	0,941	2,546984329	0,882	0,9	0,06
Microglia	DOCK2	ENSG00000134516	0,94	2,277365709	0,88	0,89	0,05
Microglia	CSF1R	ENSG00000182578	0,923	2,058760159	0,846	0,85	0,02
Microglia	SYK	ENSG00000165025	0,914	2,092685014	0,828	0,83	0,01
Microglia	ARHGAP24	ENSG00000138639	0,978	3,03249252	0,956	0,97	0,16
Microglia	RUNX1	ENSG00000159216	0,949	2,658780849	0,898	0,92	0,13
Microglia	SLCO2B1	ENSG00000137491	0,909	2,028970077	0,818	0,85	0,06
Microglia	C3	ENSG00000125730	0,907	2,02029567	0,814	0,84	0,05
Microglia	RIN3	ENSG00000100599	0,907	1,957166864	0,814	0,83	0,05
Microglia	ST6GAL1	ENSG00000073849	0,957	2,447245806	0,914	0,95	0,17
Microglia	PALD1	ENSG00000107719	0,913	2,03668727	0,826	0,85	0,08
Microglia	RHBDF2	ENSG00000129667	0,898	1,878199507	0,796	0,81	0,03
Microglia	ATP8B4	ENSG00000104043	0,909	2,210551197	0,818	0,83	0,06
Microglia	LYN	ENSG00000254087	0,891	1,906069396	0,782	0,79	0,03
Microglia	MEF2C	ENSG00000081189	0,947	2,327815541	0,894	0,94	0,18
Microglia	MYO1F	ENSG00000142347	0,885	1,751114265	0,77	0,78	0,04
Microglia	IKZF1	ENSG00000185811	0,875	1,946197231	0,75	0,75	0,01
Microglia	DENND3	ENSG00000105339	0,897	2,11884716	0,794	0,82	0,08
Microglia	HS3ST4	ENSG00000182601	0,899	2,644210094	0,798	0,84	0,1
Microglia	FLI1	ENSG00000151702	0,858	1,430993199	0,716	0,79	0,07
Microglia	TLR2	ENSG00000137462	0,855	1,813714723	0,71	0,71	0,01
Microglia	SP100	ENSG00000067066	0,873	1,683212851	0,746	0,79	0,09
Microglia	LY86	ENSG00000112799	0,854	1,741972888	0,708	0,71	0,01
Microglia	SLC8A1	ENSG00000183023	0,919	1,905218075	0,838	0,94	0,24
Microglia	SRGAP2B	ENSG00000196369	0,916	2,049174758	0,832	0,87	0,17
Microglia	RBM47	ENSG00000163694	0,86	1,966218079	0,72	0,73	0,03
Microglia	WDFY4	ENSG00000128815	0,852	1,653206346	0,704	0,71	0,01
Microglia	C1QB	ENSG00000173369	0,854	1,924511404	0,708	0,72	0,02
Microglia	HCLS1	ENSG00000180353	0,852	1,655153782	0,704	0,71	0,01

Microglia	ABCC4	ENSG00000125257	0,887	1,992210963	0,774	0,82	0,12
Microglia	CHST11	ENSG00000171310	0,961	2,288254222	0,922	0,97	0,28
Microglia	BNC2	ENSG00000173068	0,867	1,923883022	0,734	0,78	0,1
Microglia	LRRK1	ENSG00000154237	0,849	1,764846044	0,698	0,71	0,03
Microglia	PIK3R5	ENSG00000141506	0,841	1,682510896	0,682	0,68	0,01
Microglia	ALOX5	ENSG00000012779	0,846	1,651069443	0,692	0,7	0,02
Microglia	LINC02232	ENSG00000250125	0,844	1,970611868	0,688	0,7	0,02
Microglia	LHFPL2	ENSG00000145685	0,913	2,184610538	0,826	0,87	0,2
Microglia	SH3RF3	ENSG00000172985	0,894	2,211992046	0,788	0,83	0,16
Microglia	PIK3AP1	ENSG00000155629	0,838	1,69928445	0,676	0,68	0,01
Microglia	SLC11A1	ENSG00000018280	0,838	1,883122611	0,676	0,68	0,02
Microglia	LAPTM5	ENSG00000162511	0,836	1,553939161	0,672	0,68	0,01
Microglia	AOAH	ENSG00000136250	0,871	1,867537164	0,742	0,8	0,14
Microglia	CD53	ENSG00000143119	0,827	1,484041867	0,654	0,66	0,01
Microglia	SKAP2	ENSG00000005020	0,885	1,819423585	0,77	0,82	0,17
Microglia	FAM149A	ENSG00000109794	0,842	1,850049457	0,684	0,71	0,06
Microglia	DISC1	ENSG00000162946	0,896	1,847156953	0,792	0,87	0,22
Microglia	CYFIP1	ENSG00000273749	0,896	1,862848611	0,792	0,84	0,2
Microglia	RUNX2	ENSG00000124813	0,857	1,813395669	0,714	0,75	0,11
Microglia	NFATC2	ENSG00000101096	0,838	1,600902307	0,676	0,71	0,07
Microglia	ENTPD1	ENSG00000138185	0,84	1,686762748	0,68	0,71	0,07
Microglia	IRAK3	ENSG00000090376	0,827	1,519302322	0,654	0,71	0,07
Microglia	LPCAT2	ENSG00000087253	0,885	1,816989683	0,77	0,82	0,18
Microglia	SLC2A5	ENSG00000142583	0,821	1,547159605	0,642	0,65	0,01
Microglia	ABR	ENSG00000159842	0,932	1,940609586	0,864	0,93	0,3
Microglia	LRMDA	ENSG00000148655	0,974	2,497914773	0,948	0,98	0,35
Microglia	CPED1	ENSG00000106034	0,82	1,794816778	0,64	0,65	0,02
Microglia	SLA	ENSG00000155926	0,815	1,517044208	0,63	0,64	0,01
Microglia	MS4A6A	ENSG00000110077	0,812	1,580368368	0,624	0,63	0,01
Microglia	SCIN	ENSG00000006747	0,814	1,697402472	0,628	0,64	0,02
Microglia	CIITA	ENSG00000179583	0,814	1,489005445	0,628	0,64	0,02
Microglia	CD86	ENSG00000114013	0,808	1,549058316	0,616	0,62	0,01
Microglia	BLNK	ENSG00000095585	0,809	1,600734286	0,618	0,62	0,01
Microglia	RCSD1	ENSG00000198771	0,811	1,507638668	0,622	0,65	0,04
Microglia	IL18	ENSG00000150782	0,808	1,405192389	0,616	0,62	0,01
Microglia	TGFBR2	ENSG00000163513	0,812	1,377885529	0,624	0,69	0,08
Microglia	FOXP2	ENSG00000128573	0,82	1,549252241	0,64	0,74	0,13
Microglia	ETV6	ENSG00000139083	0,92	1,9326664	0,84	0,91	0,31
Microglia	TGFBR1	ENSG00000106799	0,85	1,831817715	0,7	0,75	0,14
Microglia	AKAP13	ENSG00000170776	0,916	1,722789308	0,832	0,91	0,31
Microglia	ARHGAP25	ENSG00000163219	0,808	1,531404485	0,616	0,63	0,02
Microglia	TFEC	ENSG00000105967	0,801	1,43294111	0,602	0,61	0,01
Microglia	KCNQ3	ENSG00000184156	0,866	2,005959664	0,732	0,83	0,24
Microglia	MGAT4A	ENSG00000071073	0,909	1,879303376	0,818	0,88	0,28
Microglia	SAMSN1	ENSG00000155307	0,801	1,638422058	0,602	0,61	0,01
Microglia	CSF3R	ENSG00000119535	0,798	1,381205627	0,596	0,6	0,01
Microglia	CELF2	ENSG00000048740	0,936	1,726446767	0,872	0,98	0,38
Microglia	OSBPL3	ENSG00000070882	0,813	1,505809217	0,626	0,67	0,09
Microglia	IFI16	ENSG00000163565	0,829	1,35587177	0,658	0,75	0,16
Microglia	FGD2	ENSG00000146192	0,792	1,379754504	0,584	0,59	0
Microglia	SMAP2	ENSG00000084070	0,881	1,768458537	0,762	0,82	0,24
Microglia	NCKAP1L	ENSG00000123338	0,792	1,315757829	0,584	0,59	0,01
Microglia	HLA-DRA	ENSG00000204287	0,794	1,63686405	0,588	0,6	0,02
Microglia	FMN1	ENSG00000248905	0,862	1,999122279	0,724	0,8	0,22
Microglia	SRGAP2	ENSG00000266028	0,963	2,286365841	0,926	0,96	0,38

Microglia	C1QC	ENSG00000159189	0,79	1,512496267	0,58	0,58	0,01
Microglia	CTSB	ENSG00000164733	0,864	1,7463214	0,728	0,8	0,22
Microglia	IPCEF1	ENSG00000074706	0,813	1,944637659	0,626	0,67	0,1
Microglia	APOE	ENSG00000130203	0,836	1,532531989	0,672	0,83	0,26
Microglia	TRPM2	ENSG00000142185	0,808	1,434552452	0,616	0,65	0,09
Microglia	HCK	ENSG00000101336	0,785	1,353694801	0,57	0,57	0,01
Microglia	ALPK1	ENSG00000073331	0,792	1,420848197	0,584	0,62	0,05
Microglia	SAT1	ENSG00000130066	0,831	1,646518128	0,662	0,75	0,19
Microglia	ZFP36L1	ENSG00000185650	0,805	1,299365749	0,61	0,74	0,19
Microglia	RREB1	ENSG00000124782	0,792	1,343782014	0,584	0,65	0,1
Microglia	DIAPH2	ENSG00000147202	0,868	1,650818968	0,736	0,84	0,29
Microglia	KCNIP1	ENSG00000182132	0,806	1,578030803	0,612	0,72	0,17
Microglia	ITGAX	ENSG00000140678	0,774	1,377008747	0,548	0,55	0,01
Microglia	LAT2	ENSG00000086730	0,779	1,257262732	0,558	0,57	0,03
Microglia	CSF2RA	ENSG00000198223	0,778	1,374454065	0,556	0,56	0,02
Microglia	IL13RA1	ENSG00000131724	0,785	1,29574741	0,57	0,62	0,08
Microglia	BMP2K	ENSG00000138756	0,94	2,042906063	0,88	0,94	0,4
Microglia	CPVL	ENSG00000106066	0,779	1,50446665	0,558	0,58	0,04
Microglia	SRGAP2C	ENSG00000171943	0,846	1,556141456	0,692	0,77	0,24
Microglia	DOCK11	ENSG00000147251	0,809	1,415515786	0,618	0,69	0,15
Microglia	CSGALNACT1	ENSG00000147408	0,807	1,558860677	0,614	0,77	0,23
Microglia	ZFHX3	ENSG00000140836	0,875	1,362510706	0,75	0,91	0,38
Microglia	LINC02798	ENSG00000227082	0,799	1,612452384	0,598	0,66	0,13
Microglia	ARHGAP26	ENSG00000145819	0,929	1,75554517	0,858	0,97	0,44
Microglia	LRCH1	ENSG00000136141	0,834	1,372662333	0,668	0,78	0,26
Microglia	LPAR6	ENSG00000139679	0,772	1,249563229	0,544	0,57	0,04
Microglia	PLA2G4A	ENSG00000116711	0,766	1,262369463	0,532	0,56	0,03
Microglia	C1QA	ENSG00000173372	0,765	1,349367523	0,53	0,54	0,01
Microglia	IL17RA	ENSG00000177663	0,798	1,332936598	0,596	0,66	0,14
Microglia	NCK2	ENSG00000071051	0,823	1,676258603	0,646	0,72	0,2
Microglia	GNB4	ENSG00000114450	0,779	1,2784887	0,558	0,61	0,09
Microglia	TMEM156	ENSG00000121895	0,763	1,376262434	0,526	0,53	0,01
Microglia	ITPR2	ENSG00000123104	0,928	1,862913817	0,856	0,96	0,44
Microglia	POU2F2	ENSG00000028277	0,781	1,351327429	0,562	0,61	0,09
Microglia	ST8SIA4	ENSG00000113532	0,774	1,407790343	0,548	0,59	0,07
Microglia	TLR1	ENSG00000174125	0,759	1,182164824	0,518	0,53	0,02
Microglia	FRMD4A	ENSG00000151474	0,943	2,057261328	0,886	0,98	0,47
Microglia	RGS10	ENSG00000148908	0,756	1,193518603	0,512	0,52	0,01
Microglia	SUSD6	ENSG00000100647	0,825	1,367238615	0,65	0,75	0,24
Microglia	TNFRSF1B	ENSG00000028137	0,76	1,254772607	0,52	0,55	0,04
Microglia	HLA-DRB1	ENSG00000196126	0,758	1,336991308	0,516	0,53	0,03
Microglia	TNS3	ENSG00000136205	0,792	1,373226346	0,584	0,68	0,18
Microglia	FMNL3	ENSG00000161791	0,793	1,213680497	0,586	0,71	0,2
Microglia	CAMK1D	ENSG00000183049	0,814	1,326609312	0,628	0,78	0,28
Microglia	P2RY12	ENSG00000169313	0,757	1,591758509	0,514	0,53	0,03
Microglia	PAG1	ENSG00000076641	0,846	1,529138978	0,692	0,8	0,31
Microglia	VAV1	ENSG00000141968	0,751	1,166102644	0,502	0,51	0,01
Microglia	SLC25A37	ENSG00000147454	0,838	1,636744651	0,676	0,77	0,27
Microglia	CYBA	ENSG00000051523	0,752	1,162501011	0,504	0,54	0,05
Microglia	MAML3	ENSG00000196782	0,894	1,720040365	0,788	0,91	0,41
Microglia	SLC4A7	ENSG00000033867	0,802	1,392943884	0,604	0,7	0,21
Microglia	SH3TC1	ENSG00000125089	0,748	1,151262051	0,496	0,51	0,01
Microglia	FCGR2A	ENSG00000143226	0,747	1,245371062	0,494	0,5	0,01
Microglia	PRKAG2	ENSG00000106617	0,797	1,361323778	0,594	0,71	0,22
Microglia	MTHFD1L	ENSG00000120254	0,853	1,531701506	0,706	0,82	0,34

Microglia	ZFP36L2	ENSG00000152518	0,778	1,394766116	0,556	0,65	0,17
Microglia	GPRIN3	ENSG00000185477	0,767	1,280220887	0,534	0,6	0,12
Microglia	LRMP	ENSG00000118308	0,743	1,195411115	0,486	0,5	0,02
Microglia	A2M	ENSG00000175899	0,742	0,973158151	0,484	0,59	0,11
Microglia	SRGN	ENSG00000122862	0,741	1,501584414	0,482	0,54	0,06
Microglia	KCTD12	ENSG00000178695	0,746	1,082458037	0,492	0,55	0,07
Microglia	PDE3B	ENSG00000152270	0,773	1,537728023	0,546	0,64	0,17
Microglia	MSR1	ENSG00000038945	0,742	1,347371604	0,484	0,51	0,04
Microglia	PLXDC2	ENSG00000120594	0,986	2,760625668	0,972	0,99	0,52
Microglia	ANOS1	ENSG00000011201	0,753	1,233937156	0,506	0,57	0,1
Microglia	CCND3	ENSG00000112576	0,783	1,347771065	0,566	0,67	0,21
Microglia	ZNF710	ENSG00000140548	0,79	1,284211053	0,58	0,68	0,22
Microglia	ARHGAP6	ENSG00000047648	0,754	1,256213996	0,508	0,59	0,13
Microglia	ADCY7	ENSG00000121281	0,738	1,068470546	0,476	0,49	0,03
Microglia	REL	ENSG00000162924	0,759	1,185587612	0,518	0,58	0,12
Microglia	CTSC	ENSG00000109861	0,738	1,135853001	0,476	0,5	0,04
Microglia	BACH1	ENSG00000156273	0,818	1,548030126	0,636	0,77	0,3
Microglia	KCNK13	ENSG00000152315	0,736	1,326069528	0,472	0,49	0,03
Microglia	CARD11	ENSG00000198286	0,736	1,234611736	0,472	0,48	0,02
Microglia	TBC1D1	ENSG00000065882	0,782	1,223728938	0,564	0,69	0,23
Microglia	GAB3	ENSG00000160219	0,733	1,085603466	0,466	0,48	0,02
Microglia	RAB31	ENSG00000168461	0,776	1,123323566	0,552	0,72	0,26
Microglia	TM6SF1	ENSG00000136404	0,738	1,119147549	0,476	0,51	0,05
Microglia	PRAM1	ENSG00000133246	0,733	1,073653661	0,466	0,48	0,03
Microglia	IGSF21	ENSG00000117154	0,747	1,362204614	0,494	0,57	0,11
Microglia	MYOF	ENSG00000138119	0,729	0,95437921	0,458	0,53	0,08
Microglia	MIR646HG	ENSG00000228340	0,753	1,41106182	0,506	0,6	0,15
Microglia	TSPAN14	ENSG00000108219	0,762	1,129541146	0,524	0,63	0,19
Microglia	MAP3K5	ENSG00000197442	0,751	0,852399873	0,502	0,74	0,3
Microglia	CMTM7	ENSG00000153551	0,724	1,054956396	0,448	0,47	0,03
Microglia	RAPGEF1	ENSG00000107263	0,833	1,376964036	0,666	0,81	0,37
Microglia	KCNMA1	ENSG00000156113	0,823	1,537017014	0,646	0,85	0,41
Microglia	LINC00278	ENSG00000231535	0,725	1,315511745	0,45	0,49	0,05
Microglia	FCGR3A	ENSG00000203747	0,719	1,088982338	0,438	0,44	0,01
Microglia	RYR1	ENSG00000196218	0,744	1,147445409	0,488	0,57	0,13
Microglia	MAF	ENSG00000178573	0,737	1,129975854	0,474	0,54	0,11
Microglia	ITGAM	ENSG00000169896	0,718	1,044379594	0,436	0,44	0,01
Microglia	PGM5	ENSG00000154330	0,738	1,158382515	0,476	0,55	0,11
Microglia	LCP2	ENSG00000043462	0,721	1,095063523	0,442	0,46	0,03
Microglia	HLA-DPA1	ENSG00000231389	0,72	1,13041294	0,44	0,46	0,02
Microglia	RUBCNL	ENSG00000102445	0,727	1,101716806	0,454	0,49	0,06
Microglia	KYNU	ENSG00000115919	0,717	1,116219804	0,434	0,44	0,01
Microglia	ITGB2	ENSG00000160255	0,717	0,975283265	0,434	0,44	0,01
Microglia	OXR1	ENSG00000164830	0,838	1,760907387	0,676	0,84	0,41
Microglia	ARRB2	ENSG00000141480	0,736	1,018595462	0,472	0,53	0,1
Microglia	CD14	ENSG00000170458	0,715	1,243688221	0,43	0,44	0,01
Microglia	GRB2	ENSG00000177885	0,822	1,416770315	0,644	0,76	0,33
Microglia	NIBAN1	ENSG00000135842	0,717	1,391780769	0,434	0,45	0,03
Microglia	TYROBP	ENSG00000011600	0,714	1,000309901	0,428	0,43	0,01
Microglia	SORL1	ENSG00000137642	0,945	2,010540699	0,89	0,96	0,53
Microglia	PELI1	ENSG00000197329	0,786	1,32984157	0,572	0,71	0,29
Microglia	BHLHE41	ENSG00000123095	0,786	1,247212406	0,572	0,7	0,28
Microglia	LINC01094	ENSG00000251442	0,712	0,956779842	0,424	0,5	0,07
Microglia	CLEC7A	ENSG00000172243	0,711	1,012244942	0,422	0,42	0
Microglia	MIS18BP1	ENSG00000129534	0,729	1,130552421	0,458	0,52	0,1

Microglia	CTSS	ENSG00000163131	0,718	1,011300085	0,436	0,46	0,04
Microglia	LRRK2	ENSG00000188906	0,735	1,172909021	0,47	0,57	0,15
Microglia	ETS2	ENSG00000157557	0,719	0,988927568	0,438	0,52	0,1
Microglia	APOC1	ENSG00000130208	0,713	1,226499974	0,426	0,45	0,03
Microglia	PRKCH	ENSG00000027075	0,712	0,873643554	0,424	0,53	0,11
Microglia	PARVG	ENSG00000138964	0,71	0,997973253	0,42	0,43	0,01
Microglia	PARP14	ENSG00000173193	0,713	0,896764288	0,426	0,51	0,1
Microglia	CHST15	ENSG00000182022	0,723	1,149603477	0,446	0,52	0,11
Microglia	NHSL1	ENSG00000135540	0,73	1,580479973	0,46	0,59	0,18
Microglia	RGS1	ENSG00000090104	0,705	1,46116494	0,41	0,41	0
Microglia	VSIG4	ENSG00000155659	0,705	1,09357028	0,41	0,41	0
Microglia	UTRN	ENSG00000152818	0,707	0,613724677	0,414	0,7	0,29
Microglia	PCNX2	ENSG00000135749	0,793	1,172970085	0,586	0,78	0,37
Microglia	SPI1	ENSG00000066336	0,706	0,942054632	0,412	0,43	0,02
Microglia	OLR1	ENSG00000173391	0,702	1,163325255	0,404	0,41	0
Microglia	MSN	ENSG00000147065	0,731	1,012083727	0,462	0,58	0,18
Microglia	ADGRG1	ENSG00000205336	0,719	0,967377366	0,438	0,54	0,14
Microglia	OTULINL	ENSG00000145569	0,704	1,017119428	0,408	0,42	0,02
Microglia	FGD4	ENSG00000139132	0,895	1,552486407	0,79	0,92	0,52
Microglia	PLCL2	ENSG00000154822	0,734	1,077305733	0,468	0,6	0,2
Microglia	B3GNT5	ENSG00000176597	0,702	1,037580377	0,404	0,42	0,03
Microglia	SLC1A3	ENSG00000079215	0,935	1,971717822	0,87	0,96	0,57
Microglia	PARP8	ENSG00000151883	0,73	1,089421882	0,46	0,62	0,23
Microglia	MAN1A1	ENSG00000111885	0,711	1,096519076	0,422	0,51	0,12
Microglia	IFNGR2	ENSG00000159128	0,735	1,077901283	0,47	0,57	0,19
Microglia	TBC1D2B	ENSG00000167202	0,731	1,011204508	0,462	0,57	0,19
Microglia	CARD8	ENSG00000105483	0,716	0,908379721	0,432	0,53	0,15
Microglia	GALNT2	ENSG00000143641	0,806	1,289949058	0,612	0,77	0,38
Microglia	COTL1	ENSG00000103187	0,711	1,022057924	0,422	0,5	0,12
Microglia	MERTK	ENSG00000153208	0,709	1,345796531	0,418	0,5	0,12
Microglia	GRK3	ENSG00000100077	0,75	1,002859311	0,5	0,69	0,31
Microglia	ARHGAP22	ENSG00000128805	0,85	1,346968455	0,7	0,88	0,5
Microglia	PLEKHA2	ENSG00000169499	0,717	0,955196489	0,434	0,54	0,16
Microglia	IFNGR1	ENSG00000027697	0,759	1,082718859	0,518	0,67	0,29
Microglia	KCNQ1	ENSG00000053918	0,726	1,093858092	0,452	0,55	0,17
Microglia	USP6NL	ENSG00000148429	0,74	1,06332604	0,48	0,63	0,26
Microglia	STK4	ENSG00000101109	0,745	1,062140042	0,49	0,63	0,25
Microglia	TBC1D14	ENSG00000132405	0,75	1,366911545	0,5	0,62	0,25
Microglia	CMIP	ENSG00000153815	0,838	1,259739477	0,676	0,84	0,47
Microglia	SOAT1	ENSG00000057252	0,708	0,953651644	0,416	0,5	0,13
Microglia	SFMBT2	ENSG00000198879	0,918	1,73622818	0,836	0,95	0,58
Microglia	LRRFIP1	ENSG00000124831	0,736	0,950251691	0,472	0,65	0,29
Microglia	SSH1	ENSG00000084112	0,742	1,037670808	0,484	0,62	0,26
Microglia	ST3GAL6	ENSG00000064225	0,717	0,892423548	0,434	0,59	0,23
Microglia	FCHSD2	ENSG00000137478	0,796	0,978554564	0,592	0,88	0,52
Microglia	EML4	ENSG00000143924	0,735	1,064778792	0,47	0,61	0,25
Microglia	GALNT10	ENSG00000164574	0,736	1,119266033	0,472	0,63	0,26
Microglia	SDK1	ENSG00000146555	0,715	1,184720479	0,43	0,6	0,24
Microglia	EPB41	ENSG00000159023	0,706	0,9641749	0,412	0,54	0,18
Microglia	PPARD	ENSG00000112033	0,746	1,225046629	0,492	0,64	0,28
Microglia	ITPR1	ENSG00000150995	0,716	1,018476966	0,432	0,57	0,22
Microglia	SKI	ENSG00000157933	0,741	1,061048743	0,482	0,63	0,28
Microglia	FOXN3	ENSG00000053254	0,932	1,719614776	0,864	0,96	0,61
Microglia	APMAP	ENSG00000101474	0,702	0,971531406	0,404	0,49	0,14
Microglia	HIF1A	ENSG00000100644	0,758	1,270050602	0,516	0,69	0,34

Microglia	SIPA1L2	ENSG00000116991	0,708	0,985184871	0,416	0,55	0,2
Microglia	TAB2	ENSG00000055208	0,82	1,283212162	0,64	0,81	0,47
Microglia	MEF2A	ENSG00000068305	0,965	2,000732047	0,93	0,98	0,64
Microglia	JAK2	ENSG00000096968	0,746	1,031028017	0,492	0,67	0,33
Microglia	SOCS6	ENSG00000170677	0,72	1,1869071	0,44	0,56	0,22
Microglia	SYNDIG1	ENSG00000101463	0,822	1,781592107	0,644	0,81	0,48
Microglia	SKIL	ENSG00000136603	0,707	0,966248412	0,414	0,54	0,21
Microglia	VOPP1	ENSG00000154978	0,75	1,047289753	0,5	0,67	0,34
Microglia	ACSL1	ENSG00000151726	0,765	1,492772902	0,53	0,69	0,36
Microglia	NUMB	ENSG00000133961	0,786	1,086667056	0,572	0,76	0,43
Microglia	PACSIN2	ENSG00000100266	0,721	0,94298667	0,442	0,6	0,27
Microglia	FAM49B	ENSG00000153310	0,921	1,46713839	0,842	0,96	0,63
Microglia	AP1B1	ENSG00000100280	0,709	0,935404872	0,418	0,55	0,22
Microglia	PTPRE	ENSG00000132334	0,789	1,285397828	0,578	0,78	0,45
Microglia	LIMS1	ENSG00000169756	0,758	1,140250436	0,516	0,72	0,39
Microglia	CTTNBP2NL	ENSG00000143079	0,707	0,928113482	0,414	0,56	0,24
Microglia	TBC1D22A	ENSG00000054611	0,878	1,378514437	0,756	0,91	0,58
Microglia	MAML2	ENSG00000184384	0,854	1,268994258	0,708	0,94	0,62
Microglia	USP15	ENSG00000135655	0,81	1,168973896	0,62	0,81	0,49
Microglia	SH3BGRL	ENSG00000131171	0,753	1,096147836	0,506	0,69	0,38
Microglia	MANBA	ENSG00000109323	0,732	1,007890723	0,464	0,64	0,33
Microglia	LPIN2	ENSG00000101577	0,72	0,946754406	0,44	0,61	0,3
Microglia	IL6ST	ENSG00000134352	0,764	1,108267993	0,528	0,73	0,42
Microglia	NCOA3	ENSG00000124151	0,729	0,956187184	0,458	0,65	0,34
Microglia	SUCLG2	ENSG00000172340	0,706	0,880775369	0,412	0,61	0,3
Microglia	RGS12	ENSG00000159788	0,705	0,929496577	0,41	0,58	0,27
Microglia	EPB41L2	ENSG00000079819	0,921	1,688127021	0,842	0,95	0,65
Microglia	STX7	ENSG00000079950	0,763	1,024777149	0,526	0,73	0,42
Microglia	TNRC18	ENSG00000182095	0,718	0,897591928	0,436	0,63	0,33
Microglia	TMSB4X	ENSG00000205542	0,762	1,025207735	0,524	0,78	0,48
Microglia	SH3KBP1	ENSG00000147010	0,801	1,063579091	0,602	0,84	0,54
Microglia	PTPN2	ENSG00000175354	0,718	1,083675411	0,436	0,59	0,3
Microglia	SRGAP1	ENSG00000196935	0,794	1,308351214	0,588	0,8	0,51
Microglia	FAM13A	ENSG00000138640	0,712	0,975423688	0,424	0,63	0,34
Microglia	LDLRAD4	ENSG00000168675	0,927	1,745138903	0,854	0,96	0,67
Microglia	RB1	ENSG00000139687	0,777	1,099851621	0,554	0,77	0,48
Microglia	B2M	ENSG00000166710	0,708	0,723862668	0,416	0,68	0,39
Microglia	CAB39	ENSG00000135932	0,74	1,004495693	0,48	0,68	0,39
Microglia	RNF213	ENSG00000173821	0,727	0,89005906	0,454	0,67	0,38
Microglia	FNDC3B	ENSG00000075420	0,705	0,945284186	0,41	0,64	0,35
Microglia	ARL15	ENSG00000185305	0,72	0,733012094	0,44	0,73	0,44
Microglia	NAV3	ENSG00000067798	0,761	0,907891347	0,522	0,87	0,59
Microglia	ANKRD44	ENSG00000065413	0,91	1,549504284	0,82	0,95	0,67
Microglia	JAZF1	ENSG00000153814	0,847	1,365777895	0,694	0,89	0,61
Microglia	PACS1	ENSG00000175115	0,718	0,870856955	0,436	0,69	0,4
Microglia	FKBP5	ENSG00000096060	0,898	1,452187578	0,796	0,96	0,67
Microglia	PADI2	ENSG00000117115	0,726	1,171572972	0,452	0,69	0,4
Microglia	RNF149	ENSG00000163162	0,744	1,107011528	0,488	0,68	0,4
Microglia	PTPRJ	ENSG00000149177	0,819	1,170126429	0,638	0,87	0,59
Microglia	PREX1	ENSG00000124126	0,82	1,050740289	0,64	0,9	0,62
Microglia	SPP1	ENSG00000118785	0,79	1,769240397	0,58	0,87	0,59
Microglia	ABI1	ENSG00000136754	0,705	0,811257636	0,41	0,64	0,37
Microglia	H2AFY	ENSG00000113648	0,714	0,890614404	0,428	0,61	0,34
Microglia	ANKS1A	ENSG00000064999	0,756	0,947595871	0,512	0,76	0,49
Microglia	RASAL2	ENSG00000075391	0,731	0,896414871	0,462	0,77	0,5

Microglia	P4HA1	ENSG00000122884	0,707	1,062655212	0,414	0,63	0,38
Microglia	AFF1	ENSG00000172493	0,727	0,851398281	0,454	0,73	0,49
Microglia	HDAC9	ENSG00000048052	0,723	1,163463972	0,446	0,72	0,48
Microglia	RAP1A	ENSG00000116473	0,769	0,942391193	0,538	0,81	0,57
Microglia	ATF6	ENSG00000118217	0,73	0,878982789	0,46	0,71	0,48
Microglia	RASSF8	ENSG00000123094	0,714	1,187375144	0,428	0,68	0,45
Microglia	ATM	ENSG00000149311	0,792	0,983398621	0,584	0,83	0,6
Microglia	PIAS1	ENSG00000033800	0,769	0,87729274	0,538	0,81	0,58
Microglia	CAPZB	ENSG00000077549	0,757	0,903791128	0,514	0,77	0,54
Microglia	TTC7A	ENSG00000068724	0,703	0,900815802	0,406	0,67	0,46
Microglia	SPRED1	ENSG00000166068	0,72	0,88193229	0,44	0,72	0,51
Microglia	NAIP	ENSG00000249437	0,757	0,953786002	0,514	0,78	0,57
Microglia	WASF2	ENSG00000158195	0,704	0,745998545	0,408	0,69	0,49
Microglia	PLCG2	ENSG00000197943	0,701	0,611388045	0,402	0,79	0,59
Microglia	SSH2	ENSG00000141298	0,938	1,503862219	0,876	0,98	0,79
Microglia	PHC2	ENSG00000134686	0,721	0,839523287	0,442	0,72	0,52
Microglia	ATP2C1	ENSG00000017260	0,717	0,850015409	0,434	0,72	0,52
Microglia	SPTLC2	ENSG00000100596	0,707	0,840289658	0,414	0,71	0,52
Microglia	SLC9A9	ENSG00000181804	0,785	0,948358412	0,57	0,89	0,7
Microglia	STAG1	ENSG00000118007	0,749	0,768175026	0,498	0,84	0,69
Microglia	PKN2	ENSG00000065243	0,717	0,754431145	0,434	0,76	0,61
Microglia	MED13L	ENSG00000123066	0,851	1,092969494	0,702	0,94	0,79
Microglia	GAB2	ENSG00000033327	0,721	0,734857787	0,442	0,85	0,71
Microglia	LPP	ENSG00000145012	0,74	0,762475111	0,48	0,85	0,72
Microglia	MBNL1	ENSG00000152601	0,844	1,037904047	0,688	0,95	0,82
Microglia	ANKRD11	ENSG00000167522	0,739	0,750230556	0,478	0,82	0,69
Microglia	MYCBP2	ENSG00000005810	0,74	0,702479701	0,48	0,86	0,73
Microglia	UBE2E2	ENSG00000182247	0,747	0,737444781	0,494	0,92	0,79
Microglia	SDCCAG8	ENSG00000054282	0,75	0,777068546	0,5	0,85	0,73
Microglia	PICALM	ENSG00000073921	0,836	1,003711625	0,672	0,96	0,84
Microglia	DOCK4	ENSG00000128512	0,956	1,750929775	0,912	0,99	0,9
Microglia	GNAQ	ENSG00000156052	0,838	0,934364541	0,676	0,95	0,87
Microglia	ZFAND3	ENSG00000156639	0,753	0,705351092	0,506	0,9	0,82
Microglia	NIPBL	ENSG00000164190	0,703	0,591850269	0,406	0,83	0,76
Microglia	FOXP1	ENSG00000114861	0,72	0,729857423	0,44	0,86	0,81
OPCs	MEGF11	ENSG00000157890	0,983	2,621750837	0,966	0,97	0,06
OPCs	VCAN	ENSG00000038427	0,994	2,941497108	0,988	0,99	0,09
OPCs	CA10	ENSG00000154975	0,962	2,319424768	0,924	0,96	0,12
OPCs	PCDH15	ENSG00000150275	0,975	2,486761309	0,95	0,99	0,15
OPCs	NXPH1	ENSG00000122584	0,925	1,934946985	0,85	0,91	0,08
OPCs	SNTG1	ENSG00000147481	0,939	1,899191889	0,878	0,98	0,15
OPCs	TNR	ENSG00000116147	0,998	3,375104408	0,996	1	0,17
OPCs	MMP16	ENSG00000156103	0,977	2,578156823	0,954	0,97	0,15
OPCs	OPCML	ENSG00000183715	0,979	2,510720776	0,958	0,99	0,18
OPCs	LHFPL3	ENSG00000187416	0,986	2,894139696	0,972	0,99	0,18
OPCs	GRIK1	ENSG00000171189	0,922	1,525092041	0,844	0,92	0,11
OPCs	ATRNL1	ENSG00000107518	0,97	2,311314838	0,94	0,98	0,17
OPCs	TMEM132D	ENSG00000151952	0,926	1,850847178	0,852	0,89	0,09
OPCs	COL11A1	ENSG00000060718	0,929	1,869221927	0,858	0,9	0,1
OPCs	PTPRZ1	ENSG00000106278	0,99	2,517146329	0,98	1	0,2
OPCs	SOX6	ENSG00000110693	0,971	2,271712733	0,942	0,98	0,19
OPCs	BRINP3	ENSG00000162670	0,945	1,96996274	0,89	0,96	0,16
OPCs	CHST9	ENSG00000154080	0,904	1,836818823	0,808	0,83	0,04
OPCs	COL9A1	ENSG00000112280	0,898	1,621103344	0,796	0,8	0,02
OPCs	SLC35F1	ENSG00000196376	0,96	2,140495779	0,92	0,97	0,19

OPCs	ADAMTS17	ENSG00000140470	0,911	1,826406835	0,822	0,85	0,08
OPCs	GRM7	ENSG00000196277	0,921	1,691065421	0,842	0,96	0,19
OPCs	FGF12	ENSG00000114279	0,907	1,593568017	0,814	0,95	0,19
OPCs	GRM5	ENSG00000168959	0,892	1,416298645	0,784	0,93	0,17
OPCs	LUZP2	ENSG00000187398	0,935	1,820654593	0,87	0,96	0,21
OPCs	TMEM132C	ENSG00000181234	0,95	1,950322053	0,9	0,96	0,21
OPCs	BCAN	ENSG00000132692	0,893	1,527816538	0,786	0,83	0,08
OPCs	FGF14	ENSG00000102466	0,948	1,983511926	0,896	0,99	0,25
OPCs	DCC	ENSG00000187323	0,913	1,577760314	0,826	0,95	0,21
OPCs	HIF3A	ENSG00000124440	0,881	1,213466135	0,762	0,94	0,19
OPCs	SCN1A	ENSG00000144285	0,896	1,43741716	0,792	0,91	0,16
OPCs	KCNIP4	ENSG00000185774	0,93	1,645571584	0,86	0,99	0,25
OPCs	DGKG	ENSG00000058866	0,958	1,984514884	0,916	0,98	0,24
OPCs	KCND2	ENSG00000184408	0,942	1,877548565	0,884	0,99	0,25
OPCs	LRRTM4	ENSG00000176204	0,947	1,95640539	0,894	0,99	0,26
OPCs	CHL1	ENSG00000134121	0,932	1,706888956	0,864	0,95	0,22
OPCs	LINC00511	ENSG00000227036	0,938	1,754459933	0,876	0,95	0,22
OPCs	NRCAM	ENSG00000091129	0,983	2,256571231	0,966	1	0,27
OPCs	DSCAM	ENSG00000171587	0,991	2,79017089	0,982	1	0,27
OPCs	FERMT1	ENSG00000101311	0,862	1,479737071	0,724	0,73	0,02
OPCs	UNC80	ENSG00000144406	0,89	1,416669453	0,78	0,9	0,19
OPCs	GRIK2	ENSG00000164418	0,909	1,425559135	0,818	0,97	0,26
OPCs	ARPP21	ENSG00000172995	0,898	1,429050286	0,796	0,91	0,2
OPCs	ZFPM2	ENSG00000169946	0,942	2,139775344	0,884	0,96	0,25
OPCs	CNTNAP5	ENSG00000155052	0,857	1,135780529	0,714	0,86	0,15
OPCs	SLC1A2	ENSG00000110436	0,875	1,000087503	0,75	0,92	0,22
OPCs	PCDH7	ENSG00000169851	0,893	1,444196743	0,786	0,95	0,25
OPCs	HS6ST3	ENSG00000185352	0,86	1,331689479	0,72	0,87	0,16
OPCs	SORCS3	ENSG00000156395	0,864	1,384938605	0,728	0,81	0,11
OPCs	PLPP4	ENSG00000203805	0,882	1,506936232	0,764	0,82	0,12
OPCs	MYT1	ENSG00000196132	0,868	1,385383934	0,736	0,76	0,06
OPCs	SLC24A3	ENSG00000185052	0,868	1,462547442	0,736	0,82	0,13
OPCs	UST	ENSG00000111962	0,879	1,424565427	0,758	0,86	0,17
OPCs	CSMD2	ENSG00000121904	0,907	1,535903505	0,814	0,92	0,23
OPCs	CACNA1A	ENSG00000141837	0,879	1,25186385	0,758	0,93	0,24
OPCs	PDZRN4	ENSG00000165966	0,863	1,587353366	0,726	0,82	0,14
OPCs	HECW1	ENSG00000002746	0,849	1,240789497	0,698	0,81	0,12
OPCs	FAM155A	ENSG00000204442	0,872	1,199190208	0,744	0,98	0,3
OPCs	SEZ6L	ENSG00000100095	0,86	1,487873791	0,72	0,82	0,13
OPCs	DLGAP1	ENSG00000170579	0,871	1,037708659	0,742	0,93	0,25
OPCs	NTRK3	ENSG00000140538	0,887	1,254639321	0,774	0,95	0,27
OPCs	SULF2	ENSG00000196562	0,855	1,299239131	0,71	0,77	0,09
OPCs	PID1	ENSG00000153823	0,881	1,445274894	0,762	0,87	0,19
OPCs	KCNIP1	ENSG00000182132	0,86	1,277973697	0,72	0,85	0,18
OPCs	SORCS1	ENSG00000108018	0,849	1,32487608	0,698	0,78	0,1
OPCs	AC004852.2	ENSG00000278254	0,837	1,844784452	0,674	0,68	0,01
OPCs	CSMD3	ENSG00000164796	0,939	1,736324337	0,878	0,98	0,31
OPCs	MDGA2	ENSG00000139915	0,916	1,520815274	0,832	0,98	0,32
OPCs	STK32A	ENSG00000169302	0,838	1,106038091	0,676	0,8	0,13
OPCs	SMOC1	ENSG00000198732	0,96	1,944753885	0,92	0,97	0,31
OPCs	CNTN3	ENSG00000113805	0,859	1,40952387	0,718	0,81	0,14
OPCs	XKR4	ENSG00000206579	0,846	1,389903403	0,692	0,81	0,15
OPCs	SOX5	ENSG00000134532	0,894	1,391110845	0,788	0,98	0,32
OPCs	PTPRG	ENSG00000144724	0,86	0,971610987	0,72	0,95	0,29
OPCs	SHISA9	ENSG00000237515	0,84	1,381418625	0,68	0,79	0,14

OPCs	GPM6A	ENSG00000150625	0,843	0,962555376	0,686	0,96	0,31
OPCs	GSG1L	ENSG00000169181	0,845	1,273355132	0,69	0,74	0,08
OPCs	CHST11	ENSG00000171310	0,879	1,202847507	0,758	0,95	0,3
OPCs	CNTN1	ENSG00000018236	0,868	1,237233793	0,736	0,96	0,31
OPCs	NOL4	ENSG00000101746	0,848	1,206828232	0,696	0,83	0,18
OPCs	ALK	ENSG00000171094	0,876	1,668154573	0,752	0,84	0,19
OPCs	APCDD1	ENSG00000154856	0,846	1,276587207	0,692	0,78	0,14
OPCs	AMZ1	ENSG00000174945	0,838	1,28709037	0,676	0,72	0,08
OPCs	LINC01322	ENSG00000244128	0,846	1,459728585	0,692	0,77	0,13
OPCs	CSMD1	ENSG00000183117	0,97	2,305673515	0,94	1	0,36
OPCs	LRRK2	ENSG00000188906	0,838	1,13518151	0,676	0,79	0,15
OPCs	RNF150	ENSG00000170153	0,857	1,292896627	0,714	0,84	0,21
OPCs	CDH10	ENSG00000040731	0,842	1,312630768	0,684	0,78	0,15
OPCs	SCN9A	ENSG00000169432	0,83	1,140260212	0,66	0,75	0,12
OPCs	CSGALNACT1	ENSG00000147408	0,828	0,955877876	0,656	0,87	0,24
OPCs	STK32B	ENSG00000152953	0,823	1,333123878	0,646	0,7	0,07
OPCs	SCN3A	ENSG00000153253	0,822	1,074392342	0,644	0,76	0,14
OPCs	PLCB1	ENSG00000182621	0,827	0,892829671	0,654	0,94	0,31
OPCs	POU6F2	ENSG00000106536	0,821	1,369037632	0,642	0,7	0,08
OPCs	AC092691.1	ENSG00000239268	0,855	1,159477162	0,71	0,95	0,33
OPCs	STXBP5L	ENSG00000145087	0,804	0,84414313	0,608	0,83	0,21
OPCs	KIAA1217	ENSG00000120549	0,823	1,095516424	0,646	0,83	0,22
OPCs	PLEKHH2	ENSG00000152527	0,82	1,10705455	0,64	0,71	0,09
OPCs	SEMA3E	ENSG00000170381	0,821	1,301502062	0,642	0,7	0,09
OPCs	TOX	ENSG00000198846	0,818	1,106050729	0,636	0,77	0,16
OPCs	OPHN1	ENSG00000079482	0,937	1,783127479	0,874	0,95	0,34
OPCs	RAB31	ENSG00000168461	0,861	1,233854048	0,722	0,88	0,26
OPCs	CACNG4	ENSG00000075461	0,82	1,196422653	0,64	0,67	0,05
OPCs	SLC22A3	ENSG00000146477	0,811	1,242234019	0,622	0,65	0,03
OPCs	CSPG4	ENSG00000173546	0,805	1,061066642	0,61	0,63	0,02
OPCs	ATP13A4	ENSG00000127249	0,836	1,132752753	0,672	0,81	0,2
OPCs	ZEB1	ENSG00000148516	0,856	1,141916074	0,712	0,92	0,31
OPCs	AC007563.2	ENSG00000236886	0,812	1,502134699	0,624	0,67	0,06
OPCs	KIF26B	ENSG00000162849	0,832	1,245719855	0,664	0,78	0,18
OPCs	NLGN4X	ENSG00000146938	0,9	1,572748331	0,8	0,9	0,3
OPCs	CDH13	ENSG00000140945	0,816	1,087141023	0,632	0,77	0,17
OPCs	IGSF21	ENSG00000117154	0,804	0,937286988	0,608	0,71	0,11
OPCs	LRRN1	ENSG00000175928	0,828	1,196380931	0,656	0,74	0,14
OPCs	TAFA1	ENSG00000183662	0,809	1,360035609	0,618	0,71	0,12
OPCs	PRRX1	ENSG00000116132	0,793	1,026790704	0,586	0,69	0,1
OPCs	NTN1	ENSG00000065320	0,802	1,070793247	0,604	0,66	0,07
OPCs	NRXN1	ENSG00000179915	0,917	1,531849618	0,834	0,99	0,4
OPCs	CRISPLD2	ENSG00000103196	0,797	1,199003818	0,594	0,62	0,04
OPCs	MAP2	ENSG00000078018	0,88	1,270113266	0,76	0,96	0,37
OPCs	MPPED2	ENSG00000066382	0,823	1,206892975	0,646	0,75	0,17
OPCs	PDZD2	ENSG00000133401	0,979	2,352019131	0,958	0,99	0,41
OPCs	TNK2	ENSG00000061938	0,864	1,273514614	0,728	0,84	0,26
OPCs	SHC3	ENSG00000148082	0,817	1,142888696	0,634	0,72	0,14
OPCs	LINC02588	ENSG00000257842	0,803	1,37314315	0,606	0,65	0,07
OPCs	C1orf21	ENSG00000116667	0,82	1,082412586	0,64	0,78	0,2
OPCs	PARD3	ENSG00000148498	0,847	1,021239183	0,694	0,96	0,39
OPCs	CALCRL	ENSG00000064989	0,788	1,098418445	0,576	0,62	0,05
OPCs	LRP1	ENSG00000123384	0,822	0,995192186	0,644	0,82	0,25
OPCs	EGFR	ENSG00000146648	0,782	0,907309503	0,564	0,73	0,16
OPCs	SEMA5A	ENSG00000112902	0,888	1,439629577	0,776	0,93	0,37

OPCs	ETV1	ENSG00000006468	0,787	1,04532403	0,574	0,63	0,07
OPCs	CCSER1	ENSG00000184305	0,779	0,804425878	0,558	0,82	0,26
OPCs	PTPRT	ENSG00000196090	0,784	0,996319266	0,568	0,68	0,12
OPCs	TAFA2	ENSG00000198673	0,794	1,075021796	0,588	0,75	0,19
OPCs	PHLDA1	ENSG00000139289	0,789	1,05418305	0,578	0,61	0,05
OPCs	AFAP1L2	ENSG00000169129	0,782	0,984930048	0,564	0,6	0,05
OPCs	TMEM108	ENSG00000144868	0,805	1,036161213	0,61	0,8	0,25
OPCs	PDGFRA	ENSG00000134853	0,775	0,985623489	0,55	0,55	0
OPCs	ASIC2	ENSG00000108684	0,777	0,867705781	0,554	0,67	0,12
OPCs	MEG8	ENSG00000225746	0,766	0,810825507	0,532	0,68	0,14
OPCs	MGLL	ENSG00000074416	0,802	1,140165804	0,604	0,73	0,19
OPCs	RAMP1	ENSG00000132329	0,797	0,988554303	0,594	0,72	0,18
OPCs	FAM110B	ENSG00000169122	0,799	1,03910985	0,598	0,73	0,19
OPCs	MTSS2	ENSG00000132613	0,805	0,969738269	0,61	0,76	0,22
OPCs	GRAMD1C	ENSG00000178075	0,776	0,916970221	0,552	0,67	0,13
OPCs	XYLT1	ENSG00000103489	0,929	1,815419323	0,858	0,96	0,42
OPCs	DAB1	ENSG00000173406	0,815	0,884036724	0,63	0,89	0,36
OPCs	ASTN1	ENSG00000152092	0,804	0,976545896	0,608	0,79	0,26
OPCs	ARHGAP31	ENSG00000031081	0,766	0,814271586	0,532	0,73	0,2
OPCs	KCNMA1	ENSG00000156113	0,833	0,880628422	0,666	0,95	0,42
OPCs	CTNNA2	ENSG00000066032	0,806	0,814806999	0,612	0,96	0,43
OPCs	MARCKS	ENSG00000277443	0,807	1,068166661	0,614	0,76	0,23
OPCs	GRIK3	ENSG00000163873	0,77	0,961641663	0,54	0,61	0,09
OPCs	CCDC50	ENSG00000152492	0,829	1,056002212	0,658	0,81	0,3
OPCs	RHBDL3	ENSG00000141314	0,766	0,909360877	0,532	0,63	0,11
OPCs	OLIG1	ENSG00000184221	0,81	1,08743951	0,62	0,8	0,29
OPCs	RAPGEF4	ENSG00000091428	0,794	0,885945421	0,588	0,83	0,32
OPCs	NCALD	ENSG00000104490	0,78	1,177522359	0,56	0,67	0,16
OPCs	KCNMB2-AS1	ENSG00000237978	0,768	1,018500011	0,536	0,64	0,13
OPCs	KLF12	ENSG00000118922	0,808	1,00304597	0,616	0,84	0,33
OPCs	RGS7	ENSG00000182901	0,799	0,865352029	0,598	0,9	0,39
OPCs	OLIG2	ENSG00000205927	0,766	0,93889797	0,532	0,62	0,11
OPCs	C1orf61	ENSG00000125462	0,774	0,778845352	0,548	0,75	0,25
OPCs	PRKG2	ENSG00000138669	0,761	1,004005704	0,522	0,56	0,05
OPCs	CMYA5	ENSG00000164309	0,762	0,917293389	0,524	0,6	0,1
OPCs	GRID2	ENSG00000152208	0,885	1,184504473	0,77	0,98	0,48
OPCs	KHDRBS3	ENSG00000131773	0,833	1,154604814	0,666	0,84	0,34
OPCs	TNS3	ENSG00000136205	0,758	0,81484995	0,516	0,69	0,19
OPCs	LRRC7	ENSG00000033122	0,747	0,658071167	0,494	0,79	0,29
OPCs	SLC44A5	ENSG00000137968	0,752	0,900278436	0,504	0,63	0,13
OPCs	KCNT2	ENSG00000162687	0,751	0,83377697	0,502	0,61	0,11
OPCs	BEST3	ENSG00000127325	0,747	1,037704116	0,494	0,5	0
OPCs	GRIA3	ENSG00000125675	0,797	0,887793682	0,594	0,81	0,32
OPCs	ITGA9	ENSG00000144668	0,758	0,932852898	0,516	0,61	0,11
OPCs	PALLD	ENSG00000129116	0,769	0,875110907	0,538	0,69	0,2
OPCs	NTNG1	ENSG00000162631	0,745	0,744022542	0,49	0,66	0,17
OPCs	CST3	ENSG00000101439	0,778	0,919144394	0,556	0,81	0,32
OPCs	SERPINE2	ENSG00000135919	0,751	0,715407613	0,502	0,67	0,18
OPCs	LRRTM3	ENSG00000198739	0,826	1,101004242	0,652	0,87	0,38
OPCs	ARHGAP10	ENSG00000071205	0,746	0,771955524	0,492	0,59	0,11
OPCs	ARHGAP42	ENSG00000165895	0,738	0,677719093	0,476	0,61	0,13
OPCs	KCND3	ENSG00000171385	0,761	0,889806597	0,522	0,66	0,18
OPCs	IL1RAP	ENSG00000196083	0,746	0,862711327	0,492	0,58	0,1
OPCs	ARHGEF4	ENSG00000136002	0,77	0,697066794	0,54	0,77	0,29
OPCs	AL133346.1	ENSG00000227220	0,752	1,044589996	0,504	0,58	0,1

OPCs	GALNT13	ENSG00000144278	0,911	1,529046966	0,822	0,96	0,48
OPCs	SCN2A	ENSG00000136531	0,735	0,681974115	0,47	0,66	0,18
OPCs	LINC02223	ENSG00000249937	0,747	1,027435167	0,494	0,55	0,07
OPCs	GUCY1A2	ENSG00000152402	0,741	0,733460701	0,482	0,66	0,18
OPCs	RIT2	ENSG00000152214	0,741	0,937406621	0,482	0,56	0,09
OPCs	AFAP1	ENSG00000196526	0,756	0,94891714	0,512	0,58	0,11
OPCs	RUNX1T1	ENSG00000079102	0,747	0,742829283	0,494	0,74	0,27
OPCs	RALYL	ENSG00000184672	0,743	0,924897331	0,486	0,71	0,24
OPCs	RIMS1	ENSG00000079841	0,739	0,658577654	0,478	0,73	0,26
OPCs	LRRC4C	ENSG00000148948	0,985	2,346357992	0,97	1	0,53
OPCs	NLGN1	ENSG00000169760	0,949	1,582617007	0,898	1	0,53
OPCs	CABLES1	ENSG00000134508	0,733	0,788176446	0,466	0,61	0,14
OPCs	ABHD2	ENSG00000140526	0,812	1,025709429	0,624	0,81	0,35
OPCs	GRIA4	ENSG00000152578	0,949	1,720371038	0,898	0,99	0,52
OPCs	CD82	ENSG00000085117	0,738	0,786327001	0,476	0,58	0,12
OPCs	SYT17	ENSG00000103528	0,734	0,660504826	0,468	0,65	0,18
OPCs	DGKI	ENSG00000157680	0,783	0,822271157	0,566	0,84	0,38
OPCs	SLC1A1	ENSG00000106688	0,745	0,802968893	0,49	0,59	0,13
OPCs	ASCL1	ENSG00000139352	0,734	0,84091111	0,468	0,48	0,01
OPCs	THSD7A	ENSG00000005108	0,743	0,694299155	0,486	0,72	0,26
OPCs	KCNAB1	ENSG00000169282	0,739	0,744151111	0,478	0,66	0,2
OPCs	ATCAY	ENSG00000167654	0,744	0,785063813	0,488	0,58	0,12
OPCs	TRIM9	ENSG00000100505	0,879	1,22641127	0,758	0,95	0,49
OPCs	ATP2B4	ENSG00000058668	0,815	0,955196664	0,63	0,86	0,41
OPCs	TACC2	ENSG00000138162	0,75	0,835339484	0,5	0,59	0,14
OPCs	SPSB4	ENSG00000175093	0,73	0,839250929	0,46	0,47	0,02
OPCs	ADARB2	ENSG00000185736	0,874	1,16177107	0,748	0,95	0,5
OPCs	SLC2A13	ENSG00000151229	0,722	0,622069041	0,444	0,67	0,22
OPCs	SAMHD1	ENSG00000101347	0,742	0,816994951	0,484	0,64	0,19
OPCs	NKAIN3	ENSG00000185942	0,722	0,708987914	0,444	0,64	0,19
OPCs	FIGN	ENSG00000182263	0,738	0,670187833	0,476	0,78	0,34
OPCs	BMP7	ENSG00000101144	0,737	0,735869776	0,474	0,6	0,15
OPCs	SGCZ	ENSG00000185053	0,722	0,85556654	0,444	0,61	0,17
OPCs	ST8SIA1	ENSG00000111728	0,739	0,79390635	0,478	0,67	0,23
OPCs	SEMA3D	ENSG00000153993	0,725	0,826621036	0,45	0,5	0,06
OPCs	CDK14	ENSG00000058091	0,778	0,840905568	0,556	0,82	0,38
OPCs	AL445250.1	ENSG00000225096	0,749	1,140350013	0,498	0,65	0,21
OPCs	LMO3	ENSG00000048540	0,724	0,753352241	0,448	0,56	0,13
OPCs	CSPG5	ENSG00000114646	0,732	0,85367895	0,464	0,52	0,08
OPCs	NFIB	ENSG00000147862	0,775	0,696631397	0,55	0,92	0,49
OPCs	RORA	ENSG00000069667	0,829	0,880220684	0,658	0,98	0,55
OPCs	TMEM132B	ENSG00000139364	0,727	0,788436254	0,454	0,59	0,15
OPCs	ATP2C2	ENSG00000064270	0,72	0,823421301	0,44	0,46	0,03
OPCs	COL4A3	ENSG00000169031	0,72	0,781138628	0,44	0,5	0,07
OPCs	GFRA1	ENSG00000151892	0,721	0,856195829	0,442	0,47	0,04
OPCs	PRKD1	ENSG00000184304	0,757	0,780366365	0,514	0,81	0,38
OPCs	TNIK	ENSG00000154310	0,763	0,625519204	0,526	0,91	0,48
OPCs	ADCY1	ENSG00000164742	0,726	0,760362433	0,452	0,57	0,14
OPCs	C1QL1	ENSG00000131094	0,716	0,899952217	0,432	0,45	0,02
OPCs	PLPPR1	ENSG00000148123	0,748	0,81362709	0,496	0,73	0,31
OPCs	GNPTAB	ENSG00000111670	0,75	0,838008734	0,5	0,66	0,23
OPCs	THRB	ENSG00000151090	0,756	0,823048072	0,512	0,77	0,34
OPCs	LRFN5	ENSG00000165379	0,701	0,600787062	0,402	0,6	0,17
OPCs	ITGA8	ENSG00000077943	0,713	0,942861017	0,426	0,46	0,04
OPCs	SNAP91	ENSG00000065609	0,716	0,593147336	0,432	0,73	0,31

OPCs	GPR37L1	ENSG00000170075	0,72	0,685725127	0,44	0,6	0,18
OPCs	SPRED2	ENSG00000198369	0,737	0,77870847	0,474	0,68	0,26
OPCs	GRIA2	ENSG00000120251	0,867	1,06819506	0,734	0,95	0,54
OPCs	TENM1	ENSG00000009694	0,706	0,61536445	0,412	0,54	0,13
OPCs	PTN	ENSG00000105894	0,734	0,695298192	0,468	0,72	0,31
OPCs	DZIP1	ENSG00000134874	0,725	0,689010963	0,45	0,59	0,18
OPCs	WSCD1	ENSG00000179314	0,728	0,803124625	0,456	0,56	0,15
OPCs	PLAAT1	ENSG00000127252	0,711	0,843524196	0,422	0,45	0,04
OPCs	NSG2	ENSG00000170091	0,71	0,679638028	0,42	0,53	0,12
OPCs	KANK1	ENSG00000107104	0,757	0,673759464	0,514	0,87	0,47
OPCs	VIPR2	ENSG00000106018	0,703	0,810125069	0,406	0,41	0,01
OPCs	GLCCI1	ENSG00000106415	0,77	0,923507852	0,54	0,77	0,37
OPCs	FGFR1	ENSG00000077782	0,732	0,749303155	0,464	0,65	0,25
OPCs	ADRA1A	ENSG00000120907	0,704	0,740807847	0,408	0,5	0,1
OPCs	GPC6	ENSG00000183098	0,705	0,73167646	0,41	0,59	0,19
OPCs	OLFM2	ENSG00000105088	0,71	0,682123531	0,42	0,54	0,14
OPCs	CSRNP3	ENSG00000178662	0,739	0,718604448	0,478	0,77	0,37
OPCs	SNX22	ENSG00000157734	0,705	0,73768128	0,41	0,47	0,07
OPCs	MAP3K1	ENSG00000095015	0,715	0,736549372	0,43	0,59	0,19
OPCs	HS3ST1	ENSG00000002587	0,702	0,783310883	0,404	0,43	0,03
OPCs	ANO6	ENSG00000177119	0,712	0,665111447	0,424	0,61	0,22
OPCs	SDC3	ENSG00000162512	0,71	0,703737745	0,42	0,53	0,13
OPCs	DPY19L2	ENSG00000177990	0,708	0,650843319	0,416	0,55	0,15
OPCs	ZNF462	ENSG00000148143	0,844	1,109162412	0,688	0,9	0,51
OPCs	CPNE5	ENSG00000124772	0,704	0,726147952	0,408	0,47	0,08
OPCs	APOD	ENSG00000189058	0,763	0,868344521	0,526	0,85	0,46
OPCs	ILDR2	ENSG00000143195	0,708	0,689745852	0,416	0,51	0,12
OPCs	GPR158	ENSG00000151025	0,721	0,776154644	0,442	0,68	0,3
OPCs	HIP1R	ENSG00000130787	0,823	0,952432072	0,646	0,9	0,51
OPCs	BAALC	ENSG00000164929	0,703	0,657681218	0,406	0,58	0,19
OPCs	RXRA	ENSG00000186350	0,71	0,685552913	0,42	0,56	0,17
OPCs	SOX8	ENSG00000005513	0,716	0,730776654	0,432	0,63	0,24
OPCs	U91319.1	ENSG00000262801	0,702	0,935875385	0,404	0,47	0,09
OPCs	PLPPR5	ENSG00000117598	0,701	0,719726546	0,402	0,46	0,08
OPCs	GRIK4	ENSG00000149403	0,709	0,623965478	0,418	0,69	0,33
OPCs	USP24	ENSG00000162402	0,822	0,97147449	0,644	0,88	0,52
OPCs	CTNND2	ENSG00000169862	0,797	0,68155909	0,594	0,98	0,62
OPCs	SGCD	ENSG00000170624	0,74	0,796069144	0,48	0,81	0,45
OPCs	CASK	ENSG00000147044	0,732	0,666974521	0,464	0,79	0,44
OPCs	ASTN2	ENSG00000148219	0,789	0,766109164	0,578	0,94	0,59
OPCs	SAMD4A	ENSG00000020577	0,727	0,619420455	0,454	0,82	0,48
OPCs	CYFIP2	ENSG00000055163	0,704	0,623097362	0,408	0,65	0,3
OPCs	KAT2B	ENSG00000114166	0,877	1,161821727	0,754	0,95	0,61
OPCs	DNER	ENSG00000187957	0,787	0,759458751	0,574	0,91	0,57
OPCs	DPP6	ENSG00000130226	0,951	1,52357438	0,902	0,99	0,66
OPCs	ADAM22	ENSG00000008277	0,704	0,587166521	0,408	0,72	0,38
OPCs	TRIO	ENSG00000038382	0,826	0,910052656	0,652	0,95	0,62
OPCs	DMD	ENSG00000198947	0,811	0,902773478	0,622	0,94	0,61
OPCs	NBEA	ENSG00000172915	0,764	0,652546936	0,528	0,9	0,58
OPCs	NAV1	ENSG00000134369	0,868	1,137041711	0,736	0,95	0,63
OPCs	KAZN	ENSG00000189337	0,833	0,934025237	0,666	0,98	0,65
OPCs	RIN2	ENSG00000132669	0,73	0,672652446	0,46	0,81	0,49
OPCs	NOVA1	ENSG00000139910	0,899	1,256159349	0,798	0,97	0,66
OPCs	MTSS1	ENSG00000170873	0,745	0,689289718	0,49	0,85	0,54
OPCs	PIK3R1	ENSG00000145675	0,832	1,008605363	0,664	0,92	0,61

OPCs	HIP1	ENSG00000127946	0,788	0,862751612	0,576	0,93	0,63
OPCs	ZDHHC14	ENSG00000175048	0,807	0,898773308	0,614	0,93	0,62
OPCs	MAML2	ENSG00000184384	0,749	0,597160805	0,498	0,94	0,63
OPCs	PHYHIPL	ENSG00000165443	0,738	0,678912588	0,476	0,85	0,55
OPCs	ARNT2	ENSG00000172379	0,726	0,616769085	0,452	0,81	0,51
OPCs	ERBB4	ENSG00000178568	0,83	0,864088932	0,66	0,99	0,7
OPCs	EPN2	ENSG00000072134	0,895	1,359761412	0,79	0,96	0,68
OPCs	NAV2	ENSG00000166833	0,788	0,777345358	0,576	0,96	0,68
OPCs	NLGN4Y	ENSG00000165246	0,74	1,030594788	0,48	0,74	0,46
OPCs	PHACTR3	ENSG00000087495	0,744	0,65857457	0,488	0,91	0,65
OPCs	ASAP1	ENSG00000153317	0,787	0,745337837	0,574	0,94	0,69
OPCs	KIF13A	ENSG00000137177	0,814	0,929573604	0,628	0,94	0,7
OPCs	SCD5	ENSG00000145284	0,84	0,89403382	0,68	0,98	0,74
OPCs	PRKCA	ENSG00000154229	0,829	0,82299835	0,658	0,98	0,75
OPCs	ADGRL3	ENSG00000150471	0,893	1,030822425	0,786	0,99	0,77
OPCs	LSAMP	ENSG00000185565	0,851	0,930199912	0,702	1	0,78
OPCs	ZHX2	ENSG00000178764	0,713	0,609677772	0,426	0,87	0,65
OPCs	LRP1B	ENSG00000168702	0,909	1,137687638	0,818	0,99	0,78
OPCs	ADGRB3	ENSG00000135298	0,821	0,76188164	0,642	0,99	0,8
OPCs	ZSWIM6	ENSG00000130449	0,737	0,593003858	0,474	0,97	0,88
Astrocytes	OBI1-AS1	ENSG00000234377	0,951	2,736114088	0,902	0,91	0,05
Astrocytes	RFX4	ENSG00000111783	0,955	2,396612762	0,91	0,92	0,06
Astrocytes	HPSE2	ENSG00000172987	0,959	3,328073605	0,918	0,93	0,07
Astrocytes	SLC14A1	ENSG00000141469	0,936	2,627401325	0,872	0,88	0,03
Astrocytes	AQP4	ENSG00000171885	0,934	2,198522936	0,868	0,88	0,06
Astrocytes	RYR3	ENSG00000198838	0,963	2,49723103	0,926	0,95	0,13
Astrocytes	TRPM3	ENSG00000083067	0,986	2,941241796	0,972	0,99	0,18
Astrocytes	LINC01088	ENSG00000249307	0,953	2,814103486	0,906	0,93	0,12
Astrocytes	ATP1A2	ENSG00000018625	0,934	1,784140078	0,868	0,93	0,13
Astrocytes	ADCY2	ENSG00000078295	0,968	2,416138821	0,936	0,96	0,16
Astrocytes	TNC	ENSG00000041982	0,893	2,284663774	0,786	0,79	0,01
Astrocytes	GLIS3	ENSG00000107249	0,95	2,248201841	0,9	0,95	0,17
Astrocytes	HIF3A	ENSG00000124440	0,92	1,757852621	0,84	0,93	0,15
Astrocytes	MAPK4	ENSG00000141639	0,913	2,068757784	0,826	0,85	0,08
Astrocytes	GJA1	ENSG00000152661	0,896	1,944073055	0,792	0,82	0,06
Astrocytes	RGMA	ENSG00000182175	0,893	1,798417354	0,786	0,8	0,05
Astrocytes	GPC5	ENSG00000179399	0,939	2,311162187	0,878	0,95	0,21
Astrocytes	FAM189A2	ENSG00000135063	0,891	1,938869812	0,782	0,8	0,06
Astrocytes	SDC4	ENSG00000124145	0,872	1,714830508	0,744	0,76	0,03
Astrocytes	WDR49	ENSG00000174776	0,867	1,623923044	0,734	0,77	0,04
Astrocytes	PTCHD1-AS	ENSG00000233067	0,89	1,998057609	0,78	0,83	0,11
Astrocytes	SLC4A4	ENSG00000080493	0,88	1,95809425	0,76	0,79	0,07
Astrocytes	PTPRZ1	ENSG00000106278	0,865	1,045668638	0,73	0,89	0,17
Astrocytes	EFEMP1	ENSG00000115380	0,862	1,697939471	0,724	0,74	0,03
Astrocytes	SLC25A18	ENSG00000182902	0,884	1,723803384	0,768	0,8	0,09
Astrocytes	SLC1A2	ENSG00000110436	0,914	2,339001231	0,828	0,89	0,19
Astrocytes	MGST1	ENSG00000008394	0,866	1,686291455	0,732	0,76	0,06
Astrocytes	AC073941.1	ENSG00000259255	0,852	1,900075379	0,704	0,71	0,01
Astrocytes	BMPR1B	ENSG00000138696	0,859	1,635364897	0,718	0,74	0,05
Astrocytes	GPM6A	ENSG00000150625	0,929	1,828722317	0,858	0,97	0,27
Astrocytes	SOX5	ENSG00000134532	0,919	1,667328543	0,838	0,97	0,28
Astrocytes	SPON1	ENSG00000262655	0,865	1,635082724	0,73	0,79	0,1
Astrocytes	PAMR1	ENSG00000149090	0,851	1,605223282	0,702	0,72	0,03
Astrocytes	FMN2	ENSG00000155816	0,951	1,97231427	0,902	0,95	0,27
Astrocytes	MGAT4C	ENSG00000182050	0,851	1,366257916	0,702	0,81	0,13

Astrocytes	NRG3	ENSG00000185737	0,963	2,082791891	0,926	1	0,32
Astrocytes	NEBL	ENSG00000078114	0,936	1,784006973	0,872	0,97	0,3
Astrocytes	RANBP3L	ENSG00000164188	0,846	1,826604486	0,692	0,71	0,04
Astrocytes	GFAP	ENSG00000131095	0,93	2,178838532	0,86	0,93	0,26
Astrocytes	PITPNC1	ENSG00000154217	0,965	2,251227336	0,93	0,98	0,31
Astrocytes	ADCY8	ENSG00000155897	0,872	1,634481984	0,744	0,81	0,14
Astrocytes	KCNN3	ENSG00000143603	0,884	1,734348874	0,768	0,82	0,15
Astrocytes	SLC24A4	ENSG00000140090	0,849	1,634306229	0,698	0,72	0,06
Astrocytes	CNTN1	ENSG00000018236	0,875	1,385205023	0,75	0,94	0,27
Astrocytes	ABLIM1	ENSG00000099204	0,901	1,620993618	0,802	0,89	0,24
Astrocytes	APOE	ENSG00000130203	0,881	1,663126404	0,762	0,89	0,24
Astrocytes	ANKFN1	ENSG00000153930	0,853	1,474960654	0,706	0,78	0,13
Astrocytes	TSHZ2	ENSG00000182463	0,843	1,484877038	0,686	0,78	0,13
Astrocytes	NTRK3	ENSG00000140538	0,865	1,311641617	0,73	0,89	0,24
Astrocytes	ACSBG1	ENSG00000103740	0,843	1,370814277	0,686	0,78	0,14
Astrocytes	ACOT11	ENSG00000162390	0,86	1,512399423	0,72	0,78	0,14
Astrocytes	NRCAM	ENSG00000091129	0,834	0,904096303	0,668	0,89	0,25
Astrocytes	EYA2	ENSG00000064655	0,833	1,47256884	0,666	0,7	0,06
Astrocytes	SORBS1	ENSG00000095637	0,968	2,187876208	0,936	0,98	0,35
Astrocytes	BOC	ENSG00000144857	0,831	1,511641789	0,662	0,69	0,05
Astrocytes	AC092691.1	ENSG00000239268	0,871	1,377153617	0,742	0,93	0,3
Astrocytes	WWC1	ENSG00000113645	0,839	1,511462404	0,678	0,72	0,09
Astrocytes	CARMIL1	ENSG00000079691	0,856	1,59462575	0,712	0,79	0,16
Astrocytes	STK32A	ENSG00000169302	0,83	1,434924529	0,66	0,73	0,11
Astrocytes	NHSL1	ENSG00000135540	0,826	1,132918485	0,652	0,77	0,14
Astrocytes	FGFR3	ENSG00000068078	0,822	1,476616242	0,644	0,67	0,05
Astrocytes	FAT3	ENSG00000165323	0,839	1,46916525	0,678	0,78	0,16
Astrocytes	NKAIN3	ENSG00000185942	0,837	1,480427525	0,674	0,77	0,15
Astrocytes	CD44	ENSG00000026508	0,828	1,969816778	0,656	0,69	0,08
Astrocytes	PLCB1	ENSG00000182621	0,855	1,337713073	0,71	0,9	0,28
Astrocytes	ATP1B2	ENSG00000129244	0,845	1,504547461	0,69	0,76	0,14
Astrocytes	SERPINI2	ENSG00000114204	0,81	1,324252675	0,62	0,64	0,03
Astrocytes	ARHGEF4	ENSG00000136002	0,892	1,664189467	0,784	0,86	0,25
Astrocytes	AQP1	ENSG00000240583	0,811	1,842175799	0,622	0,64	0,03
Astrocytes	RGS6	ENSG00000182732	0,823	1,571644835	0,646	0,75	0,14
Astrocytes	PARD3	ENSG00000148498	0,919	1,603329353	0,838	0,97	0,36
Astrocytes	GRAMD2B	ENSG00000155324	0,886	1,523491372	0,772	0,88	0,27
Astrocytes	SLC7A11	ENSG00000151012	0,843	1,560308875	0,686	0,77	0,17
Astrocytes	DGKG	ENSG00000058866	0,831	1,211901226	0,662	0,82	0,22
Astrocytes	CDHR3	ENSG00000128536	0,82	1,373431355	0,64	0,7	0,1
Astrocytes	IQCA1	ENSG00000132321	0,83	1,524617771	0,66	0,72	0,12
Astrocytes	ITGB4	ENSG00000132470	0,81	1,35730368	0,62	0,66	0,07
Astrocytes	WLS	ENSG00000116729	0,827	1,324632322	0,654	0,73	0,14
Astrocytes	ALDH1L1	ENSG00000144908	0,805	1,323376211	0,61	0,64	0,05
Astrocytes	ADGRA3	ENSG00000152990	0,82	1,352441016	0,64	0,69	0,1
Astrocytes	NPL	ENSG00000135838	0,849	1,499434483	0,698	0,79	0,2
Astrocytes	SOX6	ENSG00000110693	0,792	0,797108775	0,584	0,76	0,17
Astrocytes	PRKG1	ENSG00000185532	0,802	1,078190314	0,604	0,78	0,2
Astrocytes	MAP3K5	ENSG00000197442	0,853	1,334536512	0,706	0,85	0,28
Astrocytes	SLC6A11	ENSG00000132164	0,805	1,661729589	0,61	0,64	0,07
Astrocytes	UTRN	ENSG00000152818	0,806	0,968274077	0,612	0,84	0,26
Astrocytes	SPARCL1	ENSG00000152583	0,814	1,242558849	0,628	0,79	0,22
Astrocytes	SERPINE2	ENSG00000135919	0,82	1,353579634	0,64	0,72	0,15
Astrocytes	RFX2	ENSG00000087903	0,808	1,266469458	0,616	0,69	0,12
Astrocytes	ACACB	ENSG00000076555	0,859	1,538639475	0,718	0,81	0,25

Astrocytes	LGI1	ENSG00000108231	0,81	1,394567011	0,62	0,67	0,11
Astrocytes	CPE	ENSG00000109472	0,876	1,497618354	0,752	0,87	0,3
Astrocytes	COL5A3	ENSG00000080573	0,785	1,39538187	0,57	0,61	0,05
Astrocytes	ARHGEF26	ENSG00000114790	0,807	1,303460832	0,614	0,67	0,11
Astrocytes	CAMK2G	ENSG00000148660	0,848	1,492643191	0,696	0,79	0,23
Astrocytes	LHFPL6	ENSG00000183722	0,856	1,386920828	0,712	0,88	0,33
Astrocytes	CTNNA2	ENSG00000066032	0,869	1,37871502	0,738	0,96	0,41
Astrocytes	DCLK2	ENSG00000170390	0,889	1,611444284	0,778	0,88	0,33
Astrocytes	MED12L	ENSG00000144893	0,826	1,356830241	0,652	0,77	0,22
Astrocytes	ATP13A4	ENSG00000127249	0,808	1,191663774	0,616	0,73	0,18
Astrocytes	GMPR	ENSG00000137198	0,781	1,24448954	0,562	0,59	0,05
Astrocytes	EGFR	ENSG00000146648	0,784	1,155428618	0,568	0,68	0,14
Astrocytes	LINC01748	ENSG00000226476	0,776	1,304526178	0,552	0,57	0,02
Astrocytes	GLI3	ENSG00000106571	0,777	1,317242984	0,554	0,58	0,03
Astrocytes	FRMPD2	ENSG00000170324	0,771	1,127254621	0,542	0,57	0,02
Astrocytes	PRRX1	ENSG00000116132	0,777	1,238466155	0,554	0,62	0,08
Astrocytes	NTRK2	ENSG00000148053	0,941	1,78894997	0,882	0,97	0,43
Astrocytes	AHCYL1	ENSG00000168710	0,939	1,898526446	0,878	0,93	0,39
Astrocytes	PCDH7	ENSG00000169851	0,752	0,66849013	0,504	0,77	0,23
Astrocytes	YAP1	ENSG00000137693	0,774	1,094831828	0,548	0,61	0,07
Astrocytes	CACNA2D3	ENSG00000157445	0,811	1,562850759	0,622	0,77	0,24
Astrocytes	CACHD1	ENSG00000158966	0,798	1,208384168	0,596	0,71	0,17
Astrocytes	ETNPPL	ENSG00000164089	0,77	1,314193124	0,54	0,55	0,01
Astrocytes	NPAS2	ENSG00000170485	0,8	1,301360439	0,6	0,72	0,18
Astrocytes	GABRB1	ENSG00000163288	0,78	1,082387962	0,56	0,74	0,21
Astrocytes	PCSK5	ENSG00000099139	0,791	1,221497402	0,582	0,68	0,15
Astrocytes	BCL6	ENSG00000113916	0,844	1,411112451	0,688	0,81	0,28
Astrocytes	TCF7L1	ENSG00000152284	0,771	1,177806378	0,542	0,59	0,06
Astrocytes	KALRN	ENSG00000160145	0,86	1,312482901	0,72	0,9	0,37
Astrocytes	LRIG1	ENSG00000144749	0,853	1,541096098	0,706	0,81	0,28
Astrocytes	C1orf61	ENSG00000125462	0,82	1,368678963	0,64	0,75	0,22
Astrocytes	ABCA1	ENSG00000165029	0,828	1,38256521	0,656	0,79	0,26
Astrocytes	ADAMTS9-AS2	ENSG00000241684	0,772	1,249401605	0,544	0,64	0,12
Astrocytes	PDE7B	ENSG00000171408	0,784	1,054438899	0,568	0,73	0,21
Astrocytes	LINC00299	ENSG00000236790	0,767	1,290589335	0,534	0,55	0,03
Astrocytes	MRVI1	ENSG00000072952	0,764	1,271008346	0,528	0,54	0,02
Astrocytes	AGT	ENSG00000135744	0,771	1,257201718	0,542	0,59	0,07
Astrocytes	MCC	ENSG00000171444	0,786	1,122086602	0,572	0,69	0,17
Astrocytes	COL27A1	ENSG00000196739	0,772	1,308038973	0,544	0,6	0,08
Astrocytes	LAMA1	ENSG00000101680	0,764	1,160199521	0,528	0,55	0,03
Astrocytes	NR2F1-AS1	ENSG00000237187	0,782	1,132506005	0,564	0,67	0,15
Astrocytes	CABLES1	ENSG00000134508	0,781	1,714689351	0,562	0,63	0,11
Astrocytes	RASL12	ENSG00000103710	0,759	1,126040051	0,518	0,53	0,02
Astrocytes	PRDM16	ENSG00000142611	0,761	1,126990913	0,522	0,55	0,04
Astrocytes	PSD2	ENSG00000146005	0,782	1,172829678	0,564	0,63	0,12
Astrocytes	SFXN5	ENSG00000144040	0,851	1,416851492	0,702	0,81	0,3
Astrocytes	NRXN1	ENSG00000179915	0,771	0,822554189	0,542	0,89	0,39
Astrocytes	EYA1	ENSG00000104313	0,752	1,182442959	0,504	0,55	0,05
Astrocytes	ACSS3	ENSG00000111058	0,759	1,142241679	0,518	0,57	0,07
Astrocytes	ZNRF3	ENSG00000183579	0,798	1,258796473	0,596	0,71	0,21
Astrocytes	PHF21B	ENSG00000056487	0,764	1,109693225	0,528	0,6	0,11
Astrocytes	LUZP2	ENSG00000187398	0,751	0,906738883	0,502	0,7	0,21
Astrocytes	GRAMD1C	ENSG00000178075	0,761	1,121926444	0,522	0,6	0,11
Astrocytes	OGFRL1	ENSG00000119900	0,801	1,180112982	0,602	0,75	0,26
Astrocytes	SYTL4	ENSG00000102362	0,758	1,094126304	0,516	0,57	0,08

Astrocytes	ATP2B2	ENSG00000157087	0,766	1,050660963	0,532	0,67	0,18
Astrocytes	CSGALNACT1	ENSG00000147408	0,744	0,709422674	0,488	0,72	0,23
Astrocytes	TOGARAM2	ENSG00000189350	0,75	1,13395865	0,5	0,55	0,06
Astrocytes	PBXIP1	ENSG00000163346	0,751	1,063078889	0,502	0,54	0,06
Astrocytes	DAPK1	ENSG00000196730	0,766	1,107702638	0,532	0,62	0,15
Astrocytes	ZFP36L1	ENSG00000185650	0,742	0,832891104	0,484	0,66	0,18
Astrocytes	BCAR3	ENSG00000137936	0,767	1,102746247	0,534	0,64	0,17
Astrocytes	EDNRB	ENSG00000136160	0,741	1,08599258	0,482	0,5	0,03
Astrocytes	TNIK	ENSG00000154310	0,874	1,383844992	0,748	0,93	0,46
Astrocytes	DOCK7	ENSG00000116641	0,841	1,312862152	0,682	0,81	0,34
Astrocytes	RORA	ENSG00000069667	0,923	1,576514852	0,846	0,99	0,52
Astrocytes	FARP1	ENSG00000152767	0,774	1,051990659	0,548	0,71	0,24
Astrocytes	KIAA1671	ENSG00000197077	0,763	1,081945016	0,526	0,62	0,15
Astrocytes	SLC39A12	ENSG00000148482	0,736	1,15099676	0,472	0,49	0,02
Astrocytes	SOX9	ENSG00000125398	0,74	1,073078401	0,48	0,52	0,05
Astrocytes	BAALC	ENSG00000164929	0,762	1,157282085	0,524	0,63	0,17
Astrocytes	ITPKB	ENSG00000143772	0,885	1,393128482	0,77	0,92	0,45
Astrocytes	MARCH3	ENSG00000173926	0,757	1,164860916	0,514	0,65	0,19
Astrocytes	AQP4-AS1	ENSG00000260372	0,746	1,449654037	0,492	0,54	0,08
Astrocytes	LINC00461	ENSG00000245526	0,786	1,128906359	0,572	0,72	0,25
Astrocytes	OPHN1	ENSG00000079482	0,788	0,980905679	0,576	0,78	0,33
Astrocytes	PHYHD1	ENSG00000175287	0,731	1,061904599	0,462	0,48	0,02
Astrocytes	CHL1	ENSG00000134121	0,727	0,686653796	0,454	0,67	0,22
Astrocytes	ARHGAP26	ENSG00000145819	0,787	0,709034397	0,574	0,89	0,44
Astrocytes	MIR4300HG	ENSG00000245832	0,75	1,50402946	0,5	0,57	0,12
Astrocytes	ARHGEF10L	ENSG00000074964	0,763	1,094574406	0,526	0,63	0,18
Astrocytes	AC099792.1	ENSG00000231252	0,729	1,127280698	0,458	0,47	0,02
Astrocytes	PTCHD1	ENSG00000165186	0,737	1,088115754	0,474	0,51	0,06
Astrocytes	RBPMS	ENSG00000157110	0,726	0,919378042	0,452	0,53	0,08
Astrocytes	CLU	ENSG00000120885	0,929	2,018319861	0,858	0,95	0,5
Astrocytes	ECHDC2	ENSG00000121310	0,771	1,075353371	0,542	0,66	0,21
Astrocytes	IGFBP7	ENSG00000163453	0,719	0,750070547	0,438	0,55	0,1
Astrocytes	CDH23	ENSG00000107736	0,739	1,150025564	0,478	0,55	0,1
Astrocytes	COLEC12	ENSG00000158270	0,726	0,98154504	0,452	0,52	0,07
Astrocytes	PLEKHA5	ENSG00000052126	0,809	1,28757527	0,618	0,83	0,39
Astrocytes	C1orf21	ENSG00000116667	0,75	1,025868482	0,5	0,64	0,19
Astrocytes	DNAH7	ENSG00000118997	0,733	1,043529881	0,466	0,53	0,08
Astrocytes	FGF2	ENSG00000138685	0,745	1,038473696	0,49	0,56	0,12
Astrocytes	FLRT2	ENSG00000185070	0,739	1,024297385	0,478	0,61	0,17
Astrocytes	LINC01094	ENSG00000251442	0,722	1,167564215	0,444	0,5	0,06
Astrocytes	DPP10	ENSG00000175497	0,781	2,139438944	0,562	0,71	0,28
Astrocytes	SYT17	ENSG00000103528	0,735	0,909040894	0,47	0,6	0,16
Astrocytes	CHPT1	ENSG00000111666	0,78	1,118007825	0,56	0,71	0,27
Astrocytes	ABR	ENSG00000159842	0,747	0,612361948	0,494	0,74	0,31
Astrocytes	MRAS	ENSG00000158186	0,78	1,304820214	0,56	0,67	0,24
Astrocytes	CST3	ENSG00000101439	0,764	1,135807461	0,528	0,74	0,31
Astrocytes	SNTA1	ENSG00000101400	0,735	0,995597284	0,47	0,53	0,09
Astrocytes	CPAMD8	ENSG00000160111	0,721	1,566456592	0,442	0,46	0,03
Astrocytes	NWD1	ENSG00000188039	0,72	1,06713276	0,44	0,46	0,03
Astrocytes	BBOX1	ENSG00000129151	0,718	1,000317873	0,436	0,45	0,02
Astrocytes	STON2	ENSG00000140022	0,745	1,086015197	0,49	0,58	0,15
Astrocytes	DCLK1	ENSG00000133083	0,742	1,350035078	0,484	0,64	0,21
Astrocytes	MT1E	ENSG00000169715	0,723	0,982330743	0,446	0,54	0,12
Astrocytes	ARHGAP42	ENSG00000165895	0,714	0,76752919	0,428	0,53	0,11
Astrocytes	COL21A1	ENSG00000124749	0,722	1,071171067	0,444	0,5	0,08

Astrocytes	LPIN1	ENSG00000134324	0,823	1,311436582	0,646	0,78	0,36
Astrocytes	TMTC1	ENSG00000133687	0,727	0,866859669	0,454	0,62	0,2
Astrocytes	LINC00836	ENSG00000280809	0,713	1,174086461	0,426	0,43	0,01
Astrocytes	WFS1	ENSG00000109501	0,721	0,94990066	0,442	0,49	0,07
Astrocytes	MT2A	ENSG00000125148	0,769	0,989867021	0,538	0,75	0,33
Astrocytes	PLSCR4	ENSG00000114698	0,716	0,829569238	0,432	0,52	0,1
Astrocytes	ALDH1A1	ENSG00000165092	0,711	1,258211464	0,422	0,44	0,02
Astrocytes	PARD3B	ENSG00000116117	0,848	1,193626253	0,696	0,9	0,48
Astrocytes	MAOB	ENSG00000069535	0,726	1,163723155	0,452	0,52	0,1
Astrocytes	RNF19A	ENSG00000034677	0,797	1,236547037	0,594	0,75	0,33
Astrocytes	PAPLN	ENSG00000100767	0,708	0,917979386	0,416	0,42	0,01
Astrocytes	PLEC	ENSG00000178209	0,737	0,974580581	0,474	0,59	0,18
Astrocytes	CHI3L1	ENSG00000133048	0,709	1,760233603	0,418	0,45	0,05
Astrocytes	PHKA1	ENSG00000067177	0,719	0,994921707	0,438	0,49	0,08
Astrocytes	ANTXR1	ENSG00000169604	0,73	1,01871846	0,46	0,58	0,17
Astrocytes	TCF7L2	ENSG00000148737	0,777	0,946496037	0,554	0,79	0,39
Astrocytes	AL160272.2	ENSG00000285082	0,706	0,947138596	0,412	0,44	0,03
Astrocytes	ASPH	ENSG00000198363	0,811	1,15399267	0,622	0,79	0,39
Astrocytes	NTNG1	ENSG00000162631	0,706	0,677878826	0,412	0,56	0,16
Astrocytes	SLC16A9	ENSG00000165449	0,711	0,925143369	0,422	0,45	0,05
Astrocytes	ANGPTL4	ENSG00000167772	0,709	1,444962314	0,418	0,46	0,05
Astrocytes	PFKP	ENSG00000067057	0,722	1,073705831	0,444	0,51	0,11
Astrocytes	NDRG2	ENSG00000165795	0,782	1,024134897	0,564	0,81	0,41
Astrocytes	KIAA1217	ENSG00000120549	0,713	0,932646694	0,426	0,61	0,21
Astrocytes	AC027117.2	ENSG00000253944	0,732	1,006873797	0,464	0,6	0,2
Astrocytes	TIMP3	ENSG00000100234	0,705	0,693264037	0,41	0,55	0,15
Astrocytes	MTSS2	ENSG00000132613	0,732	0,893040736	0,464	0,6	0,21
Astrocytes	GPR37L1	ENSG00000170075	0,719	0,894181677	0,438	0,56	0,16
Astrocytes	STK33	ENSG00000130413	0,732	0,9078295	0,464	0,62	0,23
Astrocytes	SHROOM3	ENSG00000138771	0,706	1,04113941	0,412	0,46	0,06
Astrocytes	OSBPL11	ENSG00000144909	0,729	0,965265465	0,458	0,58	0,19
Astrocytes	NFIB	ENSG00000147862	0,765	0,775927535	0,53	0,86	0,47
Astrocytes	DDR2	ENSG00000162733	0,704	1,032656004	0,408	0,46	0,07
Astrocytes	CTNND2	ENSG00000169862	0,925	1,441852914	0,85	0,99	0,6
Astrocytes	PRKD1	ENSG00000184304	0,739	0,778243668	0,478	0,75	0,36
Astrocytes	CACNB2	ENSG00000165995	0,718	1,09978946	0,436	0,63	0,25
Astrocytes	PALLD	ENSG00000129116	0,72	0,943827207	0,44	0,57	0,19
Astrocytes	LINC00511	ENSG00000227036	0,711	0,880967681	0,422	0,61	0,23
Astrocytes	GAN	ENSG00000261609	0,744	0,980005702	0,488	0,64	0,26
Astrocytes	ZBTB7C	ENSG00000184828	0,701	0,895863201	0,402	0,48	0,11
Astrocytes	LRP1	ENSG00000123384	0,714	0,734618291	0,428	0,62	0,25
Astrocytes	MICALL2	ENSG00000164877	0,703	0,874796087	0,406	0,48	0,11
Astrocytes	TTYH1	ENSG00000167614	0,761	0,953454281	0,522	0,74	0,37
Astrocytes	ST8SIA1	ENSG00000111728	0,714	0,893384984	0,428	0,59	0,22
Astrocytes	SAMD4A	ENSG00000020577	0,804	1,302964445	0,608	0,83	0,46
Astrocytes	SPARC	ENSG00000113140	0,731	1,008286559	0,462	0,68	0,32
Astrocytes	ANKDD1A	ENSG00000166839	0,705	0,898681644	0,41	0,5	0,14
Astrocytes	RAMP1	ENSG00000132329	0,701	0,78207011	0,402	0,54	0,18
Astrocytes	FUT9	ENSG00000172461	0,813	1,017406787	0,626	0,88	0,51
Astrocytes	CTDSPL	ENSG00000144677	0,753	0,965801275	0,506	0,7	0,35
Astrocytes	NAV3	ENSG00000067798	0,782	0,773334059	0,564	0,93	0,57
Astrocytes	CNN3	ENSG00000117519	0,706	0,861572391	0,412	0,55	0,19
Astrocytes	ACSS1	ENSG00000154930	0,702	0,89607762	0,404	0,53	0,18
Astrocytes	GABBR1	ENSG00000204681	0,715	0,869592084	0,43	0,57	0,21
Astrocytes	PFKFB3	ENSG00000170525	0,735	0,868057364	0,47	0,67	0,32

Astrocytes	PDLIM5	ENSG00000163110	0,709	0,728049049	0,418	0,64	0,29
Astrocytes	DKK3	ENSG00000050165	0,708	0,833904869	0,416	0,56	0,22
Astrocytes	ACADVL	ENSG00000072778	0,722	0,933471045	0,444	0,6	0,26
Astrocytes	BCL2	ENSG00000171791	0,796	0,976107359	0,592	0,87	0,53
Astrocytes	EGLN3	ENSG00000129521	0,718	1,31622593	0,436	0,59	0,25
Astrocytes	ASTN2	ENSG00000148219	0,8	0,972645927	0,6	0,9	0,58
Astrocytes	ATP2B4	ENSG00000058668	0,726	0,771716959	0,452	0,72	0,4
Astrocytes	PALM	ENSG00000099864	0,703	0,834517498	0,406	0,56	0,25
Astrocytes	TEAD1	ENSG00000187079	0,745	0,826327977	0,49	0,76	0,45
Astrocytes	NAV2	ENSG00000166833	0,881	1,251127605	0,762	0,97	0,66
Astrocytes	PLXNB1	ENSG00000164050	0,706	0,816874155	0,412	0,63	0,33
Astrocytes	MAPK10	ENSG00000109339	0,892	1,225806367	0,784	0,96	0,66
Astrocytes	LIFR	ENSG00000113594	0,828	1,07515354	0,656	0,9	0,6
Astrocytes	SRGAP1	ENSG00000196935	0,735	0,64965647	0,47	0,8	0,5
Astrocytes	TRPS1	ENSG00000104447	0,87	1,203471496	0,74	0,94	0,64
Astrocytes	CDH20	ENSG00000101542	0,801	0,911911321	0,602	0,97	0,67
Astrocytes	ERBB4	ENSG00000178568	0,782	0,735505171	0,564	0,98	0,68
Astrocytes	APC2	ENSG00000115266	0,707	0,857403516	0,414	0,61	0,32
Astrocytes	MAML2	ENSG00000184384	0,752	0,721075865	0,504	0,91	0,62
Astrocytes	PDE4DIP	ENSG00000178104	0,897	1,308717416	0,794	0,96	0,67
Astrocytes	RHOBTB3	ENSG00000164292	0,723	0,833810741	0,446	0,7	0,42
Astrocytes	APC	ENSG00000134982	0,766	0,946905067	0,532	0,78	0,5
Astrocytes	MSI2	ENSG00000153944	0,927	1,365474652	0,854	0,98	0,7
Astrocytes	DTNA	ENSG00000134769	0,977	1,829188811	0,954	1	0,73
Astrocytes	FYN	ENSG00000010810	0,83	1,079625317	0,66	0,9	0,63
Astrocytes	BCKDHB	ENSG00000083123	0,738	0,840614427	0,476	0,76	0,5
Astrocytes	GLUD1	ENSG00000148672	0,711	0,826502511	0,422	0,66	0,4
Astrocytes	SLC39A11	ENSG00000133195	0,775	0,911307466	0,55	0,93	0,68
Astrocytes	ZHX3	ENSG00000174306	0,764	0,85024105	0,528	0,82	0,57
Astrocytes	REPS1	ENSG00000135597	0,703	0,741952139	0,406	0,66	0,41
Astrocytes	KIAA0930	ENSG00000100364	0,703	0,673826753	0,406	0,73	0,49
Astrocytes	CLEC16A	ENSG00000038532	0,753	0,913479465	0,506	0,79	0,55
Astrocytes	ARNT2	ENSG00000172379	0,709	0,713087418	0,418	0,74	0,5
Astrocytes	LSAMP	ENSG00000185565	0,928	1,397229682	0,856	1	0,77
Astrocytes	CKB	ENSG00000166165	0,715	0,785043969	0,43	0,76	0,54
Astrocytes	SASH1	ENSG00000111961	0,784	0,863777122	0,568	0,92	0,7
Astrocytes	TRAK1	ENSG00000182606	0,706	0,73562172	0,412	0,71	0,48
Astrocytes	LRP1B	ENSG00000168702	0,818	0,848525891	0,636	0,99	0,77
Astrocytes	PRKCA	ENSG00000154229	0,816	0,922006164	0,632	0,95	0,74
Astrocytes	REV3L	ENSG00000009413	0,73	0,77201734	0,46	0,81	0,62
Astrocytes	NPAS3	ENSG00000151322	0,845	0,874976655	0,69	0,99	0,82
Astrocytes	MIR99AHG	ENSG00000215386	0,892	1,156373342	0,784	0,98	0,8
Astrocytes	NFIA	ENSG00000162599	0,828	0,876962256	0,656	0,97	0,8
Astrocytes	TNRC6A	ENSG00000090905	0,856	0,978881535	0,712	0,97	0,82
Astrocytes	SSBP2	ENSG00000145687	0,737	0,707813636	0,474	0,88	0,74
Astrocytes	AC245297.3	ENSG00000274265	0,702	0,698096865	0,404	0,8	0,66
Astrocytes	NEAT1	ENSG00000245532	0,806	1,070522977	0,612	1	0,87
Astrocytes	MKLN1	ENSG00000128585	0,838	0,858508804	0,676	0,96	0,87
Astrocytes	MACF1	ENSG00000127603	0,91	1,048599585	0,82	0,99	0,9
Endothelial cells	CLDN5	ENSG00000184113	0,987	3,171582047	0,974	0,98	0,04
Endothelial cells	FLT1	ENSG00000102755	0,99	3,542137792	0,98	0,99	0,06
Endothelial cells	ATP10A	ENSG00000206190	0,979	3,213763802	0,958	0,97	0,04
Endothelial cells	ABCB1	ENSG00000085563	0,983	3,329673439	0,966	0,97	0,05
Endothelial cells	VWF	ENSG00000110799	0,983	3,088698552	0,966	0,97	0,05
Endothelial cells	EPAS1	ENSG00000116016	0,979	2,717071709	0,958	0,98	0,07

Endothelial cells	ERG	ENSG00000157554	0,954	2,367403183	0,908	0,92	0,02
Endothelial cells	ANO2	ENSG00000047617	0,958	2,909174449	0,916	0,93	0,05
Endothelial cells	COBLL1	ENSG00000082438	0,951	2,511059011	0,902	0,93	0,06
Endothelial cells	ADGRL4	ENSG00000162618	0,936	2,181629371	0,872	0,88	0,02
Endothelial cells	PODXL	ENSG00000128567	0,948	2,198738402	0,896	0,91	0,05
Endothelial cells	EGFL7	ENSG00000172889	0,944	2,16155874	0,888	0,9	0,05
Endothelial cells	HLA-E	ENSG00000204592	0,964	2,301178019	0,928	0,96	0,11
Endothelial cells	IFITM3	ENSG00000142089	0,94	2,198961462	0,88	0,92	0,08
Endothelial cells	FLI1	ENSG00000151702	0,935	2,078844055	0,87	0,92	0,1
Endothelial cells	TGM2	ENSG00000198959	0,916	2,094136474	0,832	0,84	0,02
Endothelial cells	PECAM1	ENSG00000261371	0,934	2,043588868	0,868	0,89	0,07
Endothelial cells	MECOM	ENSG00000085276	0,964	2,804102615	0,928	0,95	0,13
Endothelial cells	PTPRB	ENSG00000127329	0,941	2,344455769	0,882	0,9	0,09
Endothelial cells	IFI27	ENSG00000165949	0,913	2,344182971	0,826	0,84	0,04
Endothelial cells	BTNL9	ENSG00000165810	0,907	2,033572357	0,814	0,82	0,02
Endothelial cells	SYNE2	ENSG00000054654	0,936	1,974373788	0,872	0,91	0,12
Endothelial cells	ADGRF5	ENSG00000069122	0,899	1,880431431	0,798	0,81	0,03
Endothelial cells	ARHGAP29	ENSG00000137962	0,928	1,998917035	0,856	0,9	0,11
Endothelial cells	ABCG2	ENSG00000118777	0,899	2,187659149	0,798	0,81	0,02
Endothelial cells	THSD4	ENSG00000187720	0,935	2,883039223	0,87	0,9	0,11
Endothelial cells	PRKCH	ENSG00000027075	0,928	2,047408372	0,856	0,9	0,12
Endothelial cells	A2M	ENSG00000175899	0,921	1,947483353	0,842	0,9	0,12
Endothelial cells	TMSB10	ENSG00000034510	0,965	2,667374806	0,93	0,96	0,19
Endothelial cells	ITGA1	ENSG00000213949	0,901	1,944969911	0,802	0,83	0,06
Endothelial cells	SLC7A5	ENSG00000103257	0,937	2,470558572	0,874	0,91	0,14
Endothelial cells	SLCO4A1	ENSG00000101187	0,892	1,933027003	0,784	0,8	0,03
Endothelial cells	ENG	ENSG00000106991	0,896	1,873483692	0,792	0,82	0,06
Endothelial cells	IGFBP7	ENSG00000163453	0,899	1,765797173	0,798	0,88	0,12
Endothelial cells	CMTM8	ENSG00000170293	0,88	2,006984157	0,76	0,77	0,03
Endothelial cells	CGNL1	ENSG00000128849	0,891	1,817559967	0,782	0,83	0,09
Endothelial cells	CD34	ENSG00000174059	0,871	1,797393396	0,742	0,75	0,01
Endothelial cells	ST8SIA6	ENSG00000148488	0,884	1,977330008	0,768	0,79	0,06
Endothelial cells	SLC2A1	ENSG00000117394	0,915	2,027083075	0,83	0,87	0,14
Endothelial cells	TAGLN2	ENSG00000158710	0,879	1,696329337	0,758	0,79	0,07
Endothelial cells	TBC1D4	ENSG00000136111	0,93	2,123052116	0,86	0,91	0,18
Endothelial cells	HLA-B	ENSG00000234745	0,898	1,809352509	0,796	0,86	0,14
Endothelial cells	ITM2A	ENSG00000078596	0,872	1,893568732	0,744	0,77	0,05
Endothelial cells	IFITM2	ENSG00000185201	0,864	1,627786756	0,728	0,76	0,04
Endothelial cells	SPARCL1	ENSG00000152583	0,927	1,763342735	0,854	0,97	0,25
Endothelial cells	ITGA6	ENSG00000091409	0,89	1,983521311	0,78	0,82	0,11
Endothelial cells	GALNT18	ENSG00000110328	0,945	2,376696322	0,89	0,94	0,23
Endothelial cells	RNF144B	ENSG00000137393	0,872	1,960926789	0,744	0,79	0,08
Endothelial cells	PAPSS2	ENSG00000198682	0,859	1,743648911	0,718	0,74	0,03
Endothelial cells	ID1	ENSG00000125968	0,859	1,963298288	0,718	0,74	0,03
Endothelial cells	IL4R	ENSG00000077238	0,864	1,676472827	0,728	0,77	0,07
Endothelial cells	EMP2	ENSG00000213853	0,863	1,585936347	0,726	0,76	0,06
Endothelial cells	NXN	ENSG00000167693	0,896	1,851863793	0,792	0,84	0,15
Endothelial cells	SLC9A3R2	ENSG00000065054	0,861	1,694958562	0,722	0,75	0,05
Endothelial cells	LEF1	ENSG00000138795	0,861	1,792360534	0,722	0,76	0,07
Endothelial cells	VIM	ENSG00000026025	0,857	1,555039173	0,714	0,77	0,08
Endothelial cells	IRAK3	ENSG00000090376	0,852	1,44090473	0,704	0,79	0,1
Endothelial cells	NOSTRIN	ENSG00000163072	0,847	1,624011616	0,694	0,71	0,02
Endothelial cells	NEDD9	ENSG00000111859	0,864	1,685217132	0,728	0,77	0,09
Endothelial cells	SRGN	ENSG00000122862	0,845	1,403444168	0,69	0,76	0,08
Endothelial cells	CRIM1	ENSG00000150938	0,928	2,381640445	0,856	0,91	0,23

Endothelial cells	ICAM2	ENSG00000108622	0,841	1,528116736	0,682	0,69	0,02
Endothelial cells	LIMS2	ENSG00000072163	0,844	1,480284712	0,688	0,7	0,03
Endothelial cells	SORBS2	ENSG00000154556	0,904	1,98549362	0,808	0,89	0,22
Endothelial cells	CDYL2	ENSG00000166446	0,871	1,755625482	0,742	0,78	0,11
Endothelial cells	TM4SF1	ENSG00000169908	0,838	1,582744048	0,676	0,69	0,02
Endothelial cells	ID3	ENSG00000117318	0,841	1,65584821	0,682	0,72	0,05
Endothelial cells	SLCO2B1	ENSG00000137491	0,827	1,147634641	0,654	0,77	0,11
Endothelial cells	TIE1	ENSG00000066056	0,833	1,454577999	0,666	0,68	0,02
Endothelial cells	PTPRG	ENSG00000144724	0,948	2,257232525	0,896	0,97	0,31
Endothelial cells	STOM	ENSG00000148175	0,875	1,520990488	0,75	0,83	0,17
Endothelial cells	PALMD	ENSG00000099260	0,836	1,679680134	0,672	0,69	0,03
Endothelial cells	CPNE8	ENSG00000139117	0,865	1,656543818	0,73	0,79	0,14
Endothelial cells	FAM107A	ENSG00000168309	0,857	1,560756937	0,714	0,79	0,13
Endothelial cells	TGFBR2	ENSG00000163513	0,836	1,37025601	0,672	0,77	0,11
Endothelial cells	ETS2	ENSG00000157557	0,849	1,629742196	0,698	0,76	0,11
Endothelial cells	CADPS2	ENSG00000081803	0,872	1,643784171	0,744	0,84	0,19
Endothelial cells	OSMR	ENSG00000145623	0,839	1,504222109	0,678	0,72	0,08
Endothelial cells	RGS5	ENSG00000143248	0,84	1,656157275	0,68	0,74	0,1
Endothelial cells	HLA-C	ENSG00000204525	0,862	1,627774362	0,724	0,79	0,15
Endothelial cells	ELOVL7	ENSG00000164181	0,971	2,49835492	0,942	0,97	0,33
Endothelial cells	SLC1A1	ENSG00000106688	0,858	1,728428935	0,716	0,77	0,13
Endothelial cells	JCAD	ENSG00000165757	0,826	1,40853077	0,652	0,67	0,03
Endothelial cells	WWTR1	ENSG00000018408	0,883	1,723457092	0,766	0,85	0,21
Endothelial cells	XAF1	ENSG00000132530	0,829	1,398008273	0,658	0,71	0,07
Endothelial cells	SLC7A1	ENSG00000139514	0,877	2,06497787	0,754	0,8	0,17
Endothelial cells	S100A10	ENSG00000197747	0,826	1,577376079	0,652	0,68	0,04
Endothelial cells	UTRN	ENSG00000152818	0,883	1,40802982	0,766	0,93	0,3
Endothelial cells	CRIP2	ENSG00000182809	0,843	1,444447494	0,686	0,74	0,11
Endothelial cells	CYYR1	ENSG00000166265	0,82	1,621069684	0,64	0,65	0,02
Endothelial cells	RBMS2	ENSG00000076067	0,848	1,389983319	0,696	0,76	0,13
Endothelial cells	ANXA3	ENSG00000138772	0,818	1,550601685	0,636	0,65	0,02
Endothelial cells	PLXNA2	ENSG00000076356	0,862	1,680392639	0,724	0,8	0,18
Endothelial cells	SGPP2	ENSG00000163082	0,839	1,665615316	0,678	0,72	0,09
Endothelial cells	EMCN	ENSG00000164035	0,815	1,65702389	0,63	0,64	0,02
Endothelial cells	SRARP	ENSG00000183888	0,81	1,375284899	0,62	0,63	0,01
Endothelial cells	CEMIP2	ENSG00000135048	0,841	1,616012461	0,682	0,75	0,13
Endothelial cells	MRTFB	ENSG00000186260	0,931	2,084390837	0,862	0,92	0,31
Endothelial cells	LRRC32	ENSG00000137507	0,806	1,44808985	0,612	0,63	0,02
Endothelial cells	ADAMTS9	ENSG00000163638	0,814	2,145084682	0,628	0,68	0,08
Endothelial cells	ESAM	ENSG00000149564	0,805	1,29613925	0,61	0,63	0,02
Endothelial cells	NAMPT	ENSG00000105835	0,849	1,675427787	0,698	0,77	0,17
Endothelial cells	BSG	ENSG00000172270	0,948	2,497179755	0,896	0,96	0,36
Endothelial cells	ITIH5	ENSG00000123243	0,799	1,430999738	0,598	0,65	0,06
Endothelial cells	PARP14	ENSG00000173193	0,81	1,301828504	0,62	0,7	0,11
Endothelial cells	APOLD1	ENSG00000178878	0,846	1,369004914	0,692	0,82	0,23
Endothelial cells	CAVIN2	ENSG00000168497	0,794	1,443722658	0,588	0,6	0,02
Endothelial cells	EBF1	ENSG00000164330	0,798	1,187433123	0,596	0,71	0,13
Endothelial cells	RPGR	ENSG00000156313	0,843	1,522940217	0,686	0,76	0,18
Endothelial cells	MYRIP	ENSG00000170011	0,881	2,015033567	0,762	0,85	0,27
Endothelial cells	AHNAK	ENSG00000124942	0,801	1,115003822	0,602	0,69	0,11
Endothelial cells	ZNF366	ENSG00000178175	0,791	1,613239201	0,582	0,59	0,02
Endothelial cells	BST2	ENSG00000130303	0,792	1,29691963	0,584	0,62	0,04
Endothelial cells	GIMAP7	ENSG00000179144	0,789	1,300356638	0,578	0,59	0,02
Endothelial cells	ADGRL2	ENSG00000117114	0,801	1,32318412	0,602	0,71	0,13
Endothelial cells	PTPN14	ENSG00000152104	0,802	1,249873302	0,604	0,68	0,11

Endothelial cells	ARHGAP31	ENSG00000031081	0,814	1,198763534	0,628	0,78	0,21
Endothelial cells	ACSL5	ENSG00000197142	0,787	1,230690803	0,574	0,59	0,02
Endothelial cells	GNG11	ENSG00000127920	0,784	1,254502536	0,568	0,59	0,03
Endothelial cells	HLA-A	ENSG00000206503	0,843	1,513684904	0,686	0,78	0,22
Endothelial cells	MT2A	ENSG00000125148	0,899	2,171978902	0,798	0,91	0,35
Endothelial cells	GGT5	ENSG00000099998	0,78	1,083395128	0,56	0,61	0,05
Endothelial cells	FN1	ENSG00000115414	0,798	1,409446836	0,596	0,66	0,1
Endothelial cells	ACER2	ENSG00000177076	0,797	1,551197214	0,594	0,63	0,07
Endothelial cells	YBX3	ENSG00000060138	0,792	1,302118874	0,584	0,65	0,09
Endothelial cells	RUNDC3B	ENSG00000105784	0,807	1,411601913	0,614	0,7	0,14
Endothelial cells	CFH	ENSG00000000971	0,781	1,734162634	0,562	0,58	0,03
Endothelial cells	ANGPT2	ENSG00000091879	0,798	2,684612869	0,596	0,63	0,08
Endothelial cells	ST6GAL1	ENSG00000073849	0,789	0,933453895	0,578	0,78	0,22
Endothelial cells	EPB41L4A	ENSG00000129595	0,834	1,46175371	0,668	0,77	0,21
Endothelial cells	SP100	ENSG00000067066	0,782	1,093029658	0,564	0,68	0,13
Endothelial cells	TIMP3	ENSG00000100234	0,804	1,301225372	0,608	0,72	0,17
Endothelial cells	LY6E	ENSG00000160932	0,789	1,310077682	0,578	0,61	0,06
Endothelial cells	PAM	ENSG00000145730	0,825	1,271382607	0,65	0,82	0,26
Endothelial cells	ACVRL1	ENSG00000139567	0,777	1,130664335	0,554	0,56	0,01
Endothelial cells	SMAD6	ENSG00000137834	0,781	1,623825405	0,562	0,59	0,04
Endothelial cells	NOTCH4	ENSG00000204301	0,775	1,142628776	0,55	0,56	0,01
Endothelial cells	ETS1	ENSG00000134954	0,784	1,209650115	0,568	0,63	0,08
Endothelial cells	PARVB	ENSG00000188677	0,816	1,357638402	0,632	0,71	0,16
Endothelial cells	GALNT15	ENSG00000131386	0,855	1,438197361	0,71	0,84	0,3
Endothelial cells	CDH5	ENSG00000179776	0,773	1,114642302	0,546	0,55	0,01
Endothelial cells	B2M	ENSG00000166710	0,922	1,896244497	0,844	0,94	0,39
Endothelial cells	DIPK2B	ENSG00000147113	0,805	1,321213124	0,61	0,7	0,16
Endothelial cells	LGALS3	ENSG00000131981	0,776	1,215933392	0,552	0,58	0,03
Endothelial cells	MYOF	ENSG00000138119	0,781	1,256870279	0,562	0,64	0,1
Endothelial cells	ROBO4	ENSG00000154133	0,772	1,14052962	0,544	0,55	0,01
Endothelial cells	NFKBIA	ENSG00000100906	0,818	1,442210431	0,636	0,73	0,19
Endothelial cells	MFSD2A	ENSG00000168389	0,776	1,375516769	0,552	0,57	0,03
Endothelial cells	C22orf34	ENSG00000188511	0,776	1,304894161	0,552	0,6	0,06
Endothelial cells	HIF1A-AS3	ENSG00000258667	0,786	1,88547011	0,572	0,63	0,1
Endothelial cells	SLC16A1	ENSG00000155380	0,797	1,782931914	0,594	0,65	0,12
Endothelial cells	DOCK6	ENSG00000130158	0,776	1,147627709	0,552	0,6	0,07
Endothelial cells	PDE10A	ENSG00000112541	0,824	1,430451043	0,648	0,78	0,25
Endothelial cells	INPP5D	ENSG00000168918	0,755	0,744362704	0,51	0,65	0,12
Endothelial cells	SLC2A3	ENSG00000059804	0,79	1,574128932	0,58	0,66	0,13
Endothelial cells	TEK	ENSG00000120156	0,776	1,269242851	0,552	0,59	0,06
Endothelial cells	PPFIBP1	ENSG00000110841	0,92	1,786420814	0,84	0,93	0,4
Endothelial cells	SPOCK2	ENSG00000107742	0,812	1,314115549	0,624	0,73	0,2
Endothelial cells	TFRC	ENSG00000072274	0,795	1,441857488	0,59	0,66	0,13
Endothelial cells	FGD5	ENSG00000154783	0,766	1,094428866	0,532	0,55	0,02
Endothelial cells	DEPP1	ENSG00000165507	0,769	1,617427409	0,538	0,56	0,04
Endothelial cells	SLC39A10	ENSG00000196950	0,86	1,815087034	0,72	0,82	0,3
Endothelial cells	PLSCR1	ENSG00000188313	0,783	1,173834047	0,566	0,65	0,13
Endothelial cells	SEC14L1	ENSG00000129657	0,916	1,74706784	0,832	0,91	0,39
Endothelial cells	CHSY1	ENSG00000131873	0,834	1,465939962	0,668	0,79	0,27
Endothelial cells	HERPUD1	ENSG00000051108	0,819	1,390055661	0,638	0,74	0,22
Endothelial cells	CCDC85A	ENSG00000055813	0,785	1,490280014	0,57	0,66	0,14
Endothelial cells	PLSCR4	ENSG00000114698	0,778	1,207749016	0,556	0,65	0,13
Endothelial cells	EPHA4	ENSG00000116106	0,778	1,314423309	0,556	0,62	0,1
Endothelial cells	HYAL2	ENSG00000068001	0,765	1,188813578	0,53	0,55	0,03
Endothelial cells	CP	ENSG00000047457	0,775	1,95218664	0,55	0,6	0,08

Endothelial cells	MEF2C	ENSG00000081189	0,768	0,734464007	0,536	0,75	0,23
Endothelial cells	KANK3	ENSG00000186994	0,762	1,029622962	0,524	0,55	0,04
Endothelial cells	ESYT2	ENSG00000117868	0,923	1,806800913	0,846	0,93	0,41
Endothelial cells	ZEB1	ENSG00000148516	0,8	0,993972298	0,6	0,85	0,33
Endothelial cells	TPM1	ENSG00000140416	0,807	1,187144168	0,614	0,73	0,22
Endothelial cells	DUSP1	ENSG00000120129	0,772	1,255170713	0,544	0,62	0,12
Endothelial cells	TMEM123	ENSG00000152558	0,828	1,330151745	0,656	0,78	0,28
Endothelial cells	SAT1	ENSG00000130066	0,794	1,403456665	0,588	0,73	0,22
Endothelial cells	GRASP	ENSG00000161835	0,755	1,104817955	0,51	0,52	0,02
Endothelial cells	LMO2	ENSG00000135363	0,76	1,060242851	0,52	0,57	0,06
Endothelial cells	RHOC	ENSG00000155366	0,76	1,065944787	0,52	0,57	0,06
Endothelial cells	LHFPL6	ENSG00000183722	0,832	1,352608752	0,664	0,87	0,37
Endothelial cells	SVIL	ENSG00000197321	0,78	1,326495258	0,56	0,63	0,13
Endothelial cells	TPST2	ENSG00000128294	0,766	1,185154447	0,532	0,58	0,07
Endothelial cells	FOSL2	ENSG00000075426	0,757	1,147011312	0,514	0,55	0,04
Endothelial cells	RBMS3	ENSG00000144642	0,778	1,222939575	0,556	0,67	0,17
Endothelial cells	RELL1	ENSG00000181826	0,792	1,254123642	0,584	0,68	0,18
Endothelial cells	ABLIM1	ENSG00000099204	0,79	1,036041003	0,58	0,79	0,29
Endothelial cells	MYH9	ENSG00000100345	0,82	1,192065106	0,64	0,78	0,27
Endothelial cells	RAMP2	ENSG00000131477	0,753	1,16912527	0,506	0,52	0,02
Endothelial cells	SCARB1	ENSG00000073060	0,819	1,47602711	0,638	0,75	0,26
Endothelial cells	MCC	ENSG00000171444	0,789	1,462361878	0,578	0,71	0,21
Endothelial cells	MYO10	ENSG00000145555	0,795	1,2858151	0,59	0,71	0,21
Endothelial cells	PLEKHG1	ENSG00000120278	0,866	1,500788953	0,732	0,88	0,39
Endothelial cells	ANXA2	ENSG00000182718	0,752	1,073928002	0,504	0,55	0,05
Endothelial cells	HEG1	ENSG00000173706	0,814	1,321423719	0,628	0,76	0,27
Endothelial cells	EDN1	ENSG00000078401	0,749	1,863044192	0,498	0,51	0,02
Endothelial cells	SPARC	ENSG00000113140	0,82	1,100299861	0,64	0,83	0,34
Endothelial cells	NEBL	ENSG00000078114	0,751	0,718886283	0,502	0,84	0,35
Endothelial cells	DGKH	ENSG00000102780	0,782	1,186743198	0,564	0,67	0,18
Endothelial cells	SULF2	ENSG00000196562	0,758	1,036946762	0,516	0,61	0,12
Endothelial cells	SLC19A3	ENSG00000135917	0,751	1,101623798	0,502	0,52	0,04
Endothelial cells	NPAS2	ENSG00000170485	0,776	1,215182186	0,552	0,71	0,22
Endothelial cells	RBPMS	ENSG00000157110	0,747	1,005722863	0,494	0,6	0,11
Endothelial cells	PCAT19	ENSG00000267107	0,745	1,109286918	0,49	0,5	0,01
Endothelial cells	SLC39A8	ENSG00000138821	0,752	1,113142012	0,504	0,54	0,05
Endothelial cells	UACA	ENSG00000137831	0,809	1,172979811	0,618	0,77	0,29
Endothelial cells	SLC38A5	ENSG00000017483	0,742	1,18392068	0,484	0,49	0,01
Endothelial cells	LRCH1	ENSG00000136141	0,798	1,143699279	0,596	0,76	0,29
Endothelial cells	KCTD12	ENSG00000178695	0,747	1,03993489	0,494	0,58	0,1
Endothelial cells	SDCBP	ENSG00000137575	0,778	1,089972957	0,556	0,67	0,19
Endothelial cells	CAVIN1	ENSG00000177469	0,758	1,043061864	0,516	0,59	0,12
Endothelial cells	KLF2	ENSG00000127528	0,739	1,190432358	0,478	0,5	0,02
Endothelial cells	SLC30A1	ENSG00000170385	0,745	1,018520658	0,49	0,53	0,06
Endothelial cells	ACVR1	ENSG00000115170	0,788	1,334634449	0,576	0,68	0,21
Endothelial cells	TSPAN14	ENSG00000108219	0,772	0,997117525	0,544	0,68	0,21
Endothelial cells	CEP112	ENSG00000154240	0,773	1,035524235	0,546	0,73	0,26
Endothelial cells	RASIP1	ENSG00000105538	0,739	0,988883238	0,478	0,49	0,02
Endothelial cells	SORBS1	ENSG00000095637	0,795	0,793634869	0,59	0,87	0,4
Endothelial cells	PON2	ENSG00000105854	0,858	1,416025579	0,716	0,86	0,39
Endothelial cells	FOXC1	ENSG00000054598	0,736	0,979725068	0,472	0,49	0,02
Endothelial cells	TM4SF18	ENSG00000163762	0,736	1,091154179	0,472	0,48	0,01
Endothelial cells	MAP3K20	ENSG00000091436	0,753	1,032501066	0,506	0,61	0,15
Endothelial cells	SNED1	ENSG00000162804	0,753	1,122366342	0,506	0,62	0,15
Endothelial cells	S1PR1	ENSG00000170989	0,732	0,980010552	0,464	0,49	0,03

Endothelial cells	CEBPD	ENSG00000221869	0,741	1,053979699	0,482	0,6	0,14
Endothelial cells	PTPRM	ENSG00000173482	0,914	1,88520418	0,828	0,94	0,48
Endothelial cells	MT1E	ENSG00000169715	0,743	1,244729372	0,486	0,6	0,14
Endothelial cells	ARL15	ENSG00000185305	0,881	1,887434772	0,762	0,9	0,45
Endothelial cells	GPCPD1	ENSG00000125772	0,76	1,222133542	0,52	0,64	0,19
Endothelial cells	S100A11	ENSG00000163191	0,729	0,962078544	0,458	0,51	0,06
Endothelial cells	CFLAR	ENSG00000003402	0,853	1,314734464	0,706	0,85	0,39
Endothelial cells	VAMP5	ENSG00000168899	0,73	1,046431419	0,46	0,49	0,05
Endothelial cells	EPSTI1	ENSG00000133106	0,728	1,11718168	0,456	0,51	0,06
Endothelial cells	PDXK	ENSG00000160209	0,795	1,233750605	0,59	0,73	0,28
Endothelial cells	PTTG1IP	ENSG00000183255	0,794	1,065214304	0,588	0,74	0,29
Endothelial cells	RBMS1	ENSG00000153250	0,773	1,026878708	0,546	0,73	0,28
Endothelial cells	MYL12B	ENSG00000118680	0,758	1,111476083	0,516	0,61	0,16
Endothelial cells	EGFR	ENSG00000146648	0,734	0,970563932	0,468	0,63	0,18
Endothelial cells	NET1	ENSG00000173848	0,733	1,207283768	0,466	0,5	0,06
Endothelial cells	USP6NL	ENSG00000148429	0,777	1,040931516	0,554	0,72	0,27
Endothelial cells	TMEM132C	ENSG00000181234	0,754	1,176706078	0,508	0,69	0,24
Endothelial cells	UNC13B	ENSG00000198722	0,79	1,264605051	0,58	0,71	0,26
Endothelial cells	TMEM204	ENSG00000131634	0,723	0,938082652	0,446	0,46	0,02
Endothelial cells	MGLL	ENSG00000074416	0,748	0,952755492	0,496	0,65	0,2
Endothelial cells	PLAT	ENSG00000104368	0,727	1,197332667	0,454	0,48	0,04
Endothelial cells	TSPAN9	ENSG00000011105	0,735	0,979724814	0,47	0,53	0,09
Endothelial cells	THSD7A	ENSG00000005108	0,772	1,401447077	0,544	0,71	0,27
Endothelial cells	GBP4	ENSG00000162654	0,719	1,406470974	0,438	0,45	0,02
Endothelial cells	DAB2IP	ENSG00000136848	0,744	1,038861882	0,488	0,59	0,15
Endothelial cells	MSRB3	ENSG00000174099	0,739	1,060483706	0,478	0,57	0,14
Endothelial cells	LSR	ENSG00000105699	0,719	0,906778035	0,438	0,45	0,01
Endothelial cells	RAPGEF4	ENSG00000091428	0,773	1,04745858	0,546	0,76	0,33
Endothelial cells	GPR85	ENSG00000164604	0,725	1,242988213	0,45	0,48	0,05
Endothelial cells	BMPR2	ENSG00000204217	0,893	1,468382511	0,786	0,93	0,5
Endothelial cells	SLC52A3	ENSG00000101276	0,714	0,937419278	0,428	0,43	0,01
Endothelial cells	TMOD3	ENSG00000138594	0,76	0,992262291	0,52	0,66	0,23
Endothelial cells	CLIC1	ENSG00000213719	0,715	0,861014506	0,43	0,46	0,04
Endothelial cells	YES1	ENSG00000176105	0,733	0,988364306	0,466	0,55	0,13
Endothelial cells	BMP6	ENSG00000153162	0,717	1,712003479	0,434	0,45	0,03
Endothelial cells	CST3	ENSG00000101439	0,739	0,733381076	0,478	0,76	0,34
Endothelial cells	LEPR	ENSG00000116678	0,744	1,036343476	0,488	0,61	0,19
Endothelial cells	ADIRF	ENSG00000148671	0,713	1,040423974	0,426	0,47	0,05
Endothelial cells	ITPR3	ENSG00000096433	0,712	0,899599528	0,424	0,43	0,01
Endothelial cells	WWC2	ENSG00000151718	0,808	1,268318988	0,616	0,78	0,36
Endothelial cells	RIMKLB	ENSG00000166532	0,755	1,378807418	0,51	0,63	0,22
Endothelial cells	JAM2	ENSG00000154721	0,723	1,03242282	0,446	0,53	0,11
Endothelial cells	TINAGL1	ENSG00000142910	0,71	0,832104169	0,42	0,43	0,02
Endothelial cells	AKAP12	ENSG00000131016	0,737	1,245145476	0,474	0,59	0,17
Endothelial cells	TCN2	ENSG00000185339	0,715	0,893561419	0,43	0,46	0,04
Endothelial cells	CNOT8	ENSG00000155508	0,736	0,94623393	0,472	0,56	0,14
Endothelial cells	CNTNAP3B	ENSG00000154529	0,735	1,121518738	0,47	0,6	0,18
Endothelial cells	TUBB6	ENSG00000176014	0,711	0,874988158	0,422	0,45	0,03
Endothelial cells	MT1M	ENSG00000205364	0,716	1,114499293	0,432	0,5	0,09
Endothelial cells	SLC3A2	ENSG00000168003	0,744	1,073482563	0,488	0,61	0,19
Endothelial cells	RHOJ	ENSG00000126785	0,71	0,947956171	0,42	0,48	0,06
Endothelial cells	NES	ENSG00000132688	0,713	0,896465609	0,426	0,47	0,05
Endothelial cells	MYL12A	ENSG00000101608	0,716	0,927899779	0,432	0,48	0,07
Endothelial cells	SHE	ENSG00000169291	0,708	0,869740079	0,416	0,42	0,01
Endothelial cells	CCDC85B	ENSG00000175602	0,735	0,952874695	0,47	0,57	0,16

Endothelial cells	LAMA5	ENSG00000130702	0,715	0,888763895	0,43	0,47	0,06
Endothelial cells	IFI16	ENSG00000163565	0,722	0,863341009	0,444	0,61	0,2
Endothelial cells	RAPGEF1	ENSG00000107263	0,795	1,07248656	0,59	0,8	0,39
Endothelial cells	LMBR1	ENSG00000105983	0,823	1,172838978	0,646	0,81	0,4
Endothelial cells	VSIR	ENSG00000107738	0,723	0,912399711	0,446	0,58	0,17
Endothelial cells	PXN	ENSG00000089159	0,728	0,948851235	0,456	0,56	0,15
Endothelial cells	LAMA3	ENSG00000053747	0,712	1,149026295	0,424	0,46	0,05
Endothelial cells	AP3S1	ENSG00000177879	0,777	1,154367591	0,554	0,7	0,29
Endothelial cells	HMCN1	ENSG00000143341	0,707	1,208872993	0,414	0,44	0,03
Endothelial cells	KLF6	ENSG00000067082	0,729	0,93016606	0,458	0,58	0,18
Endothelial cells	PRSS23	ENSG00000150687	0,714	1,150193819	0,428	0,47	0,07
Endothelial cells	TSC22D1	ENSG00000102804	0,889	1,604183134	0,778	0,92	0,52
Endothelial cells	TPT1	ENSG00000133112	0,802	1,155021151	0,604	0,8	0,4
Endothelial cells	ARHGAP26	ENSG00000145819	0,772	0,808599201	0,544	0,87	0,47
Endothelial cells	WARS	ENSG00000140105	0,731	1,324949149	0,462	0,55	0,15
Endothelial cells	PTMS	ENSG00000159335	0,714	0,876511071	0,428	0,51	0,11
Endothelial cells	MSN	ENSG00000147065	0,724	0,860749317	0,448	0,6	0,2
Endothelial cells	CTNNB1	ENSG00000168036	0,812	1,143357328	0,624	0,81	0,41
Endothelial cells	CLEC14A	ENSG00000176435	0,701	0,863731333	0,402	0,42	0,03
Endothelial cells	PERP	ENSG00000112378	0,701	0,871121511	0,402	0,42	0,03
Endothelial cells	TMEM87B	ENSG00000153214	0,73	0,978430179	0,46	0,58	0,18
Endothelial cells	NEDD4	ENSG00000069869	0,727	0,980577398	0,454	0,58	0,2
Endothelial cells	PRICKLE2	ENSG00000163637	0,752	1,231382622	0,504	0,69	0,3
Endothelial cells	MCTP1	ENSG00000175471	0,719	1,396973486	0,438	0,55	0,16
Endothelial cells	CNOT6L	ENSG00000138767	0,792	1,152849901	0,584	0,76	0,37
Endothelial cells	PDZRN3	ENSG00000121440	0,716	1,31270756	0,432	0,56	0,18
Endothelial cells	GNAI2	ENSG00000114353	0,798	1,055105003	0,596	0,79	0,41
Endothelial cells	OCLN	ENSG00000197822	0,712	0,903917689	0,424	0,5	0,12
Endothelial cells	NFIB	ENSG00000147862	0,803	1,120520597	0,606	0,89	0,5
Endothelial cells	SYNE1	ENSG00000131018	0,88	1,546591985	0,76	0,94	0,55
Endothelial cells	SHANK3	ENSG00000251322	0,741	0,932569103	0,482	0,63	0,25
Endothelial cells	TDRP	ENSG00000180190	0,713	0,957461617	0,426	0,51	0,13
Endothelial cells	EEF1A1	ENSG00000156508	0,82	1,195585662	0,64	0,84	0,47
Endothelial cells	KLHL5	ENSG00000109790	0,754	0,972863887	0,508	0,71	0,34
Endothelial cells	CCDC50	ENSG00000152492	0,747	0,887058268	0,494	0,69	0,32
Endothelial cells	SWAP70	ENSG00000133789	0,715	0,829114397	0,43	0,58	0,21
Endothelial cells	TMSB4X	ENSG00000205542	0,822	1,137556506	0,644	0,87	0,5
Endothelial cells	ITGB1	ENSG00000150093	0,777	0,969729144	0,554	0,75	0,38
Endothelial cells	CD2AP	ENSG00000198087	0,731	0,926590839	0,462	0,61	0,24
Endothelial cells	ACTN1	ENSG00000072110	0,707	0,936093007	0,414	0,51	0,14
Endothelial cells	PRKD3	ENSG00000115825	0,701	0,804435549	0,402	0,54	0,17
Endothelial cells	GFAP	ENSG00000131095	0,703	0,636374964	0,406	0,69	0,32
Endothelial cells	KIAA1549	ENSG00000122778	0,706	0,884697985	0,412	0,51	0,14
Endothelial cells	FAT4	ENSG00000196159	0,704	0,990298282	0,408	0,53	0,16
Endothelial cells	ETV6	ENSG00000139083	0,717	0,68085918	0,434	0,71	0,35
Endothelial cells	BACE2	ENSG00000182240	0,711	1,130396952	0,422	0,59	0,23
Endothelial cells	TPM3	ENSG00000143549	0,722	0,874594696	0,444	0,59	0,23
Endothelial cells	CTTNBP2NL	ENSG00000143079	0,721	0,88999629	0,442	0,61	0,25
Endothelial cells	FNDC3B	ENSG00000075420	0,729	0,799952281	0,458	0,72	0,36
Endothelial cells	RAC1	ENSG00000136238	0,795	1,010758264	0,59	0,81	0,46
Endothelial cells	SPTAN1	ENSG00000197694	0,714	0,747185309	0,428	0,67	0,32
Endothelial cells	SERPINB6	ENSG00000124570	0,775	0,980103386	0,55	0,76	0,41
Endothelial cells	GMDS	ENSG00000112699	0,741	0,981775819	0,482	0,7	0,36
Endothelial cells	SUSD6	ENSG00000100647	0,709	0,786281943	0,418	0,62	0,28
Endothelial cells	ARHGEF12	ENSG00000196914	0,754	0,819125955	0,508	0,79	0,45

Endothelial cells	HIVEP1	ENSG00000095951	0,706	1,061386869	0,412	0,57	0,23
Endothelial cells	NPIPB5	ENSG00000243716	0,741	1,263472767	0,482	0,65	0,32
Endothelial cells	GFOD1	ENSG00000145990	0,723	1,048114151	0,446	0,61	0,29
Endothelial cells	STAT3	ENSG00000168610	0,76	0,854926595	0,52	0,78	0,46
Endothelial cells	SERF2	ENSG00000140264	0,758	0,916454921	0,516	0,76	0,44
Endothelial cells	TACC1	ENSG00000147526	0,932	1,584014363	0,864	0,97	0,66
Endothelial cells	ANKS1A	ENSG00000064999	0,796	1,186122411	0,592	0,82	0,5
Endothelial cells	ARFGEF2	ENSG00000124198	0,749	0,936985783	0,498	0,72	0,4
Endothelial cells	HIPK3	ENSG00000110422	0,759	1,101773836	0,518	0,74	0,42
Endothelial cells	PDLIM5	ENSG00000163110	0,702	0,839056854	0,404	0,63	0,32
Endothelial cells	SH3BP4	ENSG00000130147	0,702	0,797347234	0,404	0,6	0,29
Endothelial cells	EXOC6	ENSG00000138190	0,738	0,96637052	0,476	0,7	0,39
Endothelial cells	PPP3CC	ENSG00000120910	0,743	0,936165683	0,486	0,7	0,39
Endothelial cells	RBM17	ENSG00000134453	0,708	0,857562506	0,416	0,59	0,28
Endothelial cells	MCF2L	ENSG00000126217	0,749	0,879107175	0,498	0,74	0,43
Endothelial cells	TPM4	ENSG00000167460	0,703	0,771922811	0,406	0,6	0,29
Endothelial cells	DSTN	ENSG00000125868	0,712	0,834503872	0,424	0,61	0,31
Endothelial cells	UBC	ENSG00000150991	0,788	1,035214075	0,576	0,85	0,55
Endothelial cells	TNS1	ENSG00000079308	0,715	0,816802144	0,43	0,71	0,41
Endothelial cells	RASAL2	ENSG00000075391	0,763	0,96802948	0,526	0,81	0,51
Endothelial cells	GRB10	ENSG00000106070	0,766	1,085536084	0,532	0,78	0,48
Endothelial cells	CD46	ENSG00000117335	0,734	0,905249818	0,468	0,68	0,38
Endothelial cells	ST6GALNAC3	ENSG00000184005	0,94	1,696170693	0,88	0,98	0,69
Endothelial cells	AGFG1	ENSG00000173744	0,8	1,024297362	0,6	0,82	0,52
Endothelial cells	FLNB	ENSG00000136068	0,728	0,901920933	0,456	0,67	0,37
Endothelial cells	H3F3B	ENSG00000132475	0,74	0,773010209	0,48	0,77	0,47
Endothelial cells	RBFOX2	ENSG00000100320	0,779	0,891825917	0,558	0,84	0,55
Endothelial cells	TUBA1B	ENSG00000123416	0,711	0,756843747	0,422	0,68	0,39
Endothelial cells	NR3C2	ENSG00000151623	0,756	1,08553587	0,512	0,78	0,49
Endothelial cells	FCHO2	ENSG00000157107	0,714	0,865106496	0,428	0,65	0,36
Endothelial cells	ACTG1	ENSG00000184009	0,76	0,93149781	0,52	0,8	0,51
Endothelial cells	AFF1	ENSG00000172493	0,74	0,843361834	0,48	0,78	0,5
Endothelial cells	PIK3C2A	ENSG00000011405	0,744	0,856264985	0,488	0,74	0,46
Endothelial cells	PTPN12	ENSG00000127947	0,731	0,806343441	0,462	0,72	0,43
Endothelial cells	KIAA0355	ENSG00000166398	0,703	0,811330393	0,406	0,62	0,34
Endothelial cells	PPP3CA	ENSG00000138814	0,716	0,727966848	0,432	0,81	0,54
Endothelial cells	RAP1B	ENSG00000127314	0,712	0,762575833	0,424	0,68	0,41
Endothelial cells	HERC2	ENSG00000128731	0,922	1,705746009	0,844	0,95	0,68
Endothelial cells	ADIPOR2	ENSG00000006831	0,79	0,925645015	0,58	0,93	0,66
Endothelial cells	FEZ2	ENSG00000171055	0,706	0,763026251	0,412	0,66	0,39
Endothelial cells	DOCK9	ENSG00000088387	0,892	1,305233956	0,784	0,97	0,71
Endothelial cells	RAPGEF2	ENSG00000109756	0,821	1,103897525	0,642	0,88	0,63
Endothelial cells	TRIO	ENSG00000038382	0,77	0,829651418	0,54	0,89	0,63
Endothelial cells	TANC1	ENSG00000115183	0,752	0,814627813	0,504	0,84	0,59
Endothelial cells	RIN2	ENSG00000132669	0,711	0,780892578	0,422	0,75	0,5
Endothelial cells	LDLRAD3	ENSG00000179241	0,735	0,900122704	0,47	0,79	0,54
Endothelial cells	EIF1	ENSG00000173812	0,725	0,766214438	0,45	0,77	0,53
Endothelial cells	IGF1R	ENSG00000140443	0,856	1,27269968	0,712	0,93	0,69
Endothelial cells	ACTN4	ENSG00000130402	0,736	0,733864953	0,472	0,81	0,58
Endothelial cells	ASAP1	ENSG00000153317	0,801	0,925225655	0,602	0,91	0,69
Endothelial cells	TTC28	ENSG00000100154	0,729	0,802530557	0,458	0,82	0,6
Endothelial cells	ZBTB38	ENSG00000177311	0,708	0,712043331	0,416	0,73	0,52
Endothelial cells	MEF2A	ENSG00000068305	0,741	0,596746983	0,482	0,87	0,66
Endothelial cells	PTMA	ENSG00000187514	0,741	0,803397725	0,482	0,86	0,66
Endothelial cells	SPTBN1	ENSG00000115306	0,847	1,053559229	0,694	0,94	0,74

Endothelial cells	GAPDH	ENSG00000111640	0,735	0,694219085	0,47	0,87	0,7
Endothelial cells	SMCHD1	ENSG00000101596	0,708	0,783131586	0,416	0,74	0,57
Endothelial cells	ADD1	ENSG00000087274	0,762	0,815775663	0,524	0,85	0,68
Endothelial cells	AFDN	ENSG00000130396	0,718	0,722865105	0,436	0,82	0,65
Endothelial cells	CCNY	ENSG00000108100	0,828	1,124076879	0,656	0,93	0,76
Endothelial cells	KAT6A	ENSG00000083168	0,718	0,687498049	0,436	0,78	0,61
Endothelial cells	ACTB	ENSG00000075624	0,753	0,754994248	0,506	0,92	0,77
Endothelial cells	KTN1	ENSG00000126777	0,702	0,619467061	0,404	0,79	0,64
Endothelial cells	INSR	ENSG00000171105	0,717	0,970673537	0,434	0,8	0,66
Endothelial cells	TCF4	ENSG00000196628	0,806	0,895752074	0,612	0,96	0,84
Endothelial cells	CSNK1A1	ENSG00000113712	0,712	0,60404674	0,424	0,84	0,73
Endothelial cells	FOXP1	ENSG00000114861	0,725	0,636648476	0,45	0,91	0,81
Endothelial cells	RALGAPA1	ENSG00000174373	0,713	0,801664277	0,426	0,85	0,76
Pericytes	PDGFRB	ENSG00000113721	0,961	2,339882991	0,922	0,94	0,08
Pericytes	COBLL1	ENSG00000082438	0,941	1,99810348	0,882	0,93	0,07
Pericytes	PTH1R	ENSG00000160801	0,944	2,446474131	0,888	0,9	0,04
Pericytes	DCN	ENSG00000011465	0,933	2,249121022	0,866	0,87	0,02
Pericytes	NDUFA4L2	ENSG00000185633	0,931	2,436985004	0,862	0,87	0,03
Pericytes	NOTCH3	ENSG00000074181	0,921	2,129174017	0,842	0,85	0,01
Pericytes	EBF1	ENSG00000164330	0,961	2,44401493	0,922	0,96	0,13
Pericytes	MYO1B	ENSG00000128641	0,926	2,011191797	0,852	0,87	0,05
Pericytes	IFITM3	ENSG00000142089	0,93	2,090321944	0,86	0,91	0,09
Pericytes	GGT5	ENSG00000099998	0,914	1,836593641	0,828	0,86	0,05
Pericytes	SLC6A12	ENSG00000111181	0,91	2,304409469	0,82	0,83	0,02
Pericytes	RGS5	ENSG00000143248	0,938	2,463966182	0,876	0,9	0,1
Pericytes	ITIH5	ENSG00000123243	0,914	2,021194478	0,828	0,86	0,06
Pericytes	IGFBP7	ENSG00000163453	0,938	2,23398022	0,876	0,93	0,13
Pericytes	SLC38A11	ENSG00000169507	0,901	2,185125767	0,802	0,8	0,01
Pericytes	EPAS1	ENSG00000116016	0,902	1,757601673	0,804	0,87	0,08
Pericytes	ARHGAP29	ENSG00000137962	0,927	1,85985594	0,854	0,91	0,12
Pericytes	RBPMS	ENSG00000157110	0,91	1,863850017	0,82	0,88	0,11
Pericytes	NID1	ENSG00000116962	0,897	1,833652478	0,794	0,81	0,04
Pericytes	PRELP	ENSG00000188783	0,885	1,896307632	0,77	0,78	0,02
Pericytes	BGN	ENSG00000182492	0,886	1,902660268	0,772	0,79	0,03
Pericytes	GJC1	ENSG00000182963	0,88	1,745315843	0,76	0,77	0,01
Pericytes	HES4	ENSG00000188290	0,889	1,910801169	0,778	0,8	0,06
Pericytes	TIMP3	ENSG00000100234	0,92	2,050179382	0,84	0,9	0,17
Pericytes	ARHGAP10	ENSG00000071205	0,9	1,821159034	0,8	0,85	0,12
Pericytes	ARHGAP42	ENSG00000165895	0,902	1,817070182	0,804	0,87	0,14
Pericytes	MYL9	ENSG00000101335	0,868	1,740806178	0,736	0,75	0,01
Pericytes	SYNE2	ENSG00000054654	0,891	1,563994762	0,782	0,85	0,13
Pericytes	LAMA2	ENSG00000196569	0,934	2,330059004	0,868	0,95	0,23
Pericytes	NR2F2-AS1	ENSG00000247809	0,883	1,778252792	0,766	0,84	0,12
Pericytes	NR2F2	ENSG00000185551	0,88	1,601655402	0,76	0,79	0,07
Pericytes	SLC30A10	ENSG00000196660	0,882	1,794722117	0,764	0,79	0,08
Pericytes	CARMN	ENSG00000249669	0,857	1,759181624	0,714	0,72	0
Pericytes	ATP1A2	ENSG00000018625	0,901	2,095947337	0,802	0,91	0,2
Pericytes	TFPI	ENSG00000003436	0,856	1,652987272	0,712	0,72	0,01
Pericytes	FN1	ENSG00000115414	0,883	1,846996816	0,766	0,81	0,11
Pericytes	RBMS3	ENSG00000144642	0,898	1,69621362	0,796	0,87	0,17
Pericytes	LZTS1	ENSG00000061337	0,856	1,572996396	0,712	0,72	0,01
Pericytes	MIR4435-2HG	ENSG00000172965	0,885	2,165707558	0,77	0,81	0,11
Pericytes	PRKG1	ENSG00000185532	0,915	1,904190267	0,83	0,94	0,25
Pericytes	RHOJ	ENSG00000126785	0,854	1,677253254	0,708	0,75	0,06
Pericytes	SPARCL1	ENSG00000152583	0,925	1,939554268	0,85	0,95	0,26

Pericytes	RIPOR3	ENSG00000042062	0,849	1,644118815	0,698	0,71	0,03
Pericytes	COLEC12	ENSG00000158270	0,86	1,966114718	0,72	0,78	0,1
Pericytes	ITGA1	ENSG00000213949	0,848	1,455318357	0,696	0,75	0,07
Pericytes	PDE7B	ENSG00000171408	0,92	2,185156834	0,84	0,92	0,25
Pericytes	PLXDC1	ENSG00000161381	0,846	1,575173583	0,692	0,71	0,04
Pericytes	COL5A3	ENSG00000080573	0,839	1,382268353	0,678	0,76	0,09
Pericytes	HIGD1B	ENSG00000131097	0,835	1,572186887	0,67	0,68	0,02
Pericytes	CFH	ENSG00000000971	0,831	1,463712764	0,662	0,69	0,03
Pericytes	COL1A2	ENSG00000164692	0,831	1,430815902	0,662	0,66	0
Pericytes	COL4A2	ENSG00000134871	0,849	1,827888779	0,698	0,74	0,08
Pericytes	AC092957.1	ENSG00000243620	0,835	1,847297301	0,67	0,7	0,04
Pericytes	FRMD3	ENSG00000172159	0,886	1,981649999	0,772	0,83	0,17
Pericytes	CEBPD	ENSG00000221869	0,863	1,78864675	0,726	0,8	0,14
Pericytes	UTRN	ENSG00000152818	0,919	1,619731605	0,838	0,96	0,31
Pericytes	C11orf96	ENSG00000187479	0,838	2,010205619	0,676	0,7	0,05
Pericytes	SLC12A7	ENSG00000113504	0,832	1,488145995	0,664	0,69	0,04
Pericytes	KCNT2	ENSG00000162687	0,847	1,597972849	0,694	0,78	0,13
Pericytes	PTPRG	ENSG00000144724	0,903	1,565141098	0,806	0,96	0,32
Pericytes	ZIC1	ENSG00000152977	0,848	1,453562857	0,696	0,78	0,14
Pericytes	FLNA	ENSG00000196924	0,844	1,505333234	0,688	0,75	0,11
Pericytes	RNF152	ENSG00000176641	0,885	2,135966664	0,77	0,83	0,19
Pericytes	LAMC3	ENSG00000050555	0,82	1,446904582	0,64	0,65	0,02
Pericytes	VIM	ENSG00000026025	0,821	1,239307774	0,642	0,72	0,09
Pericytes	TRPC4	ENSG00000133107	0,833	1,763201987	0,666	0,71	0,08
Pericytes	PLCB4	ENSG00000101333	0,856	1,398153024	0,712	0,86	0,24
Pericytes	PTMS	ENSG00000159335	0,838	1,368602023	0,676	0,73	0,11
Pericytes	COL4A1	ENSG00000187498	0,823	1,909486632	0,646	0,67	0,04
Pericytes	SLC19A1	ENSG00000173638	0,824	1,459258168	0,648	0,67	0,05
Pericytes	ZEB1	ENSG00000148516	0,914	1,695229522	0,828	0,96	0,34
Pericytes	COL4A3	ENSG00000169031	0,83	1,581376316	0,66	0,7	0,08
Pericytes	SMOC2	ENSG00000112562	0,817	1,492448558	0,634	0,65	0,03
Pericytes	DOCK6	ENSG00000130158	0,822	1,313103891	0,644	0,69	0,07
Pericytes	TAGLN2	ENSG00000158710	0,818	1,276608993	0,636	0,7	0,08
Pericytes	RBMS1	ENSG00000153250	0,897	1,513887188	0,794	0,9	0,28
Pericytes	P2RY14	ENSG00000174944	0,822	1,654021685	0,644	0,67	0,05
Pericytes	COL4A4	ENSG00000081052	0,822	1,546376049	0,644	0,68	0,06
Pericytes	AXL	ENSG00000167601	0,818	1,225586264	0,636	0,71	0,09
Pericytes	GRM8	ENSG00000179603	0,832	2,047637433	0,664	0,72	0,11
Pericytes	IFITM2	ENSG00000185201	0,81	1,319133974	0,62	0,67	0,05
Pericytes	TMSB10	ENSG00000034510	0,854	1,706311196	0,708	0,82	0,2
Pericytes	LEF1	ENSG00000138795	0,814	1,345157107	0,628	0,69	0,08
Pericytes	ABCC9	ENSG00000069431	0,813	1,477072212	0,626	0,66	0,05
Pericytes	GNG11	ENSG00000127920	0,805	1,188803255	0,61	0,64	0,03
Pericytes	ETS1	ENSG00000134954	0,812	1,193594436	0,624	0,69	0,09
Pericytes	C1QTNF1	ENSG00000173918	0,804	1,244932179	0,608	0,62	0,02
Pericytes	FLT1	ENSG00000102755	0,799	1,329009137	0,598	0,68	0,08
Pericytes	ADIRF	ENSG00000148671	0,808	1,550403776	0,616	0,65	0,05
Pericytes	LGALS1	ENSG00000100097	0,863	1,648428692	0,726	0,82	0,22
Pericytes	ADAMTS9-AS2	ENSG00000241684	0,824	1,642072858	0,648	0,76	0,16
Pericytes	ADGRF5	ENSG00000069122	0,798	1,170088755	0,596	0,64	0,04
Pericytes	CACNA1C	ENSG00000151067	0,909	1,807501478	0,818	0,94	0,34
Pericytes	MGLL	ENSG00000074416	0,842	1,321423786	0,684	0,8	0,2
Pericytes	A2M	ENSG00000175899	0,809	1,16115623	0,618	0,73	0,14
Pericytes	TIMP1	ENSG00000102265	0,805	2,065761923	0,61	0,64	0,04
Pericytes	CASC15	ENSG00000272168	0,844	1,538581169	0,688	0,77	0,17

Pericytes	EHD2	ENSG00000024422	0,799	1,164014684	0,598	0,63	0,03
Pericytes	SNTB1	ENSG00000172164	0,808	1,404564089	0,616	0,69	0,1
Pericytes	JAG1	ENSG00000101384	0,806	1,356303968	0,612	0,65	0,06
Pericytes	GPC5	ENSG00000179399	0,828	1,305407381	0,656	0,86	0,28
Pericytes	CDH6	ENSG00000113361	0,812	1,501308264	0,624	0,67	0,09
Pericytes	PLCE1	ENSG00000138193	0,82	1,389706205	0,64	0,73	0,15
Pericytes	LINC02147	ENSG00000249797	0,791	1,904490248	0,582	0,6	0,03
Pericytes	PDE3A	ENSG00000172572	0,801	1,55812963	0,602	0,69	0,12
Pericytes	COL6A2	ENSG00000142173	0,789	1,153296255	0,578	0,59	0,02
Pericytes	HLA-E	ENSG00000204592	0,791	1,157605029	0,582	0,69	0,13
Pericytes	KCNE4	ENSG00000152049	0,788	1,55761556	0,576	0,6	0,03
Pericytes	KANK3	ENSG00000186994	0,787	1,062119181	0,574	0,61	0,04
Pericytes	CLDN5	ENSG00000184113	0,781	1,383390528	0,562	0,63	0,06
Pericytes	LHFPL6	ENSG00000183722	0,886	1,546003975	0,772	0,94	0,37
Pericytes	TBX2	ENSG00000121068	0,785	1,23893374	0,57	0,57	0,01
Pericytes	CYTH3	ENSG00000008256	0,82	1,296620692	0,64	0,74	0,17
Pericytes	SLC6A13	ENSG00000010379	0,787	1,469601849	0,574	0,58	0,02
Pericytes	ADAMTS9	ENSG00000163638	0,794	2,173499218	0,588	0,65	0,09
Pericytes	MAP3K20	ENSG00000091436	0,801	1,116716415	0,602	0,71	0,15
Pericytes	PAPSS2	ENSG00000198682	0,78	1,166956492	0,56	0,61	0,05
Pericytes	RHOC	ENSG00000155366	0,785	1,080777123	0,57	0,62	0,07
Pericytes	SOX5	ENSG00000134532	0,782	0,801725414	0,564	0,9	0,34
Pericytes	MSC-AS1	ENSG0000235531	0,779	1,347306446	0,558	0,56	0,01
Pericytes	YBX3	ENSG00000060138	0,787	1,149854035	0,574	0,65	0,1
Pericytes	PCBP3	ENSG00000183570	0,846	1,90998112	0,692	0,79	0,23
Pericytes	PARD3	ENSG00000148498	0,844	1,128095909	0,688	0,96	0,41
Pericytes	SPARC	ENSG00000113140	0,878	1,470331588	0,756	0,89	0,34
Pericytes	PLAC9	ENSG00000189129	0,774	1,104403217	0,548	0,55	0,01
Pericytes	GRK5	ENSG00000198873	0,811	1,343866741	0,622	0,72	0,17
Pericytes	ARHGAP6	ENSG00000047648	0,789	1,25648784	0,578	0,7	0,16
Pericytes	CNTN4	ENSG00000144619	0,771	0,89892499	0,542	0,71	0,17
Pericytes	PHLDB2	ENSG00000144824	0,773	1,084432056	0,546	0,57	0,03
Pericytes	TESC	ENSG00000088992	0,774	1,113596951	0,548	0,56	0,03
Pericytes	IFI27	ENSG00000165949	0,769	1,274750094	0,538	0,59	0,06
Pericytes	TRPC3	ENSG00000138741	0,776	1,290303933	0,552	0,57	0,04
Pericytes	TNS2	ENSG00000111077	0,807	1,105775842	0,614	0,73	0,19
Pericytes	ISYNA1	ENSG00000105655	0,775	1,263004452	0,55	0,57	0,03
Pericytes	SNRK	ENSG00000163788	0,817	1,303841668	0,634	0,74	0,21
Pericytes	ARHGEF17	ENSG00000110237	0,79	1,123962549	0,58	0,65	0,12
Pericytes	ADGRD1	ENSG00000111452	0,767	1,298842132	0,534	0,55	0,02
Pericytes	VWF	ENSG00000110799	0,764	1,317763846	0,528	0,6	0,07
Pericytes	AC012409.2	ENSG00000275443	0,769	1,426883627	0,538	0,55	0,03
Pericytes	MYOF	ENSG00000138119	0,764	0,971672767	0,528	0,63	0,1
Pericytes	MYH9	ENSG00000100345	0,835	1,201675879	0,67	0,8	0,28
Pericytes	GPER1	ENSG00000164850	0,762	1,161302228	0,524	0,53	0,01
Pericytes	PID1	ENSG00000153823	0,792	1,292353497	0,584	0,74	0,22
Pericytes	EPS8	ENSG00000151491	0,957	2,061150168	0,914	0,97	0,45
Pericytes	DACH1	ENSG00000276644	0,772	1,256352681	0,544	0,63	0,11
Pericytes	UACA	ENSG00000137831	0,832	1,250887875	0,664	0,81	0,3
Pericytes	HSPB1	ENSG00000106211	0,786	1,130402138	0,572	0,71	0,2
Pericytes	VCL	ENSG00000035403	0,788	1,102539071	0,576	0,68	0,17
Pericytes	RAPH1	ENSG00000173166	0,818	1,281003589	0,636	0,76	0,25
Pericytes	ECE1	ENSG00000117298	0,802	1,042840862	0,604	0,77	0,26
Pericytes	TMTC1	ENSG00000133687	0,784	1,141396742	0,568	0,74	0,23
Pericytes	STOM	ENSG00000148175	0,779	0,986030926	0,558	0,69	0,18

Pericytes	BSG	ENSG00000172270	0,877	1,568101709	0,754	0,88	0,37
Pericytes	RRAS	ENSG00000126458	0,759	1,029121063	0,518	0,54	0,03
Pericytes	PLOD1	ENSG00000083444	0,78	1,072298997	0,56	0,64	0,13
Pericytes	TGFBR2	ENSG00000163513	0,752	0,925902613	0,504	0,63	0,12
Pericytes	ZBTB46	ENSG00000130584	0,776	1,057565606	0,552	0,65	0,14
Pericytes	S100A11	ENSG00000163191	0,754	0,984800533	0,508	0,57	0,06
Pericytes	GJA4	ENSG00000187513	0,753	1,154313346	0,506	0,51	0
Pericytes	MFGE8	ENSG00000140545	0,774	1,043650764	0,548	0,64	0,13
Pericytes	AHNAK	ENSG00000124942	0,76	0,966276517	0,52	0,63	0,12
Pericytes	SLC9A3R2	ENSG00000065054	0,758	1,075991333	0,516	0,57	0,07
Pericytes	APOLD1	ENSG00000178878	0,792	1,114396043	0,584	0,74	0,24
Pericytes	CRIP2	ENSG00000182809	0,766	0,994736544	0,532	0,62	0,12
Pericytes	ZFHX3	ENSG00000140836	0,84	1,17715434	0,68	0,92	0,42
Pericytes	HLA-B	ENSG00000234745	0,759	0,989126184	0,518	0,65	0,15
Pericytes	DGKB	ENSG00000136267	0,761	1,01743441	0,522	0,7	0,21
Pericytes	KIRREL1	ENSG00000183853	0,756	1,054044601	0,512	0,55	0,05
Pericytes	EDNRA	ENSG00000151617	0,748	1,161094948	0,496	0,51	0,02
Pericytes	HLA-C	ENSG00000204525	0,768	1,037435321	0,536	0,65	0,16
Pericytes	BCAM	ENSG00000187244	0,747	0,941295139	0,494	0,52	0,03
Pericytes	KANK2	ENSG00000197256	0,749	0,9752534	0,498	0,53	0,04
Pericytes	EVA1B	ENSG00000142694	0,745	0,932554788	0,49	0,51	0,03
Pericytes	CYSLTR2	ENSG00000152207	0,743	1,304641751	0,486	0,49	0,01
Pericytes	NR2F1	ENSG00000175745	0,757	1,03812448	0,514	0,61	0,12
Pericytes	ITGA10	ENSG00000143127	0,747	0,969303321	0,494	0,52	0,03
Pericytes	FBLN1	ENSG00000077942	0,745	1,213624124	0,49	0,52	0,03
Pericytes	MEF2C	ENSG00000081189	0,745	0,588903279	0,49	0,72	0,24
Pericytes	EGFR	ENSG00000146648	0,741	0,819929978	0,482	0,67	0,19
Pericytes	TPM1	ENSG00000140416	0,788	1,151346812	0,576	0,71	0,23
Pericytes	GRAMD2B	ENSG00000155324	0,781	0,96673839	0,562	0,81	0,33
Pericytes	LAMA4	ENSG00000112769	0,75	1,104034318	0,5	0,6	0,12
Pericytes	CDC42EP4	ENSG00000179604	0,747	0,877995703	0,494	0,58	0,1
Pericytes	ESAM	ENSG00000149564	0,738	0,834166844	0,476	0,51	0,03
Pericytes	ZFP36L1	ENSG00000185650	0,734	0,667907714	0,468	0,7	0,22
Pericytes	S1PR3	ENSG00000213694	0,739	0,973886018	0,478	0,49	0,01
Pericytes	CD151	ENSG00000177697	0,747	0,941571202	0,494	0,54	0,06
Pericytes	SERPING1	ENSG00000149131	0,744	0,926476879	0,488	0,54	0,06
Pericytes	TRIB2	ENSG00000071575	0,747	0,925667702	0,494	0,54	0,07
Pericytes	CYBA	ENSG00000051523	0,736	0,835502407	0,472	0,55	0,08
Pericytes	PDE5A	ENSG00000138735	0,779	1,125111645	0,558	0,66	0,19
Pericytes	CSPG4	ENSG00000173546	0,738	0,920757965	0,476	0,52	0,05
Pericytes	BTG1	ENSG00000133639	0,766	1,037141379	0,532	0,64	0,16
Pericytes	EMP2	ENSG00000213853	0,737	0,87849916	0,474	0,54	0,07
Pericytes	SVIL	ENSG00000197321	0,757	1,184854557	0,514	0,6	0,13
Pericytes	CPE	ENSG00000109472	0,788	1,06933841	0,576	0,82	0,35
Pericytes	TNS1	ENSG00000079308	0,846	1,188194942	0,692	0,87	0,4
Pericytes	HEYL	ENSG00000163909	0,734	1,091222987	0,468	0,48	0,01
Pericytes	FAM107A	ENSG00000168309	0,746	1,057480788	0,492	0,61	0,15
Pericytes	SMIM3	ENSG00000256235	0,733	0,848795083	0,466	0,5	0,04
Pericytes	PDZD2	ENSG00000133401	0,816	0,899380345	0,632	0,89	0,43
Pericytes	CALD1	ENSG00000122786	0,97	2,148469232	0,94	0,98	0,52
Pericytes	DGKH	ENSG00000102780	0,757	0,978968745	0,514	0,65	0,19
Pericytes	LPL	ENSG00000175445	0,737	1,150014987	0,474	0,52	0,06
Pericytes	RGL3	ENSG00000205517	0,732	1,010323512	0,464	0,51	0,05
Pericytes	VAMP5	ENSG00000168899	0,731	0,877412899	0,462	0,51	0,05
Pericytes	FRY	ENSG00000073910	0,777	1,012831322	0,554	0,8	0,35

Pericytes	MAOA	ENSG00000189221	0,737	0,945495707	0,474	0,55	0,09
Pericytes	FSTL1	ENSG00000163430	0,735	0,975639363	0,47	0,53	0,08
Pericytes	B2M	ENSG00000166710	0,833	1,248150847	0,666	0,86	0,4
Pericytes	ANXA2	ENSG00000182718	0,728	0,867929933	0,456	0,51	0,06
Pericytes	TBX3	ENSG00000135111	0,725	0,92302553	0,45	0,47	0,02
Pericytes	TGM2	ENSG00000198959	0,722	0,965239893	0,444	0,49	0,04
Pericytes	WWTR1	ENSG00000018408	0,751	0,8817815	0,502	0,68	0,23
Pericytes	PAWR	ENSG00000177425	0,729	0,899084518	0,458	0,54	0,1
Pericytes	LAMB2	ENSG00000172037	0,726	0,835832485	0,452	0,49	0,05
Pericytes	GUCY1B1	ENSG00000061918	0,746	0,983967884	0,492	0,57	0,13
Pericytes	PMEPA1	ENSG00000124225	0,755	1,010268371	0,51	0,63	0,18
Pericytes	TMEM204	ENSG00000131634	0,722	0,798875335	0,444	0,47	0,03
Pericytes	EGFL7	ENSG00000172889	0,723	0,871308105	0,446	0,51	0,07
Pericytes	TXNIP	ENSG00000265972	0,827	1,396517566	0,654	0,85	0,4
Pericytes	UST	ENSG00000111962	0,732	0,922899031	0,464	0,65	0,2
Pericytes	GSTP1	ENSG00000084207	0,782	1,070324536	0,564	0,72	0,28
Pericytes	LRRC32	ENSG00000137507	0,718	0,888185038	0,436	0,47	0,03
Pericytes	SLC2A3	ENSG00000059804	0,733	1,030130237	0,466	0,57	0,13
Pericytes	NBL1	ENSG00000158747	0,727	0,885131592	0,454	0,49	0,05
Pericytes	CST3	ENSG00000101439	0,741	0,648708641	0,482	0,78	0,34
Pericytes	FARP1	ENSG00000152767	0,735	0,751442228	0,47	0,71	0,28
Pericytes	MT2A	ENSG00000125148	0,788	1,201829884	0,576	0,8	0,36
Pericytes	YAP1	ENSG00000137693	0,714	0,702611755	0,428	0,55	0,12
Pericytes	NTRK3	ENSG00000140538	0,739	1,18572231	0,478	0,73	0,3
Pericytes	PTEN	ENSG00000171862	0,904	1,575694807	0,808	0,94	0,51
Pericytes	ACSS3	ENSG00000111058	0,721	0,894162525	0,442	0,55	0,12
Pericytes	INPP4B	ENSG00000109452	0,755	0,999949142	0,51	0,71	0,28
Pericytes	CLIC1	ENSG00000213719	0,716	0,816689618	0,432	0,47	0,04
Pericytes	CPM	ENSG00000135678	0,82	1,396510717	0,64	0,83	0,4
Pericytes	LIN7A	ENSG00000111052	0,732	0,990400536	0,464	0,56	0,13
Pericytes	COL6A1	ENSG00000142156	0,729	0,900431285	0,458	0,54	0,12
Pericytes	FOXD1	ENSG00000251493	0,714	0,842356106	0,428	0,43	0
Pericytes	ABCB1	ENSG00000085563	0,713	1,103636554	0,426	0,5	0,07
Pericytes	RHOB	ENSG00000143878	0,788	1,111238458	0,576	0,76	0,33
Pericytes	IL1R1	ENSG00000115594	0,713	0,889719408	0,426	0,44	0,02
Pericytes	ID3	ENSG00000117318	0,71	0,837830511	0,42	0,49	0,07
Pericytes	HLA-A	ENSG00000206503	0,747	0,950039819	0,494	0,65	0,23
Pericytes	KLF6	ENSG00000067082	0,735	0,867144169	0,47	0,6	0,18
Pericytes	CAV1	ENSG00000105974	0,715	0,833510992	0,43	0,47	0,05
Pericytes	FOXC1	ENSG00000054598	0,71	0,777861541	0,42	0,44	0,02
Pericytes	MYL6	ENSG00000092841	0,758	0,991106852	0,516	0,66	0,24
Pericytes	SMTN	ENSG00000183963	0,711	0,745276964	0,422	0,49	0,07
Pericytes	FRMD6	ENSG00000139926	0,724	0,964496827	0,448	0,53	0,11
Pericytes	SORBS3	ENSG00000120896	0,725	0,865386514	0,45	0,55	0,13
Pericytes	CPT1A	ENSG00000110090	0,718	0,849001363	0,436	0,52	0,1
Pericytes	LAMC1	ENSG00000135862	0,725	1,049094864	0,45	0,54	0,12
Pericytes	PDE8B	ENSG00000113231	0,72	0,87320983	0,44	0,5	0,09
Pericytes	RBMS2	ENSG00000076067	0,718	0,755277463	0,436	0,56	0,14
Pericytes	AC002546.1	ENSG00000267653	0,706	0,839008445	0,412	0,42	0,01
Pericytes	KLHL23	ENSG00000213160	0,749	1,008819776	0,498	0,64	0,22
Pericytes	AL138828.1	ENSG00000237596	0,713	0,983633373	0,426	0,46	0,05
Pericytes	PODXL	ENSG00000128567	0,708	0,863633553	0,416	0,49	0,08
Pericytes	NXN	ENSG00000167693	0,725	0,95214352	0,45	0,58	0,17
Pericytes	CD63	ENSG00000135404	0,748	0,902117152	0,496	0,66	0,25
Pericytes	ARHGEF12	ENSG00000196914	0,816	1,024846294	0,632	0,86	0,45

Pericytes	RBMS3-AS3	ENSG00000235904	0,71	0,862028551	0,42	0,46	0,05
Pericytes	CERS6	ENSG00000172292	0,731	0,851336554	0,462	0,69	0,28
Pericytes	RORA	ENSG00000069667	0,822	1,089941873	0,644	0,97	0,57
Pericytes	JAK1	ENSG00000162434	0,785	0,930308066	0,57	0,78	0,37
Pericytes	PARVA	ENSG00000197702	0,716	0,843646641	0,432	0,5	0,1
Pericytes	CRISPLD2	ENSG00000103196	0,709	1,293821653	0,418	0,47	0,06
Pericytes	KALRN	ENSG00000160145	0,753	0,841838107	0,506	0,82	0,42
Pericytes	ADGRL2	ENSG00000117114	0,702	0,66351076	0,404	0,55	0,15
Pericytes	NOSTRIN	ENSG00000163072	0,701	0,79449528	0,402	0,44	0,04
Pericytes	PACSIN2	ENSG00000100266	0,756	0,953907575	0,512	0,7	0,29
Pericytes	ENG	ENSG00000106991	0,702	0,78240275	0,404	0,49	0,08
Pericytes	SEPTIN11	ENSG00000138758	0,734	0,879137146	0,468	0,63	0,23
Pericytes	TAGLN	ENSG00000149591	0,727	2,032769944	0,454	0,52	0,12
Pericytes	GUCY1A1	ENSG00000164116	0,712	0,920522461	0,424	0,5	0,09
Pericytes	MYO1C	ENSG00000197879	0,706	0,760531837	0,412	0,46	0,06
Pericytes	OSMR	ENSG00000145623	0,705	0,844281784	0,41	0,5	0,09
Pericytes	CEBPB	ENSG00000172216	0,704	0,739602812	0,408	0,48	0,08
Pericytes	MRC2	ENSG00000011028	0,701	0,75897503	0,402	0,46	0,06
Pericytes	AGRN	ENSG00000188157	0,717	0,936807688	0,434	0,53	0,13
Pericytes	SELENOM	ENSG00000198832	0,725	0,853485045	0,45	0,57	0,17
Pericytes	CACHD1	ENSG00000158966	0,714	0,881292336	0,428	0,62	0,22
Pericytes	NT5DC2	ENSG00000168268	0,702	0,790494509	0,404	0,44	0,04
Pericytes	MSN	ENSG00000147065	0,716	0,736508408	0,432	0,6	0,21
Pericytes	C2CD2	ENSG00000157617	0,767	1,062376422	0,534	0,73	0,33
Pericytes	CYTOR	ENSG00000222041	0,702	0,90107519	0,404	0,44	0,04
Pericytes	JUNB	ENSG00000171223	0,703	1,193365385	0,406	0,47	0,07
Pericytes	CNN3	ENSG00000117519	0,72	0,742899884	0,44	0,61	0,22
Pericytes	MECOM	ENSG00000085276	0,711	0,99902004	0,422	0,54	0,15
Pericytes	MYL12A	ENSG00000101608	0,702	0,780545082	0,404	0,47	0,08
Pericytes	HIF1A-AS3	ENSG00000258667	0,702	1,034233369	0,404	0,5	0,11
Pericytes	PRKCH	ENSG00000027075	0,701	0,850977259	0,402	0,53	0,14
Pericytes	NR2F1-AS1	ENSG00000237187	0,706	0,86998222	0,412	0,59	0,2
Pericytes	PPFIBP1	ENSG00000110841	0,773	0,890170481	0,546	0,8	0,41
Pericytes	KLF9	ENSG00000119138	0,756	0,940833501	0,512	0,71	0,33
Pericytes	PAG1	ENSG00000076641	0,754	0,997138697	0,508	0,73	0,34
Pericytes	RUNX1T1	ENSG00000079102	0,707	0,732801826	0,414	0,67	0,29
Pericytes	MSRB3	ENSG00000174099	0,703	0,753020065	0,406	0,53	0,14
Pericytes	FNDC3B	ENSG00000075420	0,736	0,816688616	0,472	0,75	0,37
Pericytes	MYL12B	ENSG00000118680	0,715	0,811764687	0,43	0,55	0,17
Pericytes	CAVIN1	ENSG00000177469	0,703	0,793176296	0,406	0,51	0,13
Pericytes	TMOD3	ENSG00000138594	0,725	0,797005743	0,45	0,62	0,24
Pericytes	ROCK2	ENSG00000134318	0,802	1,001160884	0,604	0,82	0,44
Pericytes	SLC25A6	ENSG00000169100	0,713	0,743169942	0,426	0,56	0,18
Pericytes	PDZRN3	ENSG00000121440	0,702	1,019199853	0,404	0,56	0,18
Pericytes	TLN1	ENSG00000137076	0,713	0,725952928	0,426	0,57	0,19
Pericytes	CRIM1	ENSG00000150938	0,723	1,023145503	0,446	0,63	0,25
Pericytes	ITGB5	ENSG00000082781	0,708	0,815516355	0,416	0,56	0,19
Pericytes	CYB5R3	ENSG00000100243	0,734	0,847771287	0,468	0,63	0,26
Pericytes	AKAP13	ENSG00000170776	0,717	0,598644718	0,434	0,73	0,36
Pericytes	PTN	ENSG00000105894	0,728	0,859674486	0,456	0,7	0,33
Pericytes	KLF12	ENSG00000118922	0,722	0,802452514	0,444	0,73	0,36
Pericytes	STK24	ENSG00000102572	0,714	0,749467453	0,428	0,59	0,21
Pericytes	RELL1	ENSG00000181826	0,706	0,735269731	0,412	0,56	0,19
Pericytes	ITGB1	ENSG00000150093	0,767	0,892735406	0,534	0,75	0,38
Pericytes	TPT1	ENSG00000133112	0,764	0,935406667	0,528	0,77	0,4

Pericytes	SEPTIN9	ENSG00000184640	0,701	0,72823818	0,402	0,54	0,19
Pericytes	PFN1	ENSG00000108518	0,709	0,796185581	0,418	0,57	0,22
Pericytes	MAST4	ENSG00000069020	0,752	0,911129152	0,504	0,79	0,44
Pericytes	DENND2A	ENSG00000146966	0,721	0,885820314	0,442	0,67	0,32
Pericytes	SERF2	ENSG00000140264	0,778	0,997454292	0,556	0,78	0,44
Pericytes	BACH1	ENSG00000156273	0,71	0,725606386	0,42	0,68	0,34
Pericytes	TMSB4X	ENSG00000205542	0,794	1,088113558	0,588	0,84	0,5
Pericytes	RASAL2	ENSG00000075391	0,785	0,937653521	0,57	0,86	0,52
Pericytes	SLC6A1	ENSG00000157103	0,791	1,264738865	0,582	0,82	0,48
Pericytes	TLE5	ENSG00000104964	0,726	0,765780216	0,452	0,68	0,35
Pericytes	RHOA	ENSG00000067560	0,736	0,806804044	0,472	0,7	0,37
Pericytes	TPM4	ENSG00000167460	0,715	0,848953149	0,43	0,62	0,29
Pericytes	H3F3B	ENSG00000132475	0,774	0,923238759	0,548	0,8	0,48
Pericytes	NBEAL1	ENSG00000144426	0,821	1,034906515	0,642	0,88	0,56
Pericytes	SH3RF1	ENSG00000154447	0,725	0,833659813	0,45	0,75	0,43
Pericytes	TUBA1B	ENSG00000123416	0,713	0,718759255	0,426	0,7	0,39
Pericytes	TSC22D1	ENSG00000102804	0,77	0,915204099	0,54	0,83	0,53
Pericytes	CFLAR	ENSG00000003402	0,711	0,653658941	0,422	0,7	0,4
Pericytes	SLC38A2	ENSG00000134294	0,753	0,903779535	0,506	0,81	0,52
Pericytes	UBA2	ENSG00000126261	0,706	0,726337355	0,412	0,65	0,36
Pericytes	CD81	ENSG00000110651	0,735	0,73331625	0,47	0,8	0,52
Pericytes	TACC1	ENSG00000147526	0,866	1,16267375	0,732	0,94	0,66
Pericytes	EEF1A1	ENSG00000156508	0,732	0,851414064	0,464	0,75	0,48
Pericytes	TXNRD1	ENSG00000198431	0,714	0,727460591	0,428	0,7	0,43
Pericytes	PTPN12	ENSG00000127947	0,709	0,664534797	0,418	0,71	0,44
Pericytes	PTPRM	ENSG00000173482	0,72	0,792923412	0,44	0,76	0,5
Pericytes	CAST	ENSG00000153113	0,704	0,655608614	0,408	0,7	0,43
Pericytes	ESYT2	ENSG00000117868	0,705	0,76208513	0,41	0,69	0,42
Pericytes	DLC1	ENSG00000164741	0,935	1,915572047	0,87	0,97	0,71
Pericytes	RAC1	ENSG00000136238	0,708	0,662855641	0,416	0,72	0,46
Pericytes	ZBTB38	ENSG00000177311	0,736	0,765365753	0,472	0,77	0,52
Pericytes	EIF1	ENSG00000173812	0,718	0,686179259	0,436	0,78	0,53
Pericytes	SLC20A2	ENSG00000168575	0,738	1,022555412	0,476	0,81	0,57
Pericytes	UBC	ENSG00000150991	0,715	0,645400165	0,43	0,79	0,55
Pericytes	NCKAP1	ENSG00000061676	0,701	0,592175291	0,402	0,75	0,52
Pericytes	LPP	ENSG00000145012	0,855	1,193702009	0,71	0,95	0,72
Pericytes	SLC12A2	ENSG00000064651	0,724	0,840523695	0,448	0,81	0,6
Pericytes	PTPRK	ENSG00000152894	0,725	0,655706151	0,45	0,95	0,75
Pericytes	BTBD9	ENSG00000183826	0,72	0,890933455	0,44	0,81	0,61
Pericytes	PTMA	ENSG00000187514	0,724	0,67728879	0,448	0,86	0,66
Pericytes	SPTBN1	ENSG00000115306	0,824	0,862875255	0,648	0,94	0,75
Pericytes	SGIP1	ENSG00000118473	0,718	0,906342271	0,436	0,87	0,7
Pericytes	GAPDH	ENSG00000111640	0,747	0,796488991	0,494	0,87	0,7
Pericytes	KIAA1109	ENSG00000138688	0,753	0,802901763	0,506	0,87	0,7
Pericytes	ACTB	ENSG00000075624	0,797	1,124369314	0,594	0,92	0,77
Pericytes	MALAT1	ENSG00000251562	0,858	0,799895867	0,716	1	1
Inhibitory	GAD2	ENSG00000136750	0,923	1,397908849	0,846	0,88	0,03
Inhibitory	SRRM3	ENSG00000177679	0,918	1,103087194	0,836	0,96	0,12
Inhibitory	GRIN1	ENSG00000176884	0,921	1,161657744	0,842	0,95	0,11
Inhibitory	PAK3	ENSG00000077264	0,935	1,501764919	0,87	0,97	0,13
Inhibitory	CELF4	ENSG00000101489	0,93	1,446027046	0,86	0,96	0,12
Inhibitory	MYT1L	ENSG00000186487	0,936	1,615282481	0,872	0,98	0,15
Inhibitory	SYT1	ENSG00000067715	0,935	1,876918171	0,87	0,97	0,15
Inhibitory	ANKRD30BL	ENSG00000163046	0,925	1,424473661	0,85	0,96	0,15
Inhibitory	SYN2	ENSG00000157152	0,907	1,189138171	0,814	0,94	0,13

Inhibitory	GRIN2B	ENSG00000273079	0,901	1,170996221	0,802	0,94	0,14
Inhibitory	ATP8A2	ENSG00000132932	0,935	1,588379993	0,87	0,98	0,18
Inhibitory	LINGO2	ENSG00000174482	0,909	1,842833173	0,818	0,91	0,11
Inhibitory	MEG8	ENSG00000225746	0,916	1,382592735	0,832	0,94	0,14
Inhibitory	CACNA1B	ENSG00000148408	0,915	1,350261751	0,83	0,96	0,16
Inhibitory	SLC4A10	ENSG00000144290	0,907	1,240655264	0,814	0,93	0,13
Inhibitory	RIMS2	ENSG00000176406	0,934	1,717285279	0,868	0,99	0,19
Inhibitory	GRIA1	ENSG00000155511	0,908	1,629677278	0,816	0,91	0,11
Inhibitory	GABBR2	ENSG00000136928	0,896	1,071317696	0,792	0,91	0,11
Inhibitory	RAB3C	ENSG00000152932	0,907	1,293398766	0,814	0,93	0,14
Inhibitory	STMN2	ENSG00000104435	0,9	1,214506105	0,8	0,88	0,09
Inhibitory	GAD1	ENSG00000128683	0,899	1,273719137	0,798	0,85	0,07
Inhibitory	SCN2A	ENSG00000136531	0,914	1,180738096	0,828	0,96	0,18
Inhibitory	CELF5	ENSG00000161082	0,891	0,903235428	0,782	0,89	0,11
Inhibitory	KSR2	ENSG00000171435	0,905	1,217203155	0,81	0,94	0,16
Inhibitory	CALY	ENSG00000130643	0,898	1,300353501	0,796	0,88	0,1
Inhibitory	GABRB3	ENSG00000166206	0,899	1,185011986	0,798	0,95	0,17
Inhibitory	NMNAT2	ENSG00000157064	0,895	1,074049043	0,79	0,92	0,15
Inhibitory	DLGAP2	ENSG00000198010	0,894	1,375908015	0,788	0,9	0,13
Inhibitory	CHD5	ENSG00000116254	0,884	0,798762583	0,768	0,86	0,08
Inhibitory	GABRG2	ENSG00000113327	0,885	0,927703919	0,77	0,86	0,08
Inhibitory	DNM1	ENSG00000106976	0,901	1,020386023	0,802	0,93	0,16
Inhibitory	LRFN5	ENSG00000165379	0,904	1,439328787	0,808	0,94	0,17
Inhibitory	CDH18	ENSG00000145526	0,907	1,741167107	0,814	0,93	0,16
Inhibitory	SCN3A	ENSG00000153253	0,882	0,992108952	0,764	0,92	0,15
Inhibitory	CAMK2B	ENSG00000058404	0,877	0,85566681	0,754	0,92	0,16
Inhibitory	SYN3	ENSG00000185666	0,891	1,359065841	0,782	0,9	0,13
Inhibitory	FGF12	ENSG00000114279	0,908	1,497652419	0,816	0,97	0,21
Inhibitory	XKR4	ENSG00000206579	0,88	1,206421068	0,76	0,92	0,16
Inhibitory	IQSEC3	ENSG00000120645	0,88	0,822636302	0,76	0,84	0,08
Inhibitory	SNAP25	ENSG00000132639	0,916	1,306283357	0,832	0,97	0,21
Inhibitory	PTPRN2	ENSG00000155093	0,92	1,421382435	0,84	0,97	0,21
Inhibitory	CADPS	ENSG00000163618	0,918	1,441645933	0,836	0,98	0,22
Inhibitory	SHANK2	ENSG00000162105	0,874	0,926367262	0,748	0,91	0,15
Inhibitory	ARHGAP44	ENSG00000006740	0,895	1,072561869	0,79	0,93	0,18
Inhibitory	TMEM130	ENSG00000166448	0,877	0,816011651	0,754	0,84	0,08
Inhibitory	PRKAR1B	ENSG00000188191	0,887	0,898521826	0,774	0,91	0,15
Inhibitory	MAST1	ENSG00000105613	0,876	0,801430087	0,752	0,85	0,1
Inhibitory	RUNDC3B	ENSG00000105784	0,869	0,860614013	0,738	0,89	0,14
Inhibitory	FRRS1L	ENSG00000260230	0,885	0,885738038	0,77	0,91	0,16
Inhibitory	ELAVL4	ENSG00000162374	0,876	0,898241692	0,752	0,86	0,11
Inhibitory	ROBO2	ENSG00000185008	0,915	1,947208306	0,83	0,95	0,2
Inhibitory	TENM2	ENSG00000145934	0,909	1,939476844	0,818	0,93	0,18
Inhibitory	NRG1	ENSG00000157168	0,888	1,989118635	0,776	0,88	0,13
Inhibitory	JPH4	ENSG00000092051	0,869	0,765404704	0,738	0,85	0,1
Inhibitory	STXBP5L	ENSG00000145087	0,907	1,415515203	0,814	0,96	0,22
Inhibitory	DNAH14	ENSG00000185842	0,884	1,128068536	0,768	0,89	0,14
Inhibitory	UNC80	ENSG00000144406	0,87	0,960477595	0,74	0,95	0,21
Inhibitory	MIAT	ENSG00000225783	0,864	0,828659603	0,728	0,87	0,12
Inhibitory	EPHA6	ENSG00000080224	0,881	1,615982851	0,762	0,85	0,11
Inhibitory	HECW1	ENSG00000002746	0,863	1,026793938	0,726	0,88	0,14
Inhibitory	WNK2	ENSG00000165238	0,862	0,801954029	0,724	0,9	0,16
Inhibitory	SCN1A	ENSG00000144285	0,876	1,133632597	0,752	0,92	0,18
Inhibitory	PNMA2	ENSG00000240694	0,874	0,909834707	0,748	0,84	0,1
Inhibitory	TUSC3	ENSG00000104723	0,87	0,85947116	0,74	0,86	0,13

Inhibitory	AMPH	ENSG00000078053	0,87	0,950499396	0,74	0,87	0,14
Inhibitory	SYT16	ENSG00000139973	0,87	0,992562711	0,74	0,88	0,15
Inhibitory	CACNA1E	ENSG00000198216	0,866	1,002141388	0,732	0,87	0,14
Inhibitory	NSG2	ENSG00000170091	0,864	0,834742378	0,728	0,86	0,12
Inhibitory	UNC5D	ENSG00000156687	0,878	1,770226423	0,756	0,86	0,12
Inhibitory	GALNT17	ENSG00000185274	0,865	1,068898433	0,73	0,86	0,13
Inhibitory	NHS	ENSG00000188158	0,87	1,037517622	0,74	0,87	0,14
Inhibitory	DCLK1	ENSG00000133083	0,87	1,042346879	0,74	0,96	0,23
Inhibitory	SPTBN4	ENSG00000160460	0,903	1,130729588	0,806	0,96	0,23
Inhibitory	NEXMIF	ENSG00000050030	0,864	0,933752822	0,728	0,86	0,13
Inhibitory	SRRM4	ENSG00000139767	0,86	0,8637414	0,72	0,81	0,08
Inhibitory	OPCML	ENSG00000183715	0,85	1,049931609	0,7	0,93	0,2
Inhibitory	SYBU	ENSG00000147642	0,855	0,812093721	0,71	0,89	0,17
Inhibitory	KCNB2	ENSG00000182674	0,859	1,327553616	0,718	0,8	0,08
Inhibitory	AGBL4	ENSG00000186094	0,901	1,37057013	0,802	0,97	0,25
Inhibitory	FGF14	ENSG00000102466	0,895	1,40444024	0,79	0,99	0,27
Inhibitory	ATCAY	ENSG00000167654	0,857	0,741221493	0,714	0,85	0,12
Inhibitory	SNTG1	ENSG00000147481	0,862	1,428111885	0,724	0,9	0,18
Inhibitory	ELAVL2	ENSG00000107105	0,862	1,019827293	0,724	0,79	0,07
Inhibitory	REEP1	ENSG00000068615	0,859	0,780515137	0,718	0,84	0,12
Inhibitory	GRM7	ENSG00000196277	0,872	1,314210616	0,744	0,93	0,21
Inhibitory	SV2A	ENSG00000159164	0,872	0,924774382	0,744	0,88	0,16
Inhibitory	GRIP1	ENSG00000155974	0,909	1,812895749	0,818	0,94	0,22
Inhibitory	VSNL1	ENSG00000163032	0,86	0,960322804	0,72	0,8	0,09
Inhibitory	BASP1	ENSG00000176788	0,861	0,894255046	0,722	0,91	0,2
Inhibitory	SCN8A	ENSG00000196876	0,877	0,980368003	0,754	0,92	0,2
Inhibitory	ATP2B2	ENSG00000157087	0,852	0,851437559	0,704	0,92	0,21
Inhibitory	TENM3	ENSG00000218336	0,875	1,36597033	0,75	0,9	0,19
Inhibitory	GABRG3	ENSG00000182256	0,862	1,626489973	0,724	0,79	0,08
Inhibitory	ADD2	ENSG00000075340	0,852	0,723632421	0,704	0,81	0,1
Inhibitory	GRM5	ENSG00000168959	0,86	1,259528945	0,72	0,9	0,19
Inhibitory	DOK6	ENSG00000206052	0,861	1,196311109	0,722	0,86	0,15
Inhibitory	CCSER1	ENSG00000184305	0,92	1,593809097	0,84	0,98	0,27
Inhibitory	OSBPL6	ENSG00000079156	0,858	0,922246959	0,716	0,93	0,23
Inhibitory	SNRPN	ENSG00000128739	0,91	1,340384	0,82	0,95	0,25
Inhibitory	SUSD4	ENSG00000143502	0,843	0,727688198	0,686	0,82	0,12
Inhibitory	GRIK2	ENSG00000164418	0,923	1,690183704	0,846	0,98	0,28
Inhibitory	ANK1	ENSG00000029534	0,849	0,90097065	0,698	0,79	0,09
Inhibitory	NOL4	ENSG00000101746	0,839	0,805841303	0,678	0,9	0,2
Inhibitory	FRMPD4	ENSG00000169933	0,852	1,205309007	0,704	0,86	0,16
Inhibitory	GAP43	ENSG00000172020	0,847	0,863428352	0,694	0,8	0,1
Inhibitory	SLC35F1	ENSG00000196376	0,836	0,765124243	0,672	0,91	0,22
Inhibitory	CACNA1A	ENSG00000141837	0,858	0,944166512	0,716	0,96	0,26
Inhibitory	CLSTN3	ENSG00000139182	0,845	0,666068914	0,69	0,79	0,09
Inhibitory	MTUS2	ENSG00000132938	0,848	1,292901538	0,696	0,8	0,11
Inhibitory	ATRNL1	ENSG00000107518	0,828	0,798592829	0,656	0,9	0,2
Inhibitory	KCNC2	ENSG00000166006	0,845	1,185218603	0,69	0,77	0,08
Inhibitory	THY1	ENSG00000154096	0,847	0,841032462	0,694	0,8	0,11
Inhibitory	ZNF385D	ENSG00000151789	0,856	1,494525072	0,712	0,82	0,12
Inhibitory	KCNJ3	ENSG00000162989	0,838	0,906611325	0,676	0,83	0,14
Inhibitory	KCNC1	ENSG00000129159	0,845	0,669656256	0,69	0,78	0,09
Inhibitory	BTBD11	ENSG00000151136	0,839	0,862978323	0,678	0,8	0,11
Inhibitory	RIMBP2	ENSG00000060709	0,848	0,802025713	0,696	0,83	0,14
Inhibitory	PLCB4	ENSG00000101333	0,843	0,930028884	0,686	0,92	0,23
Inhibitory	CACNG2	ENSG00000166862	0,842	0,756705235	0,684	0,79	0,1

Inhibitory	PLPPR4	ENSG00000117600	0,838	0,658868444	0,676	0,8	0,11
Inhibitory	MYH10	ENSG00000133026	0,847	0,770543287	0,694	0,87	0,18
Inhibitory	SLC12A5	ENSG00000124140	0,845	0,689128543	0,69	0,76	0,07
Inhibitory	RPH3A	ENSG00000089169	0,844	0,969802767	0,688	0,77	0,08
Inhibitory	BICDL1	ENSG00000135127	0,842	0,693943328	0,684	0,8	0,12
Inhibitory	RIMS1	ENSG00000079841	0,877	1,096066305	0,754	0,95	0,26
Inhibitory	VAT1L	ENSG00000171724	0,852	0,985925217	0,704	0,83	0,14
Inhibitory	GABRB1	ENSG00000163288	0,859	1,116021983	0,718	0,93	0,25
Inhibitory	GABRB2	ENSG00000145864	0,844	1,191585426	0,688	0,78	0,1
Inhibitory	SGCZ	ENSG00000185053	0,86	1,674728273	0,72	0,86	0,17
Inhibitory	ADAM23	ENSG00000114948	0,846	0,868605947	0,692	0,89	0,2
Inhibitory	NRSN1	ENSG00000152954	0,839	0,675950697	0,678	0,77	0,09
Inhibitory	KIAA1549L	ENSG00000110427	0,833	0,801089812	0,666	0,81	0,13
Inhibitory	STXBP5-AS1	ENSG00000233452	0,847	1,063369731	0,694	0,83	0,15
Inhibitory	SLC41A2	ENSG00000136052	0,836	0,716423378	0,672	0,82	0,14
Inhibitory	CNTNAP5	ENSG00000155052	0,848	1,334142491	0,696	0,86	0,17
Inhibitory	SORBS2	ENSG00000154556	0,84	0,895388351	0,68	0,9	0,22
Inhibitory	KLHL29	ENSG00000119771	0,838	0,838466676	0,676	0,81	0,13
Inhibitory	MAP7D2	ENSG00000184368	0,839	0,645305241	0,678	0,76	0,08
Inhibitory	DPY19L2	ENSG00000177990	0,835	0,708174251	0,67	0,83	0,16
Inhibitory	OLFM3	ENSG00000118733	0,844	1,11639857	0,688	0,8	0,13
Inhibitory	SLC8A3	ENSG00000100678	0,828	0,685829891	0,656	0,81	0,13
Inhibitory	FHOD3	ENSG00000134775	0,839	0,892411753	0,678	0,82	0,14
Inhibitory	GNB5	ENSG00000069966	0,832	0,613790787	0,664	0,81	0,14
Inhibitory	FAM155A	ENSG00000204442	0,92	1,666660882	0,84	1	0,32
Inhibitory	PTPRN	ENSG00000054356	0,837	0,671626395	0,674	0,75	0,07
Inhibitory	CACNB2	ENSG00000165995	0,862	1,116516623	0,724	0,95	0,27
Inhibitory	GUCY1A2	ENSG00000152402	0,841	1,004564084	0,682	0,86	0,19
Inhibitory	KIF5A	ENSG00000155980	0,838	0,648924338	0,676	0,79	0,12
Inhibitory	PLXNA4	ENSG00000221866	0,834	0,908346883	0,668	0,81	0,14
Inhibitory	PRICKLE1	ENSG00000139174	0,831	0,834727785	0,662	0,79	0,11
Inhibitory	ELMOD1	ENSG00000110675	0,827	0,672459681	0,654	0,82	0,14
Inhibitory	PPM1E	ENSG00000175175	0,87	1,038778265	0,74	0,94	0,26
Inhibitory	DSCAM	ENSG00000171587	0,852	0,793775248	0,704	0,97	0,3
Inhibitory	CNTN4	ENSG00000144619	0,848	1,413660015	0,696	0,83	0,16
Inhibitory	KHDRBS2	ENSG00000112232	0,857	1,19826517	0,714	0,9	0,23
Inhibitory	DGKB	ENSG00000136267	0,844	1,170541804	0,688	0,87	0,2
Inhibitory	SYN1	ENSG00000008056	0,834	0,671378849	0,668	0,75	0,08
Inhibitory	SLC24A3	ENSG00000185052	0,828	0,949229612	0,656	0,82	0,15
Inhibitory	TMEM196	ENSG00000173452	0,836	0,757045053	0,672	0,73	0,06
Inhibitory	PTPRR	ENSG00000153233	0,834	0,939827042	0,668	0,75	0,08
Inhibitory	PAM	ENSG00000145730	0,852	1,082241382	0,704	0,93	0,26
Inhibitory	CLVS1	ENSG00000177182	0,826	0,689423136	0,652	0,83	0,17
Inhibitory	CNNM1	ENSG00000119946	0,829	0,592547848	0,658	0,75	0,08
Inhibitory	LRRTM4	ENSG00000176204	0,883	1,560585365	0,766	0,95	0,28
Inhibitory	ABLIM2	ENSG00000163995	0,834	0,73292776	0,668	0,85	0,19
Inhibitory	TENM1	ENSG00000009694	0,84	1,148241861	0,68	0,79	0,13
Inhibitory	SLC38A1	ENSG00000111371	0,819	0,630666083	0,638	0,88	0,22
Inhibitory	SLC8A1	ENSG00000183023	0,814	0,759841661	0,628	0,95	0,28
Inhibitory	CNTN5	ENSG00000149972	0,847	1,800391895	0,694	0,79	0,13
Inhibitory	LHFPL4	ENSG00000156959	0,823	0,585387039	0,646	0,78	0,12
Inhibitory	RYR2	ENSG00000198626	0,886	1,421852643	0,772	0,93	0,27
Inhibitory	RALYL	ENSG00000184672	0,841	1,25587784	0,682	0,9	0,24
Inhibitory	HS6ST3	ENSG00000185352	0,842	1,442066043	0,684	0,84	0,19
Inhibitory	ASIC2	ENSG00000108684	0,837	1,293497585	0,674	0,79	0,13

Inhibitory	RNF175	ENSG00000145428	0,826	0,620386427	0,652	0,74	0,09
Inhibitory	SIDT1	ENSG00000072858	0,823	0,691161446	0,646	0,76	0,1
Inhibitory	C11orf80	ENSG00000173715	0,823	0,643993765	0,646	0,81	0,15
Inhibitory	STXBP1	ENSG00000136854	0,873	0,877591398	0,746	0,94	0,29
Inhibitory	RALGPS2	ENSG00000116191	0,816	0,660407037	0,632	0,84	0,18
Inhibitory	ZNF804A	ENSG00000170396	0,833	1,332745368	0,666	0,79	0,13
Inhibitory	DLGAP1	ENSG00000170579	0,857	1,230465092	0,714	0,93	0,27
Inhibitory	KCNH7	ENSG00000184611	0,831	1,274707872	0,662	0,76	0,1
Inhibitory	SNAP91	ENSG00000065609	0,887	1,067245227	0,774	0,97	0,32
Inhibitory	HSPA4L	ENSG00000164070	0,832	0,727358956	0,664	0,84	0,19
Inhibitory	SYP	ENSG00000102003	0,83	0,68455285	0,66	0,85	0,2
Inhibitory	SLC2A13	ENSG00000151229	0,821	0,867318017	0,642	0,88	0,23
Inhibitory	CNTN1	ENSG00000018236	0,808	0,755257716	0,616	0,98	0,32
Inhibitory	KCND2	ENSG00000184408	0,808	0,770919252	0,616	0,93	0,27
Inhibitory	EPHA5	ENSG00000145242	0,828	1,055605463	0,656	0,75	0,09
Inhibitory	RBFOX3	ENSG00000167281	0,823	0,728881982	0,646	0,73	0,08
Inhibitory	SEZ6L	ENSG00000100095	0,813	0,837740692	0,626	0,81	0,16
Inhibitory	EPB41L4B	ENSG00000095203	0,825	0,726715165	0,65	0,77	0,12
Inhibitory	SLC44A5	ENSG00000137968	0,83	1,150342245	0,66	0,79	0,14
Inhibitory	ANO5	ENSG00000171714	0,812	0,611031978	0,624	0,79	0,14
Inhibitory	CSMD2	ENSG00000121904	0,826	0,844968183	0,652	0,9	0,25
Inhibitory	INA	ENSG00000148798	0,825	0,691027456	0,65	0,71	0,06
Inhibitory	GNAL	ENSG00000141404	0,818	0,703495804	0,636	0,78	0,13
Inhibitory	MDGA2	ENSG00000139915	0,873	1,247088384	0,746	0,98	0,34
Inhibitory	LRRC7	ENSG00000033122	0,858	1,126787265	0,716	0,94	0,3
Inhibitory	NAP1L3	ENSG00000186310	0,826	0,708274419	0,652	0,76	0,12
Inhibitory	SCN7A	ENSG00000136546	0,821	0,738566027	0,642	0,72	0,07
Inhibitory	DENND1B	ENSG00000213047	0,809	0,614360383	0,618	0,81	0,16
Inhibitory	EFNA5	ENSG00000184349	0,819	1,037187884	0,638	0,79	0,14
Inhibitory	ADGRL2	ENSG00000117114	0,823	1,168856954	0,646	0,78	0,13
Inhibitory	TRERF1	ENSG00000124496	0,81	0,64224479	0,62	0,81	0,17
Inhibitory	RNF150	ENSG00000170153	0,799	0,612670477	0,598	0,87	0,23
Inhibitory	PCDH7	ENSG00000169851	0,841	1,223159193	0,682	0,91	0,27
Inhibitory	AC073050.1	ENSG00000228222	0,827	1,094393444	0,654	0,81	0,17
Inhibitory	ASTN1	ENSG00000152092	0,813	0,690379015	0,626	0,91	0,27
Inhibitory	SYT14	ENSG00000143469	0,831	0,745663131	0,662	0,91	0,27
Inhibitory	FP700111.1	ENSG00000224363	0,826	0,764814838	0,652	0,9	0,26
Inhibitory	PRR16	ENSG00000184838	0,818	1,054337686	0,636	0,73	0,09
Inhibitory	AC092683.1	ENSG00000230606	0,888	1,095017179	0,776	0,96	0,32
Inhibitory	CSMD3	ENSG00000164796	0,875	1,301400114	0,75	0,97	0,33
Inhibitory	TMEM59L	ENSG00000105696	0,817	0,681121866	0,634	0,76	0,12
Inhibitory	KIAA1211	ENSG00000109265	0,816	0,760794592	0,632	0,85	0,21
Inhibitory	PODXL2	ENSG00000114631	0,816	0,592125932	0,632	0,82	0,18
Inhibitory	CAP2	ENSG00000112186	0,812	0,62225382	0,624	0,76	0,12
Inhibitory	TSPYL1	ENSG00000189241	0,819	0,665194455	0,638	0,76	0,13
Inhibitory	LINC00599	ENSG00000253230	0,816	0,597439322	0,632	0,72	0,08
Inhibitory	GABRA2	ENSG00000151834	0,814	0,762712277	0,628	0,73	0,1
Inhibitory	CAMK4	ENSG00000152495	0,813	0,758696983	0,626	0,76	0,13
Inhibitory	KCTD16	ENSG00000183775	0,832	1,127332933	0,664	0,84	0,21
Inhibitory	CACNA1D	ENSG00000157388	0,817	0,699645657	0,634	0,91	0,27
Inhibitory	PAK5	ENSG00000101349	0,804	0,590516517	0,608	0,75	0,12
Inhibitory	HCN1	ENSG00000164588	0,817	1,081787809	0,634	0,71	0,08
Inhibitory	SPTAN1	ENSG00000197694	0,838	0,777238692	0,676	0,94	0,31
Inhibitory	PCSK1N	ENSG00000102109	0,836	1,143816	0,672	0,84	0,21
Inhibitory	CIT	ENSG00000122966	0,811	0,606386437	0,622	0,83	0,2

Inhibitory	PWRN1	ENSG00000259905	0,814	0,742698781	0,628	0,88	0,26
Inhibitory	RGS17	ENSG00000091844	0,813	0,699702243	0,626	0,73	0,1
Inhibitory	TSPYL2	ENSG00000184205	0,81	0,621790103	0,62	0,8	0,17
Inhibitory	JAKMIP1	ENSG00000152969	0,808	0,614610455	0,616	0,72	0,1
Inhibitory	NYAP2	ENSG00000144460	0,808	0,626023842	0,616	0,7	0,07
Inhibitory	EPHB1	ENSG00000154928	0,82	0,886315092	0,64	0,8	0,17
Inhibitory	EPB41	ENSG00000159023	0,799	0,590256943	0,598	0,82	0,19
Inhibitory	PCDH15	ENSG00000150275	0,808	1,151708947	0,616	0,81	0,18
Inhibitory	CACNA1C	ENSG00000151067	0,86	0,982182814	0,72	0,96	0,33
Inhibitory	CERS6	ENSG00000172292	0,799	0,654044099	0,598	0,9	0,27
Inhibitory	CNKSR2	ENSG00000149970	0,796	0,678234012	0,592	0,8	0,18
Inhibitory	ANKRD34C-AS1	ENSG00000259234	0,807	0,702402537	0,614	0,69	0,07
Inhibitory	PCLO	ENSG00000186472	0,897	1,195580292	0,794	0,97	0,35
Inhibitory	LONRF2	ENSG00000170500	0,819	0,741615047	0,638	0,86	0,25
Inhibitory	NDRG4	ENSG00000103034	0,803	0,637434458	0,606	0,75	0,13
Inhibitory	SCG2	ENSG00000171951	0,805	0,901597428	0,61	0,7	0,09
Inhibitory	GALNTL6	ENSG00000174473	0,811	1,531383094	0,622	0,74	0,13
Inhibitory	PGM2L1	ENSG00000165434	0,798	0,586661053	0,596	0,72	0,11
Inhibitory	BRINP1	ENSG00000078725	0,792	0,664313409	0,584	0,77	0,16
Inhibitory	NRG3	ENSG00000185737	0,818	0,865418522	0,636	0,98	0,38
Inhibitory	XKR6	ENSG00000171044	0,874	1,080168463	0,748	0,96	0,36
Inhibitory	DAAM1	ENSG00000100592	0,807	0,62545302	0,614	0,91	0,31
Inhibitory	KCNJ6	ENSG00000157542	0,795	0,684701719	0,59	0,7	0,1
Inhibitory	MAP2	ENSG00000078018	0,908	1,262956737	0,816	0,99	0,39
Inhibitory	TMEM132B	ENSG00000139364	0,797	0,806746456	0,594	0,76	0,16
Inhibitory	SEZ6L2	ENSG00000174938	0,808	0,659791481	0,616	0,84	0,24
Inhibitory	SAMD5	ENSG00000203727	0,802	1,081841077	0,604	0,67	0,07
Inhibitory	FBXL2	ENSG00000153558	0,796	0,604574873	0,592	0,85	0,24
Inhibitory	ADCY1	ENSG00000164742	0,792	0,599426349	0,584	0,75	0,14
Inhibitory	GPC6	ENSG00000183098	0,821	1,468298647	0,642	0,8	0,2
Inhibitory	PATJ	ENSG00000132849	0,819	0,788143299	0,638	0,9	0,3
Inhibitory	FRY	ENSG00000073910	0,795	0,663852359	0,59	0,93	0,34
Inhibitory	MPP6	ENSG00000105926	0,793	0,657550084	0,586	0,74	0,15
Inhibitory	NDST3	ENSG00000164100	0,797	0,906800784	0,594	0,68	0,08
Inhibitory	SNCG	ENSG00000173267	0,797	0,730569437	0,594	0,67	0,08
Inhibitory	RAPGEF4	ENSG00000091428	0,805	0,70426135	0,61	0,92	0,33
Inhibitory	AFF2	ENSG00000155966	0,797	0,875558596	0,594	0,67	0,07
Inhibitory	KCNIP4	ENSG00000185774	0,819	1,23998003	0,638	0,87	0,27
Inhibitory	VPS13A	ENSG00000197969	0,793	0,586151107	0,586	0,87	0,28
Inhibitory	PIP5K1B	ENSG00000107242	0,787	0,59102149	0,574	0,74	0,14
Inhibitory	EML6	ENSG00000214595	0,784	0,629050424	0,568	0,82	0,22
Inhibitory	GRM1	ENSG00000152822	0,797	1,010832377	0,594	0,68	0,08
Inhibitory	KIAA0825	ENSG00000185261	0,793	0,653278166	0,586	0,87	0,28
Inhibitory	KCNQ5	ENSG00000185760	0,801	1,625554606	0,602	0,67	0,08
Inhibitory	LINC02389	ENSG00000255693	0,795	0,702492662	0,59	0,67	0,08
Inhibitory	TMEM178B	ENSG00000261115	0,865	1,046041374	0,73	0,96	0,37
Inhibitory	ERC2	ENSG00000187672	0,836	0,967397707	0,672	0,93	0,34
Inhibitory	EEF1A2	ENSG00000101210	0,798	0,661161819	0,596	0,7	0,11
Inhibitory	TMEM108	ENSG00000144868	0,782	0,626305544	0,564	0,85	0,26
Inhibitory	SGSM1	ENSG00000167037	0,796	0,625693084	0,592	0,88	0,29
Inhibitory	UCHL1	ENSG00000154277	0,811	0,772175085	0,622	0,86	0,27
Inhibitory	VSTM2A	ENSG00000170419	0,795	0,640372607	0,59	0,64	0,06
Inhibitory	BEND6	ENSG00000151917	0,789	0,606728012	0,578	0,73	0,14
Inhibitory	SHISA9	ENSG00000237515	0,785	0,854564228	0,57	0,75	0,16

Inhibitory	AL033504.1	ENSG00000227681	0,794	1,068356182	0,588	0,65	0,07
Inhibitory	BAIAP3	ENSG00000007516	0,789	0,703725622	0,578	0,68	0,09
Inhibitory	LINC00632	ENSG00000203930	0,872	0,983036755	0,744	0,95	0,36
Inhibitory	PACRG	ENSG00000112530	0,788	0,693893988	0,576	0,81	0,22
Inhibitory	GABRA1	ENSG00000022355	0,792	0,788009771	0,584	0,65	0,07
Inhibitory	LINC01122	ENSG00000233723	0,783	0,720976516	0,566	0,78	0,2
Inhibitory	DAB1	ENSG00000173406	0,873	1,374225675	0,746	0,95	0,37
Inhibitory	AFF3	ENSG00000144218	0,828	0,848989304	0,656	0,95	0,36
Inhibitory	PLCXD3	ENSG00000182836	0,788	0,823298858	0,576	0,68	0,1
Inhibitory	PCSK2	ENSG00000125851	0,782	0,721793257	0,564	0,69	0,11
Inhibitory	GPR158	ENSG00000151025	0,781	0,655627662	0,562	0,88	0,3
Inhibitory	CCDC85A	ENSG00000055813	0,78	0,704837887	0,56	0,73	0,14
Inhibitory	BRINP3	ENSG00000162670	0,783	0,863121488	0,566	0,78	0,2
Inhibitory	RAB27B	ENSG00000041353	0,787	0,681203621	0,574	0,65	0,07
Inhibitory	FAM189A1	ENSG00000104059	0,783	0,61656823	0,566	0,68	0,1
Inhibitory	CSMD1	ENSG00000183117	0,862	1,208347276	0,724	0,96	0,38
Inhibitory	PRKG1	ENSG00000185532	0,799	1,141678735	0,598	0,82	0,24
Inhibitory	MIR137HG	ENSG00000225206	0,789	0,937050277	0,578	0,67	0,09
Inhibitory	LY6H	ENSG00000176956	0,787	0,67699362	0,574	0,65	0,07
Inhibitory	CSRNP3	ENSG00000178662	0,844	0,855791063	0,688	0,95	0,37
Inhibitory	AC025159.1	ENSG00000257815	0,809	0,892567737	0,618	0,87	0,3
Inhibitory	EPHA7	ENSG00000135333	0,783	0,814235887	0,566	0,66	0,09
Inhibitory	NRXN1	ENSG00000179915	0,826	0,9549001	0,652	0,99	0,42
Inhibitory	SLC35F4	ENSG00000151812	0,786	1,080945425	0,572	0,64	0,07
Inhibitory	RANBP17	ENSG00000204764	0,781	0,649056774	0,562	0,86	0,29
Inhibitory	FAAH2	ENSG00000165591	0,78	0,593010027	0,56	0,72	0,16
Inhibitory	NELL2	ENSG00000184613	0,772	0,662350851	0,544	0,7	0,13
Inhibitory	SCN9A	ENSG00000169432	0,774	0,678715056	0,548	0,71	0,14
Inhibitory	LINC01414	ENSG00000253554	0,782	0,895161395	0,564	0,66	0,09
Inhibitory	FLRT2	ENSG00000185070	0,773	0,757354018	0,546	0,76	0,2
Inhibitory	ZFYVE9	ENSG00000157077	0,79	0,587965924	0,58	0,89	0,33
Inhibitory	FAT3	ENSG00000165323	0,765	0,697816077	0,53	0,77	0,21
Inhibitory	FOCAD	ENSG00000188352	0,836	0,901211492	0,672	0,94	0,38
Inhibitory	SLC25A12	ENSG00000115840	0,814	0,715639321	0,628	0,91	0,35
Inhibitory	KIAA1217	ENSG00000120549	0,782	0,981066822	0,564	0,8	0,24
Inhibitory	LIN7A	ENSG00000111052	0,77	0,585167011	0,54	0,68	0,13
Inhibitory	LINC01250	ENSG00000234423	0,777	0,591919153	0,554	0,63	0,08
Inhibitory	KLHL1	ENSG00000150361	0,773	0,882366615	0,546	0,65	0,1
Inhibitory	PCNX2	ENSG00000135749	0,804	0,639704512	0,608	0,94	0,39
Inhibitory	INPP4B	ENSG00000109452	0,809	1,117520692	0,618	0,83	0,28
Inhibitory	CHRM2	ENSG00000181072	0,775	1,14696824	0,55	0,61	0,06
Inhibitory	RGS7	ENSG00000182901	0,831	1,042741003	0,662	0,96	0,41
Inhibitory	SLC22A17	ENSG00000092096	0,799	0,7092015	0,598	0,88	0,33
Inhibitory	IDS	ENSG00000010404	0,816	0,799009776	0,632	0,89	0,34
Inhibitory	PCDH11X	ENSG00000102290	0,783	1,441847982	0,566	0,65	0,1
Inhibitory	MCF2L2	ENSG00000053524	0,8	0,693422173	0,6	0,91	0,36
Inhibitory	KIFAP3	ENSG00000075945	0,815	0,70150334	0,63	0,92	0,37
Inhibitory	TENM4	ENSG00000149256	0,807	0,875324106	0,614	0,9	0,35
Inhibitory	MAGI3	ENSG00000081026	0,772	0,614709142	0,544	0,85	0,31
Inhibitory	HSPA12A	ENSG00000165868	0,818	0,752969966	0,636	0,92	0,38
Inhibitory	CALM3	ENSG00000160014	0,773	0,612167635	0,546	0,77	0,23
Inhibitory	BEX1	ENSG00000133169	0,77	0,632974569	0,54	0,64	0,1
Inhibitory	MARCH11	ENSG00000183654	0,773	0,612612118	0,546	0,59	0,05
Inhibitory	SORCS3	ENSG00000156395	0,763	0,768088123	0,526	0,68	0,14
Inhibitory	PLEKHA5	ENSG00000052126	0,802	0,698636277	0,604	0,96	0,42

Inhibitory	TMTC1	ENSG00000133687	0,754	0,586473116	0,508	0,77	0,23
Inhibitory	NEFL	ENSG00000277586	0,77	0,614900383	0,54	0,6	0,06
Inhibitory	YWHAG	ENSG00000170027	0,783	0,604144863	0,566	0,89	0,35
Inhibitory	PTPRO	ENSG00000151490	0,765	0,885093642	0,53	0,65	0,11
Inhibitory	KIF26B	ENSG00000162849	0,762	0,776723642	0,524	0,74	0,2
Inhibitory	PEG10	ENSG00000242265	0,765	0,733380884	0,53	0,67	0,14
Inhibitory	DCC	ENSG00000187323	0,778	1,178689617	0,556	0,77	0,24
Inhibitory	FGF13	ENSG00000129682	0,761	0,850179742	0,522	0,61	0,08
Inhibitory	DGKI	ENSG00000157680	0,802	0,78458939	0,604	0,92	0,39
Inhibitory	CLSTN2	ENSG00000158258	0,764	0,871855219	0,528	0,65	0,12
Inhibitory	AC090578.1	ENSG00000253553	0,76	0,632594494	0,52	0,62	0,1
Inhibitory	RUNX1T1	ENSG00000079102	0,752	0,599287068	0,504	0,81	0,28
Inhibitory	ADAM22	ENSG00000008277	0,793	0,663909384	0,586	0,91	0,39
Inhibitory	KCNH5	ENSG00000140015	0,761	0,760245231	0,522	0,6	0,07
Inhibitory	NSF	ENSG00000073969	0,856	0,922774397	0,712	0,96	0,44
Inhibitory	UNC79	ENSG00000133958	0,831	0,817328033	0,662	0,96	0,43
Inhibitory	CTNNA2	ENSG00000066032	0,784	0,750675418	0,568	0,98	0,45
Inhibitory	UNC13C	ENSG00000137766	0,76	0,930225392	0,52	0,6	0,08
Inhibitory	CNTN3	ENSG00000113805	0,749	0,600378802	0,498	0,69	0,17
Inhibitory	MGAT4C	ENSG00000182050	0,757	1,009028254	0,514	0,71	0,19
Inhibitory	KALRN	ENSG00000160145	0,763	0,591984388	0,526	0,93	0,41
Inhibitory	AC120193.1	ENSG00000253535	0,759	1,003448804	0,518	0,63	0,12
Inhibitory	PTCHD4	ENSG00000244694	0,755	0,764093843	0,51	0,59	0,08
Inhibitory	FSTL5	ENSG00000168843	0,772	1,075541621	0,544	0,74	0,22
Inhibitory	VWC2L	ENSG00000174453	0,754	0,701724206	0,508	0,58	0,06
Inhibitory	GRM8	ENSG00000179603	0,757	1,059625104	0,514	0,62	0,11
Inhibitory	SCN1A-AS1	ENSG00000236107	0,753	0,691597659	0,506	0,59	0,08
Inhibitory	UBA6-AS1	ENSG00000248049	0,776	0,727422578	0,552	0,89	0,39
Inhibitory	CACNA2D3	ENSG00000157445	0,748	0,743877261	0,496	0,78	0,28
Inhibitory	PTPRT	ENSG00000196090	0,752	1,165129105	0,504	0,64	0,14
Inhibitory	CDH4	ENSG00000179242	0,757	0,856863091	0,514	0,71	0,22
Inhibitory	TRPC5	ENSG00000072315	0,746	0,702327319	0,492	0,58	0,08
Inhibitory	MCTP1	ENSG00000175471	0,739	0,623524658	0,478	0,66	0,16
Inhibitory	DPP10	ENSG00000175497	0,754	0,732469881	0,508	0,8	0,31
Inhibitory	TAFA2	ENSG00000198673	0,748	0,893919966	0,496	0,7	0,21
Inhibitory	GRIN3A	ENSG00000198785	0,745	0,630432596	0,49	0,55	0,06
Inhibitory	CNTN6	ENSG00000134115	0,745	0,681287378	0,49	0,58	0,08
Inhibitory	NXPH1	ENSG00000122584	0,745	1,249871182	0,49	0,61	0,11
Inhibitory	MYO16	ENSG00000041515	0,75	0,96508435	0,5	0,59	0,11
Inhibitory	RIT2	ENSG00000152214	0,74	0,790470423	0,48	0,59	0,1
Inhibitory	ZNF385B	ENSG00000144331	0,738	0,799711423	0,476	0,59	0,1
Inhibitory	ST6GALNAC5	ENSG00000117069	0,743	1,138927966	0,486	0,54	0,06
Inhibitory	LUZP2	ENSG00000187398	0,728	0,686209141	0,456	0,73	0,24
Inhibitory	ZMAT4	ENSG00000165061	0,739	0,885234803	0,478	0,55	0,07
Inhibitory	GRIN2A	ENSG00000183454	0,737	0,861093227	0,474	0,62	0,14
Inhibitory	FRMD4A	ENSG00000151474	0,832	0,834691255	0,664	0,98	0,5
Inhibitory	HTR2C	ENSG00000147246	0,741	1,324997194	0,482	0,55	0,08
Inhibitory	CDH12	ENSG00000154162	0,757	1,46445356	0,514	0,65	0,17
Inhibitory	ZFPM2	ENSG00000169946	0,747	0,802255189	0,494	0,76	0,28
Inhibitory	PREPL	ENSG00000138078	0,815	0,71226972	0,63	0,94	0,47
Inhibitory	GRIA3	ENSG00000125675	0,743	0,629124702	0,486	0,81	0,34
Inhibitory	CPNE4	ENSG00000196353	0,731	0,708902701	0,462	0,56	0,09
Inhibitory	THSD7A	ENSG00000005108	0,728	0,639084663	0,456	0,74	0,27
Inhibitory	AC233296.1	ENSG00000280870	0,742	0,733442457	0,484	0,81	0,34
Inhibitory	CHRM3	ENSG00000133019	0,735	1,04545439	0,47	0,54	0,07

Inhibitory	SLIT2	ENSG00000145147	0,731	0,789192033	0,462	0,66	0,19
Inhibitory	MPPED2	ENSG00000066382	0,727	0,605157786	0,454	0,65	0,19
Inhibitory	GRID2	ENSG00000152208	0,848	1,294900442	0,696	0,96	0,5
Inhibitory	ZNF804B	ENSG00000182348	0,733	1,201914001	0,466	0,54	0,08
Inhibitory	DANT2	ENSG00000235244	0,79	0,683248501	0,58	0,94	0,48
Inhibitory	RBFOX1	ENSG00000078328	0,795	1,236704788	0,59	0,91	0,45
Inhibitory	HS6ST2	ENSG00000171004	0,727	0,623112734	0,454	0,54	0,08
Inhibitory	DYNC1I1	ENSG00000158560	0,789	0,691993606	0,578	0,96	0,5
Inhibitory	SEMA6D	ENSG00000137872	0,737	0,876412526	0,474	0,73	0,27
Inhibitory	VWC2	ENSG00000188730	0,721	0,588096734	0,442	0,55	0,1
Inhibitory	SLC35F3	ENSG00000183780	0,713	0,605319691	0,426	0,74	0,29
Inhibitory	MACROD2	ENSG00000172264	0,795	0,794784511	0,59	0,98	0,53
Inhibitory	CUX2	ENSG00000111249	0,72	0,676735194	0,44	0,52	0,08
Inhibitory	DENND5B	ENSG00000170456	0,779	0,612499857	0,558	0,95	0,5
Inhibitory	CEP112	ENSG00000154240	0,715	0,59922875	0,43	0,71	0,26
Inhibitory	KCNT2	ENSG00000162687	0,714	0,609211295	0,428	0,57	0,13
Inhibitory	TAFA1	ENSG00000183662	0,715	0,906919816	0,43	0,58	0,14
Inhibitory	GRIK1	ENSG00000171189	0,716	0,938906971	0,432	0,58	0,15
Inhibitory	NELL1	ENSG00000165973	0,718	1,170079019	0,436	0,5	0,07
Inhibitory	NLGN1	ENSG00000169760	0,78	0,779300502	0,56	0,98	0,54
Inhibitory	ANKRD30B	ENSG00000180777	0,716	0,612700257	0,432	0,47	0,04
Inhibitory	LDB2	ENSG00000169744	0,711	0,718556445	0,422	0,6	0,18
Inhibitory	TMEM132D	ENSG00000151952	0,707	0,690964702	0,414	0,56	0,13
Inhibitory	ZFHX3	ENSG00000140836	0,722	0,699366013	0,444	0,84	0,42
Inhibitory	PCDH11Y	ENSG00000099715	0,713	1,121597	0,426	0,51	0,08
Inhibitory	FRAS1	ENSG00000138759	0,707	0,647815308	0,414	0,49	0,07
Inhibitory	CNTNAP2	ENSG00000174469	0,86	1,256891233	0,72	0,99	0,57
Inhibitory	SDK1	ENSG00000146555	0,705	0,70876211	0,41	0,67	0,26
Inhibitory	GULP1	ENSG00000144366	0,706	0,800482515	0,412	0,5	0,09
Inhibitory	LRRC4C	ENSG00000148948	0,811	0,819884656	0,622	0,96	0,54
Inhibitory	PPFIA2	ENSG00000139220	0,795	0,74314529	0,59	0,97	0,56
Inhibitory	SYNE1	ENSG00000131018	0,786	0,585089578	0,572	0,96	0,56
Inhibitory	COL25A1	ENSG00000188517	0,701	0,843559511	0,402	0,49	0,08
Inhibitory	TTTY14	ENSG00000176728	0,745	0,744464688	0,49	0,81	0,41
Inhibitory	GRIA4	ENSG00000152578	0,771	0,742107738	0,542	0,94	0,54
Inhibitory	CDH13	ENSG00000140945	0,704	0,890473727	0,408	0,59	0,2
Inhibitory	HDAC9	ENSG00000048052	0,726	0,613625587	0,452	0,88	0,48
Inhibitory	NBEA	ENSG00000172915	0,87	1,100140504	0,74	0,97	0,58
Inhibitory	AC124312.1	ENSG00000214265	0,89	1,011345238	0,78	0,98	0,6
Inhibitory	ROBO1	ENSG00000169855	0,869	1,518487213	0,738	0,96	0,58
Inhibitory	SMYD3	ENSG00000185420	0,781	0,704915435	0,562	0,96	0,6
Inhibitory	NEGR1	ENSG00000172260	0,817	1,046426234	0,634	0,98	0,62
Inhibitory	GNAS	ENSG00000087460	0,887	1,30332537	0,774	0,98	0,63
Inhibitory	NRXN3	ENSG00000021645	0,896	1,380081004	0,792	0,98	0,63
Inhibitory	KAZN	ENSG00000189337	0,851	1,108212135	0,702	0,98	0,66
Inhibitory	MAP1B	ENSG00000131711	0,81	0,77881359	0,62	0,98	0,67
Inhibitory	NOVA1	ENSG00000139910	0,774	0,670371029	0,548	0,97	0,67
Inhibitory	AHI1	ENSG00000135541	0,851	0,856484916	0,702	0,98	0,68
Inhibitory	PDE4D	ENSG00000113448	0,775	0,820152719	0,55	0,97	0,69
Inhibitory	ANKS1B	ENSG00000185046	0,748	0,608692262	0,496	0,98	0,75
Inhibitory	CAMTA1	ENSG00000171735	0,797	0,611284782	0,594	0,98	0,75
Inhibitory	ADGRB3	ENSG00000135298	0,787	0,72157357	0,574	0,99	0,8
Inhibitory	TNRC6A	ENSG00000090905	0,811	0,632008386	0,622	0,99	0,83
Ependymal	ARMC3	ENSG00000165309	0,988	2,454235417	0,976	0,98	0,06
Ependymal	VWA3A	ENSG00000175267	0,978	2,331218792	0,956	0,96	0,05

Ependymal	SPAG17	ENSG00000155761	0,97	2,649520041	0,94	0,94	0,04
Ependymal	ADGB	ENSG00000118492	0,953	2,436900377	0,906	0,91	0,01
Ependymal	CFAP299	ENSG00000197826	0,969	3,628812551	0,938	0,94	0,04
Ependymal	LMNTD1	ENSG00000152936	0,963	2,26270833	0,926	0,94	0,04
Ependymal	ZBBX	ENSG00000169064	0,959	2,258773969	0,918	0,92	0,03
Ependymal	CFAP157	ENSG00000160401	0,942	2,170514203	0,884	0,88	0,01
Ependymal	LRRIQ1	ENSG00000133640	0,966	2,333707195	0,932	0,94	0,07
Ependymal	DNAH11	ENSG00000105877	0,956	2,66537352	0,912	0,92	0,04
Ependymal	CFAP43	ENSG00000197748	0,979	2,760995413	0,958	0,97	0,09
Ependymal	CFAP47	ENSG00000165164	0,967	2,518603335	0,934	0,94	0,07
Ependymal	DTHD1	ENSG00000197057	0,942	2,230561005	0,884	0,89	0,02
Ependymal	WDR49	ENSG00000174776	0,97	2,12616289	0,94	0,98	0,11
Ependymal	SPATA17	ENSG00000162814	0,949	2,138778585	0,898	0,91	0,04
Ependymal	FRMPD2	ENSG00000170324	0,949	1,814280637	0,898	0,93	0,07
Ependymal	DNAH7	ENSG00000118997	0,981	2,4804796	0,962	0,98	0,12
Ependymal	TOGARAM2	ENSG00000189350	0,964	1,940382989	0,928	0,96	0,11
Ependymal	LRRC9	ENSG00000131951	0,951	2,038646185	0,902	0,91	0,06
Ependymal	CFAP73	ENSG00000186710	0,929	1,73835261	0,858	0,86	0,01
Ependymal	DNAAF1	ENSG00000154099	0,944	1,98066704	0,888	0,9	0,04
Ependymal	CFAP54	ENSG00000188596	0,99	3,074637821	0,98	0,99	0,14
Ependymal	DCDC1	ENSG00000170959	0,964	2,64179765	0,928	0,94	0,09
Ependymal	TEKT1	ENSG00000167858	0,928	1,71393145	0,856	0,86	0,02
Ependymal	TTC6	ENSG00000139865	0,94	2,100981861	0,88	0,9	0,06
Ependymal	TTC29	ENSG00000137473	0,933	2,139794918	0,866	0,87	0,03
Ependymal	KIAA2012	ENSG00000182329	0,935	1,888336335	0,87	0,88	0,04
Ependymal	DNAH5	ENSG00000039139	0,933	2,120426539	0,866	0,88	0,04
Ependymal	SPAG6	ENSG00000077327	0,937	1,934474035	0,874	0,89	0,05
Ependymal	ARMC4	ENSG00000169126	0,924	1,772874942	0,848	0,85	0,02
Ependymal	DNAH6	ENSG00000115423	0,97	2,523769593	0,94	0,95	0,12
Ependymal	C8orf34	ENSG00000165084	0,963	2,418997373	0,926	0,95	0,12
Ependymal	DNAI1	ENSG00000122735	0,927	1,785511969	0,854	0,86	0,03
Ependymal	MAP3K19	ENSG00000176601	0,922	1,818669682	0,844	0,85	0,03
Ependymal	CFAP46	ENSG00000171811	0,945	1,996554482	0,89	0,9	0,08
Ependymal	SERPINI2	ENSG00000114204	0,926	1,604873337	0,852	0,91	0,09
Ependymal	CFAP52	ENSG00000166596	0,921	1,8231822	0,842	0,85	0,03
Ependymal	RSPH1	ENSG00000160188	0,925	1,712179681	0,85	0,86	0,04
Ependymal	VWA3B	ENSG00000168658	0,916	1,616118577	0,832	0,84	0,02
Ependymal	PPP1R42	ENSG00000178125	0,928	1,684137626	0,856	0,88	0,06
Ependymal	NEK5	ENSG00000197168	0,931	1,813790684	0,862	0,88	0,07
Ependymal	C6orf118	ENSG00000112539	0,911	1,48257634	0,822	0,83	0,02
Ependymal	CCDC173	ENSG00000154479	0,912	1,578378327	0,824	0,83	0,03
Ependymal	FANK1	ENSG00000203780	0,942	1,899606156	0,884	0,91	0,1
Ependymal	WDR63	ENSG00000162643	0,905	1,557775845	0,81	0,82	0,02
Ependymal	SLC47A2	ENSG00000180638	0,902	1,639678802	0,804	0,81	0,02
Ependymal	DNAH12	ENSG00000174844	0,938	2,27628678	0,876	0,9	0,11
Ependymal	CFAP61	ENSG00000089101	0,926	1,665951057	0,852	0,88	0,09
Ependymal	CFAP100	ENSG00000163885	0,906	1,575898509	0,812	0,82	0,03
Ependymal	CCDC170	ENSG00000120262	0,907	1,562882606	0,814	0,83	0,05
Ependymal	ROPN1L	ENSG00000145491	0,897	1,522130663	0,794	0,8	0,01
Ependymal	DNAH9	ENSG00000007174	0,967	3,062582906	0,934	0,95	0,17
Ependymal	CFAP221	ENSG00000163075	0,922	1,675093487	0,844	0,87	0,09
Ependymal	LINC01088	ENSG00000249307	0,934	1,864973971	0,868	0,98	0,21
Ependymal	CASC1	ENSG00000118307	0,91	1,710271311	0,82	0,84	0,06
Ependymal	LRGUK	ENSG00000155530	0,905	1,552172179	0,81	0,83	0,06
Ependymal	FAM227A	ENSG00000184949	0,918	1,534906397	0,836	0,87	0,09

Ependymal	FHAD1	ENSG00000142621	0,895	1,622421223	0,79	0,8	0,03
Ependymal	CASC2	ENSG00000177640	0,931	1,806731392	0,862	0,91	0,14
Ependymal	CFAP44	ENSG00000206530	0,97	2,247711058	0,94	0,96	0,19
Ependymal	CCDC40	ENSG00000141519	0,911	1,416005647	0,822	0,87	0,1
Ependymal	DRC3	ENSG00000171962	0,891	1,367044917	0,782	0,8	0,03
Ependymal	DNAH3	ENSG00000158486	0,889	1,66909286	0,778	0,79	0,02
Ependymal	MOK	ENSG00000080823	0,941	1,718603796	0,882	0,92	0,16
Ependymal	RFX2	ENSG00000087903	0,941	1,808324488	0,882	0,93	0,18
Ependymal	CAPS	ENSG00000105519	0,921	2,059121696	0,842	0,88	0,13
Ependymal	PLCH1	ENSG00000114805	0,899	1,499587693	0,798	0,86	0,11
Ependymal	DLEC1	ENSG00000008226	0,882	1,367451258	0,764	0,78	0,03
Ependymal	SPEF2	ENSG00000152582	0,935	1,885036549	0,87	0,9	0,15
Ependymal	DOC2A	ENSG00000149927	0,893	1,563109568	0,786	0,81	0,06
Ependymal	TMEM67	ENSG00000164953	0,914	1,570526022	0,828	0,86	0,11
Ependymal	AL357093.2	ENSG00000258752	0,875	1,490493984	0,75	0,75	0,01
Ependymal	LRRC6	ENSG00000129295	0,915	1,553965198	0,83	0,86	0,12
Ependymal	CFAP69	ENSG00000105792	0,912	1,5683774	0,824	0,86	0,11
Ependymal	RGS22	ENSG00000132554	0,889	1,611392284	0,778	0,81	0,07
Ependymal	WDR78	ENSG00000152763	0,933	1,715816911	0,866	0,91	0,17
Ependymal	C4orf47	ENSG00000205129	0,908	1,507988604	0,816	0,86	0,12
Ependymal	DNAI2	ENSG00000171595	0,871	1,436523874	0,742	0,75	0,01
Ependymal	AL022068.1	ENSG00000228412	0,901	1,800782344	0,802	0,86	0,12
Ependymal	PPIL6	ENSG00000185250	0,891	1,406977731	0,782	0,81	0,08
Ependymal	SPAG8	ENSG00000137098	0,876	1,355107529	0,752	0,77	0,03
Ependymal	PACRG	ENSG00000112530	0,962	2,044212863	0,924	0,97	0,23
Ependymal	ANKFN1	ENSG00000153930	0,905	1,431795043	0,81	0,93	0,19
Ependymal	DNAH10	ENSG00000197653	0,891	1,57662468	0,782	0,8	0,07
Ependymal	LINC02055	ENSG00000254101	0,875	1,980722426	0,75	0,77	0,04
Ependymal	EFHC2	ENSG00000183690	0,873	1,357166171	0,746	0,77	0,04
Ependymal	SOX6	ENSG00000110693	0,887	1,208972419	0,774	0,96	0,23
Ependymal	IQCA1	ENSG00000132321	0,9	1,37229954	0,8	0,9	0,17
Ependymal	EFCAB6	ENSG00000186976	0,919	1,751597302	0,838	0,88	0,15
Ependymal	EYA1	ENSG00000104313	0,875	1,517407545	0,75	0,82	0,1
Ependymal	YAP1	ENSG00000137693	0,871	1,205971198	0,742	0,85	0,12
Ependymal	DRC1	ENSG00000157856	0,871	1,189835292	0,742	0,76	0,03
Ependymal	GLIS3	ENSG00000107249	0,927	1,725164399	0,854	0,98	0,25
Ependymal	TCTEX1D1	ENSG00000152760	0,874	1,322203643	0,748	0,76	0,04
Ependymal	CFAP70	ENSG00000156042	0,939	1,829896505	0,878	0,93	0,21
Ependymal	SLC9C2	ENSG00000162753	0,869	1,319979384	0,738	0,76	0,04
Ependymal	DCDC2	ENSG00000146038	0,87	1,412524163	0,74	0,76	0,04
Ependymal	SHANK2	ENSG00000162105	0,908	1,655712857	0,816	0,89	0,17
Ependymal	AGBL4	ENSG00000186094	0,969	2,276763903	0,938	0,98	0,27
Ependymal	NEK10	ENSG00000163491	0,912	1,740348959	0,824	0,87	0,16
Ependymal	CDHR3	ENSG00000128536	0,896	1,75010446	0,792	0,87	0,15
Ependymal	SPAG1	ENSG00000104450	0,893	1,393255369	0,786	0,84	0,13
Ependymal	COL21A1	ENSG00000124749	0,878	1,468494985	0,756	0,83	0,12
Ependymal	STK33	ENSG00000130413	0,957	1,814424295	0,914	0,97	0,26
Ependymal	DNAH2	ENSG00000183914	0,861	1,288241123	0,722	0,73	0,02
Ependymal	NEK11	ENSG00000114670	0,967	2,093772275	0,934	0,96	0,25
Ependymal	DAW1	ENSG00000123977	0,86	1,248497263	0,72	0,73	0,02
Ependymal	WDR66	ENSG00000158023	0,886	1,462330014	0,772	0,81	0,1
Ependymal	CASC15	ENSG00000272168	0,914	1,775291255	0,828	0,89	0,18
Ependymal	MAATS1	ENSG00000183833	0,881	1,302565411	0,762	0,81	0,11
Ependymal	ADCY2	ENSG00000078295	0,888	1,325373449	0,776	0,95	0,25
Ependymal	CFAP77	ENSG00000188523	0,854	1,195258162	0,708	0,72	0,01

Ependymal	PAMR1	ENSG00000149090	0,85	1,211651764	0,7	0,8	0,1
Ependymal	TTC34	ENSG00000215912	0,854	1,300244938	0,708	0,72	0,02
Ependymal	SPATA6L	ENSG00000106686	0,892	1,384545764	0,784	0,84	0,14
Ependymal	EFCAB1	ENSG00000034239	0,852	1,155345504	0,704	0,71	0,01
Ependymal	TEX9	ENSG00000151575	0,887	1,420646416	0,774	0,84	0,15
Ependymal	C1orf87	ENSG00000162598	0,847	1,37886183	0,694	0,7	0,01
Ependymal	EYA4	ENSG00000112319	0,909	1,836432886	0,818	0,88	0,2
Ependymal	WWC1	ENSG00000113645	0,86	1,280490855	0,72	0,83	0,15
Ependymal	TRDN	ENSG00000186439	0,845	1,570802447	0,69	0,71	0,03
Ependymal	RFX4	ENSG00000111783	0,825	0,885825619	0,65	0,82	0,15
Ependymal	PLTP	ENSG00000100979	0,841	1,14856394	0,682	0,72	0,05
Ependymal	ACOT11	ENSG00000162390	0,864	1,114580896	0,728	0,88	0,2
Ependymal	CEP126	ENSG00000110318	0,941	1,715389292	0,882	0,94	0,27
Ependymal	VWA5B1	ENSG00000158816	0,84	1,18725514	0,68	0,69	0,03
Ependymal	ARMC2	ENSG00000118690	0,913	1,405724083	0,826	0,9	0,24
Ependymal	IQCG	ENSG00000114473	0,868	1,310550311	0,736	0,8	0,13
Ependymal	NRG4	ENSG00000169752	0,852	1,312685997	0,704	0,74	0,07
Ependymal	BMPR1B	ENSG00000138696	0,829	1,073727368	0,658	0,78	0,12
Ependymal	NELL2	ENSG00000184613	0,849	1,241022635	0,698	0,8	0,14
Ependymal	SNTB1	ENSG00000172164	0,836	1,124525927	0,672	0,77	0,11
Ependymal	CCDC60	ENSG00000183273	0,833	1,480096039	0,666	0,67	0,01
Ependymal	CCDC30	ENSG00000186409	0,959	2,070016522	0,918	0,96	0,3
Ependymal	GYG2	ENSG00000056998	0,842	1,179066984	0,684	0,73	0,08
Ependymal	ARHGEF26	ENSG00000114790	0,85	1,123192947	0,7	0,82	0,17
Ependymal	GMPR	ENSG00000137198	0,833	1,153444947	0,666	0,75	0,1
Ependymal	CD36	ENSG00000135218	0,832	1,280499201	0,664	0,68	0,03
Ependymal	IQCH	ENSG00000103599	0,849	1,252289848	0,698	0,75	0,1
Ependymal	CFAP65	ENSG00000181378	0,828	1,044006038	0,656	0,66	0,01
Ependymal	TTLL9	ENSG00000131044	0,869	1,233911488	0,738	0,82	0,17
Ependymal	ADGRA3	ENSG00000152990	0,842	1,112629945	0,684	0,81	0,16
Ependymal	AC013470.2	ENSG00000226690	0,827	1,122063689	0,654	0,66	0,01
Ependymal	IL16	ENSG00000172349	0,829	1,059777987	0,658	0,69	0,04
Ependymal	TMEM232	ENSG00000186952	0,968	2,657294612	0,936	0,97	0,32
Ependymal	RNF213-AS1	ENSG00000263069	0,868	1,362037888	0,736	0,81	0,17
Ependymal	CCDC114	ENSG00000105479	0,823	1,031768051	0,646	0,65	0,01
Ependymal	BAIAP3	ENSG00000007516	0,835	1,130441119	0,67	0,75	0,1
Ependymal	RPGR	ENSG00000156313	0,862	1,141540969	0,724	0,84	0,2
Ependymal	BBOF1	ENSG00000119636	0,852	1,104141155	0,704	0,77	0,13
Ependymal	AK8	ENSG00000165695	0,832	1,182689304	0,664	0,7	0,06
Ependymal	ULK4	ENSG00000168038	0,948	2,281681137	0,896	0,94	0,3
Ependymal	SNCAIP	ENSG00000064692	0,832	1,112367295	0,664	0,73	0,09
Ependymal	DYNLRB2	ENSG00000168589	0,824	1,037597299	0,648	0,66	0,03
Ependymal	IQGAP2	ENSG00000145703	0,819	1,1737774	0,638	0,67	0,04
Ependymal	FBXL13	ENSG00000161040	0,859	1,36415391	0,718	0,79	0,16
Ependymal	MLF1	ENSG00000178053	0,841	1,097757984	0,682	0,74	0,11
Ependymal	EFHC1	ENSG00000096093	0,886	1,329083454	0,772	0,85	0,22
Ependymal	NPHP1	ENSG00000144061	0,851	1,106812861	0,702	0,78	0,15
Ependymal	CFAP53	ENSG00000172361	0,828	1,040011948	0,656	0,69	0,06
Ependymal	GABRG1	ENSG00000163285	0,828	1,117115634	0,656	0,74	0,11
Ependymal	AGBL1	ENSG00000273540	0,849	2,198136071	0,698	0,74	0,11
Ependymal	AP001831.1	ENSG00000254733	0,835	1,20430455	0,67	0,72	0,09
Ependymal	EFCAB12	ENSG00000172771	0,819	0,929371492	0,638	0,65	0,02
Ependymal	KIAA0319	ENSG00000137261	0,835	1,047999666	0,67	0,78	0,15
Ependymal	USP2-AS1	ENSG00000245248	0,821	1,222721476	0,642	0,66	0,03
Ependymal	PLCB4	ENSG00000101333	0,835	1,070906809	0,67	0,87	0,25

Ependymal	MNS1	ENSG00000138587	0,823	1,03852148	0,646	0,68	0,05
Ependymal	EZR	ENSG00000092820	0,813	0,921621389	0,626	0,72	0,09
Ependymal	CRB1	ENSG00000134376	0,911	1,641764319	0,822	0,92	0,3
Ependymal	ARMH1	ENSG00000198520	0,83	1,094163774	0,66	0,74	0,12
Ependymal	ROR2	ENSG00000169071	0,816	1,323982413	0,632	0,66	0,04
Ependymal	CFAP45	ENSG00000213085	0,812	0,94604214	0,624	0,63	0
Ependymal	USP43	ENSG00000154914	0,817	1,096758709	0,634	0,66	0,04
Ependymal	FZD3	ENSG00000104290	0,857	1,234451807	0,714	0,82	0,2
Ependymal	MYLK3	ENSG00000140795	0,812	1,257308618	0,624	0,63	0,01
Ependymal	CLIC6	ENSG00000159212	0,812	1,204604862	0,624	0,63	0,01
Ependymal	CCDC146	ENSG00000135205	0,94	1,775868277	0,88	0,94	0,33
Ependymal	SOX5	ENSG00000134532	0,859	1,179129609	0,718	0,97	0,35
Ependymal	TRMT9B	ENSG00000250305	0,831	1,068429725	0,662	0,76	0,14
Ependymal	GJA1	ENSG00000152661	0,796	0,824219976	0,592	0,76	0,14
Ependymal	C8orf37-AS1	ENSG00000253773	0,822	1,374250045	0,644	0,71	0,1
Ependymal	CCDC81	ENSG00000149201	0,812	1,049029113	0,624	0,65	0,04
Ependymal	NEBL	ENSG00000078114	0,87	1,258221256	0,74	0,98	0,36
Ependymal	C5AR1	ENSG00000197405	0,811	1,01141145	0,622	0,65	0,04
Ependymal	KIAA0825	ENSG00000185261	0,905	1,655306789	0,81	0,9	0,29
Ependymal	C5orf49	ENSG00000215217	0,81	0,987130317	0,62	0,63	0,02
Ependymal	NWD1	ENSG00000188039	0,807	1,118671908	0,614	0,68	0,07
Ependymal	DPP10	ENSG00000175497	0,89	1,89012931	0,78	0,93	0,32
Ependymal	HSPB8	ENSG00000152137	0,813	1,536422278	0,626	0,69	0,08
Ependymal	NSMF	ENSG00000165802	0,824	0,975028771	0,648	0,76	0,15
Ependymal	KIAA1671	ENSG00000197077	0,83	1,021384518	0,66	0,8	0,2
Ependymal	ZBTB7C	ENSG00000184828	0,816	1,067085521	0,632	0,75	0,14
Ependymal	SPATA18	ENSG00000163071	0,805	1,016887799	0,61	0,62	0,01
Ependymal	FMN2	ENSG00000155816	0,86	1,076302125	0,72	0,95	0,34
Ependymal	MARCH10	ENSG00000173838	0,809	1,0756067	0,618	0,64	0,04
Ependymal	CD99	ENSG00000002586	0,801	0,88092445	0,602	0,72	0,12
Ependymal	FAM81B	ENSG00000153347	0,801	0,959816061	0,602	0,61	0,01
Ependymal	WLS	ENSG00000116729	0,805	0,896400044	0,61	0,8	0,2
Ependymal	CFAP97D2	ENSG00000283361	0,801	0,974098542	0,602	0,61	0,01
Ependymal	KIF27	ENSG00000165115	0,865	1,182718719	0,73	0,85	0,25
Ependymal	FOXP2	ENSG00000128573	0,789	0,792212816	0,578	0,77	0,18
Ependymal	KCNN3	ENSG00000143603	0,807	0,737906201	0,614	0,81	0,22
Ependymal	CROCC2	ENSG00000226321	0,799	1,123138377	0,598	0,6	0
Ependymal	CEP112	ENSG00000154240	0,829	0,988159658	0,658	0,87	0,27
Ependymal	WWTR1	ENSG00000018408	0,829	1,147578042	0,658	0,83	0,23
Ependymal	TTC26	ENSG00000105948	0,817	1,011257285	0,634	0,7	0,11
Ependymal	SPAG16	ENSG00000144451	0,932	2,047599244	0,864	0,92	0,33
Ependymal	CNTN3	ENSG00000113805	0,813	1,127145444	0,626	0,77	0,18
Ependymal	NRG3	ENSG00000185737	0,804	0,855570836	0,608	0,98	0,39
Ependymal	AK7	ENSG00000140057	0,801	1,0060597	0,602	0,63	0,04
Ependymal	SDK1	ENSG00000146555	0,849	1,357832247	0,698	0,85	0,26
Ependymal	SLC6A16	ENSG00000063127	0,8	0,928569354	0,6	0,63	0,04
Ependymal	CCDC191	ENSG00000163617	0,827	0,918575121	0,654	0,77	0,19
Ependymal	GRAMD2B	ENSG00000155324	0,862	1,162546894	0,724	0,92	0,34
Ependymal	COL8A1	ENSG00000144810	0,793	1,165453491	0,586	0,62	0,04
Ependymal	CSMD3	ENSG00000164796	0,816	0,895175646	0,632	0,93	0,35
Ependymal	DNAAF4	ENSG00000256061	0,812	0,951449207	0,624	0,71	0,12
Ependymal	AC007906.2	ENSG00000277639	0,791	1,018325377	0,582	0,58	0
Ependymal	CACHD1	ENSG00000158966	0,801	0,880081523	0,602	0,81	0,23
Ependymal	PLCE1	ENSG00000138193	0,794	0,829658111	0,588	0,74	0,16
Ependymal	NME5	ENSG00000112981	0,816	0,993955842	0,632	0,72	0,14

Ependymal	C9orf135	ENSG00000204711	0,789	1,090889269	0,578	0,58	0,01
Ependymal	TTC23L	ENSG00000205838	0,81	1,110603215	0,62	0,68	0,1
Ependymal	SOX9	ENSG00000125398	0,786	0,93103583	0,572	0,67	0,1
Ependymal	ZNF273	ENSG00000198039	0,817	1,018226924	0,634	0,73	0,16
Ependymal	TTC21A	ENSG00000168026	0,8	0,898656453	0,6	0,66	0,09
Ependymal	KNDC1	ENSG00000171798	0,802	0,844034156	0,604	0,72	0,15
Ependymal	DYNC2H1	ENSG00000187240	0,945	1,781094208	0,89	0,96	0,39
Ependymal	LRP2BP	ENSG00000109771	0,821	0,957008021	0,642	0,77	0,2
Ependymal	B3GLCT	ENSG00000187676	0,793	0,920666304	0,586	0,68	0,11
Ependymal	C2orf73	ENSG00000177994	0,788	0,913288249	0,576	0,6	0,04
Ependymal	PARD3	ENSG00000148498	0,873	1,156822995	0,746	0,99	0,42
Ependymal	PPARGC1A	ENSG00000109819	0,8	1,201362692	0,6	0,7	0,13
Ependymal	CNTNAP3B	ENSG00000154529	0,799	0,990036978	0,598	0,75	0,19
Ependymal	GRIN2A	ENSG00000183454	0,795	1,171477865	0,59	0,72	0,15
Ependymal	ZMYND10	ENSG00000004838	0,783	0,948632553	0,566	0,58	0,02
Ependymal	CABCOCO1	ENSG00000183346	0,791	0,969942814	0,582	0,66	0,1
Ependymal	FAM183A	ENSG00000186973	0,78	0,833392645	0,56	0,56	0
Ependymal	TRPC6	ENSG00000137672	0,784	0,947354014	0,568	0,6	0,04
Ependymal	SLC7A11	ENSG00000151012	0,793	0,903816626	0,586	0,79	0,23
Ependymal	PTPRG	ENSG00000144724	0,782	0,622730232	0,564	0,89	0,33
Ependymal	CFAP74	ENSG00000142609	0,782	0,863554608	0,564	0,58	0,02
Ependymal	GRAMD2A	ENSG00000175318	0,782	1,104035599	0,564	0,58	0,02
Ependymal	CDS1	ENSG00000163624	0,796	0,922423001	0,592	0,66	0,1
Ependymal	BBOX1	ENSG00000129151	0,776	0,885035529	0,552	0,62	0,06
Ependymal	EXPH5	ENSG00000110723	0,779	0,983248097	0,558	0,61	0,05
Ependymal	ZFP36L1	ENSG00000185650	0,772	0,770329408	0,544	0,79	0,23
Ependymal	PITPNC1	ENSG00000154217	0,808	0,737383482	0,616	0,94	0,38
Ependymal	SORBS1	ENSG00000095637	0,886	1,367055105	0,772	0,97	0,41
Ependymal	IQUB	ENSG00000164675	0,786	0,868105158	0,572	0,62	0,06
Ependymal	RASSF9	ENSG00000198774	0,777	0,920599678	0,554	0,57	0,01
Ependymal	CCDC175	ENSG00000151838	0,783	0,920112556	0,566	0,61	0,05
Ependymal	PCAT1	ENSG00000253438	0,789	1,424283356	0,578	0,64	0,09
Ependymal	WDR93	ENSG00000140527	0,776	0,884813566	0,552	0,56	0,01
Ependymal	AKAP13	ENSG00000170776	0,837	0,970061832	0,674	0,9	0,36
Ependymal	WDR19	ENSG00000157796	0,814	0,966616118	0,628	0,76	0,22
Ependymal	ENPEP	ENSG00000138792	0,773	0,925245549	0,546	0,56	0,02
Ependymal	ADGRV1	ENSG00000164199	0,791	1,182527578	0,582	0,67	0,13
Ependymal	SSBP4	ENSG00000130511	0,789	0,850708523	0,578	0,68	0,13
Ependymal	AC004949.1	ENSG00000283117	0,774	1,567178066	0,548	0,56	0,02
Ependymal	DZANK1	ENSG00000089091	0,797	0,918195504	0,594	0,69	0,15
Ependymal	PLEKHA5	ENSG00000052126	0,913	1,511321287	0,826	0,97	0,43
Ependymal	TTC8	ENSG00000165533	0,793	0,886674011	0,586	0,69	0,16
Ependymal	DPY19L2	ENSG00000177990	0,787	0,867822284	0,574	0,71	0,17
Ependymal	ANTXR1	ENSG00000169604	0,785	0,850823882	0,57	0,74	0,21
Ependymal	ST8SIA1	ENSG00000111728	0,795	0,955738998	0,59	0,79	0,25
Ependymal	RSPH3	ENSG00000130363	0,786	0,853200815	0,572	0,66	0,12
Ependymal	C7orf57	ENSG00000164746	0,768	0,800724516	0,536	0,54	0
Ependymal	PRKG1	ENSG00000185532	0,767	0,831529738	0,534	0,79	0,26
Ependymal	NELFA	ENSG00000185049	0,825	0,990294643	0,65	0,79	0,26
Ependymal	JHY	ENSG00000109944	0,787	0,861190279	0,574	0,65	0,12
Ependymal	HPSE2	ENSG00000172987	0,759	0,901414064	0,518	0,69	0,16
Ependymal	TRAF3IP1	ENSG00000204104	0,798	0,905769788	0,596	0,72	0,19
Ependymal	RABL2A	ENSG00000144134	0,792	0,93307074	0,584	0,69	0,16
Ependymal	CFAP300	ENSG00000137691	0,767	0,805641109	0,534	0,55	0,03
Ependymal	CAPSL	ENSG00000152611	0,763	0,77972685	0,526	0,53	0

Ependymal	FAM92B	ENSG00000153789	0,763	0,790531355	0,526	0,53	0,01
Ependymal	CC2D2A	ENSG00000048342	0,789	0,889544797	0,578	0,69	0,17
Ependymal	KIF9	ENSG00000088727	0,769	0,792152991	0,538	0,57	0,04
Ependymal	PTPN3	ENSG00000070159	0,762	0,817530978	0,524	0,54	0,02
Ependymal	STRBP	ENSG00000165209	0,871	1,387544565	0,742	0,88	0,36
Ependymal	RAD9A	ENSG00000172613	0,794	0,867267426	0,588	0,76	0,24
Ependymal	EFCAB2	ENSG00000203666	0,893	1,388371832	0,786	0,92	0,4
Ependymal	RIPOR2	ENSG00000111913	0,777	1,030137809	0,554	0,65	0,13
Ependymal	ANKRD45	ENSG00000183831	0,764	0,772485612	0,528	0,55	0,02
Ependymal	GPM6A	ENSG00000150625	0,756	0,740836999	0,512	0,86	0,34
Ependymal	LRRC23	ENSG00000010626	0,779	0,81926431	0,558	0,66	0,14
Ependymal	FLACC1	ENSG00000155749	0,772	0,884365306	0,544	0,6	0,08
Ependymal	AC091078.1	ENSG00000257060	0,767	1,05578499	0,534	0,61	0,09
Ependymal	C11orf88	ENSG00000183644	0,76	0,818285186	0,52	0,52	0
Ependymal	FOS	ENSG00000170345	0,766	1,190732233	0,532	0,59	0,07
Ependymal	PAWR	ENSG00000177425	0,759	0,730566275	0,518	0,62	0,1
Ependymal	JUN	ENSG00000177606	0,777	1,08382925	0,554	0,67	0,15
Ependymal	COLEC12	ENSG00000158270	0,756	0,834003556	0,512	0,63	0,12
Ependymal	CTNNA2	ENSG00000066032	0,832	1,015751618	0,664	0,98	0,46
Ependymal	STON2	ENSG00000140022	0,773	0,77792102	0,546	0,71	0,19
Ependymal	TGFBR3	ENSG00000069702	0,762	0,75928977	0,524	0,65	0,13
Ependymal	ANTXR2	ENSG00000163297	0,761	0,848672746	0,522	0,58	0,06
Ependymal	PPP1R14C	ENSG00000198729	0,77	0,941609747	0,54	0,59	0,08
Ependymal	MED12L	ENSG00000144893	0,782	0,87224637	0,564	0,79	0,28
Ependymal	AC093689.1	ENSG00000250597	0,757	0,792568373	0,514	0,52	0
Ependymal	FAM160A1	ENSG00000164142	0,758	1,042601443	0,516	0,53	0,02
Ependymal	FAM184A	ENSG00000111879	0,798	1,148526244	0,596	0,72	0,21
Ependymal	STIM1	ENSG00000167323	0,79	1,001448619	0,58	0,74	0,23
Ependymal	CCDC180	ENSG00000197816	0,788	0,893826411	0,576	0,68	0,17
Ependymal	SYT17	ENSG00000103528	0,758	0,73387949	0,516	0,72	0,2
Ependymal	CD24	ENSG00000272398	0,758	0,958559912	0,516	0,53	0,02
Ependymal	IFT81	ENSG00000122970	0,777	0,798578803	0,554	0,66	0,15
Ependymal	RPGRIP1L	ENSG00000103494	0,776	0,85170169	0,552	0,64	0,13
Ependymal	NFATC2	ENSG00000101096	0,747	0,843589527	0,494	0,63	0,12
Ependymal	PLEKHA7	ENSG00000166689	0,887	1,348984215	0,774	0,92	0,41
Ependymal	TACC2	ENSG00000138162	0,776	0,83884369	0,552	0,67	0,16
Ependymal	ECHDC2	ENSG00000121310	0,772	0,692259219	0,544	0,77	0,26
Ependymal	KIAA1257	ENSG00000114656	0,763	0,806574483	0,526	0,56	0,05
Ependymal	EFHB	ENSG00000163576	0,76	0,817025099	0,52	0,55	0,05
Ependymal	SLC39A12	ENSG00000148482	0,75	0,828448673	0,5	0,57	0,07
Ependymal	ACSS3	ENSG00000111058	0,75	0,670374131	0,5	0,63	0,12
Ependymal	CEP83	ENSG00000173588	0,811	1,06477416	0,622	0,77	0,27
Ependymal	TTC25	ENSG00000204815	0,76	0,73789821	0,52	0,55	0,05
Ependymal	CFAP57	ENSG00000243710	0,754	0,738944581	0,508	0,52	0,01
Ependymal	HYDIN	ENSG00000157423	0,97	2,220043231	0,94	0,97	0,47
Ependymal	DPYSL3	ENSG00000113657	0,765	0,833579225	0,53	0,67	0,16
Ependymal	TMEM47	ENSG00000147027	0,764	0,90804501	0,528	0,62	0,11
Ependymal	TCTN1	ENSG00000204852	0,763	0,761423788	0,526	0,62	0,12
Ependymal	MOB1B	ENSG00000173542	0,787	0,915228596	0,574	0,73	0,23
Ependymal	KIAA1217	ENSG00000120549	0,742	0,649742539	0,484	0,75	0,25
Ependymal	NME9	ENSG00000181322	0,8	1,037394348	0,6	0,75	0,25
Ependymal	AL645937.2	ENSG00000227206	0,751	0,786399229	0,502	0,5	0
Ependymal	LAMA1	ENSG00000101680	0,747	0,757667007	0,494	0,58	0,08
Ependymal	STK32A	ENSG00000169302	0,742	0,670916645	0,484	0,67	0,17
Ependymal	WDR60	ENSG00000126870	0,892	1,296991855	0,784	0,92	0,43

Ependymal	KCNMA1	ENSG00000156113	0,797	0,739794525	0,594	0,95	0,45
Ependymal	ACACB	ENSG00000076555	0,767	0,635691947	0,534	0,8	0,3
Ependymal	FBXO15	ENSG00000141665	0,764	0,869452452	0,528	0,6	0,11
Ependymal	COL6A2	ENSG00000142173	0,746	0,718970707	0,492	0,53	0,03
Ependymal	CCDC88C	ENSG00000015133	0,756	0,793326839	0,512	0,57	0,07
Ependymal	CLHC1	ENSG00000162994	0,77	0,832090657	0,54	0,64	0,15
Ependymal	BCAR3	ENSG00000137936	0,749	0,646287881	0,498	0,71	0,21
Ependymal	SPEF1	ENSG00000101222	0,748	0,724782588	0,496	0,5	0,01
Ependymal	ABCC9	ENSG00000069431	0,744	0,714902451	0,488	0,55	0,06
Ependymal	CSPP1	ENSG00000104218	0,911	1,391476178	0,822	0,94	0,45
Ependymal	INTU	ENSG00000164066	0,826	0,909141851	0,652	0,86	0,37
Ependymal	MAPK15	ENSG00000181085	0,746	0,69580291	0,492	0,49	0
Ependymal	ARHGAP18	ENSG00000146376	0,751	0,755377525	0,502	0,64	0,15
Ependymal	MIPEP	ENSG00000027001	0,784	0,910026463	0,568	0,73	0,24
Ependymal	DNAJB1	ENSG00000132002	0,779	0,883765534	0,558	0,72	0,23
Ependymal	GRIA1	ENSG00000155511	0,735	0,696324603	0,47	0,63	0,14
Ependymal	ANKMY1	ENSG00000144504	0,781	0,845343589	0,562	0,72	0,23
Ependymal	AC234582.1	ENSG00000231064	0,744	0,729399413	0,488	0,52	0,03
Ependymal	RABL2B	ENSG00000079974	0,763	0,747301884	0,526	0,62	0,13
Ependymal	NTRK2	ENSG00000148053	0,87	1,118823473	0,74	0,98	0,49
Ependymal	ZNRF3	ENSG00000183579	0,765	0,744449718	0,53	0,75	0,26
Ependymal	LCA5	ENSG00000135338	0,76	0,793254618	0,52	0,62	0,13
Ependymal	ITGB4	ENSG00000132470	0,734	0,594367268	0,468	0,62	0,13
Ependymal	ODF2L	ENSG00000122417	0,825	1,035040963	0,65	0,83	0,34
Ependymal	NR2F1	ENSG00000175745	0,744	0,659685922	0,488	0,62	0,13
Ependymal	ZNF19	ENSG00000157429	0,745	0,81980082	0,49	0,51	0,03
Ependymal	MAK	ENSG00000111837	0,757	0,748496202	0,514	0,61	0,13
Ependymal	COL6A1	ENSG00000142156	0,748	0,716303751	0,496	0,6	0,12
Ependymal	CCN1	ENSG00000142871	0,743	1,313729223	0,486	0,5	0,02
Ependymal	CFAP58	ENSG00000120051	0,742	0,852299144	0,484	0,49	0,01
Ependymal	CRISPLD1	ENSG00000121005	0,745	0,89853676	0,49	0,55	0,07
Ependymal	TSGA10	ENSG00000135951	0,855	1,349461556	0,71	0,85	0,37
Ependymal	ZNF487	ENSG00000243660	0,753	0,717272295	0,506	0,57	0,09
Ependymal	SYNE2	ENSG00000054654	0,74	0,624420051	0,48	0,63	0,14
Ependymal	BOC	ENSG00000144857	0,733	0,59814224	0,466	0,6	0,12
Ependymal	SDK2	ENSG00000069188	0,753	0,829086491	0,506	0,59	0,11
Ependymal	NR2F1-AS1	ENSG00000237187	0,737	0,673642466	0,474	0,68	0,2
Ependymal	CERKL	ENSG00000188452	0,741	0,75930821	0,482	0,49	0,01
Ependymal	OSBPL6	ENSG00000079156	0,74	0,690168439	0,48	0,72	0,25
Ependymal	TTC12	ENSG00000149292	0,749	0,72172846	0,498	0,6	0,12
Ependymal	MAOB	ENSG00000069535	0,747	0,790593662	0,494	0,62	0,14
Ependymal	LMO2	ENSG00000135363	0,739	0,826018164	0,478	0,56	0,08
Ependymal	C9orf24	ENSG00000164972	0,748	0,79627305	0,496	0,54	0,07
Ependymal	DAAM1	ENSG00000100592	0,776	0,852430519	0,552	0,8	0,32
Ependymal	EML6	ENSG00000214595	0,759	0,822269804	0,518	0,72	0,24
Ependymal	PRR29	ENSG00000224383	0,737	0,794028742	0,474	0,48	0
Ependymal	IQCK	ENSG00000174628	0,802	0,935666464	0,604	0,81	0,34
Ependymal	FOXJ1	ENSG00000129654	0,736	0,760737777	0,472	0,48	0,01
Ependymal	AGBL2	ENSG00000165923	0,736	0,725386982	0,472	0,48	0,01
Ependymal	AGT	ENSG00000135744	0,734	0,729645789	0,468	0,59	0,12
Ependymal	KATNAL2	ENSG00000167216	0,787	0,950760961	0,574	0,77	0,3
Ependymal	SHROOM3	ENSG00000138771	0,738	0,772802507	0,476	0,57	0,1
Ependymal	CCDC57	ENSG00000176155	0,76	0,73614487	0,52	0,68	0,21
Ependymal	FGFR1OP	ENSG00000213066	0,762	0,771196175	0,524	0,67	0,2
Ependymal	COL28A1	ENSG00000215018	0,754	1,167509166	0,508	0,58	0,11

Ependymal	ARMC9	ENSG00000135931	0,773	0,756625475	0,546	0,77	0,3
Ependymal	AK9	ENSG00000155085	0,776	0,835570769	0,552	0,73	0,27
Ependymal	HSPA1B	ENSG00000204388	0,752	0,907624303	0,504	0,67	0,21
Ependymal	PLOD2	ENSG00000152952	0,739	0,659447955	0,478	0,65	0,19
Ependymal	DZIP3	ENSG00000198919	0,795	0,804661616	0,59	0,83	0,37
Ependymal	ANKRD26	ENSG00000107890	0,844	1,035335354	0,688	0,88	0,42
Ependymal	ENKUR	ENSG00000151023	0,734	0,69006597	0,468	0,5	0,04
Ependymal	FBXL2	ENSG00000153558	0,754	0,729641507	0,508	0,72	0,26
Ependymal	AC063979.2	ENSG00000251613	0,733	0,843988121	0,466	0,48	0,02
Ependymal	ID4	ENSG00000172201	0,732	0,711633586	0,464	0,53	0,07
Ependymal	DUSP18	ENSG00000167065	0,751	0,757248271	0,502	0,62	0,16
Ependymal	GNG12	ENSG00000172380	0,731	0,661914644	0,462	0,58	0,12
Ependymal	VIM	ENSG00000026025	0,722	0,692823604	0,444	0,56	0,1
Ependymal	C20orf96	ENSG00000196476	0,735	0,656333732	0,47	0,52	0,06
Ependymal	ANXA1	ENSG00000135046	0,729	0,9829546	0,458	0,48	0,02
Ependymal	FSTL1	ENSG00000163430	0,73	0,642464272	0,46	0,54	0,08
Ependymal	PARD3B	ENSG00000116117	0,922	1,430523538	0,844	0,98	0,52
Ependymal	HSPB1	ENSG00000106211	0,746	0,920917539	0,492	0,66	0,21
Ependymal	DAPK1	ENSG00000196730	0,732	0,648625415	0,464	0,65	0,19
Ependymal	FAM81A	ENSG00000157470	0,744	0,775903465	0,488	0,58	0,12
Ependymal	CROCC	ENSG00000058453	0,781	0,928240375	0,562	0,74	0,29
Ependymal	FAM182B	ENSG00000175170	0,78	0,821406445	0,56	0,8	0,34
Ependymal	DNALI1	ENSG00000163879	0,731	0,629236204	0,462	0,49	0,04
Ependymal	MEIS2	ENSG00000134138	0,794	0,929040642	0,588	0,86	0,41
Ependymal	EFCAB11	ENSG00000140025	0,744	0,826563261	0,488	0,66	0,21
Ependymal	PROS1	ENSG00000184500	0,739	0,881380994	0,478	0,56	0,11
Ependymal	ANKDD1B	ENSG00000189045	0,734	0,839011336	0,468	0,51	0,05
Ependymal	RIBC2	ENSG00000128408	0,727	0,646577224	0,454	0,46	0,01
Ependymal	WNK2	ENSG00000165238	0,725	0,614458114	0,45	0,63	0,18
Ependymal	PRKD1	ENSG00000184304	0,765	0,757728998	0,53	0,85	0,4
Ependymal	LEKR1	ENSG00000197980	0,732	0,84901465	0,464	0,51	0,06
Ependymal	OCA2	ENSG00000104044	0,73	0,882853303	0,46	0,49	0,04
Ependymal	DRC7	ENSG00000159625	0,726	0,67711745	0,452	0,46	0
Ependymal	AEBP1	ENSG00000106624	0,721	0,676058333	0,442	0,51	0,06
Ependymal	KCTD1	ENSG00000134504	0,748	0,766355865	0,496	0,7	0,25
Ependymal	PRICKLE2	ENSG00000163637	0,763	0,94634237	0,526	0,76	0,31
Ependymal	PHF21B	ENSG00000056487	0,725	0,628518054	0,45	0,61	0,16
Ependymal	GALNS	ENSG00000141012	0,734	0,672799735	0,468	0,55	0,1
Ependymal	TUBA4B	ENSG00000243910	0,725	0,725388112	0,45	0,45	0
Ependymal	ECT2L	ENSG00000203734	0,728	0,934484132	0,456	0,48	0,04
Ependymal	BAIAP2	ENSG00000175866	0,737	0,828117624	0,474	0,58	0,14
Ependymal	CATIP	ENSG00000158428	0,723	0,643169254	0,446	0,45	0,01
Ependymal	COL5A1	ENSG00000130635	0,725	0,832430102	0,45	0,48	0,03
Ependymal	ENO4	ENSG00000188316	0,735	0,792163248	0,47	0,56	0,12
Ependymal	BICC1	ENSG00000122870	0,731	0,90190594	0,462	0,55	0,11
Ependymal	PTRH1	ENSG00000187024	0,726	0,630745533	0,452	0,49	0,05
Ependymal	TPPP3	ENSG00000159713	0,739	0,900629982	0,478	0,6	0,16
Ependymal	TUSC3	ENSG00000104723	0,726	0,669091267	0,452	0,59	0,15
Ependymal	DDAH1	ENSG00000153904	0,733	0,719214021	0,466	0,64	0,2
Ependymal	NME7	ENSG00000143156	0,776	0,859995721	0,552	0,78	0,34
Ependymal	STOX1	ENSG00000165730	0,723	0,607669869	0,446	0,53	0,09
Ependymal	PPP1R15A	ENSG00000087074	0,724	0,909492663	0,448	0,49	0,05
Ependymal	AC046134.2	ENSG00000248932	0,762	0,710486058	0,524	0,81	0,37
Ependymal	IFT122	ENSG00000163913	0,738	0,668971619	0,476	0,62	0,18
Ependymal	LINC00880	ENSG00000243629	0,72	0,744290653	0,44	0,46	0,02

Ependymal	DNAH1	ENSG00000114841	0,729	0,624351458	0,458	0,55	0,11
Ependymal	IFT88	ENSG00000032742	0,804	0,881283938	0,608	0,85	0,41
Ependymal	ERICH3	ENSG00000178965	0,73	0,732990162	0,46	0,52	0,09
Ependymal	TCF7L1	ENSG00000152284	0,713	0,633827398	0,426	0,55	0,11
Ependymal	TCF7L2	ENSG00000148737	0,762	0,69571044	0,524	0,86	0,43
Ependymal	FAM167A-AS1	ENSG00000184608	0,719	0,715194244	0,438	0,45	0,02
Ependymal	OSMR-AS1	ENSG00000249740	0,714	0,601600911	0,428	0,52	0,08
Ependymal	TNIK	ENSG00000154310	0,783	0,74783152	0,566	0,94	0,5
Ependymal	HAGHL	ENSG00000103253	0,734	0,703953045	0,468	0,59	0,15
Ependymal	AC110023.1	ENSG00000258631	0,724	1,077486506	0,448	0,52	0,09
Ependymal	REEP1	ENSG00000068615	0,726	0,706403064	0,452	0,58	0,14
Ependymal	HSPH1	ENSG00000120694	0,787	1,148054693	0,574	0,78	0,35
Ependymal	SUN1	ENSG00000164828	0,782	0,757168752	0,564	0,79	0,35
Ependymal	UBXN11	ENSG00000158062	0,721	0,596964785	0,442	0,47	0,04
Ependymal	LINC00271	ENSG00000231028	0,731	0,812189811	0,462	0,58	0,14
Ependymal	MYO5C	ENSG00000128833	0,72	0,776687289	0,44	0,5	0,07
Ependymal	DNAJB13	ENSG00000187726	0,717	0,606422962	0,434	0,44	0,01
Ependymal	DCLK2	ENSG00000170390	0,754	0,744588897	0,508	0,82	0,38
Ependymal	PHTF1	ENSG00000116793	0,731	0,66096666	0,462	0,6	0,17
Ependymal	NADK2	ENSG00000152620	0,734	0,674674001	0,468	0,63	0,2
Ependymal	CLYBL	ENSG00000125246	0,73	0,769321114	0,46	0,6	0,16
Ependymal	TEAD1	ENSG00000187079	0,826	0,970838995	0,652	0,91	0,48
Ependymal	C1orf158	ENSG00000157330	0,715	0,710226984	0,43	0,43	0
Ependymal	ST5	ENSG00000166444	0,728	0,636316799	0,456	0,67	0,24
Ependymal	BMP7	ENSG00000101144	0,721	0,596906301	0,442	0,61	0,18
Ependymal	LMLN	ENSG00000185621	0,732	0,671372974	0,464	0,57	0,14
Ependymal	HIPK1	ENSG00000163349	0,74	0,715885919	0,48	0,65	0,22
Ependymal	FRMD6	ENSG00000139926	0,72	0,698921576	0,44	0,55	0,12
Ependymal	MORN1	ENSG00000116151	0,718	0,616216403	0,436	0,48	0,06
Ependymal	CLU	ENSG00000120885	0,903	1,542070405	0,806	0,97	0,55
Ependymal	TOB1	ENSG00000141232	0,737	0,970789292	0,474	0,59	0,17
Ependymal	CRLF1	ENSG00000006016	0,72	0,724152892	0,44	0,5	0,07
Ependymal	COL4A6	ENSG00000197565	0,712	0,711867116	0,424	0,44	0,01
Ependymal	SYNE1	ENSG00000131018	0,94	1,608089834	0,88	0,99	0,57
Ependymal	IFT140	ENSG00000187535	0,722	0,592561511	0,444	0,55	0,13
Ependymal	LRRC74B	ENSG00000187905	0,712	0,669499902	0,424	0,43	0,01
Ependymal	NSUN7	ENSG00000179299	0,713	0,660243492	0,426	0,45	0,03
Ependymal	LRRC46	ENSG00000141294	0,711	0,60428762	0,422	0,43	0
Ependymal	CCDC113	ENSG00000103021	0,714	0,634987121	0,428	0,46	0,04
Ependymal	CELSR1	ENSG00000075275	0,711	0,630760743	0,422	0,44	0,02
Ependymal	AC016705.2	ENSG00000259495	0,724	0,6932755	0,448	0,58	0,16
Ependymal	IFT43	ENSG00000119650	0,746	0,664232762	0,492	0,71	0,29
Ependymal	GALNT8	ENSG00000130035	0,722	0,699894268	0,444	0,54	0,12
Ependymal	FGFR1	ENSG00000077782	0,728	0,624436044	0,456	0,68	0,27
Ependymal	ASPH	ENSG00000198363	0,77	0,738238746	0,54	0,85	0,43
Ependymal	DSE	ENSG00000111817	0,751	0,763458773	0,502	0,75	0,33
Ependymal	FSIP1	ENSG00000150667	0,717	0,775854236	0,434	0,49	0,07
Ependymal	DENND6B	ENSG00000205593	0,719	0,656430826	0,438	0,51	0,1
Ependymal	FAT1	ENSG00000083857	0,72	0,732760378	0,44	0,57	0,16
Ependymal	LTBP1	ENSG00000049323	0,714	0,685973545	0,428	0,61	0,2
Ependymal	PALLD	ENSG00000129116	0,718	0,679147453	0,436	0,64	0,22
Ependymal	TGFB2	ENSG00000092969	0,705	0,643131914	0,41	0,5	0,09
Ependymal	MUSK	ENSG00000030304	0,707	0,733174412	0,414	0,44	0,03
Ependymal	ROR1	ENSG00000185483	0,705	0,772811759	0,41	0,52	0,11
Ependymal	CDC14A	ENSG00000079335	0,701	0,608458493	0,402	0,57	0,16

Ependymal	STXBP4	ENSG00000166263	0,728	0,683396658	0,456	0,66	0,25
Ependymal	CST3	ENSG00000101439	0,71	0,693113814	0,42	0,76	0,35
Ependymal	PIFO	ENSG00000173947	0,704	0,58585111	0,408	0,41	0,01
Ependymal	METRN	ENSG00000103260	0,744	0,74563816	0,488	0,75	0,34
Ependymal	LINC00907	ENSG00000267586	0,714	1,378390019	0,428	0,5	0,1
Ependymal	RALGPS2	ENSG00000116191	0,706	0,602823429	0,412	0,6	0,2
Ependymal	WDR86	ENSG00000187260	0,703	0,657435804	0,406	0,41	0,01
Ependymal	ANKUB1	ENSG00000206199	0,737	1,09747125	0,474	0,61	0,21
Ependymal	MIR4300HG	ENSG00000245832	0,719	1,224257668	0,438	0,57	0,17
Ependymal	CRNDE	ENSG00000245694	0,711	0,585693865	0,422	0,59	0,19
Ependymal	DGLUCY	ENSG00000133943	0,766	0,77518583	0,532	0,81	0,41
Ependymal	HIPK3	ENSG00000110422	0,78	0,782249061	0,56	0,83	0,43
Ependymal	TUBB4B	ENSG00000188229	0,723	0,890715418	0,446	0,58	0,18
Ependymal	NFIB	ENSG00000147862	0,755	0,659093294	0,51	0,91	0,51
Ependymal	LCLAT1	ENSG00000172954	0,719	0,64097075	0,438	0,62	0,22
Ependymal	BCO2	ENSG00000197580	0,719	0,599727082	0,438	0,6	0,21
Ependymal	SYNGAP1	ENSG00000197283	0,711	0,591158358	0,422	0,52	0,13
Ependymal	HSPD1	ENSG00000144381	0,739	0,814622848	0,478	0,7	0,3
Ependymal	UBE3D	ENSG00000118420	0,751	0,984241137	0,502	0,71	0,32
Ependymal	MAN1C1	ENSG00000117643	0,714	0,659369748	0,428	0,65	0,26
Ependymal	LINC00535	ENSG00000246662	0,701	0,598576535	0,402	0,49	0,1
Ependymal	KIAA0556	ENSG00000047578	0,736	0,674016418	0,472	0,71	0,32
Ependymal	ZFHX2	ENSG00000136367	0,714	0,591010544	0,428	0,59	0,21
Ependymal	C11orf49	ENSG00000149179	0,774	0,786111591	0,548	0,84	0,45
Ependymal	CAPS2	ENSG00000180881	0,743	0,644619797	0,486	0,83	0,44
Ependymal	SUGCT	ENSG00000175600	0,707	0,720597745	0,414	0,57	0,18
Ependymal	CNTRL	ENSG00000119397	0,732	0,648893405	0,464	0,74	0,35
Ependymal	IFT172	ENSG00000138002	0,744	0,675690507	0,488	0,73	0,35
Ependymal	RFX3-AS1	ENSG00000232104	0,722	0,650973064	0,444	0,76	0,38
Ependymal	CDKL3	ENSG00000006837	0,715	0,589283016	0,43	0,66	0,28
Ependymal	CCDC148	ENSG00000153237	0,706	0,678373604	0,412	0,57	0,18
Ependymal	DOCK7	ENSG00000116641	0,72	0,592611227	0,44	0,77	0,39
Ependymal	RHOXF1-AS1	ENSG00000258545	0,701	0,592826815	0,402	0,51	0,13
Ependymal	CCDC138	ENSG00000163006	0,707	0,656786417	0,414	0,61	0,24
Ependymal	TTLL5	ENSG00000119685	0,774	0,722091607	0,548	0,88	0,52
Ependymal	PHACTR1	ENSG00000112137	0,77	0,670021093	0,54	0,92	0,56
Ependymal	CMSS1	ENSG00000184220	0,752	0,898010977	0,504	0,8	0,46
Ependymal	ARNT2	ENSG00000172379	0,748	0,678066861	0,496	0,86	0,52
Ependymal	SRGAP3	ENSG00000196220	0,762	0,666706695	0,524	0,92	0,58
Ependymal	RFX3	ENSG00000080298	0,958	1,550950967	0,916	0,99	0,67
Ependymal	PTGES3	ENSG00000110958	0,759	0,871890276	0,518	0,81	0,49
Ependymal	ITGB8	ENSG00000105855	0,731	0,604959599	0,462	0,87	0,56
Ependymal	DPP6	ENSG00000130226	0,812	0,827587537	0,624	0,98	0,68
Ependymal	MAML2	ENSG00000184384	0,764	0,67282152	0,528	0,96	0,65
Ependymal	MAPK10	ENSG00000109339	0,922	1,231518389	0,844	0,99	0,69
Ependymal	TRPS1	ENSG00000104447	0,823	0,876366553	0,646	0,97	0,67
Ependymal	UBC	ENSG00000150991	0,764	0,856703618	0,528	0,86	0,56
Ependymal	CKB	ENSG00000166165	0,725	0,725297546	0,45	0,86	0,56
Ependymal	TLN2	ENSG00000171914	0,727	0,822715389	0,454	0,82	0,53
Ependymal	LIFR	ENSG00000113594	0,726	0,589247412	0,452	0,9	0,63
Ependymal	LDLRAD4	ENSG00000168675	0,796	0,700832933	0,592	0,96	0,69
Ependymal	EVI5	ENSG00000067208	0,734	0,602330892	0,468	0,86	0,6
Ependymal	MSI2	ENSG00000153944	0,936	1,420196003	0,872	0,99	0,73
Ependymal	PDE4DIP	ENSG00000178104	0,783	0,74275249	0,566	0,96	0,7
Ependymal	ARHGAP5	ENSG00000100852	0,736	0,593741266	0,472	0,89	0,64

Ependymal	MPDZ	ENSG00000107186	0,747	0,641731844	0,494	0,89	0,65
Ependymal	KAZN	ENSG00000189337	0,735	0,830717534	0,47	0,91	0,67
Ependymal	BBS9	ENSG00000122507	0,742	0,628588476	0,484	0,9	0,67
Ependymal	PPP1R12B	ENSG00000077157	0,876	1,076343031	0,752	0,97	0,74
Ependymal	DTNA	ENSG00000134769	0,837	0,799701127	0,674	0,99	0,76
Ependymal	AKAP9	ENSG00000127914	0,759	0,619817434	0,518	0,93	0,71
Ependymal	TMEM161B-AS1	ENSG00000247828	0,711	0,587948878	0,422	0,89	0,67
Ependymal	SSBP2	ENSG00000145687	0,776	0,664457187	0,552	0,96	0,75
Ependymal	NFIA	ENSG00000162599	0,803	0,682544109	0,606	0,98	0,82
Ependymal	MIR99AHG	ENSG00000215386	0,871	1,007117353	0,742	0,98	0,82
Ependymal	HSP90AA1	ENSG00000080824	0,839	1,413441761	0,678	0,96	0,83
Ependymal	WWOX	ENSG00000186153	0,84	0,855489244	0,68	0,99	0,88
CADPS2+ neurons	UNC13C	ENSG00000137766	0,99	2,777175102	0,98	0,99	0,1
CADPS2+ neurons	RBFOX3	ENSG00000167281	0,977	2,47934312	0,954	0,97	0,1
CADPS2+ neurons	ZNF385D	ENSG00000151789	0,985	3,208041293	0,97	0,98	0,15
CADPS2+ neurons	SYT1	ENSG00000067715	0,946	1,951045928	0,892	0,99	0,18
CADPS2+ neurons	CDH18	ENSG00000145526	0,975	2,523615503	0,95	0,99	0,19
CADPS2+ neurons	RELN	ENSG00000189056	0,947	2,31028535	0,894	0,92	0,11
CADPS2+ neurons	CAMK4	ENSG00000152495	0,962	2,487741773	0,924	0,94	0,15
CADPS2+ neurons	GALNT17	ENSG00000185274	0,955	2,076377322	0,91	0,95	0,16
CADPS2+ neurons	ZNF385B	ENSG00000144331	0,931	2,147834852	0,862	0,91	0,12
CADPS2+ neurons	GABRB2	ENSG00000145864	0,936	2,146970592	0,872	0,91	0,12
CADPS2+ neurons	SLIT3	ENSG00000184347	0,918	2,0997395	0,836	0,87	0,09
CADPS2+ neurons	KCNJ3	ENSG00000162989	0,954	2,181757234	0,908	0,94	0,16
CADPS2+ neurons	EPHA6	ENSG00000080224	0,916	1,893206344	0,832	0,91	0,14
CADPS2+ neurons	CA10	ENSG00000154975	0,927	1,863482614	0,854	0,94	0,17
CADPS2+ neurons	CADPS2	ENSG00000081803	0,982	2,605175186	0,964	0,98	0,21
CADPS2+ neurons	MCTP1	ENSG00000175471	0,947	2,105098657	0,894	0,94	0,17
CADPS2+ neurons	SPHKAP	ENSG00000153820	0,893	1,724963498	0,786	0,83	0,07
CADPS2+ neurons	ETV1	ENSG00000006468	0,913	1,984433417	0,826	0,86	0,1
CADPS2+ neurons	SRRM4	ENSG00000139767	0,915	1,889212739	0,83	0,86	0,11
CADPS2+ neurons	CNTN4	ENSG00000144619	0,904	1,542723997	0,808	0,93	0,18
CADPS2+ neurons	STXBP5L	ENSG00000145087	0,979	2,258784124	0,958	0,99	0,24
CADPS2+ neurons	SH3GL2	ENSG00000107295	0,932	2,018139739	0,864	0,92	0,17
CADPS2+ neurons	FSTL5	ENSG00000168843	0,984	2,648378905	0,968	0,98	0,24
CADPS2+ neurons	SNAP25	ENSG00000132639	0,97	2,093971667	0,94	0,98	0,24
CADPS2+ neurons	GRM4	ENSG00000124493	0,893	1,832618989	0,786	0,8	0,06
CADPS2+ neurons	RIMS2	ENSG00000176406	0,896	1,549625106	0,792	0,96	0,22
CADPS2+ neurons	ATP8A2	ENSG00000132932	0,923	1,766792193	0,846	0,93	0,2
CADPS2+ neurons	GRM1	ENSG00000152822	0,893	1,848514542	0,786	0,83	0,11
CADPS2+ neurons	RALYL	ENSG00000184672	0,953	1,993854351	0,906	0,99	0,27
CADPS2+ neurons	TRMT9B	ENSG00000250305	0,91	1,84491925	0,82	0,87	0,15
CADPS2+ neurons	SCN2A	ENSG00000136531	0,929	1,820666991	0,858	0,92	0,21
CADPS2+ neurons	CACNA1A	ENSG00000141837	0,97	2,003253922	0,94	0,99	0,29
CADPS2+ neurons	TRPM3	ENSG00000083067	0,861	1,041799208	0,722	0,97	0,27
CADPS2+ neurons	FGF14	ENSG00000102466	0,975	2,31710241	0,95	0,99	0,29
CADPS2+ neurons	SV2B	ENSG00000185518	0,87	1,829235518	0,74	0,77	0,07
CADPS2+ neurons	OPCML	ENSG00000183715	0,854	1,217549447	0,708	0,93	0,23
CADPS2+ neurons	AC007614.1	ENSG00000279249	0,859	1,770834481	0,718	0,73	0,04
CADPS2+ neurons	KCND2	ENSG00000184408	0,952	2,086957059	0,904	0,98	0,3
CADPS2+ neurons	RIMS1	ENSG00000079841	0,97	2,216184966	0,94	0,98	0,29
CADPS2+ neurons	GRIK2	ENSG00000164418	0,96	2,022130531	0,92	0,99	0,31
CADPS2+ neurons	PLCB4	ENSG00000101333	0,906	1,567689011	0,812	0,93	0,25
CADPS2+ neurons	RIT2	ENSG00000152214	0,875	1,977454083	0,75	0,8	0,12
CADPS2+ neurons	SRRM3	ENSG00000177679	0,865	1,39402854	0,73	0,83	0,15

CADPS2+ neurons	TRHDE	ENSG00000072657	0,858	1,634281126	0,716	0,77	0,09
CADPS2+ neurons	KCNIP4	ENSG00000185774	0,915	1,84229616	0,83	0,97	0,29
CADPS2+ neurons	ZFPM2	ENSG00000169946	0,942	2,020875624	0,884	0,97	0,3
CADPS2+ neurons	RYR2	ENSG00000198626	0,926	1,600233899	0,852	0,97	0,3
CADPS2+ neurons	UBASH3B	ENSG00000154127	0,882	1,777778513	0,764	0,82	0,15
CADPS2+ neurons	MYT1L	ENSG00000186487	0,837	1,200908307	0,674	0,84	0,18
CADPS2+ neurons	SLC35F4	ENSG00000151812	0,849	1,734475227	0,698	0,75	0,09
CADPS2+ neurons	CNKSR2	ENSG00000149970	0,896	1,76927157	0,792	0,86	0,2
CADPS2+ neurons	MTCL1	ENSG00000168502	0,911	1,830723157	0,822	0,87	0,21
CADPS2+ neurons	ARPP21	ENSG00000172995	0,881	1,493840122	0,762	0,9	0,25
CADPS2+ neurons	NMNAT2	ENSG00000157064	0,872	1,509145594	0,744	0,83	0,17
CADPS2+ neurons	TMEM178A	ENSG00000152154	0,871	1,490600331	0,742	0,81	0,17
CADPS2+ neurons	SYN2	ENSG00000157152	0,841	1,315237403	0,682	0,79	0,16
CADPS2+ neurons	ABLIM1	ENSG00000099204	0,913	1,578935198	0,826	0,94	0,31
CADPS2+ neurons	SYNPR	ENSG00000163630	0,826	1,596406008	0,652	0,7	0,07
CADPS2+ neurons	DLGAP1	ENSG00000170579	0,879	1,356443192	0,758	0,93	0,29
CADPS2+ neurons	TENM1	ENSG00000009694	0,853	1,69100763	0,706	0,78	0,15
CADPS2+ neurons	CNTN1	ENSG00000018236	0,876	1,327020006	0,752	0,97	0,35
CADPS2+ neurons	TLL1	ENSG00000038295	0,818	1,62893443	0,636	0,66	0,04
CADPS2+ neurons	CCSER1	ENSG00000184305	0,878	1,565657023	0,756	0,92	0,3
CADPS2+ neurons	EPHB1	ENSG00000154928	0,858	1,480898711	0,716	0,81	0,19
CADPS2+ neurons	PTPRR	ENSG00000153233	0,831	1,513183879	0,662	0,72	0,1
CADPS2+ neurons	MTUS2	ENSG00000132938	0,822	1,30860874	0,644	0,74	0,13
CADPS2+ neurons	MDGA1	ENSG00000112139	0,819	1,245227912	0,638	0,67	0,06
CADPS2+ neurons	ATP2B2	ENSG00000157087	0,854	1,369579412	0,708	0,83	0,23
CADPS2+ neurons	PATJ	ENSG00000132849	0,92	1,688138704	0,84	0,92	0,32
CADPS2+ neurons	PCLO	ENSG00000186472	0,944	1,761554965	0,888	0,97	0,37
CADPS2+ neurons	PDE3B	ENSG00000152270	0,841	1,473390184	0,682	0,8	0,21
CADPS2+ neurons	IL16	ENSG00000172349	0,803	1,379980625	0,606	0,63	0,05
CADPS2+ neurons	SEL1L3	ENSG00000091490	0,804	1,415429278	0,608	0,64	0,06
CADPS2+ neurons	ANK1	ENSG00000029534	0,814	1,385323876	0,628	0,69	0,11
CADPS2+ neurons	KSR2	ENSG00000171435	0,816	1,213782528	0,632	0,77	0,19
CADPS2+ neurons	FGF12	ENSG00000114279	0,788	0,900804714	0,576	0,82	0,24
CADPS2+ neurons	NDRG4	ENSG00000103034	0,83	1,347652309	0,66	0,73	0,15
CADPS2+ neurons	CLVS2	ENSG00000146352	0,812	1,542385605	0,624	0,67	0,1
CADPS2+ neurons	EPB41	ENSG00000159023	0,839	1,370986757	0,678	0,78	0,21
CADPS2+ neurons	UNC80	ENSG00000144406	0,811	1,109186903	0,622	0,8	0,23
CADPS2+ neurons	PLCXD3	ENSG00000182836	0,809	1,451589916	0,618	0,68	0,12
CADPS2+ neurons	PDE1A	ENSG00000115252	0,914	1,621042322	0,828	0,95	0,38
CADPS2+ neurons	MYRIP	ENSG00000170011	0,842	1,131650888	0,684	0,86	0,3
CADPS2+ neurons	FAT2	ENSG00000086570	0,782	1,368735206	0,564	0,57	0,01
CADPS2+ neurons	NRXN1	ENSG00000179915	0,932	1,542046815	0,864	1	0,44
CADPS2+ neurons	LINC01798	ENSG00000232046	0,781	1,447516188	0,562	0,57	0,01
CADPS2+ neurons	RUNX1T1	ENSG00000079102	0,847	1,387376059	0,694	0,85	0,3
CADPS2+ neurons	PAK5	ENSG00000101349	0,812	1,452446843	0,624	0,69	0,14
CADPS2+ neurons	NTRK3	ENSG00000140538	0,803	0,95600179	0,606	0,86	0,31
CADPS2+ neurons	SPTBN4	ENSG00000160460	0,82	1,14172363	0,64	0,8	0,26
CADPS2+ neurons	NRG1	ENSG00000157168	0,78	1,247321151	0,56	0,7	0,16
CADPS2+ neurons	MICAL2	ENSG00000133816	0,815	1,413465944	0,63	0,69	0,15
CADPS2+ neurons	OLFM3	ENSG00000118733	0,79	1,255049656	0,58	0,68	0,15
CADPS2+ neurons	TIAM1	ENSG00000156299	0,993	2,249162453	0,986	1	0,47
CADPS2+ neurons	RGS7	ENSG00000182901	0,907	1,521579769	0,814	0,96	0,43
CADPS2+ neurons	CELF4	ENSG00000101489	0,775	1,132802599	0,55	0,68	0,15
CADPS2+ neurons	RBFOX1	ENSG00000078328	0,939	1,659087428	0,878	1	0,47
CADPS2+ neurons	LINC00599	ENSG00000253230	0,787	1,20496658	0,574	0,63	0,1

CADPS2+ neurons	GRIN2A	ENSG00000183454	0,784	1,260847342	0,568	0,68	0,16
CADPS2+ neurons	CALN1	ENSG00000183166	0,923	1,613049895	0,846	0,97	0,45
CADPS2+ neurons	DGKG	ENSG00000058866	0,777	0,793123841	0,554	0,8	0,28
CADPS2+ neurons	AFF3	ENSG00000144218	0,84	1,133324606	0,68	0,9	0,38
CADPS2+ neurons	CDH22	ENSG00000149654	0,772	1,250915601	0,544	0,58	0,07
CADPS2+ neurons	ZNF521	ENSG00000198795	0,91	1,555541046	0,82	0,93	0,41
CADPS2+ neurons	VWC2	ENSG00000188730	0,775	1,345885903	0,55	0,63	0,11
CADPS2+ neurons	CADPS	ENSG00000163618	0,755	0,716869349	0,51	0,76	0,25
CADPS2+ neurons	XKR6	ENSG00000171044	0,837	1,243804748	0,674	0,88	0,38
CADPS2+ neurons	STMN2	ENSG00000104435	0,769	1,118692197	0,538	0,63	0,12
CADPS2+ neurons	NEBL	ENSG00000078114	0,779	0,818619524	0,558	0,88	0,37
CADPS2+ neurons	CAMK2B	ENSG00000058404	0,78	1,088973388	0,56	0,68	0,18
CADPS2+ neurons	TMEM266	ENSG00000169758	0,77	1,392710289	0,54	0,58	0,08
CADPS2+ neurons	PKIB	ENSG00000135549	0,764	1,239929415	0,528	0,57	0,07
CADPS2+ neurons	ZNF804A	ENSG00000170396	0,772	1,482431553	0,544	0,65	0,15
CADPS2+ neurons	EBF1	ENSG00000164330	0,757	1,043877149	0,514	0,65	0,15
CADPS2+ neurons	MAP2	ENSG00000078018	0,799	0,876222562	0,598	0,9	0,41
CADPS2+ neurons	PPFIA4	ENSG00000143847	0,769	1,217616583	0,538	0,59	0,1
CADPS2+ neurons	ZIC1	ENSG00000152977	0,766	1,081667899	0,532	0,64	0,15
CADPS2+ neurons	ZBTB18	ENSG00000179456	0,772	1,175107218	0,544	0,62	0,13
CADPS2+ neurons	NHSL2	ENSG00000204131	0,763	1,252097596	0,526	0,58	0,11
CADPS2+ neurons	FRY	ENSG00000073910	0,819	1,16009727	0,638	0,83	0,36
CADPS2+ neurons	CACNA1B	ENSG00000148408	0,74	0,829502995	0,48	0,66	0,18
CADPS2+ neurons	AKAP12	ENSG00000131016	0,766	1,04457593	0,532	0,66	0,19
CADPS2+ neurons	SNAP91	ENSG00000065609	0,799	1,045525401	0,598	0,81	0,34
CADPS2+ neurons	AC092957.1	ENSG00000243620	0,742	1,726616911	0,484	0,53	0,06
CADPS2+ neurons	MSRA	ENSG00000175806	0,967	1,924720338	0,934	0,98	0,51
CADPS2+ neurons	LHFPL6	ENSG00000183722	0,774	0,807292673	0,548	0,86	0,39
CADPS2+ neurons	GABBR2	ENSG00000136928	0,752	1,095051228	0,504	0,61	0,14
CADPS2+ neurons	C1orf21	ENSG00000116667	0,771	0,946298144	0,542	0,71	0,24
CADPS2+ neurons	TSPAN9	ENSG00000011105	0,751	1,135777672	0,502	0,58	0,11
CADPS2+ neurons	SCN8A	ENSG00000196876	0,771	1,022719919	0,542	0,69	0,23
CADPS2+ neurons	PCBP3	ENSG00000183570	0,779	1,02077101	0,558	0,71	0,25
CADPS2+ neurons	GRID2	ENSG00000152208	0,844	0,969150871	0,688	0,98	0,52
CADPS2+ neurons	CFAP299	ENSG00000197826	0,734	1,084444667	0,468	0,51	0,05
CADPS2+ neurons	GPR158	ENSG00000151025	0,802	1,26926028	0,604	0,78	0,32
CADPS2+ neurons	ARHGAP44	ENSG00000006740	0,758	1,096229211	0,516	0,66	0,2
CADPS2+ neurons	GRAMD1B	ENSG00000023171	0,774	1,068856908	0,548	0,71	0,26
CADPS2+ neurons	PDE10A	ENSG00000112541	0,773	1,258545359	0,546	0,72	0,27
CADPS2+ neurons	LRCH1	ENSG00000136141	0,767	0,855360081	0,534	0,75	0,3
CADPS2+ neurons	SGCZ	ENSG00000185053	0,731	1,041250763	0,462	0,64	0,2
CADPS2+ neurons	KCNH7	ENSG00000184611	0,735	1,225311505	0,47	0,57	0,13
CADPS2+ neurons	CACNB2	ENSG00000165995	0,736	0,768758949	0,472	0,73	0,29
CADPS2+ neurons	ADD2	ENSG00000075340	0,745	1,007405123	0,49	0,57	0,13
CADPS2+ neurons	IQSEC3	ENSG00000120645	0,738	1,02558318	0,476	0,54	0,11
CADPS2+ neurons	KCNK1	ENSG00000135750	0,781	1,14363409	0,562	0,71	0,27
CADPS2+ neurons	PLD5	ENSG00000180287	0,82	1,165399902	0,64	0,86	0,42
CADPS2+ neurons	KCND3	ENSG00000171385	0,752	1,126814758	0,504	0,64	0,21
CADPS2+ neurons	CDON	ENSG00000064309	0,744	1,188474489	0,488	0,55	0,12
CADPS2+ neurons	LIMA1	ENSG00000050405	0,795	1,347953085	0,59	0,7	0,27
CADPS2+ neurons	CCDC175	ENSG00000151838	0,726	1,131420882	0,452	0,49	0,06
CADPS2+ neurons	WSCD2	ENSG00000075035	0,726	1,025614162	0,452	0,48	0,05
CADPS2+ neurons	TUSC3	ENSG00000104723	0,739	0,975747557	0,478	0,58	0,15
CADPS2+ neurons	CLIP1	ENSG00000130779	0,82	1,138976672	0,64	0,81	0,38
CADPS2+ neurons	EXPH5	ENSG00000110723	0,718	1,044236687	0,436	0,48	0,06

CADPS2+ neurons	ARHGAP29	ENSG00000137962	0,717	0,658605438	0,434	0,57	0,14
CADPS2+ neurons	SEZ6L	ENSG00000100095	0,718	0,899611452	0,436	0,6	0,18
CADPS2+ neurons	ADAM22	ENSG00000008277	0,839	1,277336287	0,678	0,83	0,4
CADPS2+ neurons	KCNC1	ENSG00000129159	0,732	0,918286412	0,464	0,53	0,11
CADPS2+ neurons	ALS2	ENSG00000003393	0,782	1,172662254	0,564	0,69	0,27
CADPS2+ neurons	GPRIN3	ENSG00000185477	0,731	0,973825999	0,462	0,58	0,16
CADPS2+ neurons	ZFYVE28	ENSG00000159733	0,779	1,086869824	0,558	0,72	0,3
CADPS2+ neurons	MMP24	ENSG00000125966	0,736	1,081095847	0,472	0,54	0,13
CADPS2+ neurons	STXBP1	ENSG00000136854	0,777	1,003458479	0,554	0,73	0,31
CADPS2+ neurons	SYNE1	ENSG00000131018	0,942	1,664489256	0,884	0,98	0,57
CADPS2+ neurons	SHROOM3	ENSG00000138771	0,716	0,950792127	0,432	0,52	0,11
CADPS2+ neurons	PLCL2	ENSG00000154822	0,743	0,936467887	0,486	0,64	0,24
CADPS2+ neurons	SNAP25-AS1	ENSG00000227906	0,722	1,035959409	0,444	0,51	0,1
CADPS2+ neurons	DNM1	ENSG00000106976	0,727	0,874696028	0,454	0,59	0,19
CADPS2+ neurons	ADAMTS16	ENSG00000145536	0,706	1,077010064	0,412	0,43	0,02
CADPS2+ neurons	ST7	ENSG00000004866	0,802	1,125357485	0,604	0,79	0,39
CADPS2+ neurons	HCN1	ENSG00000164588	0,712	1,016921978	0,424	0,51	0,1
CADPS2+ neurons	RAPGEF4	ENSG00000091428	0,755	0,921849836	0,51	0,75	0,35
CADPS2+ neurons	SVEP1	ENSG00000165124	0,718	0,856917357	0,436	0,57	0,17
CADPS2+ neurons	CEP112	ENSG00000154240	0,729	0,898009373	0,458	0,68	0,28
CADPS2+ neurons	RASGRP1	ENSG00000172575	0,704	0,967515678	0,408	0,43	0,04
CADPS2+ neurons	MPP7	ENSG00000150054	0,712	1,0606733	0,424	0,48	0,08
CADPS2+ neurons	GALNT13	ENSG00000144278	0,82	1,006310929	0,64	0,91	0,51
CADPS2+ neurons	DAB1	ENSG00000173406	0,749	0,743740085	0,498	0,78	0,39
CADPS2+ neurons	CACNA1C	ENSG00000151067	0,717	0,5936623	0,434	0,75	0,36
CADPS2+ neurons	CDH10	ENSG00000040731	0,706	0,740237817	0,412	0,58	0,19
CADPS2+ neurons	TRIM9	ENSG00000100505	0,829	1,015675256	0,658	0,91	0,52
CADPS2+ neurons	CPE	ENSG00000109472	0,755	0,850678523	0,51	0,76	0,37
CADPS2+ neurons	GRIA4	ENSG00000152578	0,825	0,964462309	0,65	0,94	0,55
CADPS2+ neurons	GAP43	ENSG00000172020	0,714	0,994964158	0,428	0,52	0,13
CADPS2+ neurons	PIP5K1B	ENSG00000107242	0,721	1,121736975	0,442	0,55	0,16
CADPS2+ neurons	LIN7A	ENSG00000111052	0,712	0,837375128	0,424	0,53	0,15
CADPS2+ neurons	POLB	ENSG00000070501	0,719	0,873618733	0,438	0,53	0,15
CADPS2+ neurons	PPP3CA	ENSG00000138814	0,821	1,058364517	0,642	0,93	0,55
CADPS2+ neurons	ADAMTS18	ENSG00000140873	0,711	0,818088167	0,422	0,59	0,22
CADPS2+ neurons	RAP1GAP2	ENSG00000132359	0,714	0,906781899	0,428	0,55	0,18
CADPS2+ neurons	RORA	ENSG00000069667	0,784	0,791220539	0,568	0,95	0,58
CADPS2+ neurons	SPTB	ENSG00000070182	0,703	0,887366873	0,406	0,47	0,09
CADPS2+ neurons	CACNG2	ENSG00000166862	0,706	1,140100424	0,412	0,49	0,12
CADPS2+ neurons	BRINP1	ENSG00000078725	0,707	0,904045364	0,414	0,55	0,18
CADPS2+ neurons	CADM3	ENSG00000162706	0,703	0,853222729	0,406	0,49	0,13
CADPS2+ neurons	BMPER	ENSG00000164619	0,702	1,240819479	0,404	0,48	0,11
CADPS2+ neurons	DPF3	ENSG00000205683	0,757	0,846641078	0,514	0,75	0,39
CADPS2+ neurons	NRXN3	ENSG00000021645	0,871	1,031980454	0,742	1	0,64
CADPS2+ neurons	GLCE	ENSG00000138604	0,752	0,984112971	0,504	0,7	0,34
CADPS2+ neurons	PAG1	ENSG00000076641	0,731	0,734921218	0,462	0,71	0,35
CADPS2+ neurons	ST8SIA5	ENSG00000101638	0,701	0,973396765	0,402	0,49	0,14
CADPS2+ neurons	STK10	ENSG00000072786	0,705	0,883814314	0,41	0,53	0,18
CADPS2+ neurons	CAMK1D	ENSG00000183049	0,702	0,708878357	0,404	0,67	0,32
CADPS2+ neurons	DYNC1I1	ENSG00000158560	0,779	0,898698843	0,558	0,86	0,52
CADPS2+ neurons	SETBP1	ENSG00000152217	0,736	0,850032853	0,472	0,72	0,38
CADPS2+ neurons	UNC79	ENSG00000133958	0,728	0,791577882	0,456	0,79	0,45
CADPS2+ neurons	JAKMIP2	ENSG00000176049	0,842	1,050894358	0,684	0,92	0,6
CADPS2+ neurons	NEGR1	ENSG00000172260	0,842	1,107899666	0,684	0,95	0,63
CADPS2+ neurons	MAML3	ENSG00000196782	0,73	0,832636785	0,46	0,78	0,46

CADPS2+ neurons	NKAIN2	ENSG00000188580	0,74	0,700616497	0,48	0,97	0,66
CADPS2+ neurons	DPP6	ENSG00000130226	0,855	1,099819237	0,71	0,98	0,68
CADPS2+ neurons	KLHL3	ENSG00000146021	0,713	0,648685833	0,426	0,69	0,39
CADPS2+ neurons	PRKCZ	ENSG00000067606	0,722	0,785578973	0,444	0,65	0,35
CADPS2+ neurons	ATP2B1	ENSG00000070961	0,732	0,908466203	0,464	0,67	0,37
CADPS2+ neurons	FAM13A	ENSG00000138640	0,703	0,661217653	0,406	0,66	0,36
CADPS2+ neurons	MEIS1	ENSG00000143995	0,706	0,716552946	0,412	0,7	0,41
CADPS2+ neurons	NBEA	ENSG00000172915	0,811	0,990401087	0,622	0,88	0,6
CADPS2+ neurons	OXR1	ENSG00000164830	0,73	0,887552849	0,46	0,73	0,45
CADPS2+ neurons	CBLB	ENSG00000114423	0,744	0,832855499	0,488	0,73	0,45
CADPS2+ neurons	MICU1	ENSG00000107745	0,741	0,814045893	0,482	0,74	0,46
CADPS2+ neurons	SMYD3	ENSG00000185420	0,78	0,876703342	0,56	0,89	0,62
CADPS2+ neurons	KAZN	ENSG00000189337	0,757	0,66221684	0,514	0,95	0,67
CADPS2+ neurons	SRGAP3	ENSG00000196220	0,804	1,008070216	0,608	0,86	0,59
CADPS2+ neurons	TTC7B	ENSG00000165914	0,74	0,921853061	0,48	0,71	0,44
CADPS2+ neurons	FOXN3	ENSG00000053254	0,761	0,626064643	0,522	0,91	0,64
CADPS2+ neurons	GALNT7	ENSG00000109586	0,709	0,839344542	0,418	0,64	0,38
CADPS2+ neurons	ERC1	ENSG00000082805	0,855	1,007387547	0,71	0,97	0,7
CADPS2+ neurons	FUT9	ENSG00000172461	0,714	0,652529856	0,428	0,82	0,55
CADPS2+ neurons	KIF5C	ENSG00000168280	0,752	0,730467347	0,504	0,84	0,58
CADPS2+ neurons	PRKCE	ENSG00000171132	0,742	0,758957509	0,484	0,79	0,53
CADPS2+ neurons	MAP1B	ENSG00000131711	0,789	0,728825415	0,578	0,93	0,68
CADPS2+ neurons	TSPAN5	ENSG00000168785	0,797	0,889705729	0,594	0,91	0,65
CADPS2+ neurons	SPOCK1	ENSG00000152377	0,744	0,637341688	0,488	0,95	0,7
CADPS2+ neurons	PDE4DIP	ENSG00000178104	0,817	0,816033756	0,634	0,96	0,71
CADPS2+ neurons	RBFOX2	ENSG00000100320	0,737	0,714034663	0,474	0,81	0,56
CADPS2+ neurons	ANKS1B	ENSG00000185046	0,894	1,102245017	0,788	1	0,75
CADPS2+ neurons	CHD7	ENSG00000171316	0,76	0,862719905	0,52	0,83	0,59
CADPS2+ neurons	MAGI1	ENSG00000151276	0,871	1,038017737	0,742	0,98	0,74
CADPS2+ neurons	EPB41L3	ENSG00000082397	0,701	0,719897038	0,402	0,71	0,48
CADPS2+ neurons	AC124312.1	ENSG00000214265	0,73	0,616739448	0,46	0,83	0,61
CADPS2+ neurons	HECTD4	ENSG00000173064	0,739	0,653006008	0,478	0,83	0,62
CADPS2+ neurons	CADM1	ENSG00000182985	0,874	1,018239227	0,748	0,99	0,79
CADPS2+ neurons	CAMK2D	ENSG00000145349	0,719	0,667175024	0,438	0,79	0,6
CADPS2+ neurons	ANKRD36	ENSG00000135976	0,713	0,723485143	0,426	0,72	0,52
CADPS2+ neurons	ADGRB3	ENSG00000135298	0,939	1,261192088	0,878	0,99	0,81
CADPS2+ neurons	CUX1	ENSG00000257923	0,747	0,750673397	0,494	0,86	0,68
CADPS2+ neurons	GLS	ENSG00000115419	0,708	0,757521229	0,416	0,71	0,53
CADPS2+ neurons	MICAL3	ENSG00000243156	0,721	0,67769632	0,442	0,82	0,65
CADPS2+ neurons	BICD1	ENSG00000151746	0,713	0,662328205	0,426	0,77	0,6
CADPS2+ neurons	WAC	ENSG00000095787	0,739	0,657161561	0,478	0,84	0,68
CADPS2+ neurons	RABGAP1L	ENSG00000152061	0,764	0,756795375	0,528	0,91	0,76
CADPS2+ neurons	STXBP5	ENSG00000164506	0,703	0,720059669	0,406	0,74	0,6
CADPS2+ neurons	NFIA	ENSG00000162599	0,789	0,739417036	0,578	0,96	0,82
CADPS2+ neurons	JMJD1C	ENSG00000171988	0,816	0,877343567	0,632	0,94	0,86
CADPS2+ neurons	MED13L	ENSG00000123066	0,719	0,621740245	0,438	0,88	0,81
GABA	SPHKAP	ENSG00000153820	0,989	2,647609868	0,978	0,99	0,06
GABA	SYNPR	ENSG00000163630	0,986	2,686348206	0,972	0,98	0,06
GABA	OTX2-AS1	ENSG00000248550	0,978	2,573779936	0,956	0,96	0,05
GABA	CHRM2	ENSG00000181072	0,979	2,371909041	0,958	0,97	0,06
GABA	ZMAT4	ENSG00000165061	0,986	2,283799187	0,972	0,99	0,08
GABA	GAD2	ENSG00000136750	0,964	1,919867344	0,928	0,95	0,05
GABA	NYAP2	ENSG00000144460	0,982	2,298007962	0,964	0,97	0,09
GABA	UNC13C	ENSG00000137766	0,969	2,172139611	0,938	0,97	0,09
GABA	GALNT14	ENSG00000158089	0,964	2,020474157	0,928	0,94	0,06

GABA	NELL1	ENSG00000165973	0,961	2,360182511	0,922	0,95	0,08
GABA	LINGO2	ENSG00000174482	0,959	2,11564076	0,918	0,98	0,13
GABA	GALNTL6	ENSG00000174473	0,984	2,906185639	0,968	0,99	0,14
GABA	GRIK1	ENSG00000171189	0,998	3,394927869	0,996	1	0,15
GABA	GULP1	ENSG00000144366	0,952	1,850886341	0,904	0,94	0,09
GABA	NR2F2-AS1	ENSG00000247809	0,961	1,977978347	0,922	0,97	0,13
GABA	UNC5D	ENSG00000156687	0,948	1,87900968	0,896	0,98	0,14
GABA	SYN3	ENSG00000185666	0,98	2,293378963	0,96	0,99	0,15
GABA	ZNF385D	ENSG00000151789	0,956	1,732230676	0,912	0,98	0,14
GABA	CELF4	ENSG00000101489	0,944	1,602257529	0,888	0,97	0,14
GABA	BTBD11	ENSG00000151136	0,966	2,015712317	0,932	0,96	0,13
GABA	LINC01414	ENSG00000253554	0,947	1,892171925	0,894	0,94	0,11
GABA	SYT1	ENSG00000067715	0,99	2,753031609	0,98	1	0,17
GABA	SRRM3	ENSG00000177679	0,951	1,562572223	0,902	0,97	0,14
GABA	KIT	ENSG00000157404	0,927	1,50867724	0,854	0,88	0,05
GABA	GRIN1	ENSG00000176884	0,943	1,537260097	0,886	0,96	0,13
GABA	RORB	ENSG00000198963	0,939	1,699842726	0,878	0,94	0,11
GABA	LINC01210	ENSG00000239513	0,915	1,174313518	0,83	0,84	0,01
GABA	PTCHD4	ENSG00000244694	0,927	1,478311548	0,854	0,91	0,08
GABA	SYN2	ENSG00000157152	0,966	2,039598346	0,932	0,97	0,15
GABA	SAMD5	ENSG00000203727	0,92	1,47586386	0,84	0,9	0,08
GABA	CUX2	ENSG00000111249	0,922	1,449598794	0,844	0,9	0,08
GABA	CPNE4	ENSG00000196353	0,93	1,729282361	0,86	0,91	0,1
GABA	FRMPD4	ENSG00000169933	0,966	2,107815977	0,932	0,98	0,17
GABA	CNTN4	ENSG00000144619	0,969	2,244684221	0,938	0,99	0,18
GABA	GAD1	ENSG00000128683	0,925	1,513654713	0,85	0,9	0,08
GABA	GABBR2	ENSG00000136928	0,93	1,444644685	0,86	0,94	0,13
GABA	NRG1	ENSG00000157168	0,932	2,044739082	0,864	0,95	0,15
GABA	DOK6	ENSG00000206052	0,954	1,787237585	0,908	0,97	0,17
GABA	SLC4A10	ENSG00000144290	0,941	1,620011025	0,882	0,95	0,15
GABA	SGCZ	ENSG00000185053	0,943	1,858623351	0,886	0,98	0,19
GABA	MYT1L	ENSG00000186487	0,902	1,298351242	0,804	0,97	0,17
GABA	CNTNAP5	ENSG00000155052	0,968	2,340838035	0,936	0,99	0,19
GABA	KCNJ6	ENSG00000157542	0,921	1,33508856	0,842	0,9	0,11
GABA	GRIN2B	ENSG00000273079	0,933	1,518988977	0,866	0,95	0,15
GABA	VAV3	ENSG00000134215	0,912	1,474312763	0,824	0,88	0,09
GABA	PRICKLE1	ENSG00000139174	0,929	1,586069816	0,858	0,91	0,13
GABA	GRIA1	ENSG00000155511	0,891	1,178327323	0,782	0,92	0,13
GABA	RIMS2	ENSG00000176406	0,938	1,711792365	0,876	0,99	0,21
GABA	TOX	ENSG00000198846	0,942	1,682040428	0,884	0,97	0,19
GABA	GRIP2	ENSG00000144596	0,911	1,315199936	0,822	0,84	0,06
GABA	PAK3	ENSG00000077264	0,906	1,341331338	0,812	0,94	0,15
GABA	KCNJ3	ENSG00000162989	0,926	1,485582623	0,852	0,94	0,16
GABA	ROBO2	ENSG00000185008	0,972	2,491631027	0,944	1	0,22
GABA	CDH13	ENSG00000140945	0,956	1,797938547	0,912	0,98	0,2
GABA	LYPD6B	ENSG00000150556	0,897	1,257055456	0,794	0,82	0,04
GABA	KIAA1549L	ENSG00000110427	0,924	1,404823164	0,848	0,92	0,14
GABA	GRM5	ENSG00000168959	0,955	1,905480605	0,91	0,99	0,21
GABA	OPCML	ENSG00000183715	0,915	1,529749488	0,83	0,99	0,22
GABA	SHANK2	ENSG00000162105	0,933	1,495582205	0,866	0,94	0,17
GABA	CACNA1B	ENSG00000148408	0,888	1,163461345	0,776	0,95	0,18
GABA	RBFOX3	ENSG00000167281	0,898	1,157118278	0,796	0,86	0,09
GABA	MIR124-2HG	ENSG00000254377	0,906	1,249970729	0,812	0,85	0,08
GABA	AFF2	ENSG00000155966	0,897	1,237364955	0,794	0,85	0,08
GABA	IGF1	ENSG00000017427	0,886	1,200808043	0,772	0,79	0,02

GABA	PDZRN3	ENSG00000121440	0,933	1,649977028	0,866	0,95	0,18
GABA	FOXP2	ENSG00000128573	0,904	1,387317363	0,808	0,94	0,18
GABA	ATP8A2	ENSG00000132932	0,91	1,38169639	0,82	0,96	0,2
GABA	FAT3	ENSG00000165323	0,955	1,796782432	0,91	0,99	0,22
GABA	GUCY1A1	ENSG00000164116	0,903	1,309816995	0,806	0,86	0,1
GABA	AL033504.1	ENSG00000227681	0,889	1,339871874	0,778	0,84	0,08
GABA	GABRB3	ENSG00000166206	0,932	1,513750082	0,864	0,95	0,19
GABA	RAB3C	ENSG00000152932	0,909	1,350690844	0,818	0,92	0,16
GABA	KSR2	ENSG00000171435	0,906	1,294100071	0,812	0,94	0,18
GABA	LRFN5	ENSG00000165379	0,898	1,290413426	0,796	0,95	0,19
GABA	CADPS	ENSG00000163618	0,991	2,403798834	0,982	1	0,24
GABA	PAK5	ENSG00000101349	0,907	1,255947147	0,814	0,89	0,13
GABA	CA10	ENSG00000154975	0,887	1,341440112	0,774	0,92	0,16
GABA	LRFN2	ENSG00000156564	0,911	1,426294635	0,822	0,88	0,12
GABA	DLGAP2	ENSG00000198010	0,878	1,113955463	0,756	0,9	0,14
GABA	RELN	ENSG00000189056	0,88	1,122523983	0,76	0,86	0,11
GABA	AMPH	ENSG00000078053	0,901	1,182339531	0,802	0,91	0,16
GABA	PTPRN2	ENSG00000155093	0,914	1,334048527	0,828	0,97	0,23
GABA	RGS6	ENSG00000182732	0,914	1,582186237	0,828	0,94	0,19
GABA	PDE3A	ENSG00000172572	0,886	1,291455315	0,772	0,87	0,12
GABA	CELF5	ENSG00000161082	0,882	1,02414637	0,764	0,87	0,13
GABA	OLFM3	ENSG00000118733	0,893	1,279052469	0,786	0,89	0,14
GABA	XKR4	ENSG00000206579	0,882	1,225444028	0,764	0,93	0,18
GABA	B4GALT6	ENSG00000118276	0,908	1,324166185	0,816	0,86	0,12
GABA	PTPRO	ENSG00000151490	0,892	1,350520964	0,784	0,87	0,13
GABA	LAMB1	ENSG00000091136	0,882	1,057593932	0,764	0,8	0,06
GABA	GREM2	ENSG00000180875	0,881	1,263993869	0,762	0,79	0,05
GABA	GRIN2A	ENSG00000183454	0,909	1,667478628	0,818	0,89	0,15
GABA	NR2F2	ENSG00000185551	0,873	1,060381371	0,746	0,82	0,08
GABA	SNAP25	ENSG00000132639	0,943	1,622563451	0,886	0,97	0,23
GABA	RALYL	ENSG00000184672	0,909	1,426153167	0,818	0,99	0,26
GABA	EPHA4	ENSG00000116106	0,876	1,07758026	0,752	0,85	0,11
GABA	SNTG1	ENSG00000147481	0,848	0,958452766	0,696	0,93	0,19
GABA	STXBP5L	ENSG00000145087	0,893	1,234952586	0,786	0,97	0,24
GABA	PCBP3	ENSG00000183570	0,958	1,858586875	0,916	0,97	0,24
GABA	ELMOD1	ENSG00000110675	0,9	1,221212582	0,8	0,89	0,16
GABA	FAM135B	ENSG00000147724	0,939	1,459690342	0,878	0,96	0,23
GABA	TAFA2	ENSG00000198673	0,905	1,523490288	0,81	0,94	0,22
GABA	EFNA5	ENSG00000184349	0,88	1,351668959	0,76	0,88	0,16
GABA	ATP2B2	ENSG00000157087	0,923	1,448700456	0,846	0,94	0,22
GABA	NHS	ENSG00000188158	0,876	1,092922757	0,752	0,88	0,16
GABA	IGSF3	ENSG00000143061	0,872	1,019742463	0,744	0,77	0,05
GABA	LYPD6	ENSG00000187123	0,863	1,077175376	0,726	0,78	0,06
GABA	KHDRBS2	ENSG00000112232	0,929	1,584325041	0,858	0,96	0,25
GABA	FLRT2	ENSG00000185070	0,878	1,118983491	0,756	0,92	0,21
GABA	KCNIP4	ENSG00000185774	0,991	2,87256816	0,982	1	0,29
GABA	DLGAP1	ENSG00000170579	0,992	2,839151733	0,984	1	0,29
GABA	TRPC5	ENSG00000072315	0,867	1,244012597	0,734	0,8	0,09
GABA	SYT7	ENSG00000011347	0,866	0,898775844	0,732	0,78	0,07
GABA	PTPRT	ENSG00000196090	0,876	1,655021039	0,752	0,86	0,15
GABA	NOL4	ENSG00000101746	0,889	1,174154619	0,778	0,93	0,22
GABA	GRM7	ENSG00000196277	0,851	1,005816243	0,702	0,94	0,23
GABA	ANKRD30BL	ENSG00000163046	0,85	0,992414633	0,7	0,88	0,17
GABA	FGF14	ENSG00000102466	0,935	1,69958546	0,87	0,99	0,29
GABA	SCN2A	ENSG00000136531	0,865	1,010418904	0,73	0,91	0,2

GABA	KCTD16	ENSG00000183775	0,898	1,376447679	0,796	0,93	0,22
GABA	ANKRD34C-AS1	ENSG00000259234	0,864	1,085746487	0,728	0,79	0,08
GABA	HS3ST2	ENSG00000122254	0,858	1,095425728	0,716	0,74	0,04
GABA	KCND2	ENSG00000184408	0,957	2,059504723	0,914	0,99	0,29
GABA	RGS7BP	ENSG00000186479	0,873	1,122198867	0,746	0,81	0,11
GABA	CHD5	ENSG00000116254	0,851	0,816209489	0,702	0,81	0,1
GABA	SLC2A13	ENSG00000151229	0,896	1,201517465	0,792	0,94	0,24
GABA	ANK1	ENSG00000029534	0,853	0,918005087	0,706	0,8	0,1
GABA	PLCB4	ENSG00000101333	0,902	1,302562637	0,804	0,95	0,25
GABA	LRRTM4	ENSG00000176204	0,905	1,458041991	0,81	1	0,3
GABA	GABRB2	ENSG00000145864	0,847	0,99119138	0,694	0,82	0,12
GABA	ZFPM2	ENSG00000169946	0,942	1,714288768	0,884	0,99	0,29
GABA	TMEM108	ENSG00000144868	0,952	1,749097426	0,904	0,97	0,28
GABA	SYNDIG1L	ENSG00000183379	0,853	0,928974568	0,706	0,72	0,02
GABA	SLC8A1	ENSG00000183023	0,934	1,59564252	0,868	1	0,3
GABA	SLC6A11	ENSG00000132164	0,842	0,745199513	0,684	0,82	0,12
GABA	IQSEC3	ENSG00000120645	0,859	0,922125628	0,718	0,8	0,1
GABA	B3GLCT	ENSG00000187676	0,855	0,946947683	0,71	0,81	0,11
GABA	OSBPL6	ENSG00000079156	0,92	1,520107822	0,84	0,94	0,24
GABA	CCSER1	ENSG00000184305	0,92	1,49222058	0,84	0,99	0,29
GABA	CDH12	ENSG00000154162	0,896	1,802703624	0,792	0,88	0,18
GABA	ADGRB1	ENSG00000181790	0,865	0,963464401	0,73	0,82	0,12
GABA	VSNL1	ENSG00000163032	0,847	0,905317336	0,694	0,8	0,11
GABA	ARHGAP44	ENSG00000006740	0,869	1,037249787	0,738	0,89	0,2
GABA	FRMD3	ENSG00000172159	0,865	0,956286613	0,73	0,87	0,18
GABA	PCDH7	ENSG00000169851	0,886	1,26323873	0,772	0,97	0,29
GABA	NPAS2	ENSG00000170485	0,863	0,972014836	0,726	0,92	0,23
GABA	DSCAM	ENSG00000171587	0,921	1,456355375	0,842	1	0,31
GABA	CNKSR2	ENSG00000149970	0,871	1,167768549	0,742	0,88	0,19
GABA	SLC8A3	ENSG00000100678	0,846	0,865627549	0,692	0,83	0,15
GABA	SH3GL2	ENSG00000107295	0,87	1,292463084	0,74	0,85	0,17
GABA	ATRNL1	ENSG00000107518	0,833	0,860479784	0,666	0,9	0,22
GABA	SRRM4	ENSG00000139767	0,847	0,976785024	0,694	0,78	0,1
GABA	MIAT	ENSG00000225783	0,842	0,876893558	0,684	0,82	0,14
GABA	WNK2	ENSG00000165238	0,838	0,83892308	0,676	0,85	0,18
GABA	ARHGEF33	ENSG00000214694	0,846	0,925283923	0,692	0,73	0,05
GABA	SCN3A	ENSG00000153253	0,825	0,809945183	0,65	0,85	0,17
GABA	SEZ6L	ENSG00000100095	0,824	0,808260133	0,648	0,85	0,17
GABA	VSTM2L	ENSG00000132821	0,848	1,017057195	0,696	0,74	0,07
GABA	CACNA1D	ENSG00000157388	0,955	1,94224382	0,91	0,96	0,29
GABA	NSG2	ENSG00000170091	0,853	1,012535087	0,706	0,81	0,14
GABA	SV2C	ENSG00000122012	0,843	1,159715455	0,686	0,73	0,06
GABA	BCL11A	ENSG00000119866	0,838	0,85578602	0,676	0,73	0,06
GABA	PLCH1	ENSG00000114805	0,835	0,902484946	0,67	0,78	0,11
GABA	EPHA3	ENSG00000044524	0,838	1,025617998	0,676	0,77	0,11
GABA	JPH4	ENSG00000092051	0,839	0,841227784	0,678	0,79	0,12
GABA	AGBL4	ENSG00000186094	0,832	0,86935489	0,664	0,93	0,27
GABA	EML6	ENSG00000214595	0,886	1,215620322	0,772	0,91	0,24
GABA	SCN8A	ENSG00000196876	0,867	1,036023212	0,734	0,89	0,22
GABA	CDH10	ENSG00000040731	0,847	1,050027343	0,694	0,85	0,18
GABA	FAM155A	ENSG00000204442	0,959	1,861710595	0,918	1	0,34
GABA	SLC12A5	ENSG00000124140	0,847	0,979615787	0,694	0,75	0,08
GABA	FBN2	ENSG00000138829	0,836	0,968938244	0,672	0,71	0,05
GABA	PLCXD3	ENSG00000182836	0,836	0,957609471	0,672	0,77	0,11

GABA	STXBP5-AS1	ENSG00000233452	0,843	1,057201286	0,686	0,83	0,17
GABA	ADCY8	ENSG00000155897	0,832	0,843447485	0,664	0,87	0,21
GABA	OTX2	ENSG00000165588	0,831	0,837022813	0,662	0,68	0,02
GABA	RYR2	ENSG00000198626	0,906	1,72491355	0,812	0,95	0,29
GABA	LINC00599	ENSG00000253230	0,84	0,853693711	0,68	0,75	0,09
GABA	SORBS2	ENSG00000154556	0,819	0,720597173	0,638	0,9	0,24
GABA	SIPA1L2	ENSG00000116991	0,869	1,062183248	0,738	0,88	0,23
GABA	SPTBN4	ENSG00000160460	0,856	0,97264885	0,712	0,9	0,25
GABA	GALNT18	ENSG00000110328	0,851	0,854746673	0,702	0,9	0,25
GABA	TSPAN18	ENSG00000157570	0,828	0,783538634	0,656	0,7	0,05
GABA	CACNA1E	ENSG00000198216	0,814	0,713433734	0,628	0,81	0,15
GABA	KIRREL1	ENSG00000183853	0,828	0,757996409	0,656	0,71	0,06
GABA	CAMK2B	ENSG00000058404	0,828	0,833711541	0,656	0,83	0,18
GABA	CACHD1	ENSG00000158966	0,845	0,988067465	0,69	0,88	0,23
GABA	NCALD	ENSG00000104490	0,844	1,012550422	0,688	0,83	0,18
GABA	CACNB4	ENSG00000182389	0,835	0,995904634	0,67	0,76	0,11
GABA	LDB2	ENSG00000169744	0,833	1,006503329	0,666	0,83	0,19
GABA	CACNA1A	ENSG00000141837	0,87	1,313194694	0,74	0,93	0,28
GABA	SDK1	ENSG00000146555	0,864	1,476696257	0,728	0,91	0,26
GABA	MDGA2	ENSG00000139915	0,949	1,728672957	0,898	0,99	0,35
GABA	SYT16	ENSG00000139973	0,819	0,792259878	0,638	0,81	0,16
GABA	UNC80	ENSG00000144406	0,797	0,654157993	0,594	0,87	0,23
GABA	ADARB1	ENSG00000197381	0,853	1,001188954	0,706	0,82	0,18
GABA	PLCB1	ENSG00000182621	0,957	1,97142274	0,914	0,99	0,35
GABA	ZEB1	ENSG00000148516	0,922	1,356258245	0,844	0,99	0,35
GABA	GPM6A	ENSG00000150625	0,89	1,240226498	0,78	0,98	0,34
GABA	SIDT1	ENSG00000072858	0,824	0,79454125	0,648	0,76	0,12
GABA	GATA3	ENSG00000107485	0,82	0,69200817	0,64	0,66	0,02
GABA	PHACTR2	ENSG00000112419	0,838	0,888664568	0,676	0,86	0,23
GABA	LIN7A	ENSG00000111052	0,815	0,727567393	0,63	0,77	0,14
GABA	NRSN1	ENSG00000152954	0,825	0,811313907	0,65	0,74	0,1
GABA	ANTXR1	ENSG00000169604	0,824	0,810049999	0,648	0,84	0,21
GABA	SCN1A	ENSG00000144285	0,792	0,669891802	0,584	0,84	0,2
GABA	SNRPN	ENSG00000128739	0,844	0,943297332	0,688	0,9	0,27
GABA	CNTN5	ENSG00000149972	0,816	0,765440942	0,632	0,78	0,15
GABA	CSMD3	ENSG00000164796	0,844	0,93530077	0,688	0,98	0,35
GABA	DNM1	ENSG00000106976	0,827	0,855773332	0,654	0,81	0,18
GABA	GUCY1A2	ENSG00000152402	0,82	0,904866289	0,64	0,83	0,21
GABA	HS6ST3	ENSG00000185352	0,811	0,971360303	0,622	0,83	0,2
GABA	ASIC4	ENSG00000072182	0,814	0,711803981	0,628	0,67	0,04
GABA	CACNA1C	ENSG00000151067	0,901	1,244960428	0,802	0,98	0,35
GABA	PPM1E	ENSG00000175175	0,855	0,997258103	0,71	0,91	0,28
GABA	DLGAP3	ENSG00000116544	0,824	0,749269244	0,648	0,69	0,07
GABA	AKAP12	ENSG00000131016	0,816	0,694857369	0,632	0,81	0,18
GABA	PRSS12	ENSG00000164099	0,815	0,820137223	0,63	0,64	0,02
GABA	KCNIP2	ENSG00000120049	0,818	0,733758117	0,636	0,69	0,06
GABA	CAMK4	ENSG00000152495	0,815	0,837783471	0,63	0,77	0,14
GABA	CASC15	ENSG00000272168	0,823	0,752566565	0,646	0,81	0,18
GABA	NMNAT2	ENSG00000157064	0,796	0,667737757	0,592	0,79	0,17
GABA	CNTN1	ENSG00000018236	0,792	0,695073684	0,584	0,96	0,34
GABA	CASR	ENSG00000036828	0,812	0,952282901	0,624	0,63	0,01
GABA	SEZ6	ENSG00000063015	0,819	0,802161638	0,638	0,69	0,07
GABA	DDAH1	ENSG00000153904	0,819	0,798536439	0,638	0,81	0,19
GABA	GRAMD1B	ENSG00000023171	0,84	0,924342236	0,68	0,87	0,25
GABA	GUCY1B1	ENSG00000061918	0,821	0,788738154	0,642	0,75	0,13

GABA	SLC16A10	ENSG00000112394	0,818	0,840409555	0,636	0,7	0,09
GABA	CACNA1G	ENSG00000006283	0,814	0,721115131	0,628	0,68	0,06
GABA	CLVS2	ENSG00000146352	0,812	0,728424462	0,624	0,71	0,09
GABA	SV2A	ENSG00000159164	0,835	0,981389959	0,67	0,8	0,18
GABA	ADD2	ENSG00000075340	0,81	0,732999698	0,62	0,74	0,12
GABA	SLC38A1	ENSG00000111371	0,819	0,754240776	0,638	0,85	0,23
GABA	TENM3	ENSG00000218336	0,803	0,760257487	0,606	0,83	0,21
GABA	ERC2	ENSG00000187672	0,925	1,415890217	0,85	0,97	0,36
GABA	AC092691.1	ENSG00000239268	0,864	1,101518318	0,728	0,98	0,36
GABA	CLSTN3	ENSG00000139182	0,815	0,774949141	0,63	0,72	0,11
GABA	GSG1L	ENSG00000169181	0,805	0,731114495	0,61	0,73	0,12
GABA	PPP4R4	ENSG00000119698	0,806	0,673374201	0,612	0,76	0,15
GABA	ALK	ENSG00000171094	0,821	0,859444851	0,642	0,84	0,23
GABA	DOK5	ENSG00000101134	0,812	0,802382009	0,624	0,74	0,13
GABA	SYBU	ENSG00000147642	0,794	0,649193671	0,588	0,79	0,19
GABA	NRG3	ENSG00000185737	0,887	1,27568676	0,774	1	0,39
GABA	PGM2L1	ENSG00000165434	0,816	0,834121898	0,632	0,73	0,12
GABA	RIMBP2	ENSG00000060709	0,811	0,737796689	0,622	0,76	0,16
GABA	TRMT9B	ENSG00000250305	0,81	0,79638832	0,62	0,75	0,14
GABA	PPP1R14C	ENSG00000198729	0,808	0,718161903	0,616	0,68	0,07
GABA	GPR158	ENSG00000151025	0,873	1,13629373	0,746	0,92	0,31
GABA	LINC01122	ENSG00000233723	0,819	1,046298261	0,638	0,82	0,21
GABA	SCG2	ENSG00000171951	0,81	0,856580982	0,62	0,71	0,11
GABA	GRM4	ENSG00000124493	0,808	0,655860847	0,616	0,66	0,05
GABA	SMAD9	ENSG00000120693	0,821	0,776709155	0,642	0,81	0,21
GABA	AP001347.1	ENSG00000224905	0,809	0,808088143	0,618	0,78	0,17
GABA	KCNQ2	ENSG00000075043	0,805	0,683611613	0,61	0,72	0,12
GABA	CDH8	ENSG00000150394	0,883	1,232462865	0,766	0,92	0,32
GABA	BASP1	ENSG00000176788	0,796	0,710196535	0,592	0,82	0,22
GABA	HTR2A	ENSG00000102468	0,804	0,721709668	0,608	0,64	0,03
GABA	PLAGL1	ENSG00000118495	0,801	0,667463684	0,602	0,72	0,12
GABA	CSMD1	ENSG00000183117	0,923	1,740636952	0,846	0,99	0,39
GABA	ZEB1-AS1	ENSG00000237036	0,802	0,810352511	0,604	0,69	0,09
GABA	N4BP2	ENSG00000078177	0,808	0,740257969	0,616	0,75	0,15
GABA	TOX2	ENSG00000124191	0,8	0,717376893	0,6	0,73	0,13
GABA	LINC02253	ENSG00000259485	0,8	0,862162275	0,6	0,61	0,01
GABA	EPHA6	ENSG00000080224	0,787	0,65503902	0,574	0,73	0,13
GABA	MAGI3	ENSG00000081026	0,861	1,070791565	0,722	0,92	0,32
GABA	RTN4RL1	ENSG00000185924	0,802	0,701027074	0,604	0,65	0,05
GABA	CEP112	ENSG00000154240	0,863	1,397419275	0,726	0,87	0,27
GABA	CDR2	ENSG00000140743	0,826	0,839853815	0,652	0,86	0,27
GABA	AL390957.1	ENSG00000285280	0,809	1,192740506	0,618	0,67	0,07
GABA	KIFC3	ENSG00000140859	0,825	0,837922821	0,65	0,82	0,23
GABA	PARP8	ENSG00000151883	0,816	0,777596742	0,632	0,86	0,26
GABA	GPC6	ENSG00000183098	0,827	1,322102983	0,654	0,81	0,21
GABA	EPB41	ENSG00000159023	0,792	0,668625179	0,584	0,8	0,21
GABA	PACRG	ENSG00000112530	0,821	0,966986771	0,642	0,83	0,23
GABA	SLC27A6	ENSG00000113396	0,81	1,089674437	0,62	0,7	0,11
GABA	SNAP91	ENSG00000065609	0,836	0,895769717	0,672	0,92	0,33
GABA	KLHL23	ENSG00000213160	0,816	0,734835638	0,632	0,82	0,23
GABA	KCNC1	ENSG00000129159	0,806	0,742173224	0,612	0,7	0,11
GABA	RBMS3	ENSG00000144642	0,791	0,703126089	0,582	0,77	0,18
GABA	CDH18	ENSG00000145526	0,784	0,688645402	0,568	0,77	0,18
GABA	MINAR1	ENSG00000169330	0,798	0,656727239	0,596	0,64	0,05
GABA	FMN2	ENSG00000155816	0,816	0,695077046	0,632	0,93	0,34

GABA	AC016717.2	ENSG00000273301	0,798	0,657741315	0,596	0,61	0,02
GABA	C1orf21	ENSG00000116667	0,794	0,673583839	0,588	0,82	0,23
GABA	PAM	ENSG00000145730	0,783	0,633454247	0,566	0,87	0,28
GABA	FRY	ENSG00000073910	0,85	0,951362935	0,7	0,94	0,35
GABA	ANKRD6	ENSG00000135299	0,792	0,68222377	0,584	0,76	0,17
GABA	CARMIL1	ENSG00000079691	0,783	0,617511229	0,566	0,81	0,23
GABA	HTR1E	ENSG00000168830	0,794	0,791575245	0,588	0,63	0,05
GABA	RNF150	ENSG00000170153	0,788	0,66556317	0,576	0,83	0,25
GABA	RAPGEF4	ENSG00000091428	0,813	0,759317975	0,626	0,93	0,34
GABA	MYH10	ENSG00000133026	0,795	0,697405935	0,59	0,78	0,2
GABA	GABRG2	ENSG00000113327	0,782	0,588302042	0,564	0,69	0,11
GABA	CACNA2D3	ENSG00000157445	0,818	1,011974034	0,636	0,87	0,29
GABA	SYT14	ENSG00000143469	0,812	0,770408634	0,624	0,86	0,28
GABA	EPHB1	ENSG00000154928	0,798	0,827141229	0,596	0,77	0,19
GABA	GRM1	ENSG00000152822	0,784	0,725490518	0,568	0,68	0,1
GABA	TMEM178B	ENSG00000261115	0,916	1,379289481	0,832	0,96	0,38
GABA	REEP1	ENSG00000068615	0,787	0,665879679	0,574	0,72	0,14
GABA	FAM49A	ENSG00000197872	0,796	0,664645806	0,592	0,81	0,23
GABA	ZFYVE28	ENSG00000159733	0,835	0,864759112	0,67	0,87	0,29
GABA	AC004805.1	ENSG00000266076	0,794	0,920716528	0,588	0,65	0,07
GABA	HCN1	ENSG00000164588	0,795	1,116476319	0,59	0,67	0,1
GABA	MCU	ENSG00000156026	0,809	0,747056744	0,618	0,84	0,27
GABA	AFF3	ENSG00000144218	0,87	1,122280181	0,74	0,95	0,38
GABA	SUSD4	ENSG00000143502	0,785	0,673048182	0,57	0,71	0,13
GABA	MRTFB	ENSG00000186260	0,807	0,59483761	0,614	0,9	0,33
GABA	GRK3	ENSG00000100077	0,829	0,826541107	0,658	0,91	0,34
GABA	GRIN3A	ENSG00000198785	0,789	0,816699061	0,578	0,64	0,07
GABA	RALGPS2	ENSG00000116191	0,786	0,704617824	0,572	0,77	0,2
GABA	MEIS2	ENSG00000134138	0,928	1,484955896	0,856	0,98	0,41
GABA	NRG2	ENSG00000158458	0,855	0,991653134	0,71	0,88	0,31
GABA	ME3	ENSG00000151376	0,795	0,699271514	0,59	0,78	0,21
GABA	SEMA3E	ENSG00000170381	0,791	0,939598585	0,582	0,69	0,12
GABA	CPLX1	ENSG00000168993	0,792	0,782714491	0,584	0,65	0,08
GABA	RAVER2	ENSG00000162437	0,795	0,725263429	0,59	0,73	0,16
GABA	SOX4	ENSG00000124766	0,787	0,709991345	0,574	0,62	0,06
GABA	NIPAL2	ENSG00000104361	0,787	0,736973955	0,574	0,67	0,1
GABA	PTPRR	ENSG00000153233	0,782	0,75779732	0,564	0,67	0,1
GABA	MAP2	ENSG00000078018	0,84	0,922163434	0,68	0,97	0,4
GABA	DLGAP4	ENSG00000080845	0,823	0,840903666	0,646	0,83	0,26
GABA	INSYN2B	ENSG00000204767	0,779	0,671829689	0,558	0,74	0,17
GABA	LRRC7	ENSG00000033122	0,803	0,886881654	0,606	0,88	0,31
GABA	KCNH5	ENSG00000140015	0,783	0,792506368	0,566	0,65	0,08
GABA	SLC6A17	ENSG00000197106	0,789	0,682538817	0,578	0,65	0,09
GABA	PRICKLE2	ENSG00000163637	0,788	0,616427883	0,576	0,87	0,31
GABA	SYN1	ENSG00000008056	0,786	0,687499636	0,572	0,67	0,1
GABA	ZNF385B	ENSG00000144331	0,781	0,869549726	0,562	0,68	0,11
GABA	ANO5	ENSG00000171714	0,789	0,752272965	0,578	0,72	0,16
GABA	NEXMIF	ENSG00000050030	0,774	0,65943075	0,548	0,71	0,15
GABA	VASH2	ENSG00000143494	0,785	0,705280842	0,57	0,59	0,02
GABA	RIMS3	ENSG00000117016	0,788	0,666969093	0,576	0,63	0,07
GABA	NRXN1	ENSG00000179915	0,873	1,209069923	0,746	1	0,44
GABA	ZFR2	ENSG00000105278	0,786	0,6626763	0,572	0,68	0,13
GABA	PLXNC1	ENSG00000136040	0,78	0,632778375	0,56	0,72	0,16
GABA	CELF2	ENSG00000048740	0,877	1,088038275	0,754	0,99	0,43
GABA	GABRA2	ENSG00000151834	0,777	0,674706755	0,554	0,67	0,11

GABA	ATCAY	ENSG00000167654	0,775	0,604259047	0,55	0,7	0,14
GABA	CERS6	ENSG00000172292	0,778	0,647469822	0,556	0,85	0,29
GABA	PCLO	ENSG00000186472	0,812	0,747742273	0,624	0,92	0,37
GABA	PMEPA1	ENSG00000124225	0,788	0,665489342	0,576	0,74	0,19
GABA	NREP	ENSG00000134986	0,783	0,738978904	0,566	0,66	0,1
GABA	PPM1L	ENSG00000163590	0,857	1,048012883	0,714	0,92	0,37
GABA	LMO7	ENSG00000136153	0,797	0,704699573	0,594	0,8	0,25
GABA	SCN3B	ENSG00000166257	0,784	0,764381733	0,568	0,63	0,08
GABA	HPCA	ENSG00000121905	0,78	0,721941212	0,56	0,59	0,03
GABA	AC092683.1	ENSG00000230606	0,809	0,781690757	0,618	0,88	0,33
GABA	LRRC49	ENSG00000137821	0,791	0,666776133	0,582	0,8	0,25
GABA	IRAK1BP1	ENSG00000146243	0,803	0,784246805	0,606	0,84	0,29
GABA	DZIP1	ENSG00000134874	0,775	0,595126056	0,55	0,75	0,2
GABA	KIAA0825	ENSG00000185261	0,793	0,772298585	0,586	0,84	0,29
GABA	FNDC5	ENSG00000160097	0,777	0,634297022	0,554	0,59	0,04
GABA	RGS7	ENSG00000182901	0,872	1,1145233	0,744	0,96	0,42
GABA	NUP93	ENSG00000102900	0,801	0,740851274	0,602	0,8	0,26
GABA	PATJ	ENSG00000132849	0,8	0,73300633	0,6	0,86	0,31
GABA	ICA1	ENSG00000003147	0,776	0,676960268	0,552	0,68	0,13
GABA	ADAMTS19	ENSG00000145808	0,78	0,929968394	0,56	0,66	0,12
GABA	NAALAD2	ENSG00000077616	0,786	0,67527952	0,572	0,78	0,24
GABA	SPTAN1	ENSG00000197694	0,786	0,675890073	0,572	0,86	0,32
GABA	AMMECR1	ENSG00000101935	0,778	0,72065931	0,556	0,69	0,15
GABA	STRIP2	ENSG00000128578	0,774	0,592923622	0,548	0,59	0,06
GABA	SLC44A5	ENSG00000137968	0,772	0,915018492	0,544	0,69	0,16
GABA	SESN3	ENSG00000149212	0,807	0,731051081	0,614	0,85	0,31
GABA	CDK14	ENSG00000058091	0,869	1,086900379	0,738	0,94	0,4
GABA	PLEKHA5	ENSG00000052126	0,853	0,906408305	0,706	0,96	0,43
GABA	ARHGEF11	ENSG00000132694	0,828	0,835400091	0,656	0,89	0,35
GABA	CDH22	ENSG00000149654	0,767	0,616690234	0,534	0,6	0,06
GABA	GRIA3	ENSG00000125675	0,828	0,948305313	0,656	0,88	0,35
GABA	KLF12	ENSG00000118922	0,809	0,847579451	0,618	0,89	0,36
GABA	GNAL	ENSG00000141404	0,765	0,643057633	0,53	0,68	0,15
GABA	NPTN	ENSG00000156642	0,841	0,86730376	0,682	0,88	0,35
GABA	SNCB	ENSG00000074317	0,77	0,691822087	0,54	0,6	0,08
GABA	LINC02055	ENSG00000254101	0,771	2,260755515	0,542	0,57	0,04
GABA	HSPA4L	ENSG00000164070	0,763	0,615804554	0,526	0,73	0,21
GABA	AC087280.2	ENSG00000283415	0,768	0,733062079	0,536	0,59	0,07
GABA	MARK1	ENSG00000116141	0,819	0,832869143	0,638	0,88	0,36
GABA	CTNNA2	ENSG00000066032	0,804	0,717072985	0,608	0,99	0,46
GABA	FIGN	ENSG00000182263	0,787	0,746461811	0,574	0,88	0,36
GABA	TSHZ1	ENSG00000179981	0,764	0,596439849	0,528	0,74	0,22
GABA	PTPRN	ENSG00000054356	0,761	0,594748837	0,522	0,61	0,09
GABA	EDA	ENSG00000158813	0,752	0,614236347	0,504	0,7	0,18
GABA	KALRN	ENSG00000160145	0,814	0,819249533	0,628	0,94	0,42
GABA	UPP2	ENSG00000007001	0,754	0,606012261	0,508	0,69	0,18
GABA	LINC00632	ENSG00000203930	0,778	0,629889782	0,556	0,89	0,38
GABA	UNC79	ENSG00000133958	0,889	1,140979466	0,778	0,95	0,45
GABA	NXPH2	ENSG00000144227	0,753	0,865315701	0,506	0,52	0,01
GABA	HIVEP2	ENSG00000010818	0,823	0,851876827	0,646	0,91	0,41
GABA	KIF9-AS1	ENSG00000227398	0,761	0,589648835	0,522	0,83	0,33
GABA	AC006148.1	ENSG00000242593	0,861	1,101134584	0,722	0,95	0,45
GABA	NWD2	ENSG00000174145	0,749	0,656773041	0,498	0,54	0,04
GABA	DGKI	ENSG00000157680	0,801	0,741108195	0,602	0,9	0,4
GABA	MARCH4	ENSG00000144583	0,751	0,603885558	0,502	0,56	0,07

GABA	GTF2IRD1	ENSG00000006704	0,769	0,620678291	0,538	0,86	0,36
GABA	MYO3B	ENSG00000071909	0,749	0,845458574	0,498	0,53	0,04
GABA	VLDLR-AS1	ENSG00000236404	0,749	0,587326805	0,498	0,76	0,27
GABA	PRKCB	ENSG00000166501	0,773	0,635309121	0,546	0,88	0,39
GABA	STRBP	ENSG00000165209	0,772	0,594135823	0,544	0,85	0,36
GABA	LINC02398	ENSG00000256287	0,748	0,708868355	0,496	0,54	0,05
GABA	MAST4	ENSG00000069020	0,812	0,811562295	0,624	0,93	0,44
GABA	FRAS1	ENSG00000138759	0,748	1,151480708	0,496	0,57	0,08
GABA	HDAC9	ENSG00000048052	0,91	1,327607679	0,82	0,97	0,49
GABA	FRMD4A	ENSG00000151474	0,814	0,643620851	0,628	0,99	0,51
GABA	CHRM3	ENSG00000133019	0,73	0,619414193	0,46	0,55	0,08
GABA	ATP2B4	ENSG00000058668	0,776	0,599114926	0,552	0,9	0,43
GABA	GALNT13	ENSG00000144278	0,913	1,352954278	0,826	0,98	0,51
GABA	NOS1AP	ENSG00000198929	0,771	0,663220221	0,542	0,83	0,36
GABA	AL022068.1	ENSG00000228412	0,737	0,733532917	0,474	0,59	0,12
GABA	ADARB2	ENSG00000185736	0,95	1,675549763	0,9	0,99	0,52
GABA	AC024901.1	ENSG00000255910	0,734	0,668766728	0,468	0,55	0,08
GABA	NSF	ENSG00000073969	0,805	0,769549859	0,61	0,91	0,45
GABA	SLC35F4	ENSG00000151812	0,73	0,783070082	0,46	0,55	0,09
GABA	SCAMP5	ENSG00000198794	0,745	0,59755476	0,49	0,72	0,26
GABA	FOCAD	ENSG00000188352	0,768	0,69818207	0,536	0,85	0,39
GABA	PPP2R1A	ENSG00000105568	0,74	0,600481366	0,48	0,74	0,29
GABA	MARCH11	ENSG00000183654	0,729	0,60507198	0,458	0,51	0,06
GABA	OXR1	ENSG00000164830	0,776	0,596282853	0,552	0,89	0,44
GABA	TRIM9	ENSG00000100505	0,835	0,787543828	0,67	0,96	0,51
GABA	FBN1	ENSG00000166147	0,886	1,168816652	0,772	0,95	0,5
GABA	NDST3	ENSG00000164100	0,723	0,691014488	0,446	0,54	0,1
GABA	CALN1	ENSG00000183166	0,746	0,595943137	0,492	0,89	0,44
GABA	DYNC1I1	ENSG00000158560	0,862	1,087665473	0,724	0,96	0,51
GABA	KIFAP3	ENSG00000075945	0,757	0,60180471	0,514	0,83	0,38
GABA	MYO16	ENSG00000041515	0,727	0,749655607	0,454	0,56	0,12
GABA	EVL	ENSG00000196405	0,777	0,597278974	0,554	0,93	0,49
GABA	MSRA	ENSG00000175806	0,789	0,773542648	0,578	0,95	0,51
GABA	NLGN1	ENSG00000169760	0,789	0,690585776	0,578	0,99	0,55
GABA	LRRC4C	ENSG00000148948	0,894	1,260557152	0,788	0,99	0,55
GABA	AL589740.1	ENSG00000271860	0,812	0,960202049	0,624	0,92	0,49
GABA	DENND5B	ENSG00000170456	0,871	0,998259596	0,742	0,95	0,51
GABA	RORA	ENSG00000069667	0,913	1,332318869	0,826	1	0,57
GABA	GRIA4	ENSG00000152578	0,822	0,749704379	0,644	0,97	0,55
GABA	LARGE1	ENSG00000133424	0,832	0,888709994	0,664	0,93	0,51
GABA	PPFIA2	ENSG00000139220	0,88	1,019878084	0,76	0,99	0,57
GABA	KCNQ5	ENSG00000185760	0,708	0,880730547	0,416	0,51	0,1
GABA	FUT9	ENSG00000172461	0,871	1,062985107	0,742	0,96	0,55
GABA	DANT2	ENSG00000235244	0,748	0,589166739	0,496	0,91	0,49
GABA	NPSR1	ENSG00000187258	0,707	1,375458882	0,414	0,43	0,02
GABA	RASAL2	ENSG00000075391	0,802	0,731362537	0,604	0,93	0,52
GABA	SLC6A1	ENSG00000157103	0,786	0,646625841	0,572	0,89	0,49
GABA	PPP3CA	ENSG00000138814	0,81	0,74918684	0,62	0,94	0,54
GABA	ROBO1	ENSG00000169855	0,884	1,038377227	0,768	0,99	0,59
GABA	R3HDM1	ENSG00000048991	0,901	1,163919068	0,802	0,96	0,57
GABA	MLLT3	ENSG00000171843	0,775	0,623026597	0,55	0,93	0,54
GABA	CAMK2D	ENSG00000145349	0,899	1,087626613	0,798	0,98	0,59
GABA	ARHGAP32	ENSG00000134909	0,764	0,595286095	0,528	0,9	0,52
GABA	NBEA	ENSG00000172915	0,802	0,66810167	0,604	0,97	0,59
GABA	RALGAPA2	ENSG00000188559	0,856	0,947087116	0,712	0,94	0,57

GABA	NRXN3	ENSG00000021645	0,964	1,67079416	0,928	1	0,64
GABA	ASTN2	ENSG00000148219	0,803	0,837522633	0,606	0,97	0,61
GABA	KIZ	ENSG00000088970	0,77	0,617478005	0,54	0,91	0,55
GABA	KIAA1958	ENSG00000165185	0,8	0,701811171	0,6	0,94	0,59
GABA	NAV3	ENSG00000067798	0,766	0,72531132	0,532	0,96	0,61
GABA	SEMA3C	ENSG00000075223	0,705	0,831159115	0,41	0,64	0,3
GABA	KAZN	ENSG00000189337	0,83	0,831142378	0,66	0,99	0,67
GABA	RAP1GDS1	ENSG00000138698	0,775	0,623389465	0,55	0,94	0,64
GABA	FBXL17	ENSG00000145743	0,771	0,613100626	0,542	0,95	0,66
GABA	MAGI1	ENSG00000151276	0,815	0,704380838	0,63	0,97	0,74
GABA	LSAMP	ENSG00000185565	0,902	1,165778821	0,804	1	0,79
GABA	CADM1	ENSG00000182985	0,827	0,7228926	0,654	0,99	0,79
GABA	AUTS2	ENSG00000158321	0,898	1,043751165	0,796	0,99	0,86
DaNs	KCNJ6	ENSG00000157542	0,984	2,771104371	0,968	0,99	0,12
DaNs	KLHL1	ENSG00000150361	0,979	2,858055795	0,958	0,99	0,12
DaNs	TTC6	ENSG00000139865	0,935	1,51035325	0,87	0,92	0,07
DaNs	ANK1	ENSG00000029534	0,946	1,566870154	0,892	0,96	0,11
DaNs	GRIA1	ENSG00000155511	0,93	1,558093247	0,86	0,97	0,14
DaNs	STMN2	ENSG00000104435	0,915	1,047288058	0,83	0,95	0,12
DaNs	SLC18A2	ENSG00000165646	0,916	1,665896591	0,832	0,84	0,01
DaNs	ADGRV1	ENSG00000164199	0,95	1,800011928	0,9	0,96	0,14
DaNs	MIAT	ENSG00000225783	0,927	1,211695932	0,854	0,97	0,15
DaNs	CELF4	ENSG00000101489	0,927	1,398864995	0,854	0,97	0,15
DaNs	ELAVL2	ENSG00000107105	0,931	1,710503052	0,862	0,92	0,1
DaNs	MIR137HG	ENSG00000225206	0,927	1,662534138	0,854	0,93	0,11
DaNs	EBF3	ENSG00000108001	0,912	1,224858206	0,824	0,85	0,03
DaNs	GRIN1	ENSG00000176884	0,892	0,912857895	0,784	0,96	0,14
DaNs	GLRA3	ENSG00000145451	0,914	1,172628813	0,828	0,88	0,07
DaNs	SYT1	ENSG00000067715	0,933	2,038381203	0,866	0,99	0,18
DaNs	SV2B	ENSG00000185518	0,913	1,448516286	0,826	0,88	0,07
DaNs	NRG1	ENSG00000157168	0,911	1,791332481	0,822	0,96	0,16
DaNs	SRRM3	ENSG00000177679	0,904	1,090843804	0,808	0,95	0,15
DaNs	CCDC85A	ENSG00000055813	0,909	1,290616122	0,818	0,96	0,16
DaNs	ATP8A2	ENSG00000132932	0,924	1,598246984	0,848	1	0,21
DaNs	SEZ6L	ENSG00000100095	0,918	1,499497304	0,836	0,97	0,18
DaNs	KCNB2	ENSG00000182674	0,903	1,853858188	0,806	0,89	0,1
DaNs	ST8SIA6	ENSG00000148488	0,903	1,598028578	0,806	0,88	0,09
DaNs	CELF5	ENSG00000161082	0,89	0,829964205	0,78	0,92	0,13
DaNs	SLIT1	ENSG00000187122	0,901	1,039244153	0,802	0,87	0,08
DaNs	ZNF385D	ENSG00000151789	0,896	1,1291027	0,792	0,93	0,15
DaNs	TH	ENSG00000180176	0,89	1,360356782	0,78	0,78	0
DaNs	HECW1	ENSG00000002746	0,893	1,232786039	0,786	0,95	0,17
DaNs	CLSTN2	ENSG00000158258	0,917	1,962029507	0,834	0,92	0,14
DaNs	KIAA0319	ENSG00000137261	0,915	1,204056152	0,83	0,93	0,16
DaNs	DNM1	ENSG00000106976	0,885	0,832301033	0,77	0,96	0,18
DaNs	CUX2	ENSG00000111249	0,901	1,67922719	0,802	0,87	0,09
DaNs	NMNAT2	ENSG00000157064	0,904	1,101448859	0,808	0,95	0,17
DaNs	ANKRD30BL	ENSG00000163046	0,911	1,367102117	0,822	0,95	0,18
DaNs	AL445623.2	ENSG00000283982	0,894	1,281911644	0,788	0,84	0,07
DaNs	PCDH15	ENSG00000150275	0,91	1,744737984	0,82	0,97	0,2
DaNs	GRIN2B	ENSG00000273079	0,889	1,240842176	0,778	0,93	0,16
DaNs	RAB3C	ENSG00000152932	0,907	1,51746591	0,814	0,93	0,16
DaNs	MYT1L	ENSG00000186487	0,89	1,357394692	0,78	0,95	0,18
DaNs	DOK6	ENSG00000206052	0,906	1,929995615	0,812	0,95	0,18
DaNs	EPHA5	ENSG00000145242	0,898	1,62794238	0,796	0,88	0,11

DaNs	SCN9A	ENSG00000169432	0,899	1,204124484	0,798	0,92	0,16
DaNs	ADAM23	ENSG00000114948	0,914	1,194575934	0,828	0,99	0,23
DaNs	C11orf80	ENSG00000173715	0,886	0,854754762	0,772	0,93	0,17
DaNs	KCNT2	ENSG00000162687	0,885	1,152450349	0,77	0,91	0,15
DaNs	PAK3	ENSG00000077264	0,892	1,271005043	0,784	0,92	0,16
DaNs	GALNTL6	ENSG00000174473	0,923	3,080935992	0,846	0,91	0,15
DaNs	NYAP2	ENSG00000144460	0,884	1,255026881	0,768	0,85	0,1
DaNs	AMPH	ENSG00000078053	0,877	0,964846588	0,754	0,92	0,16
DaNs	RET	ENSG00000165731	0,882	0,777366532	0,764	0,78	0,03
DaNs	TMEM132B	ENSG00000139364	0,904	1,586101009	0,808	0,93	0,18
DaNs	CADPS	ENSG00000163618	0,953	1,917357794	0,906	1	0,25
DaNs	CACNA1B	ENSG00000148408	0,901	1,667704908	0,802	0,93	0,18
DaNs	MEG8	ENSG00000225746	0,897	1,433918462	0,794	0,92	0,17
DaNs	ROBO2	ENSG00000185008	0,941	2,465836256	0,882	0,97	0,23
DaNs	CAMK2B	ENSG00000058404	0,854	0,678868316	0,708	0,93	0,18
DaNs	AC007091.1	ENSG00000223838	0,875	1,235258506	0,75	0,77	0,02
DaNs	CHSY3	ENSG00000198108	0,91	1,85912369	0,82	0,93	0,19
DaNs	SNAP25	ENSG00000132639	0,902	1,208123276	0,804	0,99	0,24
DaNs	AC120193.1	ENSG00000253535	0,901	1,706458073	0,802	0,88	0,13
DaNs	LINGO2	ENSG00000174482	0,861	1,243643568	0,722	0,88	0,14
DaNs	OLFM3	ENSG00000118733	0,87	1,075263285	0,74	0,89	0,15
DaNs	ELAVL4	ENSG00000162374	0,882	0,975031429	0,764	0,88	0,14
DaNs	RIMS2	ENSG00000176406	0,897	1,475246731	0,794	0,96	0,22
DaNs	FGF12	ENSG00000114279	0,906	1,728378408	0,812	0,97	0,24
DaNs	REEP1	ENSG00000068615	0,87	0,943788377	0,74	0,88	0,14
DaNs	DRD2	ENSG00000149295	0,871	1,05460594	0,742	0,76	0,02
DaNs	TOX2	ENSG00000124191	0,861	0,756591811	0,722	0,87	0,13
DaNs	GABRB1	ENSG00000163288	0,937	1,649110789	0,874	1	0,27
DaNs	SNTG1	ENSG00000147481	0,907	2,001213945	0,814	0,93	0,2
DaNs	RIMBP2	ENSG00000060709	0,889	1,232047727	0,778	0,89	0,16
DaNs	BTBD11	ENSG00000151136	0,876	1,286034884	0,752	0,87	0,14
DaNs	AC073050.1	ENSG00000228222	0,875	1,672393794	0,75	0,92	0,19
DaNs	SCN3A	ENSG00000153253	0,851	0,843510408	0,702	0,91	0,18
DaNs	MAST1	ENSG00000105613	0,86	0,698141517	0,72	0,85	0,12
DaNs	SORBS2	ENSG00000154556	0,858	0,884734462	0,716	0,97	0,25
DaNs	FRMPD4	ENSG00000169933	0,855	1,064189895	0,71	0,91	0,18
DaNs	KCND3	ENSG00000171385	0,872	1,103262367	0,744	0,93	0,21
DaNs	BICDL1	ENSG00000135127	0,868	0,90087443	0,736	0,87	0,14
DaNs	KLHL29	ENSG00000119771	0,881	1,323136934	0,762	0,88	0,16
DaNs	WNK2	ENSG00000165238	0,855	0,786337874	0,71	0,91	0,19
DaNs	RALYL	ENSG00000184672	0,915	1,651309355	0,83	0,99	0,27
DaNs	DCLK1	ENSG00000133083	0,886	1,416713105	0,772	0,97	0,25
DaNs	CRMP1	ENSG00000072832	0,853	0,600897121	0,706	0,85	0,13
DaNs	SPTBN4	ENSG00000160460	0,895	1,171571825	0,79	0,97	0,26
DaNs	PTPRO	ENSG00000151490	0,871	1,68485314	0,742	0,85	0,13
DaNs	IQSEC3	ENSG00000120645	0,863	0,798680362	0,726	0,82	0,11
DaNs	SLC4A10	ENSG00000144290	0,846	0,892481518	0,692	0,88	0,16
DaNs	CACNA1E	ENSG00000198216	0,842	0,698411132	0,684	0,88	0,16
DaNs	GRIP1	ENSG00000155974	0,903	1,544158913	0,806	0,96	0,24
DaNs	PRICKLE1	ENSG00000139174	0,854	0,970103074	0,708	0,85	0,14
DaNs	UNC13C	ENSG00000137766	0,856	2,012335496	0,712	0,81	0,1
DaNs	EPHA6	ENSG00000080224	0,87	1,686949984	0,74	0,85	0,14
DaNs	UNC80	ENSG00000144406	0,853	0,869394575	0,706	0,95	0,23
DaNs	B4GALT6	ENSG00000118276	0,853	0,730467837	0,706	0,84	0,13
DaNs	GABRG2	ENSG00000113327	0,848	0,702032848	0,696	0,82	0,11

DaNs	PRKG1	ENSG00000185532	0,879	1,385967314	0,758	0,97	0,26
DaNs	PGM2L1	ENSG00000165434	0,853	0,703575569	0,706	0,84	0,13
DaNs	SLC35F1	ENSG00000196376	0,867	1,068150055	0,734	0,95	0,24
DaNs	INPP4B	ENSG00000109452	0,907	1,920887302	0,814	1	0,3
DaNs	SLC44A5	ENSG00000137968	0,875	1,620813001	0,75	0,87	0,16
DaNs	MIR2052HG	ENSG00000254349	0,869	1,20760234	0,738	0,88	0,18
DaNs	XKR4	ENSG00000206579	0,851	1,005912065	0,702	0,89	0,19
DaNs	ARHGEF28	ENSG00000214944	0,848	0,720262513	0,696	0,84	0,14
DaNs	EML6	ENSG00000214595	0,868	1,010085564	0,736	0,95	0,25
DaNs	OSBPL10	ENSG00000144645	0,863	1,046949046	0,726	0,8	0,1
DaNs	SCN2A	ENSG00000136531	0,849	0,942927833	0,698	0,91	0,21
DaNs	LMX1B	ENSG00000136944	0,85	0,742152179	0,7	0,72	0,02
DaNs	PRR16	ENSG00000184838	0,867	1,658253999	0,734	0,81	0,11
DaNs	GABBR2	ENSG00000136928	0,844	0,956204686	0,688	0,84	0,14
DaNs	PTPRN2	ENSG00000155093	0,895	1,425941436	0,79	0,93	0,24
DaNs	GAP43	ENSG00000172020	0,852	0,892588927	0,704	0,82	0,13
DaNs	SYT14	ENSG00000143469	0,861	0,845373375	0,722	0,99	0,29
DaNs	HSPA4L	ENSG00000164070	0,852	0,812136132	0,704	0,91	0,21
DaNs	MTUS2	ENSG00000132938	0,845	1,152007129	0,69	0,82	0,13
DaNs	JAKMIP1	ENSG00000152969	0,856	1,065759831	0,712	0,81	0,12
DaNs	GABRB3	ENSG00000166206	0,85	1,067647018	0,7	0,89	0,2
DaNs	RNF150	ENSG00000170153	0,858	1,13174591	0,716	0,95	0,25
DaNs	KCNIP4	ENSG00000185774	0,893	1,484625756	0,786	0,99	0,29
DaNs	STXBP5-AS1	ENSG00000233452	0,85	1,013409342	0,7	0,87	0,17
DaNs	VWC2L	ENSG00000174453	0,845	1,010153699	0,69	0,77	0,08
DaNs	GPC6	ENSG00000183098	0,895	2,151896855	0,79	0,91	0,22
DaNs	GPRIN3	ENSG00000185477	0,827	0,672297519	0,654	0,85	0,16
DaNs	CACNA1A	ENSG00000141837	0,868	1,222818632	0,736	0,97	0,29
DaNs	SCG2	ENSG00000171951	0,858	1,123992558	0,716	0,8	0,11
DaNs	ST6GALNAC5	ENSG00000117069	0,847	1,531780677	0,694	0,76	0,07
DaNs	MEG3	ENSG00000214548	0,848	0,721916938	0,696	0,78	0,1
DaNs	SHANK2	ENSG00000162105	0,849	1,162604021	0,698	0,87	0,18
DaNs	SLC2A13	ENSG00000151229	0,874	1,550862993	0,748	0,93	0,25
DaNs	KCNQ3	ENSG00000184156	0,88	1,218153204	0,76	0,97	0,29
DaNs	LRRC7	ENSG00000033122	0,825	0,760011767	0,65	1	0,32
DaNs	SLC8A1	ENSG00000183023	0,85	1,128561898	0,7	0,99	0,31
DaNs	KSR2	ENSG00000171435	0,852	1,102830872	0,704	0,87	0,19
DaNs	LRFN5	ENSG00000165379	0,825	0,916875232	0,65	0,88	0,2
DaNs	SRRM4	ENSG00000139767	0,828	0,651324741	0,656	0,78	0,11
DaNs	NSG2	ENSG00000170091	0,827	0,742520899	0,654	0,82	0,15
DaNs	CCSER1	ENSG00000184305	0,914	1,629477831	0,828	0,97	0,3
DaNs	SCN7A	ENSG00000136546	0,83	0,61310342	0,66	0,77	0,1
DaNs	ARHGAP44	ENSG00000006740	0,859	0,993344928	0,718	0,88	0,2
DaNs	TENM3	ENSG00000218336	0,829	0,79758148	0,658	0,89	0,22
DaNs	FRRS1L	ENSG00000260230	0,837	0,715652319	0,674	0,87	0,19
DaNs	SEMA6D	ENSG00000137872	0,938	1,883607961	0,876	0,96	0,29
DaNs	PPM1E	ENSG00000175175	0,891	1,353958387	0,782	0,96	0,29
DaNs	ADD2	ENSG00000075340	0,824	0,683472328	0,648	0,8	0,13
DaNs	DLGAP2	ENSG00000198010	0,834	1,07649953	0,668	0,82	0,15
DaNs	GNAL	ENSG00000141404	0,822	0,706076027	0,644	0,82	0,15
DaNs	KIAA1211	ENSG00000109265	0,823	0,732494248	0,646	0,91	0,24
DaNs	SLITRK5	ENSG00000165300	0,83	0,641415881	0,66	0,82	0,15
DaNs	SHISA9	ENSG00000237515	0,81	0,713551764	0,62	0,85	0,18
DaNs	BASP1	ENSG00000176788	0,817	0,640713258	0,634	0,89	0,22
DaNs	ABLIM2	ENSG00000163995	0,851	0,866429879	0,702	0,88	0,21

DaNs	SYT16	ENSG00000139973	0,828	0,788884101	0,656	0,84	0,17
DaNs	PCDH7	ENSG00000169851	0,869	1,29847983	0,738	0,96	0,29
DaNs	UCHL1	ENSG00000154277	0,871	0,888589733	0,742	0,96	0,29
DaNs	SCN8A	ENSG00000196876	0,851	0,959322918	0,702	0,89	0,23
DaNs	DLGAP1	ENSG00000170579	0,833	0,797915923	0,666	0,96	0,3
DaNs	MYRIP	ENSG00000170011	0,907	1,443324607	0,814	0,96	0,3
DaNs	RAP1GAP2	ENSG00000132359	0,832	0,871659909	0,664	0,84	0,18
DaNs	EPB41	ENSG00000159023	0,808	0,588163749	0,616	0,88	0,22
DaNs	LINC02389	ENSG00000255693	0,828	0,789464665	0,656	0,76	0,1
DaNs	ATP2B2	ENSG00000157087	0,806	0,607009546	0,612	0,89	0,23
DaNs	SYBU	ENSG00000147642	0,818	0,682740789	0,636	0,85	0,19
DaNs	NDRG4	ENSG00000103034	0,814	0,588448657	0,628	0,81	0,15
DaNs	RUNDC3B	ENSG00000105784	0,82	0,698308801	0,64	0,82	0,17
DaNs	RERG	ENSG00000134533	0,824	0,946497074	0,648	0,76	0,1
DaNs	FAM155A	ENSG00000204442	0,925	1,616422039	0,85	1	0,34
DaNs	RIMS1	ENSG00000079841	0,878	1,400431217	0,756	0,95	0,29
DaNs	PID1	ENSG00000153823	0,854	1,358558733	0,708	0,89	0,24
DaNs	KIF26B	ENSG00000162849	0,832	0,980266303	0,664	0,88	0,22
DaNs	CALY	ENSG00000130643	0,818	0,6673734	0,636	0,78	0,13
DaNs	DNAH14	ENSG00000185842	0,831	0,847290114	0,662	0,82	0,17
DaNs	SLC8A3	ENSG00000100678	0,816	0,643909329	0,632	0,81	0,16
DaNs	FOXP2	ENSG00000128573	0,839	1,267497713	0,678	0,84	0,19
DaNs	PEG10	ENSG00000242265	0,847	1,150751595	0,694	0,81	0,16
DaNs	DSCAM	ENSG00000171587	0,852	0,972294583	0,704	0,97	0,32
DaNs	MCC	ENSG00000171444	0,825	1,088258699	0,65	0,88	0,23
DaNs	KHDRBS2	ENSG00000112232	0,806	0,650369836	0,612	0,91	0,25
DaNs	CADPS2	ENSG00000081803	0,882	2,009136908	0,764	0,87	0,22
DaNs	PIP5K1B	ENSG00000107242	0,829	0,891753269	0,658	0,81	0,16
DaNs	PLXNA4	ENSG00000221866	0,826	0,901814505	0,652	0,81	0,16
DaNs	CNNM1	ENSG00000119946	0,822	0,611453039	0,644	0,76	0,11
DaNs	SDC2	ENSG00000169439	0,827	0,925071963	0,654	0,76	0,11
DaNs	PAM	ENSG00000145730	0,847	1,12936238	0,694	0,93	0,29
DaNs	AGBL4	ENSG00000186094	0,838	1,038281538	0,676	0,92	0,27
DaNs	FAM189A1	ENSG00000104059	0,819	0,704198799	0,638	0,77	0,12
DaNs	CSMD2	ENSG00000121904	0,854	1,250258683	0,708	0,92	0,27
DaNs	EFNA5	ENSG00000184349	0,835	1,282367134	0,67	0,81	0,17
DaNs	KIAA1549L	ENSG00000110427	0,814	0,742904454	0,628	0,8	0,15
DaNs	LMTK3	ENSG00000142235	0,819	0,645609255	0,638	0,87	0,22
DaNs	PLA2R1	ENSG00000153246	0,823	0,827750448	0,646	0,74	0,1
DaNs	GRIN3A	ENSG00000198785	0,824	0,860284607	0,648	0,72	0,08
DaNs	SVOP	ENSG00000166111	0,821	0,622714711	0,642	0,73	0,09
DaNs	AMMECR1	ENSG00000101935	0,846	1,145124449	0,692	0,8	0,16
DaNs	LMX1A	ENSG00000162761	0,819	0,976499459	0,638	0,65	0,01
DaNs	PLCB4	ENSG00000101333	0,812	0,803340858	0,624	0,89	0,25
DaNs	GRIK2	ENSG00000164418	0,804	0,678332838	0,608	0,95	0,31
DaNs	DGKH	ENSG00000102780	0,81	0,716554387	0,62	0,84	0,2
DaNs	RAB27B	ENSG00000041353	0,817	0,743104703	0,634	0,73	0,1
DaNs	CPLX2	ENSG00000145920	0,815	0,594112422	0,63	0,72	0,08
DaNs	UBASH3B	ENSG00000154127	0,812	0,624185151	0,624	0,78	0,15
DaNs	SNRPN	ENSG00000128739	0,855	1,293582977	0,71	0,91	0,27
DaNs	STXBP5L	ENSG00000145087	0,828	1,005364762	0,656	0,88	0,25
DaNs	ADAMTS2	ENSG00000087116	0,814	0,714754162	0,628	0,69	0,06
DaNs	TENM2	ENSG00000145934	0,827	1,565636885	0,654	0,84	0,21
DaNs	SLC41A2	ENSG00000136052	0,806	0,605275396	0,612	0,8	0,17
DaNs	DGKB	ENSG00000136267	0,795	0,711481884	0,59	0,85	0,22

DaNs	KIF5A	ENSG00000155980	0,815	0,610862767	0,63	0,77	0,14
DaNs	MGAT4C	ENSG00000182050	0,814	0,929893928	0,628	0,84	0,21
DaNs	LINC01414	ENSG00000253554	0,809	0,742829482	0,618	0,74	0,11
DaNs	DCX	ENSG00000077279	0,814	0,598048916	0,628	0,72	0,09
DaNs	LRRTM4	ENSG00000176204	0,821	0,950410994	0,642	0,93	0,31
DaNs	CNTN1	ENSG00000018236	0,861	1,260486471	0,722	0,97	0,35
DaNs	CNKSR2	ENSG00000149970	0,804	0,740771685	0,608	0,82	0,2
DaNs	CHL1	ENSG00000134121	0,797	0,669147685	0,594	0,89	0,27
DaNs	KIF9-AS1	ENSG00000227398	0,83	0,696732618	0,66	0,96	0,34
DaNs	STXBP1	ENSG00000136854	0,822	0,714320998	0,644	0,93	0,31
DaNs	RPH3A	ENSG00000089169	0,805	0,790145174	0,61	0,73	0,11
DaNs	WDR17	ENSG00000150627	0,833	0,745025434	0,666	0,91	0,29
DaNs	AC092683.1	ENSG00000230606	0,832	0,756749929	0,664	0,96	0,34
DaNs	FGF13	ENSG00000129682	0,803	0,59485551	0,606	0,72	0,1
DaNs	MYH10	ENSG00000133026	0,795	0,645017266	0,59	0,82	0,21
DaNs	AC025159.1	ENSG00000257815	0,851	0,93202621	0,702	0,93	0,32
DaNs	SHISA6	ENSG00000188803	0,821	1,385350857	0,642	0,76	0,15
DaNs	CERS6	ENSG00000172292	0,821	0,833744555	0,642	0,91	0,29
DaNs	CACNA1D	ENSG00000157388	0,827	0,83202211	0,654	0,91	0,3
DaNs	RGS6	ENSG00000182732	0,828	1,530262441	0,656	0,81	0,2
DaNs	RBMS1	ENSG00000153250	0,803	0,691712212	0,606	0,91	0,3
DaNs	ERC2	ENSG00000187672	0,879	1,385498872	0,758	0,97	0,37
DaNs	FAM135B	ENSG00000147724	0,788	0,752514064	0,576	0,85	0,24
DaNs	GABRA4	ENSG00000109158	0,803	0,599580537	0,606	0,66	0,06
DaNs	PKNOX2	ENSG00000165495	0,798	0,626069649	0,596	0,77	0,16
DaNs	SLIT2	ENSG00000145147	0,824	1,139033346	0,648	0,81	0,21
DaNs	CAP2	ENSG00000112186	0,803	0,678237146	0,606	0,74	0,14
DaNs	TMEM255A	ENSG00000125355	0,802	0,715829669	0,604	0,68	0,07
DaNs	TRPC6	ENSG00000137672	0,806	1,139566268	0,612	0,65	0,05
DaNs	PRKAR2B	ENSG00000005249	0,805	0,633905783	0,61	0,74	0,14
DaNs	SPTAN1	ENSG00000197694	0,801	0,785461872	0,602	0,93	0,33
DaNs	VSNL1	ENSG00000163032	0,788	0,681907218	0,576	0,72	0,11
DaNs	ANKRD29	ENSG00000154065	0,801	0,665658288	0,602	0,69	0,09
DaNs	FBXO16	ENSG00000214050	0,798	0,613261658	0,596	0,73	0,13
DaNs	ADGRL2	ENSG00000117114	0,812	1,299549671	0,624	0,76	0,16
DaNs	FP700111.1	ENSG00000224363	0,79	0,615787832	0,58	0,88	0,28
DaNs	CSMD1	ENSG00000183117	0,923	1,779680559	0,846	1	0,4
DaNs	TMEM178B	ENSG00000261115	0,887	1,213787511	0,774	0,99	0,39
DaNs	CNTNAP3B	ENSG00000154529	0,795	0,668362454	0,59	0,8	0,2
DaNs	CNTN4	ENSG00000144619	0,791	0,897558323	0,582	0,78	0,19
DaNs	DAB1	ENSG00000173406	0,892	1,642859848	0,784	0,99	0,39
DaNs	CEP126	ENSG00000110318	0,801	0,601850533	0,602	0,88	0,28
DaNs	SYN2	ENSG00000157152	0,779	0,59040119	0,558	0,76	0,16
DaNs	SDK1	ENSG00000146555	0,808	1,006711809	0,616	0,87	0,27
DaNs	CACNG2	ENSG00000166862	0,793	0,616214279	0,586	0,72	0,12
DaNs	PRICKLE2	ENSG00000163637	0,825	0,882908199	0,65	0,91	0,31
DaNs	SLC6A3	ENSG00000142319	0,796	1,262554146	0,592	0,6	0
DaNs	CSMD3	ENSG00000164796	0,816	0,951944498	0,632	0,95	0,36
DaNs	PCLO	ENSG00000186472	0,87	1,10661562	0,74	0,96	0,37
DaNs	LINC01876	ENSG00000226383	0,796	1,225363121	0,592	0,62	0,04
DaNs	ANKRD34C-AS1	ENSG00000259234	0,787	0,625168342	0,574	0,68	0,09
DaNs	SV2C	ENSG00000122012	0,804	1,617055077	0,608	0,65	0,06
DaNs	PPP2R2C	ENSG00000074211	0,799	0,63738079	0,598	0,96	0,37
DaNs	GRIN2A	ENSG00000183454	0,806	1,331535571	0,612	0,74	0,16

DaNs	FNBP1L	ENSG00000137942	0,776	0,60049794	0,552	0,82	0,24
DaNs	LINC01250	ENSG00000234423	0,79	0,651523801	0,58	0,68	0,1
DaNs	EML5	ENSG00000165521	0,789	0,662516574	0,578	0,8	0,22
DaNs	GRIA3	ENSG00000125675	0,862	1,043525073	0,724	0,93	0,35
DaNs	CLVS1	ENSG00000177182	0,783	0,601981978	0,566	0,77	0,19
DaNs	PLCB1	ENSG00000182621	0,809	0,993094325	0,618	0,93	0,35
DaNs	GFRA1	ENSG00000151892	0,797	1,411927978	0,594	0,65	0,07
DaNs	FHOD3	ENSG00000134775	0,774	0,603659678	0,548	0,74	0,16
DaNs	PCSK2	ENSG00000125851	0,786	0,723189831	0,572	0,7	0,13
DaNs	LINC00632	ENSG00000203930	0,839	0,780204268	0,678	0,96	0,38
DaNs	SGSM1	ENSG00000167037	0,813	0,704595154	0,626	0,89	0,32
DaNs	DAAM1	ENSG00000100592	0,796	0,593579826	0,592	0,91	0,33
DaNs	GRM8	ENSG00000179603	0,789	1,121700753	0,578	0,7	0,13
DaNs	HSPA12A	ENSG00000165868	0,844	0,778157028	0,688	0,97	0,4
DaNs	TENM1	ENSG00000009694	0,79	0,894527276	0,58	0,73	0,15
DaNs	TUSC3	ENSG00000104723	0,782	0,590387624	0,564	0,73	0,15
DaNs	AC092691.1	ENSG00000239268	0,809	0,96578097	0,618	0,95	0,37
DaNs	FBXL2	ENSG00000153558	0,797	0,671326784	0,594	0,84	0,26
DaNs	FGF14	ENSG00000102466	0,81	1,139988751	0,62	0,87	0,29
DaNs	RSPO2	ENSG00000147655	0,788	1,15088128	0,576	0,61	0,04
DaNs	ARHGAP6	ENSG00000047648	0,775	0,647723939	0,55	0,74	0,17
DaNs	RAPGEF4	ENSG00000091428	0,773	0,733689381	0,546	0,92	0,35
DaNs	ME1	ENSG00000065833	0,797	0,638328157	0,594	0,88	0,31
DaNs	CNTNAP5	ENSG00000155052	0,766	0,713998699	0,532	0,77	0,2
DaNs	BNC2	ENSG00000173068	0,778	0,891698968	0,556	0,73	0,16
DaNs	FLRT2	ENSG00000185070	0,771	0,634463374	0,542	0,78	0,22
DaNs	UBA6-AS1	ENSG00000248049	0,852	1,108161759	0,704	0,97	0,41
DaNs	CDH4	ENSG00000179242	0,804	1,061303198	0,608	0,8	0,23
DaNs	PLEKHA5	ENSG00000052126	0,867	1,185637213	0,734	1	0,44
DaNs	SPAG16	ENSG00000144451	0,774	0,623193672	0,548	0,91	0,34
DaNs	MAP2	ENSG00000078018	0,897	1,289059443	0,794	0,97	0,41
DaNs	KCNH7	ENSG00000184611	0,786	1,077519189	0,572	0,69	0,13
DaNs	NPNT	ENSG00000168743	0,782	0,898523508	0,564	0,62	0,06
DaNs	AC024901.1	ENSG00000255910	0,781	0,812051321	0,562	0,65	0,09
DaNs	AFF3	ENSG00000144218	0,856	1,241022613	0,712	0,95	0,38
DaNs	HS6ST3	ENSG00000185352	0,775	1,016353674	0,55	0,77	0,21
DaNs	FRY	ENSG00000073910	0,765	0,745253497	0,53	0,92	0,36
DaNs	NOL4	ENSG00000101746	0,779	0,903241687	0,558	0,78	0,22
DaNs	PLCXD3	ENSG00000182836	0,781	0,922197864	0,562	0,68	0,12
DaNs	LARP1B	ENSG00000138709	0,769	0,621661892	0,538	0,92	0,36
DaNs	AC008591.1	ENSG00000251680	0,783	1,164105598	0,566	0,61	0,05
DaNs	ASXL3	ENSG00000141431	0,768	0,613829474	0,536	0,81	0,26
DaNs	TMEM108	ENSG00000144868	0,756	0,600298054	0,512	0,84	0,28
DaNs	PPIP5K2	ENSG00000145725	0,811	0,663111521	0,622	0,92	0,37
DaNs	SNAP91	ENSG00000065609	0,791	0,706960103	0,582	0,89	0,34
DaNs	TRPC5	ENSG00000072315	0,785	1,154301705	0,57	0,65	0,1
DaNs	CACNG3	ENSG00000006116	0,779	0,688203954	0,558	0,6	0,04
DaNs	AC096711.2	ENSG00000251555	0,82	0,705614137	0,64	0,92	0,37
DaNs	CSRNP3	ENSG00000178662	0,791	0,793898853	0,582	0,95	0,4
DaNs	MAGI3	ENSG00000081026	0,777	0,724590628	0,554	0,88	0,33
DaNs	NEXMIF	ENSG00000050030	0,769	0,687361534	0,538	0,7	0,15
DaNs	EPB41L4B	ENSG00000095203	0,775	0,605956499	0,55	0,69	0,14
DaNs	MTCL1	ENSG00000168502	0,772	0,769889209	0,544	0,76	0,21
DaNs	NRXN1	ENSG00000179915	0,82	1,010984375	0,64	0,99	0,44
DaNs	FSTL4	ENSG00000053108	0,781	1,253062732	0,562	0,64	0,1

DaNs	NTN1	ENSG00000065320	0,768	0,826013722	0,536	0,65	0,11
DaNs	MPPED2	ENSG00000066382	0,757	0,630847775	0,514	0,74	0,2
DaNs	TBC1D19	ENSG00000109680	0,802	0,76635956	0,604	0,96	0,42
DaNs	NELL2	ENSG00000184613	0,765	0,724634353	0,53	0,69	0,15
DaNs	CACNA1C	ENSG00000151067	0,793	0,758218625	0,586	0,89	0,36
DaNs	MIR4500HG	ENSG00000228824	0,769	0,835369001	0,538	0,58	0,05
DaNs	SLC35F4	ENSG00000151812	0,765	0,942761115	0,53	0,62	0,09
DaNs	HCN1	ENSG00000164588	0,759	0,631511268	0,518	0,64	0,1
DaNs	GPR158	ENSG00000151025	0,755	0,681937303	0,51	0,85	0,32
DaNs	AJAP1	ENSG00000196581	0,766	0,620368918	0,532	0,74	0,21
DaNs	ZFHX3	ENSG00000140836	0,815	1,181505683	0,63	0,96	0,43
DaNs	CDH18	ENSG00000145526	0,768	1,163151253	0,536	0,72	0,19
DaNs	RIT2	ENSG00000152214	0,766	0,838298261	0,532	0,65	0,12
DaNs	MCTP1	ENSG00000175471	0,758	0,632368088	0,516	0,7	0,18
DaNs	CDH10	ENSG00000040731	0,754	0,629404768	0,508	0,72	0,19
DaNs	FOCAD	ENSG00000188352	0,812	0,793063968	0,624	0,92	0,4
DaNs	CDK14	ENSG00000058091	0,805	0,935113215	0,61	0,93	0,41
DaNs	ZNF804A	ENSG00000170396	0,745	0,672002643	0,49	0,68	0,15
DaNs	GSG1L	ENSG00000169181	0,758	0,638938351	0,516	0,65	0,13
DaNs	TMEM232	ENSG00000186952	0,756	0,632851406	0,512	0,85	0,33
DaNs	MARCH4	ENSG00000144583	0,762	0,606069977	0,524	0,6	0,07
DaNs	AC090578.1	ENSG00000253553	0,756	0,607791043	0,512	0,64	0,11
DaNs	KCNH5	ENSG00000140015	0,758	0,725573892	0,516	0,61	0,09
DaNs	NSF	ENSG00000073969	0,8	0,740208914	0,6	0,97	0,46
DaNs	CTNNA2	ENSG00000066032	0,763	0,625049744	0,526	0,99	0,47
DaNs	ANAPC10	ENSG00000164162	0,737	0,593153015	0,474	0,88	0,36
DaNs	CDH8	ENSG00000150394	0,753	0,887677497	0,506	0,84	0,32
DaNs	GRM5	ENSG00000168959	0,733	0,742365981	0,466	0,73	0,22
DaNs	EBF1	ENSG00000164330	0,751	0,797821781	0,502	0,66	0,15
DaNs	LUZP2	ENSG00000187398	0,738	0,742092532	0,476	0,77	0,26
DaNs	GPC5	ENSG00000179399	0,762	1,097840493	0,524	0,8	0,29
DaNs	MIPOL1	ENSG00000151338	0,76	0,659368541	0,52	0,82	0,32
DaNs	DCC	ENSG00000187323	0,79	1,775809387	0,58	0,76	0,26
DaNs	CACNB4	ENSG00000182389	0,75	0,728600388	0,5	0,62	0,12
DaNs	APBA1	ENSG00000107282	0,806	0,814823635	0,612	0,92	0,42
DaNs	CACNA2D3	ENSG00000157445	0,758	0,690366413	0,516	0,8	0,3
DaNs	IQCJ-SCHIP1	ENSG00000283154	0,94	2,108105232	0,88	1	0,5
DaNs	XKR6	ENSG00000171044	0,809	0,975997368	0,618	0,88	0,38
DaNs	CDH12	ENSG00000154162	0,746	1,054422692	0,492	0,69	0,19
DaNs	MCF2L2	ENSG00000053524	0,774	0,636180447	0,548	0,88	0,38
DaNs	HS6ST2	ENSG00000171004	0,751	0,814583329	0,502	0,6	0,1
DaNs	RGS7	ENSG00000182901	0,778	0,7800691	0,556	0,92	0,43
DaNs	PWRN1	ENSG00000259905	0,743	0,681748771	0,486	0,77	0,28
DaNs	NEDD4L	ENSG00000049759	0,723	0,599164296	0,446	0,91	0,42
DaNs	FRMD4A	ENSG00000151474	0,882	1,322370089	0,764	1	0,52
DaNs	SGCD	ENSG00000170624	0,902	2,000750288	0,804	0,96	0,48
DaNs	CNTN6	ENSG00000134115	0,734	0,71543005	0,468	0,58	0,1
DaNs	LINC00535	ENSG00000246662	0,738	0,631922882	0,476	0,58	0,1
DaNs	LINC01322	ENSG00000244128	0,736	0,617903283	0,472	0,65	0,17
DaNs	DYNC1I1	ENSG00000158560	0,83	1,023774422	0,66	0,99	0,52
DaNs	ZNF385B	ENSG00000144331	0,721	0,593009581	0,442	0,58	0,12
DaNs	UNC5D	ENSG00000156687	0,727	0,893914827	0,454	0,61	0,15
DaNs	MACROD2	ENSG00000172264	0,819	1,008889209	0,638	1	0,54
DaNs	UNC79	ENSG00000133958	0,76	0,630301902	0,52	0,91	0,45
DaNs	DENND5B	ENSG00000170456	0,817	0,73606362	0,634	0,96	0,52

DaNs	GDAP1	ENSG00000104381	0,803	0,812026449	0,606	0,95	0,51
DaNs	NLGN1	ENSG00000169760	0,785	0,753292243	0,57	0,99	0,56
DaNs	KCNAB1	ENSG00000169282	0,711	0,587018497	0,422	0,65	0,23
DaNs	AC110023.1	ENSG00000258631	0,714	0,85164163	0,428	0,51	0,09
DaNs	PACS1	ENSG00000175115	0,732	0,641774828	0,464	0,85	0,43
DaNs	AEBP2	ENSG00000139154	0,736	0,616646584	0,472	0,89	0,48
DaNs	CNTNAP2	ENSG00000174469	0,888	1,407147392	0,776	1	0,59
DaNs	PPFIA2	ENSG00000139220	0,818	1,188265231	0,636	0,99	0,57
DaNs	KCNC2	ENSG00000166006	0,702	0,634367481	0,404	0,51	0,1
DaNs	GABRG3	ENSG00000182256	0,701	0,777904688	0,402	0,51	0,1
DaNs	SORCS3	ENSG00000156395	0,702	0,720069837	0,404	0,57	0,16
DaNs	TOX3	ENSG00000103460	0,713	0,644170737	0,426	0,65	0,24
DaNs	PAPPA	ENSG00000182752	0,706	0,624749272	0,412	0,45	0,04
DaNs	SAMD3	ENSG00000164483	0,708	0,861698327	0,416	0,46	0,05
DaNs	FUT9	ENSG00000172461	0,773	0,735281675	0,546	0,96	0,55
DaNs	PPP3CA	ENSG00000138814	0,78	0,706015091	0,56	0,95	0,55
DaNs	CPEB3	ENSG00000107864	0,748	0,620375132	0,496	0,96	0,58
DaNs	ADARB2	ENSG00000185736	0,841	1,354470447	0,682	0,91	0,53
DaNs	NBEA	ENSG00000172915	0,802	0,997220246	0,604	0,97	0,6
DaNs	PBX1	ENSG00000185630	0,944	1,741966297	0,888	0,99	0,62
DaNs	PBX3	ENSG00000167081	0,745	0,710847109	0,49	0,87	0,5
DaNs	AC124312.1	ENSG00000214265	0,787	0,720682119	0,574	0,97	0,61
DaNs	SMYD3	ENSG00000185420	0,764	0,716942337	0,528	0,97	0,62
DaNs	NEGR1	ENSG00000172260	0,81	1,168034816	0,62	0,99	0,63
DaNs	GRID2	ENSG00000152208	0,708	0,797802324	0,416	0,87	0,52
DaNs	RASGEF1B	ENSG00000138670	0,73	0,703904302	0,46	0,95	0,61
DaNs	NRXN3	ENSG00000021645	0,842	1,443983858	0,684	0,97	0,64
DaNs	BICD1	ENSG00000151746	0,738	0,64064816	0,476	0,93	0,6
DaNs	KAZN	ENSG00000189337	0,864	1,057962532	0,728	1	0,68
DaNs	MAP1B	ENSG00000131711	0,829	0,795301905	0,658	1	0,68
DaNs	PDE4D	ENSG00000113448	0,874	1,73363943	0,748	0,99	0,7
DaNs	FBXL17	ENSG00000145743	0,707	0,674476807	0,414	0,95	0,66
DaNs	ANKS1B	ENSG00000185046	0,707	0,641848242	0,414	1	0,75
DaNs	DOCK3	ENSG00000088538	0,719	0,616625413	0,438	0,96	0,76
DaNs	ADGRL3	ENSG00000150471	0,752	0,774304415	0,504	0,99	0,79
DaNs	LSAMP	ENSG00000185565	0,785	0,887660475	0,57	0,99	0,8
DaNs	LRP1B	ENSG00000168702	0,704	0,738664677	0,408	0,99	0,8
DaNs	CADM2	ENSG00000175161	0,751	0,714638904	0,502	1	0,83
DaNs	ERBB4	ENSG00000178568	0,705	0,952400165	0,41	0,88	0,72
DaNs	TNRC6A	ENSG00000090905	0,784	0,61695903	0,568	0,99	0,83
DaNs	ANK2	ENSG00000145362	0,773	0,774117771	0,546	0,99	0,84
DaNs	FTX	ENSG00000230590	0,74	0,625379613	0,48	1	0,93

myAUC Area under the curve
avg_diff Mean log2 expression difference
pct.1 Percentage of expressing cells of the query cell type
pct.2 Percentage of expressing cells of the all but the query cell type

Supplementary Table 4 PD-associated variant enrichment in genes with cell type-specific expression patterns

Control + IPD					
**Cell type**	**Ngenes**	**Beta**	**Beta SD**	**SE**	**P**
Astrocytes	288	0.1017	0.012776	0.057773	0.039178
CADPS2	135	0.036384	0.0031427	0.085945	0.33603
DaNs	3558	0.031252	0.012457	0.01794	0.040758
Ependymal	789	0.021755	0.0044587	0.034048	0.26143
Excitatory	3326	0.038489	0.014951	0.018401	0.018243
GABA	1944	0.0038755	0.0012041	0.022954	0.43296
Inhibitory	3653	0.032494	0.01308	0.017858	0.034424
Microglia	312	0.12692	0.016583	0.05239	0.00771
ODC	545	0.024234	0.0041571	0.040358	0.2741
OPC	346	0.093397	0.012838	0.055126	0.04512
Pericytes	763	0.04785	0.0096511	0.034697	0.083947
endothelial	425	-0.025419	-0.0038638	0.046468	0.70781
**IPD**					
**Cell type**	**Ngenes**	**Beta**	**Beta SD**	**SE**	**P**
Astrocytes	272	0.08916	0.010889	0.060035	0.068766
CADPS2	61	0.043147	0.0025103	0.12181	0.36159
DaNs	3298	0.0051904	0.0020097	0.018263	0.38813
Endothelial	418	-0.0079903	-0.0012047	0.047089	0.56737
Ependymal	811	0.057624	0.011966	0.033824	0.044233
Excitatory	3196	0.035544	0.013595	0.018639	0.028275
GABA	1217	0.022821	0.0057358	0.028402	0.21085
Inhibitory	3716	0.041095	0.016648	0.017732	0.010245
Microglia	335	0.12451	0.016846	0.050777	0.0071079
OPC	355	0.059049	0.0082196	0.054881	0.14098
ODC	610	0.018569	0.0033636	0.038035	0.31271
Pericytes	418	0.067611	0.010194	0.046413	0.072609
**Controls**					
**Cell type**	**Ngenes**	**Beta**	**Beta SD**	**SE**	**P**
Astrocytes	292	0.086154	0.010896	0.056941	0.065146
DaNs	177	0.023938	0.0023648	0.071795	0.36941
Endothelial	441	-0.028609	-0.0044278	0.045705	0.73432
Ependymal	635	0.05483	0.010126	0.037248	0.070521
Excitatory	2846	0.025134	0.0091787	0.019482	0.09851
GABA	2451	0.0060464	0.0020757	0.020663	0.38491
Inhibitory	3547	0.026985	0.010743	0.01801	0.067032
Microglia	254	0.065616	0.007748	0.058365	0.13047
OPC	347	0.10118	0.013928	0.054997	0.032914
ODC	481	0.019398	0.0031317	0.042521	0.32413
Pericytes	442	0.089193	0.013819	0.045315	0.024526

Ngenes: the number of genes
Beta: the regression coefficient of the variable
Beta SD: the semi-standardized regression coefficient, corresponding to the predicted change in Z-value given a change of one standard deviation in the predictor gene set
SE: the standard error of the regression coefficient
P: p-value for the variable

Supplementary table 5 Per-gene information for every significant cell type within the MAGMA enrichment analysis. See separate Excel file

Supplementary Table 6 T-test results for the cell type composition differences between IPD and control samples.

Cell type	mean cell/sample (control)	mean cell/sample (IPD)	Statistic	Parameter	P.value
Astrocytes	0.1003201348	0.1350897162	-2.750440529	5.671063782	0.03527224809
CADPS2+ neurons	0.0006756662773	0.00716654498	-4.143674419	3.08556866	0.02416713591
DaNs	0.002254081783	0.002534306896	-0.1628600317	4.926478174	0.8770978439
Endothelial cells	0.03712579331	0.03379641715	0.2551487087	6.405729353	0.8066043354
Ependymal	0.008691805579	0.01714801578	-1.763472461	6.174108097	0.1268821922
Excitatory	0.07705083796	0.06105230529	0.8389981942	8.563349355	0.4242711062
GABA	0.01057109988	0.01355919251	-0.6523500725	8.899491317	0.5306619457
Inhibitory	0.03591306145	0.03436051229	0.1246382728	8.849982953	0.9035949956
Microglia	0.05494681848	0.1293926796	-2.967776818	4.335692391	0.03727960072
OPCs	0.0717005494	0.06304498374	1.108974963	5.978794357	0.3100451593
Oligodendrocytes	0.5772245274	0.4762349461	2.004862556	6.698913849	0.08684078533
Pericytes	0.0247274284	0.0290674112	-0.5552624256	6.296609446	0.5978755141

Cell type: Midbrain cell type.
mean %cell/sample (control): Mean fraction of cells per sample for controls
mean %cell/sample (IPD): Mean fraction of cells per sample for IPD
Statistic: the value of the t-statistic.
Parameter: the degrees of freedom for the t-statistic.
P.value: the p-value of the test.

Supplementary Table 7 T-test results of imaging results

DaNs									
**Region**	**.y.**	**group1**	**group2**	**p-val**	**p.adj**	**p.format**	**p.signif**	**method**	**# samples**
SN	Area	Control	IPD	0.01164196	0.035	0.012	*	T-test	Control n=6; IPD n=5.

Neuromelanin									
**Region**	**.y.**	**group1**	**group2**	**p**	**p.adj**	**p.format**	**p.signif**	**method**	**# samples**
SN	Area	Control	IPD	0.006934968	0.0069	0.0069	**	T-test	Control n=6; IPD n=5.

Microglia									
**Region**	**.y.**	**group1**	**group2**	**p**	**p.adj**	**p.format**	**p.signif**	**method**	**# samples**
CC	Area	Control	IPD	0.60947311	1	0.609	ns	T-test	Control n=6; IPD n=5.
Midbrain	Area	Control	IPD	0.29191107	0.88	0.292	ns	T-test	
NR	Area	Control	IPD	0.61652105	1	0.617	ns	T-test	
SN	Area	Control	IPD	0.02375746	0.12	0.024	*	T-test	
TT	Area	Control	IPD	0.04090638	0.16	0.041	*	T-test	

Microglia ramification									
**Region**	**.y.**	**group1**	**group2**	**p**	**p.adj**	**p.format**	**p.signif**	**method**	**# samples**
SN	BranchPerc	IPD	Control	1,57E-32	6.30E-38	< 2e-16	****	T-test	Control n=5685; IPD n=6894.

Astrocytes									
**Region**	**.y.**	**group1**	**group2**	**p**	**p.adj**	**p.format**	**p.signif**	**method**	**# samples**
CC	Area	Control	IPD	0.6390098	1	0.64	ns	T-test	Control n=6; IPD n=5.
Midbrain	Area	Control	IPD	0.1077312	0.54	0.11	ns	T-test	
NR	Area	Control	IPD	0.5876515	1	0.59	ns	T-test	
SN	Area	Control	IPD	0.1085478	0.54	0.11	ns	T-test	
TT	Area	Control	IPD	0.2826889	0.85	0.28	ns	T-test	

Oligodendrocytes									
**Region**	**.y.**	**group1**	**group2**	**p**	**p.adj**	**p.format**	**p.signif**	**method**	**# samples**
CC	Area	Control	IPD	0.565291	1	0.565	ns	T-test	Control n=6l; IPD n=5.
Midbrain	Area	Control	IPD	0.1221451	0.49	0.122	ns	T-test	
NR	Area	Control	IPD	0.5709309	1	0.571	ns	T-test	
SN	Area	Control	IPD	0.0477596	0.24	0.048	*	T-test	
TT	Area	Control	IPD	0.8594471	1	0.859	ns	T-test	

CC: Crus cerebri
NR: Nucleus ruber
SN: Substantia nigra
TT: Tectum and tegmentum of the midbrain

Supplementary Table 8 Differentially activated genes along glial activation in IPD midbrain and differentially expressed genes in each cell type

The Supplementary Table 8 is stored at https://github.com/SpielmannLab/pd_human_midbrain_snrnaseq/blob/main/scrnaseq/data/Supplementary%20Table%208.xlsx.zip
